# Heat shock factor 2 is a stress-responsive mediator of neuronal migration defects in models of fetal alcohol syndrome

**DOI:** 10.15252/emmm.201303311

**Published:** 2014-07-15

**Authors:** Rachid El Fatimy, Federico Miozzo, Anne Le Mouël, Ryma Abane, Leslie Schwendimann, Délara Sabéran-Djoneidi, Aurélie de Thonel, Illiasse Massaoudi, Liliana Paslaru, Kazue Hashimoto-Torii, Elisabeth Christians, Pasko Rakic, Pierre Gressens, Valérie Mezger

**Affiliations:** 1 CNRS, UMR7216 Épigénétique et Destin CellulaireParis Cedex 13, France; 2 Univ Paris Diderot, Sorbonne Paris CitéParis Cedex 13, France; 3 ED 387 iViv, UPMC Univ Paris 06Paris, France; 4 Univ Paris DiderotParis Cedex 13, France; 5 INSERM, U1141, Hôpital Robert DebréParis, France; 6 Faculté de Médecine Denis Diderot, Univ Paris Diderot, Sorbonne Paris CitéParis, France; 7 INSERM, UMR 866Dijon, France; 8 Faculty of Medicine and Pharmacy, Univ BurgundyDijon, France; 9 Carol Davila University of Medicine and Pharmacy, Fundeni HospitalBucharest, Romania; 10 Department of Neurobiology and Kavli Institute for Neuroscience, Yale University School of MedicineNew Haven, CT, USA; 11 Laboratoire de Biologie du Développement de Villefranche-sur-mer, Observatoire Océanologique, CNRSVillefranche-sur-mer, France; 12 Sorbonne Universités, UPMC Univ Paris 06Villefranche-sur-mer, France

**Keywords:** fetal alcohol syndrome, heat shock factors, microtubule-associated proteins, radial neuronal migration, transcription

## Abstract

Fetal alcohol spectrum disorder (FASD) is a frequent cause of mental retardation. However, the molecular mechanisms underlying brain development defects induced by maternal alcohol consumption during pregnancy are unclear. We used normal and *Hsf2*-deficient mice and cell systems to uncover a pivotal role for heat shock factor 2 (HSF2) in radial neuronal migration defects in the cortex, a hallmark of fetal alcohol exposure. Upon fetal alcohol exposure, HSF2 is essential for the triggering of HSF1 activation, which is accompanied by distinctive post-translational modifications, and HSF2 steers the formation of atypical alcohol-specific HSF1–HSF2 heterocomplexes. This perturbs the *in vivo* binding of HSF2 to heat shock elements (HSEs) in genes that control neuronal migration in normal conditions, such as *p35* or the *MAPs* (microtubule-associated proteins*, such as Dclk1* and *Dcx*), and alters their expression. In the absence of HSF2, migration defects as well as alterations in gene expression are reduced. Thus, HSF2, as a sensor for alcohol stress in the fetal brain, acts as a mediator of the neuronal migration defects associated with FASD.

**Subject Categories** Development & Differentiation; Neuroscience

## Introduction

Fetal alcohol spectrum disorders (FASD) are caused by maternal consumption of alcohol during pregnancy and are the most frequent cause of non-genetic birth defects and mental retardation (Lemoine *et al*, 1968; Jones & Smith, 1973). The most severe clinical manifestations, referred to as fetal alcohol syndrome (FAS), include dysmorphic facial features, intrauterine growth defects, and brain lesions (reviewed in Gressens *et al*, 2001; Clarke & Gibbard, 2003). Children with more moderate phenotypes along the FASD continuum represent a real diagnostic challenge, with an estimated incidence of 1 per 100 live births in Western countries. Prenatal exposure to alcohol can affect fetal brain development at any point of gestation. Postmortem brains of FAS infants show reduced brain weight, disturbances in horizontal cortical lamination, neuronal ectopias, and/or a reduced thickness of the cortical mantle (Gressens *et al*, 2001; Clarke & Gibbard, 2003; Gibbard *et al*, 2003; Thompson *et al*, 2009; Valenzuela *et al*, 2012). Fetal alcohol exposure affects many aspects of brain cortical development, including the proliferation of neural progenitors and radial neuronal migration (Gressens *et al*, 2001; Zhou *et al*, 2001; Santillano *et al*, 2005; Rubert *et al*, 2006). However, the molecular mechanisms that trigger these alterations remain unknown.

Heat shock factors (HSF) were discovered as sensors and regulators of the cellular response to environmental proteotoxic stress (reviewed in Åkerfelt *et al*, 2010). These stressors include heat shock (HS), exposure to amino acid analogs, ethanol (EtOH), oxidative stress, heavy metals and pathophysiological situations, for example, fever, aging, or neuronal injuries. The heat shock response (HSR) in mammals is characterized by the transient activation of the latent transcription factor HSF1, which binds conserved heat shock elements (HSEs) within the regulatory region of heat shock genes (*Hsp*) and drives their transcription. Of the four HSFs (HSF1-4) that bind HSEs in mammals, HSF1 and HSF2 are co-expressed in many tissues and cell systems, including the brain (where HSF4 is not expressed, Abane & Mezger, 2010), and have received the most attention. HSF1 is the dominant HSF responsible for the HSR and cannot be replaced by any other HSF in mice or model cell systems. HSF2 is ineffective in triggering the HSR on its own and is quickly degraded by HS itself in cell models (Sarge *et al*, 1993; Sistonen *et al*, 1994; Ahlskog *et al*, 2010). However, HSF2 acts as a transient fine-tuner of the HSR through physical and functional interactions with HSF1, modulating the HSF1-dependent expression of *Hsp* genes either positively or negatively (He *et al*, 2003; Loison *et al*, 2006; Östling *et al*, 2007; Sandqvist *et al*, 2009).

In addition to their function in stress responses, HSFs also play critical roles in normal development (Abane & Mezger, 2010). Basal levels of HSF2 in the developing brain and in neural progenitors and migrating neurons are high. In particular, HSF2 influences brain cortical development through HSE-containing target genes that are distinct from *Hsp* genes (Rallu *et al*, 1997; Kallio *et al*, 2002; Wang *et al*, 2003). During mammalian corticogenesis, post-mitotic neurons are generated from dividing neural progenitors in the innermost part of the developing cortex (the ventricular zone, VZ), from which they migrate radially to more external positions. Radial neuronal migration results in the formation of a six-layer cortex (reviewed in Ayala *et al*, 2007). Among the actors that control this process, the signaling pathway of Cdk5, a kinase under the control of two activators, p35 and p39, is essential for neuronal migration. A number of genes that are involved in radial neuronal migration, such as those encoding some microtubule-associated proteins (MAPs) such as *Dcx* (*Doublecortin*) and its homolog *Dclk1* (*Doublecortin-like kinase 1*), play crucial roles in corticogenesis in humans, and their mutations can cause devastating pathologies, the type I lissencephalies (Francis *et al*, 2006; Ayala *et al*, 2007; Wynshaw-Boris, 2007; Reiner & Sapir, 2009; Alkuraya *et al*, 2011).

We have previously shown that HSF2 influences radial neuronal migration by modulating the expression of *p35* and *p39*, thereby fine-tuning the activity of Cdk5 (Chang *et al*, 2006). Since components of the HSR are induced *ex vivo* in rodent postnatal neurons or human fetal cortices by acute alcohol exposure (Pignataro *et al*, 2007; Hashimoto-Torii *et al*, 2011), as well as *in vivo* upon prenatal alcohol exposure (Hashimoto-Torii *et al*, 2014), we asked whether chronic alcohol exposure could affect HSF activity in developing neural tissue. We show that HSF1 and HSF2 are both activated by chronic prenatal alcohol exposure in the cortex *in vivo*, thereby leading to disturbances in the expression of HSF2 target genes that control proliferation and/or migration. Furthermore, using cellular and FAS mouse models, we uncover a prominent role for HSF2 in the developing cortex in response to alcohol, wherein HSF2 is essential for HSF1 activation upon EtOH exposure and drives the formation of EtOH-specific HSF1–HSF2 heterotrimers with a distinctive HSF1 post-translational modification profile. These alterations in the activities of HSF1 and HSF2 perturb the expression of *p35* and *MAP* genes, including *Dcx* and *Dclk1*, that control radial neuronal migration. Accordingly, both neuronal positioning abnormalities and abnormal *p35* and *MAP* gene expression elicited by alcohol exposure are less severe in the absence of HSF2. This indicates that HSF2, which fine-tunes radial neuronal migration under normal conditions, mediates defects that are characteristic of FAS, upon fetal alcohol exposure.

## Results

### Choice of FAS paradigm

**Figure 1:**
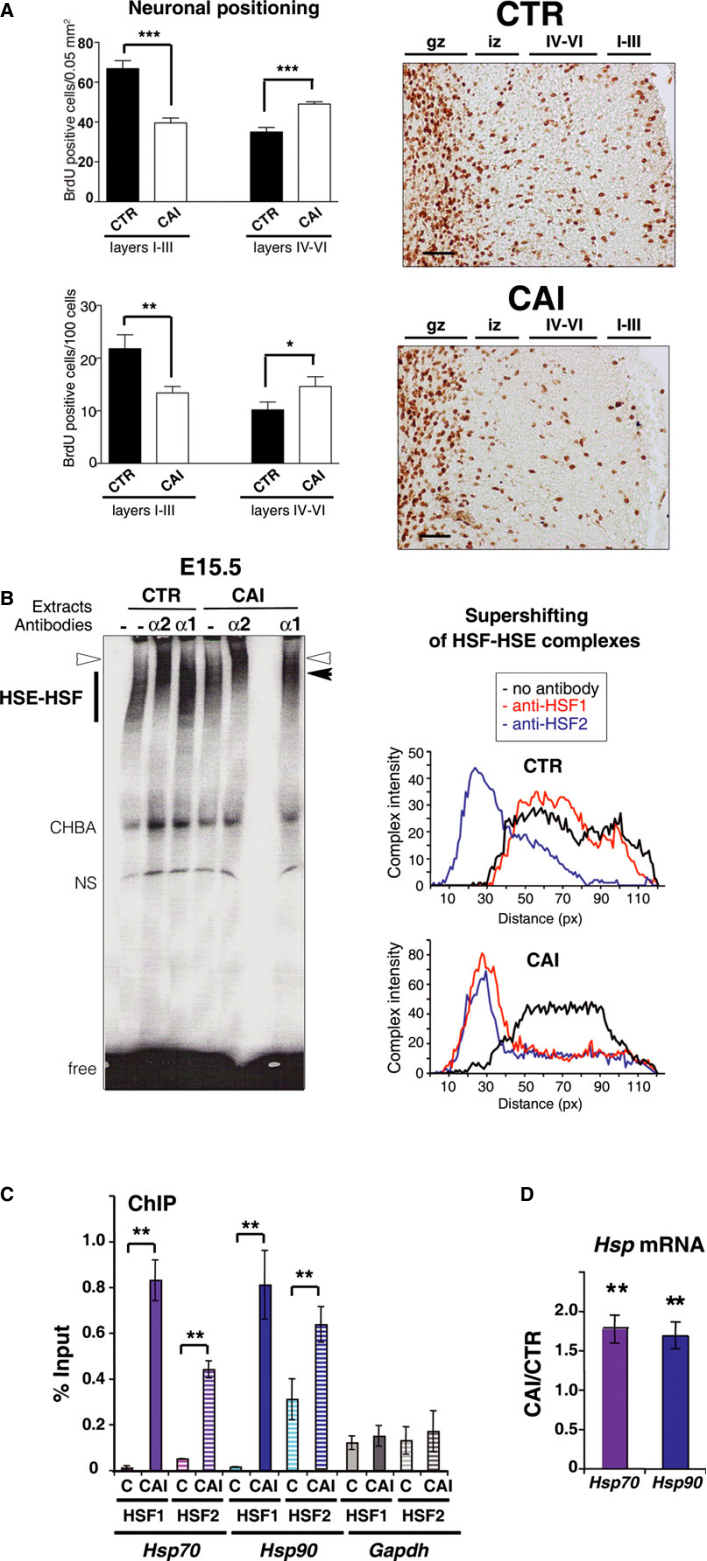
HSF2 remains active and HSF1 is activated *in vivo* by fetal alcohol exposure in the developing brain A   CAI disturbs neuronal positioning in the outer cortical layers (I–III). (Left) BrdU-labeled cells (BrdU injection at E16.5 and neuronal positioning examined at P0) in fetal cortices from embryos of pregnant dams chronically intoxicated with food containing EtOH *ad libitum* (CAI), per 0.05 mm^2^ (*n* = 5 sections from five different individuals for each conditions; upper panel; *P* = 0.0004 for layers I–III and *P* = 0.0006 for layers IV–VI) or per 100 cells (lower panel; *P* = 0.01 and *P* = 0.05). Superficial layers, I–III; deep layers, IV–VI; *P ≤ 0.05; **P ≤ 0.01; ***P < 0.001. Differences were considered statistically significant when *P*-values were ≤ 0.05, using unpaired two-way Student's *t*-test. Data are presented as mean ± SEM (Right) representative images corresponding to panels on the left. Gz, germinal zones; iz, intermediate zone; IV–VI and I–III, deep and superficial layers, respectively. Scale bars: 10 μM. B   Fetal cortices exposed to alcohol display both HSF1 and HSF2 DNA-binding activities. Gel-shift analysis of HSF1 and HSF2 DNA-binding activity in cortices at E15.5. (Left) The presence of HSF1 and/or HSF2 in the HSF–HSE complex analyzed by supershifting (arrowhead) with anti-HSF1 (α1; black arrowhead) or anti-HSF2 antibodies (α2; white arrowhead). HSF–HSE: HSF–HSE complexes. CHBA: constitutive HSE-binding activity, which is not carried by HSFs and is very variable from one sample or condition to another (Mosser *et al*, 1988; Abravaya *et al*, 1991); NS, non-specific DNA–protein complex; free: unbound HSE oligonucleotides. Each lane corresponds to three pooled cortices. (right) Quantification of the intensity and supershifting of HSF–HSE complexes. C HSF1 and HSF2 occupy the *Hsp70* and *Hsp90* gene promoter region upon CAI *in vivo*. ChIP experiments in fetal cortices from control (C) or chronically intoxicated dams with EtOH (CAI). The occupancy of *Hsp70* and *Hsp90* gene promoter regions by HSF1 or HSF2, was quantified by quantitative PCR analysis by ratio of the ChIP signal versus input signal. *Gapdh* was used as a negative control. Quantification was carried out in cortices from *n* = 6 independent litters for CAI and *n* = 3 independent litters for controls. Hatched bars: ChIP with anti-HSF2. Solid bars: ChIP with anti-HSF1. *P*-values: **P ≤ 0.01. For *Hsp70*: *P* = 0.001482 for HSF1 enrichment in CAI compared to CTR and *P* = 0.000686 for HSF2. *P*-values for *Hsp90*: *P* = 0.001517 for HSF1 enrichment in CAI compared to CTR and *P* = 0.000338 for HSF2. No significant *P*-values for *Gapdh*: *P* = 0.176985 for HSF1 enrichment in CAI compared to CTR and *P* = 0.195512 for HSF2. Differences were considered statistically significant when *P*-values were < 0.05, using unpaired two-way Student's *t*-test. Data are presented as mean ± SEM. D   Quantitative RT-PCR analysis of the *Hsp70* and *Hsp90* mRNAs. Ratio between levels of chronically intoxicated (CAI) embryonic cortices versus control cortices (C); *n* = 8 independent litters for each gene. *P*-values: **P ≤ 0.01. For *Hsp70*, *P* = 0.003345; for *Hsp90*, *P* = 0.007810. Color code: purple for *Hsp70* and blue for *Hsp90* in all Figures. Differences were considered statistically significant when *P*-values were < 0.05, using unpaired two-way Student's *t*-test. Data are presented as mean ± SEM. See also Supplementary Figs S1 and S2. Source data is available for this figure.

We tested three protocols of chronic fetal alcohol exposure that induce FAS-like brain defects in rodent fetuses (Gressens *et al*, 1992; Ikonomidou *et al*, 2000; Olney *et al*, 2002; Carloni *et al*, 2004; Ieraci *et al*, 2006, 2007)—chronic intraperitoneal injections (CIP), chronic alcohol intoxication with alcohol-containing food (CAI), and daily gavage (GAV)—to ascertain that they affected neuronal migration/positioning at stages that normally exhibit HSF2-dependent radial neuronal migration (E15.5 to E18.5; Chang *et al*, 2006). All three paradigms affected BrdU incorporation in the germinal zones at E16.5 to a similar extent (Supplementary Fig S1A). In addition, in CAI, the protocol that causes the least handling stress to the dams (Gressens *et al*, 1992), neuronal positioning in fetal cortices, as indicated by BrdU birth-dating experiments, confirmed that radial neuronal migration was compromised (Fig 1A). We therefore used this paradigm for most subsequent experiments.

### Fetal alcohol exposure leads to HSF1 activation and keeps HSF2 active in the fetal cortex

We then asked whether the HSFs were activated by EtOH exposure in fetal cortices *in vivo*. We verified that HSF1 is present throughout brain development (Supplementary Fig S1B). In control conditions, cortical extracts from E15.5 showed constitutive HSE-binding activity in gel-shift assays (Fig 1B) that was supershifted by anti-HSF2 antibodies (white arrowhead and blue curves) but not by anti-HSF1 antibodies, demonstrating that HSF2 is the main contributor to this activity and that HSF1 is mostly inactive for HSE binding under control conditions. These results are in accordance with our previous findings (Rallu *et al*, 1997; Chang *et al*, 2006). In contrast to control conditions, CAI induced HSF1 DNA-binding activity, as detected by the supershifting of the HSE–HSF complex by anti-HSF1 antibodies (Fig 1B; black arrowheads and red curves). Moreover, CAI increased the nuclear localization of HSF1 in the cortical plate (Supplementary Fig S2A and B) and the nuclear amount of HSF1 phosphorylated on residue Ser326, a hallmark of its activation (Supplementary Fig S2C; Guettouche *et al*, 2005). HSF1-binding activity was also induced by CIP (in line with Hashimoto-Torii *et al*, 2014) or daily gavage (Supplementary Fig S1D). Strikingly, although we expected that HSF2 would be inactivated by chronic EtOH exposure as for classic HS (Sarge *et al*, 1993; Ahlskog *et al*, 2010), HSF2 was seen to be bound to HSE under these conditions, as was HSF1 (CAI: Fig 1B, white arrowheads and blue curves; GAV and CIP: Supplementary Fig S1D). We then confirmed the concomitant presence of HSF1 and HSF2 DNA-binding activities in fetal cortices after CAI by chromatin immunoprecipitation (ChIP) experiments: both HSF1 and HSF2 occupied the promoters of the typical heat shock genes *Hsp70* and *Hsp90* in embryonic cortices after CAI (Fig 1C). In addition, quantitative RT-qPCR experiments demonstrated that this *in vivo* binding was accompanied by a significant induction of *Hsp70* and *Hsp90* transcription (1.78 and 1.70 fold, respectively; *P* < 0.005; *n* = 8; Fig 1D).

Thus, chronic alcohol exposure changes HSF DNA-binding activities in the fetal cortex by maintaining that of HSF2 and inducing that of HSF1 and consequently leads to the transcriptional induction of *Hsp* genes. The increase in transcription was, however, lower than upon typical HS, in line with data from mouse and human fetal cortices exposed to alcohol *ex vivo* (Hashimoto-Torii *et al*, 2011; Hashimoto-Torii *et al*, 2014).

### CAI perturbs HSF2 binding to genes involved in neuronal migration and their expression

We next tested whether these changes in HSF-binding activities in fetal cortices upon CAI could deregulate HSF2 target genes important for radial neuronal migration, such as *p35* (Chang *et al*, 2006), and thus impact radial neuronal migration.

We began by searching for HSEs in genes involved in the control of radial neuronal migration (Ayala *et al*, 2007). We identified HSEs that were conserved among species in the *Dcx* and *Dclk1* genes by bioinformatic analyses using Genomatix software (Supplementary Fig S3).

**Figure 2:**
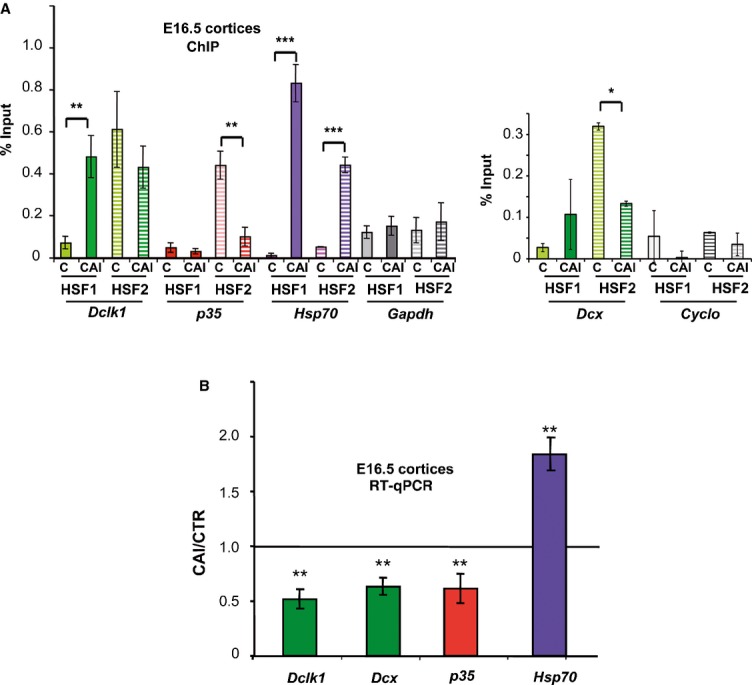
Alcohol affects HSE occupancy by HSF1-HSF2 and expression of genes that control neuronal migration A   Quantification of the occupancy of HSE by HSF1 or HSF2 using ChIP and quantitative PCR (ratio of the ChIP signal versus input signal) on *Dcx*, *Dclk1*, *p35* and *Gapdh* or *Cyclophiline B* genes in E16.5 fetal cortices from control dams (C) or those subjected to CAI (CAI); for *Dclk1*, *p35* and *Gapdh, n* = 6 independent litters for CAI; *n* = 3 independent litters for controls; for *Dcx and Cyclophiline B*, *n* = 2 independent litters for C and *n* = 3 for CAI. Hatched and solid bars: ChIP with anti-HSF2 and anti-HSF1, respectively. Color code for gene families: *Dcx* and *Dclk1* (green), *p35* (red). *P*-values for *Hsp70*: *P* = 0.001482 for HSF1 enrichment in CAI compared to C, and *P* = 0.000686 for HSF2; for *Dclk1*: *P* = 0.00364 for HSF1 and no significant *P*-value for HSF2 (*P* = 0.145678); for *p35*: *P* = 0.00431 for HSF2, no significant *P*-values for HSF1 (*P* = 0.151345); for *Dcx*: *P* = 0.03345 for HSF2, no significant *P*-value for HSF1 (*P* = 0.102345) for HSF1. ***P ≤ 0.001; **P ≤ 0.01; *P ≤ 0.05. For *Dcx* and *p35*: significant reduction in (or no) enrichment for HSF2 in CAI compared to C; no significant enrichment for HSF1*,* respectively; no enrichment of HSF2 or HSF1 for *Gapdh* (*P* = 0.176985 for HSF1 and *P* = 0.195512 for HSF2) and *Cyclo* (*P* = 0.162546 for HSF1 and *P* = 0.186584 for HSF2). Differences were considered statistically significant when *P*-values were < 0.05, using unpaired two-way Student's *t*-test. Data are presented as mean ± SEM. B   Quantitative RT-PCR analysis of mRNAs fo*r Dcx, Dclk1, p35,* and *Hsp70*. Ratio of levels in chronically intoxicated versus control embryonic cortices [*n* = 8 independent litters for each gene, except *p35* (*n* = 6)]. Note that these genes were affected by more than 20%, a cutoff that we used to distinguish non-affected to affected genes. Asterisk: ratio significantly different from 1. *P*-values: **P ≤ 0.01. For *Hsp70*, *P* = 0.003345; for *Dclk1*, *P* = 0.001649; for *Dcx*, *P* = 0.0006864; for *p35*, *P* = 0.0053387. Same color code as in Fig 1C and D and 2A. Differences were considered statistically significant when *P*-values were < 0.05, using unpaired two-way Student's *t*-test. Data are presented as mean ± SEM. See also Supplementary Figs S3 and S4.

Next, we showed using ChIP that the HSEs identified in *Dclk1 and Dcx* were bound *in vivo* by HSF2 in control E16.5 fetal cortices, as previously shown for *p35* (Fig 2A; Chang *et al*, 2006). However, under CAI conditions, we observed two distinct situations; (i) In the case of *p35*, neither HSF1 nor HSF2 was bound to the HSE (Fig 2A, left panel, red plots; Chang *et al*, 2006), as for *Dcx*, in a lesser extend (reduction in HSF2 and no HSF1 enrichment on the HSE; Fig 2A, right panel, green plots); (ii) but both HSF1 and HSF2 occupied the HSEs of *Dclk1* (Fig 2A, left panel, green plots).

We then investigated whether the switch in the occupancy of HSEs by the HSFs under CAI conditions resulted in perturbations in the expression of HSF2 target genes. Interestingly, in independent qRT-PCR experiments (*n* = 8 litters; *P* < 0.01), we observed a significant downregulation by CAI of *Dclk1* gene expression in E16.5 cortices (Fig 2B), even though both HSF1 and HSF2 were found to bind to the *Dclk1* HSE in ChIP experiments. This was not unexpected, given the fact that HSF1 and HSF2 can also negatively regulate genes (Östling *et al*, 2007; Mendillo *et al*, 2012). In keeping with the CAI-induced reduction of HSF2 binding and HSF1 binding to the *p35* and *Dcx* HSE, the levels of the mRNA for these genes were also reduced (Fig 2B). In case of *p35*, this reduction in mRNA levels resembled that observed in the fetal cortices of *Hsf2* knockout mice (Chang *et al*, 2006), an observation that we also made regarding its protein levels (Supplementary Fig S4).

Fetal alcohol exposure thus disturbs the expression of genes important for cortical neuronal migration, possibly explaining the radial migration defects seen in FAS. Our data also suggest that these defects could be mediated by changes in the activities of HSF1 and HSF2, and their potential interplay.

### Alcohol induces persistent activity and post-translational modifications of HSF1

We have shown above that HSF1 displayed strikingly persistent DNA-binding activity in fetal cortices upon chronic alcohol exposure, as shown by both gel-shift assays and ChIP experiments (Figs 1B and C, 2A). This is unexpected because, even in the case of prolonged HS, heat-induced HSF1 activation is transient (Kline & Morimoto, 1997). Such persistent HSF1 DNA-binding activity could mediate EtOH-induced disturbances in the expression of genes involved in migration. To investigate this possibility, we used two cellular models: the neuroblastoma cell line Neuro2A (N2A) as a model of neural cells, because it displays constitutive HSF2-binding activity under normal conditions, similar to fetal cortices, and immortalized mouse embryonic fibroblasts (iMEFs). In both iMEFs (Supplementary Fig S5A) and N2A cells (Supplementary Fig S6A), we observed that EtOH exposure induced HSF1 and HSF2 DNA-binding activity, as it did in the fetal cortex. In addition, prolonged EtOH exposure led to persistent HSF1 DNA-binding activity *ex vivo* in these cells (Supplementary Figs S5D, E and S6A), as in fetal cortices chronically exposed to alcohol (Fig 1B and C; see also Hashimoto-Torii *et al*, 2014). A moderate but prolonged increase in HSF transcriptional activity was observed using *Hsp70*-*Luciferase* reporter assays in N2A cells, in contrast to the potent but transient induction characteristic of HS (Supplementary Fig S6B). We also observed a moderate but reproducible induction of *Hsp70* and *Hsp90* mRNA levels in N2A cells (2–4-fold; Supplementary Fig S6C) as it was in iMEFs (Supplementary Fig S5F) and fetal cortices upon CAI (Fig 1D).

**Figure 3:**
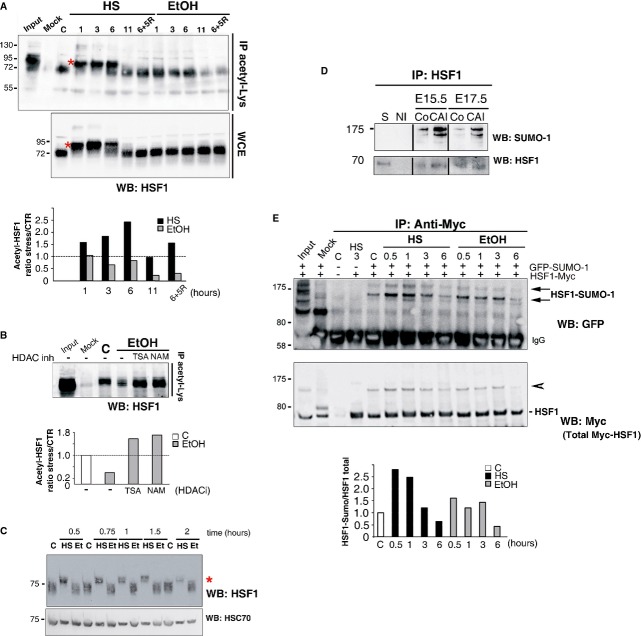
Persistent alcohol-induced HSF1 activation correlates with alcohol-specific post-translational modifications A   Acetylation of HSF1 is observed upon HS, but not EtOH exposure. (Upper panel) Representative immunoprecipitation of the acetylated form of endogeneous HSF1 detected with an anti-acetyl-lysine antibody in N2A cells (IP acetyl-Lys). HS (42°C) or EtOH exposure (0.5%) was carried out for 1–11 h, or for 6 h followed by 5 h of recovery (6+5R). (Middle panel) Immunoblot of HSF1 in whole cell extracts (WCE). C, untreated cells; mock: immunoprecipitation in the absence of specific antibodies. Input: HS, 3 h. The shift in HSF1 migration in HS samples is due to its typical heat-induced hyperphosphorylation [red asterisk; see also (C)]. (Lower panel) Corresponding quantification of the relative levels of acetylated HSF1 after HS or EtOH exposure compared to control conditions. B   HDAC inhibition by TSA or NAM restores HSF1 acetylation upon EtOH stress. (Upper panel) Immunoprecipitation as in (A). (Lower panel) Corresponding quantification of the relative levels of acetylated HSF1 before and after EtOH exposure, with or without HDAC inhibitors (TSA and NAM). C   Lack of HSF1 hyperphosphorylation in response to EtOH. Immunoblot of HSF1 in a 7% SDS–PAGE. iMEFs were analyzed at different times following HS at 41°C or 2% EtOH (Et) exposure. Hsc70: loading control. Red asterisk, shifted hyperphosphorylated HSF1 form. D   HSF1 is sumoylated upon chronic fetal alcohol exposure in cortices. Immunoprecipitation of HSF1 from control fetal cortices at E15.5 or E17.5 (Co) or after CAI and immunoblotting with anti-SUMO-1 (upper panel) or anti-HSF1 antibodies (lower panel). S: supernatant (E15.5). NI: non-immune serum. E   Limited HSF1 sumoylation in response to EtOH. (Upper panel) Immunoprecipitation of Myc-tagged human HSF1 (HSF1) and immunoblot analysis of HSF1 sumoylation. N2A cells, cotransfected with Myc-tagged human HSF1 and GFP-fused SUMO-1 expression vectors, were exposed to a 42°C HS or to 0.75% EtOH. Arrows point to sumoylated HSF1 forms. (Middle panel) Total Myc-tagged HSF1; the arrowhead points to HSF1-Myc forms that likely correspond to the sumoylated forms, according to their molecular weight. Input and mock (immunoprecipitation using anti-HA antibody) are shown for control (C) N2A cells. (Lower panel) Corresponding quantification of the ratio between the sumoylated form of HSF1 and total HSF1 amounts. See also Supplementary Figs S5, S6, and S7. Source data is available for this figure.

We next analyzed whether post-translational modifications (PTMs) that accompany heat-induced HSF1 activation and are involved in the attenuation of its DNA-binding and transcriptional abilities, such as HSF1 acetylation (Westerheide *et al*, 2009; Raychaudhuri *et al*, 2014), are operational in these cell models. We detected an increased (1.8-fold at 3 h) and prolonged acetylation of HSF1 in N2A cells exposed to HS and during recovery (Fig 3A). In contrast, EtOH provoked a marked decrease in HSF1 acetylation upon long-term exposure (0.6-fold at 3 h; Fig 3A). The incubation of N2A cells with TSA (an inhibitor of class I/II histone deacetylases (HDACs)) or with NAM (an inhibitor of class III HDACs, also known as sirtuins) restored HSF1 acetylation after EtOH treatment (Fig 3B). These results suggest that HDACs might keep HSF1 deacetylated and competent for DNA binding even during prolonged EtOH exposure. In line with this observation, a transient increase in HDAC1 and sirtuin 1 (SIRT1) levels was observed upon EtOH exposure (Supplementary Fig S6D). We detected a constitutive level of HSF1 acetylation in non-stressed N2A cells (Fig 3A and B), which might correspond to basal constitutive HSF1 DNA-binding activity in N2A cells (Supplementary Fig S6A) (Westerheide *et al*, 2009; Raychaudhuri *et al*, 2014).

The heat-induced transcriptional activity of HSF1 is controlled by phosphorylation and sumoylation. The classic HS-induced hyperphosphorylation of HSF1 is typically characterized by a shift in the mobility of HSF1 protein in SDS–PAGE (Sarge *et al*, 1993; Supplementary Fig S6E) and potentially associated with its inactivation (see Discussion). EtOH exposure did not induce HSF1 hyperphosphorylation in N2A cells, in contrast to HS in N2A cells (Fig 3A, asterisk) and iMEFs (Fig 3C, asterisk). Similarly, the shift typical of HSF1 hyperphosphorylation was not detected in fetal cortices upon CAI (Fig 3D, lower panel). Thus, hyperphosphorylation events that accompany HSF1 activation by HS are lacking upon EtOH exposure *in vivo* and *ex vivo*.

The sumoylation of HSF1 by SUMO-1, which is dependent on its phosphorylation at Ser303/307, is a mark of its activation and provides buffering capacity by repressing its transactivational activity (Hietakangas *et al*, 2003). We detected an increase in HSF1 sumoylation in E15.5 and E17.5 cortices upon CAI, as indicated by the very slow migrating form (around 175 kDa) detected with an anti-SUMO-1 antibody after HSF1 immunoprecipitation (Fig 3D, upper panel). In N2A cells, moderate HSF1 sumoylation was induced by EtOH, but notably less than by HS (1.7 × less at 30 min and 2.0 × less at 1 h; Fig 3E). Consistent with these results, the phosphorylation of HSF1 on Ser303/307 in iMEFs and N2A cells upon EtOH exposure was limited compared to that induced by HS (Supplementary Fig S6E and F). Furthermore, we observed delayed but persistent HSF1 and SUMO-1 colocalization upon EtOH exposure in the nuclear stress bodies typical of human cells (nSBs; Supplementary Fig S7; Sarge *et al*, 1993; Jolly *et al*, 1999; Hietakangas *et al*, 2003).

Altogether, these results show that EtOH exposure triggers HSF1 activation in a manner distinct from that induced by HS, via a specific pattern of post-translational modifications: reduced acetylation, lack of hyperphosphorylation, and reduced (or delayed) but prolonged sumoylation. This EtOH-induced signature is compatible with the persistent and modest activation of HSF1 (as deduced by its DNA-binding and transcriptional abilities), and, importantly, likely sustains the potential role of HSF1 in the fetal cortex subjected to chronic alcohol exposure.

### Alcohol induces specific HSF1–HSF2 interactions

**Figure 4:**
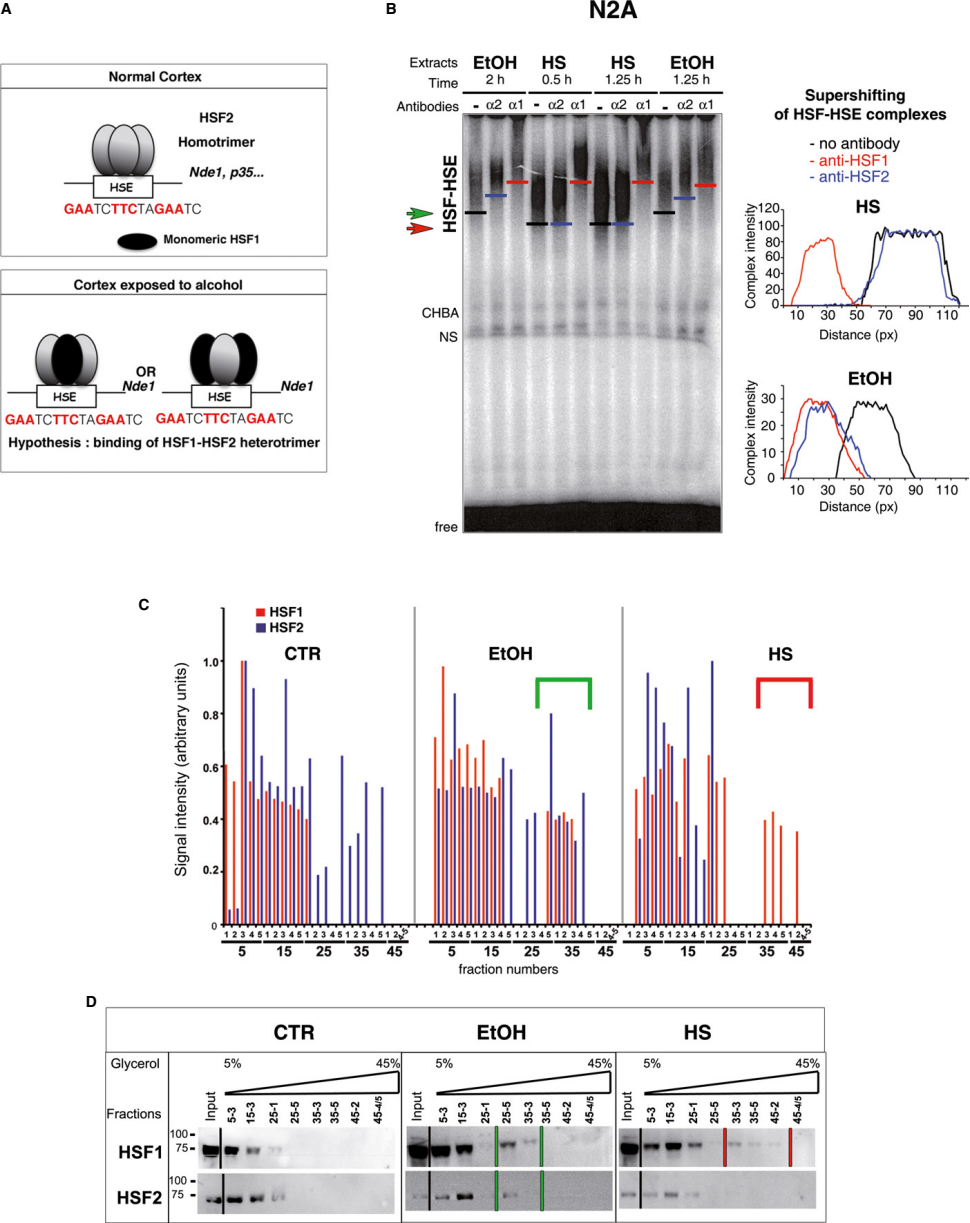
Alcohol induces the formation of HSF1–HSF2 heterocomplexes with specific signatures A   Hypothesis of HSF binding to the HSE of the *Nde1* gene in alcohol-exposed fetal cortices. Control situation: The *Nde1* HSE is bound by one HSF2 homotrimer (gray ellipses; HSF2 is known to be active in a trimeric form, Sistonen *et al*, 1994). HSF1 is inactive and monomeric (black ellipse). CAI: The *Nde1* HSE is bound by a single alcohol-specific HSF1–HSF2 heterotrimer. In the heterotrimer, the stoichiometric ratio could be 1 HSF1 monomer to 2 HSF2 monomers, or 2 HSF1 monomers to 1 HSF2 monomer. B   Representative gel-shift assays of extracts from N2A cells exposed to 0.5% EtOH or HS (41°C). The position of each HSF–HSE complex is indicated by one bar. Green arrow, EtOH-induced complex; red arrow, HS-induced complex. The specific supershift induced by the addition of anti-HSF1 (α1) or anti-HSF2 antibodies (α2) was illustrated by red and blue bars, respectively (no antibody: black bars). Right panels, relative positions of the supershifted bands after scan quantification of the HSE–HSF complexes (black curve, no antibody; red curve, with anti-HSF1 antibody; blue curve, with anti-HSF2 antibody). See also Supplementary Fig S5. C   Quantification of the immunoblot signals (arbitrary units) of HSF1 and HSF2 in all fractions after ultracentrifugation on a glycerol gradient. HSF1 (red); HSF2 (blue). Green bracket points to fractions in which HSF1 and HSF2 colocalize upon EtOH exposure. Red bracket represents the trimeric HSF1 forms induced by HS. D   Immunoblot of representative fractions collected as in (C). N2A cells were untreated (CTR), treated with 0.5% EtOH for 5 h, or heat-shocked at 42°C for 2 h. An example of the location of HSF1 and 2 is illustrated here for a representative number of fractions. The presence of HSF1 within fractions of high glycerol concentration is indicated by two bars (red for HS, corresponding to red brackets in (C). The presence of HSF1 and HSF2 in the same high glycerol concentration fractions is indicated by green bars for EtOH), corresponding to green brackets in (C). Note that this WB did not allow to HSF2 to be visualized in denser fractions from control N2A cells, which are presented in Fig 5C and correspond to the fact that HSF2 exhibits basal DNA-binding activity under control conditions in N2A cells. See also Supplementary Figs S8 and S9. Source data is available for this figure.

An important observation made above was that HSF2 activity was also sustained following EtOH exposure (Fig 1B and C and Supplementary Fig S6A). In ChIP experiments, we observed that CAI triggered the binding of both HSF1 and HSF2 to the HSEs of target genes that could only be occupied by one trimer (Fig 2A and Supplementary Fig S3). This could have resulted from the binding of HSF1 to a given HSE in some cells of the fetal cortex while HSF2 bound the same HSE in other cells, or alternatively, by the binding of both HSF1 and HSF2 to the HSE under consideration in the same cell. We observed that chronic FAE in cortices *in utero*, as well as *ex vivo* alcohol exposure of various cell systems activated both HSF1 and HSF2, as assessed by total supershifting of the HSF–HSE complex by either anti-HSF1 or anti-HSF2 antibodies in gel-shift assays, using a HSE probe that could bind only one trimer (Fig 1B, Supplementary Figs S5A, S6A and S8). Using this, we observed that in fetal cortices exposed to CAI *in utero*, as well as in various cell systems exposed to alcohol *ex vivo* (including F9 embryonic carcinoma cells, in which, as in the developing cortex, HSF2 displays high constitutive DNA-binding activity, but HSF1 does not; Rallu *et al*, 1997; Supplementary Fig S8), both HSF1 and HSF2 were activated, as assessed by the virtually complete supershifting of the HSF–HSE complex by either anti-HSF1 or anti-HSF2 antibodies in gel-shift. In addition, another gene involved in neuronal migration, *Nde1,* possesses one HSE that can accept only one trimer and is bound by HSF1 and HSF2 (Supplementary Fig S8C). This also suggests that HSF1 and HSF2 form part of the same HSF–HSE complex and that their potential effects on HSF2 target genes might be exerted by heterotrimers (see Fig 4A for a working model).

**Figure 5:**
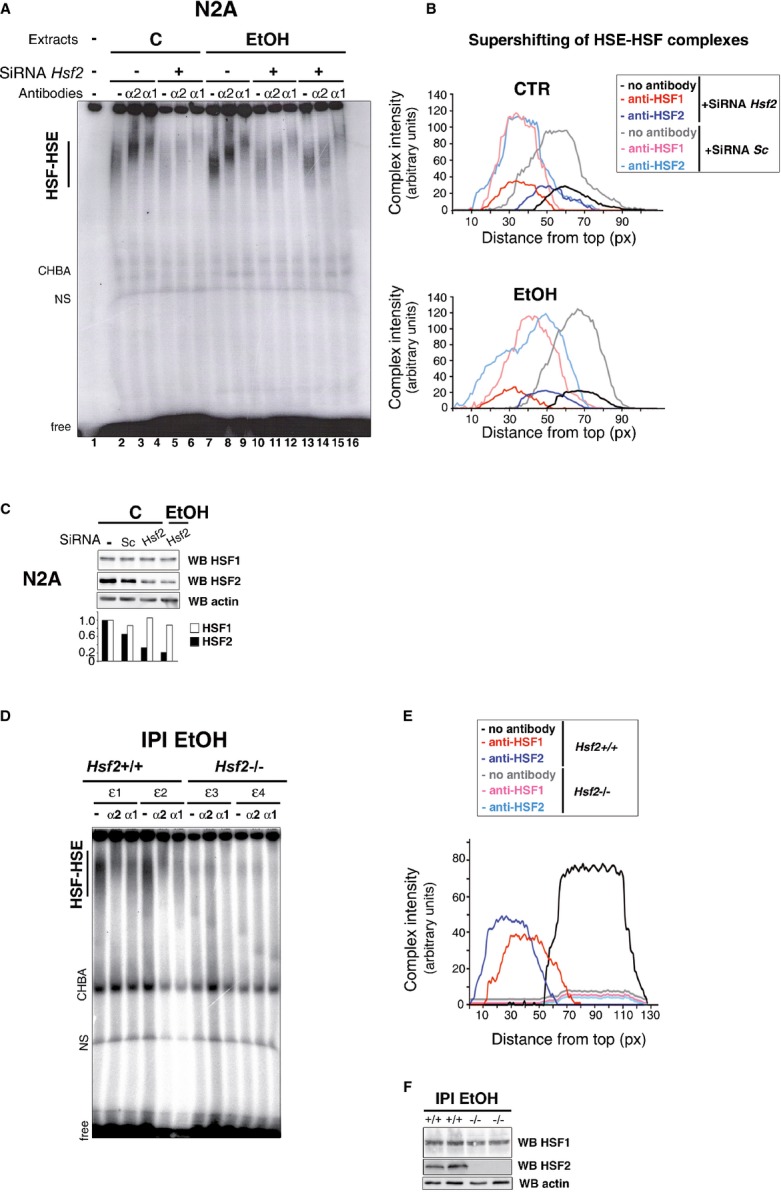
HSF2 is essential for the binding of HSF1 to HSE upon EtOH exposure A   Gel-shift analysis of the impact of *Hsf2* siRNA (+) or scrambled siRNA (−) on the migration of the HSF complex upon EtOH exposure (0.5 h at 2%). Lanes 11–13 and 14–16 represent duplicates, and the (EtOH+SiRNA *Hsf2*) curves in (B) correspond to their average values. B   Quantification of the intensity and supershifting of the HSF–HSE complexes corresponding to (A). C   Representative immunoblot of HSF1 and HSF2 levels in N2A cells after HSF2 silencing by two distinct *Hsf2* siRNAs or scrambled siRNA (Sc) in control (C) or stressed N2A cells. D   Representative gel-shift analysis of cortical extracts from individual *Hsf2*^+/+^ (ε1 and ε2) or *Hsf2*^−/−^ (ε3 and ε4) E18.5 fetuses, 2 h after EtOH (3 g/kg) intraperitoneal injection (IPI) of the mother. IPI was chosen in this case, to circumvent frequent fetal death of *Hsf2*^−/−^ embryos that frequently occurred in CAI. Note that HSE–HSF complexes are totally supershifted by either anti-HSF1 or anti-HSF2 antibody in extracts from *Hsf2*^+/+^ fetuses. E   Quantification of the intensity and supershifting of the HSF–HSE complexes corresponding to (D). F   Representative immunoblot of the levels of HSF1 and HSF2 in *Hsf2*^+/+^ and *Hsf2*^−/−^ embryonic cortices upon EtOH IPI. See also Supplementary Fig S10A. Source data is available for this figure.

We therefore analyzed the oligomeric status of HSF2 and HSF1 upon alcohol exposure by chemical cross-linking with EGS, followed by Western blotting. Trimers clearly appeared as a major oligomeric species upon HS or EtOH treatments (Supplementary Fig S9A and B). Importantly, the presence of both HSF1 and HSF2 in alcohol-induced trimers was also observed in fetal cortices exposed to CAI *in utero* (Supplementary Fig S9C).

The HSF1–HSF2 heterotrimers induced by EtOH displayed a characteristic signature: In the absence of antibodies, the migration of the HSE–HSF complexes formed in extracts of EtOH-treated N2A cells was reproducibly distinct from that of HS-induced complexes (Fig 4B). Similar distinctive complex migration characteristics in gel-shift assays were observed in other types of cells treated with EtOH (like in F9 cells, Supplementary Fig S8 A and B), but have never been detected under non-EtOH stress conditions, even upon proteasome inhibition or hemin treatment, which activate both HSF1 and HSF2 and lead to the formation of heterocomplexes (Loison *et al*, 2006; Östling *et al*, 2007). The formation of unusual HSF1–HSF2 heterotrimers upon EtOH exposure was confirmed by ultracentrifugation of N2A extracts on a glycerol gradient, where heterotrimers induced by EtOH exposure were localized in significantly less dense glycerol fractions (25–5 to 35–4; illustrated by green brackets in Fig 4C and green lines in Fig 4D) than HS-induced HSF1 trimers (fractions 35–3 to 45–2; red brackets and red lines).

Together, these gel-shift assays, ChIP, EGS cross-linking, and ultracentrifugation experiments support the formation of atypical HSF1–HSF2 heterotrimers upon alcohol exposure that could eventually mediate the disturbances in HSF2 target gene expression in fetal cortices.

### HSF2 is required for the alcohol-induced DNA-binding activity of HSF1

Since HSF2 levels and activity were unexpectedly preserved upon EtOH exposure, we analyzed the role of HSF2 in the formation of the atypical EtOH-induced HSF1–HSF2 heterotrimers. The specific downregulation of HSF2 by *Hsf2* siRNA in N2A cells lowered the intensity of the HSF1–HSF2 complex after EtOH treatment 5.7-fold in gel-shift assays (Fig 5A and B). However, this was not due to a decrease in HSF1 levels due to as-yet unknown feedback mechanisms (Fig 5C). HSF2 is thus necessary for HSF1 activation by alcohol.

We then verified whether HSF2 was also necessary for the formation of the atypical alcohol-induced HSF1–HSF2 heterotrimers in fetal cortices *in vivo*. In contrast to *Hsf2*^+/+^ cortices, no HSF1 HSE-binding activity could be detected in *Hsf2*^−/−^ cortices, when exposed to alcohol *in utero* (Fig 5D and E). This lack of binding was not due to diminished levels of HSF1 in EtOH-exposed fetal *Hsf2*^−/−^ cortices (Fig 5F), strongly suggesting that HSF2 was not only a component of the alcohol-induced heterotrimers, it was also necessary for the binding of HSF1 to HSE, at least in gel-shift assays.

This prominent role of HSF2, observed in fetal cortices and N2A cells, was not seen in *Hsf2*^−/−^ iMEFs, in which HSF1 activation could still occur (Supplementary Fig S10A), suggesting that this novel and essential role for HSF2 in alcohol-induced heterotrimer formation might be specific to the developing murine cortex and neural models.

### The presence of HSF2 worsens radial neuronal migration defects induced by fetal alcohol exposure

We next examined whether the presence of HSF2—and thereby the formation of HSF1–HSF2 heterotrimers—was beneficial or detrimental to the EtOH-induced defects in neuronal migration. To do this, we used BrdU to label neural progenitors in the cortex of *Hsf2*^*+/+*^ and *Hsf2*^−/−^ mouse fetuses exposed to CAI or not at E16.5 and analyzed the effects of alcohol exposure on neuronal positioning in the cortical plate at P0. It should be mentioned here that in the absence of alcohol exposure, HSF2 actually plays a positive regulatory role in neuronal migration in the fetal cortex. *Hsf2*^−/−^ animals show abnormalities in the final positioning of neurons (Chang *et al*, 2006). To avoid the confounding effect of these modifications to the “baseline” neuronal migration and positioning in *Hsf2*/fetal cortices, we chose a genetic background for our mice (C57Bl/6N × C57Bl/6J) in which such effects are not very pronounced. In addition, we normalized neuronal numbers to those in control animals of the same genotype so as to limit our analysis to the effects of alcohol.

**Figure 6:**
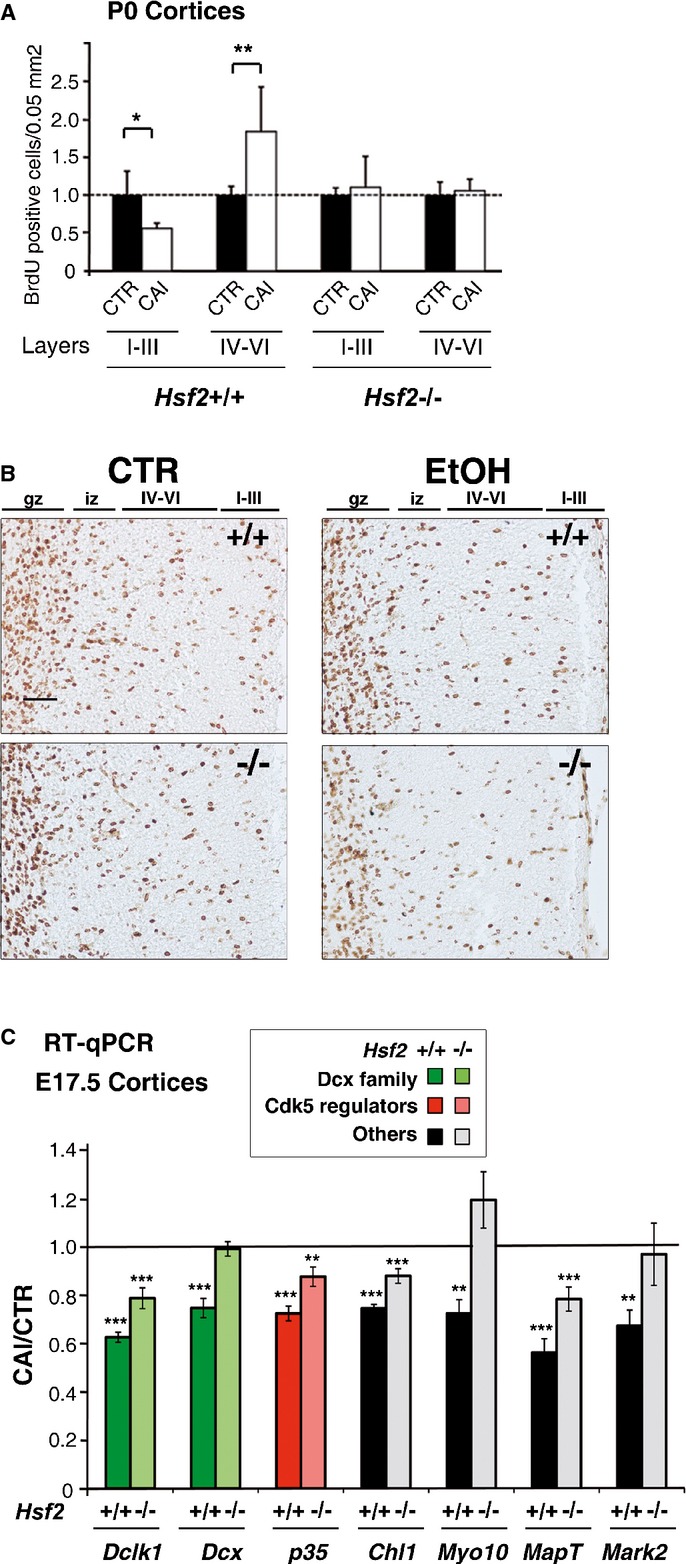
The absence of HSF2 reduces the abnormalities in neuronal positioning and disturbances in gene expression induced by fetal alcohol exposure A   Comparison of the severity of radial neuronal positioning defects between *Hsf2*^+/+^ and *Hsf2*^−/−^ cortices. BrdU-labeled cells (BrdU injection at E16.5 and neuronal positioning examined at P0) in fetal cortices from pregnant dams chronically intoxicated with food containing EtOH *ad libitum* (CAI) per 0.05 mm^2^. For each genotype and group of layers, the CTR situations were set to 1 (dotted line). Superficial layers, I–III; deep layers, IV–VI. (*n* = 5 sections from three different individuals per condition). The ratio is significantly different from 1 in *Hsf2*^+/+^ (*P*-values: *P = 0.0281 for layers I–III; **P = 0.0061 for layers IV–VI) The ratio was not significantly different from 1 in *Hsf2*^−^^/−^ (*P*-values: *P* = 0.5729 for layers I–III; *P* = 0.5627 for layers IV–VI). See also Supplementary Fig S11A for non-normalized data. Differences were considered statistically significant when *P*-values were ≤ 0.05, using unpaired two-way Student's *t*-test. Data are presented as mean ± SEM. B   Representative images corresponding to panels in (A). Gz, germinal zones; iz, intermediate zone; IV–VI and I–III, deep and superficial layers, respectively. Scale bar: 10 μM. C   Reduced disturbances of *MAP* gene expression in the absence of HSF2. Bars stand for ratios of values in CAI to those in CTR samples. Comparison of the severity of disturbances in *Dclk1*, *Dcx*, *p35*, *Chl1*, *Myo10*, *MapT*, and *Mark2* expression, in RT-qPCR experiments, between E17.5 *Hsf2*^+/+^ and *Hsf2*^−/−^ cortices subjected (CAI) or not (CTR) to fetal alcohol exposure (*n* = 4–7). A ratio of 1 is indicated by the black line. *P*-values (see Supplementary Table S1 for details): ***P < 0.0001; **P < 0.001. Same color code as in Figs 1C and D, and 2B. The graph represents the mean value of the ratio between each sample value and each control value. *P*-values were calculated by comparing ratios obtained for the control (CTR) condition versus the CAI one. For *Dclk1*, *Dclk2*, *Dcx*, *Chl1*, and *p35*, *n* = 5, individual cortices were analyzed for WT CTR; *n* = 7 for KO CTR, *n* = 6 for WT CAI; *n* = 4 for KO CAI. For *Myo10*, *MapT*, and *Mark2, n* = 3 for each conditions. Differences were considered statistically significant when *P*-values were < 0.05, using unpaired two-way Student's *t*-test. Data are presented as mean ± SEM. See also Supplementary Fig S11.

After normalizing neuronal densities to those in control animals to take into account neuronal positioning defects in the knockouts, it appeared that this loss of HSF2 protected the developing cortex from the incorrect neuronal positioning induced by EtOH (Fig 6A and B; for non-normalized data, see Supplementary Fig S11A). We then investigated the expression levels of HSF2 target genes after CAI in *Hsf2*^−/−^ and *Hsf2*^+/+^ cortices. We studied the expression of 14 genes from different families known from the literature to regulate radial neuronal migration (see Supplementary Information) and to bind HSF1 or HSF2 under control or HS conditions in unbiased ChIPSeq analyses (Mendillo *et al*, 2012; Vihervaara *et al*, 2013). As for *Dclk1*, *Dcx*, and *p35*, the expression of *Chl1* (Demyanenko *et al*, 2004), *Myo10* (Ju *et al*, 2014), *MapT* (Sapir *et al*, 2012), and *Mark2* (Sapir *et al*, 2008) was reduced upon fetal alcohol exposure in wild-type cortices (CAI/CTR ratio lowered by more than 25%; Fig 6C). In line with the BrdU birth-dating results, the EtOH-induced perturbations in the expression of genes such as *Dclk1, Dcx*, *Chl1, Myo10*, *MapT,* and *Mark2* in fetal cortices were also less severe in *Hsf2*^−/−^ animals (lighted bars) when compared with *Hsf2*^+/+^ animals (ratio CAI/CTR closer to 1; Fig 6C, see also Supplementary Fig S11B; Supplementary Table S1). Namely, the ratio CAI/CTR of mRNA levels in *Hsf2*^−/−^ fetal cortices was either significantly closer to 1 (*Dclk1*, *p35*, *Chl1*, *MapT*) or not significantly different from 1 (*Dcx*, *Myo10*, *Mark2*). The expression of eleven other genes involved in radial neuronal migration was not significantly affected by CAI. Interestingly, their expression was found not to be dependent on HSF2 (ratio CAI/CTR not notably different between *Hsf2*^+/+^ and *Hsf2*^−/−^ cortices (Supplementary Fig S11B).

**Figure 7:**
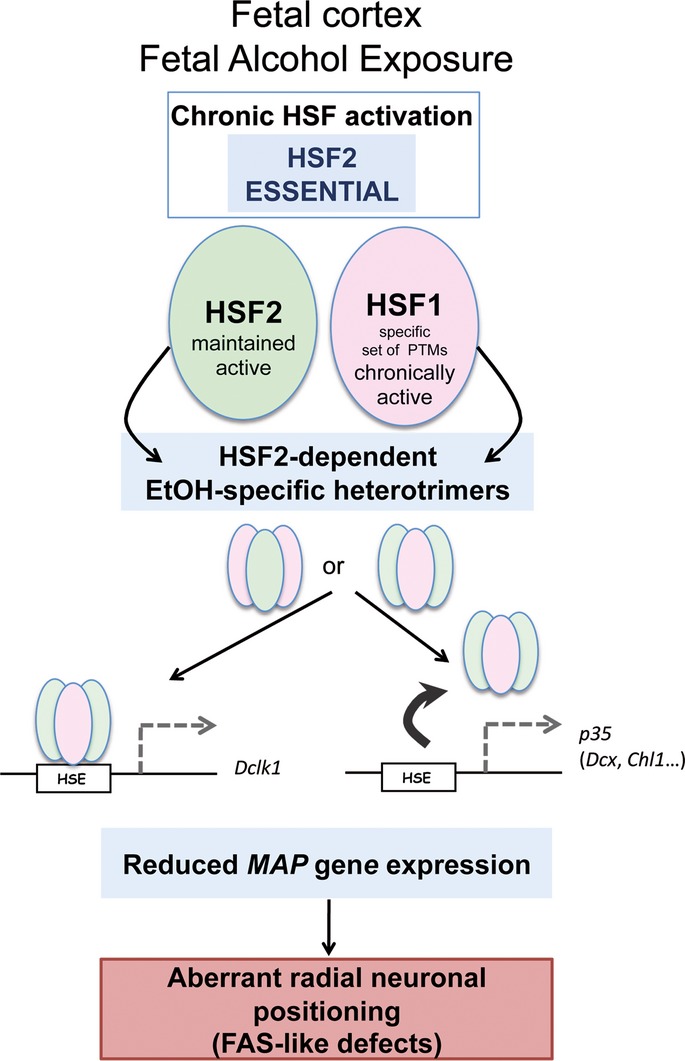
Model of the action of HSF2 as a mediator of radial neuronal migration defects upon fetal alcohol exposure Fetal alcohol exposure leads to the maintenance of HSF2 activity and to persistent HSF1 activation, through an alcohol-specific set of HSF1 post-translational modifications (lack of acetylation and hyperphosphorylation, and limited sumoylation). HSF2 is essential for the activation of HSF1 in the developing brain cortex (but not in iMEFs, for example). This leads to the formation of alcohol-specific heterotrimers (HSF1 and HSF2 monomers in pink and green, respectively) that bind to HSF2 target genes involved in neuronal migration (which are bound by HSF2 homotrimers under normal conditions) and disturb their expression. Alternatively, the formation of heterotrimers prevents binding to the HSE (arrow). In the absence of HSF2, these disturbances are less pronounced than in wild-type cortices, and defects in the positioning of neurons are less severe. Thus, through its ability to steer HSF1 activation in the developing cortex, HSF2 is a mediator of the neuronal migration defect characteristics of fetal alcohol exposure in FAS models.

Thus, HSF2 appears to play a detrimental role in the developing cortex chronically exposed to alcohol by participating in the formation of atypical heterotrimers that, by binding to genes involved in radial neuronal migration that are normally regulated by HSF2, result in their abnormal expression (Fig 7).

## Discussion

Two HSFs, HSF1 and HSF2, are expressed in the developing brain and possibly involved in the stress response in the brain. In model cell systems, the predominant role in transcriptional regulation has until now been attributed to HSF1, which indeed is absolutely essential for the response to classic HS, while HSF2 has been considered merely to fine-tune the actions of HSF1. During normal brain development, however, HSF2 is known to control the transcription of several genes involved in radial neuronal migration during corticogenesis, and mice genetically knocked out for HSF2 present neuronal positioning anomalies (Chang *et al*, 2006). Defective neuronal migration and positioning are also a hallmark of FASD, with far-reaching pathophysiological consequences. In the present study, we provide the first evidence that HSF2 plays a crucial role in the response of the fetal cortex to alcohol during cortical development and, furthermore, that HSF2 is essential for the activation of HSF1 following exposure to alcohol *in utero*. In summary, using alcohol exposure in fetal mouse models *in vivo* and neural cell systems *ex vivo*, we demonstrate that HSF2 drives the response of the fetal cortex to alcohol via a novel HSF1–HSF2 interaction based on the formation of alcohol-specific heterotrimers. Upon alcohol exposure, the sustained expression and binding of these HSF1–HSF2 heterotrimers—or conversely, the loss of their binding—to HSE-containing genes that regulate radial neuronal migration leads to the perturbation of their expression. In the absence of HSF2, these modifications in gene expression and the accompanying aberrant neuronal positioning are less severe, suggesting that HSF2 mediates some of the migration defects associated with FASD (Fig 7).

These findings are remarkable in several respects. Firstly, they reveal that HSF2 is not merely a modulator of the transcriptional activities of HSF1, as, for example, in the case of cell systems or preimplantation embryos subjected to classic HS (Östling *et al*, 2007; Le Masson *et al*, 2011), but an essential and, possibly, even the principal component of the response to fetal alcohol exposure in the brain. Secondly, HSF2, although not strictly necessary for the HSR, very transiently associates with HSF1 and fine-tunes the HSF1-dependent induction of heat shock gene transcription, before being strikingly and rapidly inactivated by HS itself (He *et al*, 2003; Östling *et al*, 2007; Sandqvist *et al*, 2009; Ahlskog *et al*, 2010). HSF1 is also inactivated following its post-translational modification, but after longer exposure to HS (reviewed in Åkerfelt *et al*, 2010). In the response to chronic alcohol exposure, however, both HSF1—which displays an unusual set of post-translational modifications—and HSF2 are moderately but persistently activated in the developing brain, with potentially drastic consequences in terms of gene transcription and its structural and functional repercussions. Furthermore, in the absence of HSF2, HSF1 cannot be activated by alcohol exposure. Thirdly, the effects of alcohol on HSF activity are mediated by yet another unusual mechanism—the formation of specific HSF1–HSF2 heterotrimers whose biochemical properties are distinct from those previously characterized for other types of stress (Sarge *et al*, 1993; Sistonen *et al*, 1994; Loison *et al*, 2006; Östling *et al*, 2007; Sandqvist *et al*, 2009). While there has been much speculation regarding the existence of HSF1–HSF2 heterotrimers *in vivo*, or at least under pathophysiological conditions, this has been remarkably difficult to demonstrate, with a couple of notable exceptions: the formation of functionally active heterotrimers following proteasome inhibition (including treatment with hemin) or with the amino acid analog AZC (L-azetidine-2-carboxylic acid) (Loison *et al*, 2006; Östling *et al*, 2007). However, these complexes are not distinguishable from those induced following HS, which is clearly not the case with our alcohol-induced heterotrimers with their distinctive behavior in gel-shift assays and glycerol gradients. This is therefore the first concrete evidence for the *in vivo* formation of HSF1–HSF2 heterotrimers, which, moreover, display distinctive alcohol-specific features. Lastly, our findings provide, for the first time, a molecular explanation for the cortical neuronal positioning defects typical of FAS, based on the differential binding and transcriptional activation of genes involved in radial neuronal migration in the developing brain by these atypical but persistent HSF1–HSF2 heterotrimers. The fact that the enigmatic HSF2, whose role in the stress response *in vivo* has remained elusive for so many years, occupies such a prominent place, both under adverse environmental conditions and during the normal development of the organism, is of great significance and is in line with recent findings regarding its prominent role in mitotic cells (Vihervaara *et al*, 2013).

How alcohol induces HSF activation is still unknown. As an amphiphilic (that is, both lipophilic and hydrophilic) molecule, alcohol could trigger HSF1 activation in a pleiotropic manner. In particular, it is known to induce oxidative stress (Henderson *et al*, 1995; Brocardo *et al*, 2011; Ikonomidou & Kaindl, 2011), and the redox-dependent activation of HSF1 has been reported previously (Ahn & Thiele, 2003). Regardless of how the mechanism is initiated, the effects of alcohol appear to be mediated by the modest but prolonged activation of the HSFs through the formation of stable alcohol-specific heterotrimers. The result of this stability on the long-term DNA-binding and transcriptional activity of HSF1–HSF2 heterotrimers is decisive for their potential role in FASD, since it allows HSF1 and HSF2 to continue exerting their alcohol-induced effects in the fetal cortex over long periods of time rather than being inactivated rapidly as with HS.

This long-term activity of HSF1–HSF2 heterotrimers depends in fact on the persistent activation of their two components. Firstly, the DNA-binding activity of HSF2 is resistant to chronic alcohol stress, whereas HSF2 is quickly inactivated and degraded upon classic HS, as mentioned above (Sarge *et al*, 1993; Sistonen *et al*, 1994; Mathew *et al*, 2001; Ahlskog *et al*, 2010). HSF2 is also activated by mild (febrile range) HS, but HSF1 is not activated under these conditions, precluding the formation of heterotrimers (Shinkawa *et al*, 2011). Secondly, HSF1 can be chronically activated *in vivo* or *ex vivo* by prolonged EtOH exposure, although it is only transiently activated by prolonged classic HS. This atypical lack of attenuation of HSF1 DNA-binding and transcriptional activity upon EtOH exposure could be related to the alcohol-specific set of post-translational modifications that HSF1 undergoes. For example, while acetylation accompanies HSF1 activation, it also seems to be necessary for the attenuation of its DNA-binding activity upon HS (Westerheide *et al*, 2009). The sumoylation and hyperphosphorylation of HSF1 are also signs of HSF1 activation in terms of transcriptional ability, but not requirements. Contrarily, HSF1 hyperphosphorylation appears to be linked to the repression of its transcriptional ability (Newton *et al*, 1996; Kline & Morimoto, 1997; Kim & Li, 1999; Hietakangas *et al*, 2003), and the HS-induced sumoylation of HSF1 is known to provide a buffering capacity for its transcriptional abilities, adapted to the severity of the HS (Hietakangas *et al*, 2003). Therefore, the reduction of HSF1 acetylation, the absence of its hyperphosphorylation, and its limited and delayed sumoylation could all contribute to its persistent DNA-binding and transcriptional activity upon chronic alcohol exposure.

While the HSF1–HSF2 heterocomplexes induced by alcohol are biochemically different from complexes induced by classic HS or certain other types of stress mentioned above (Loison *et al*, 2006; Östling *et al*, 2008), sodium salicylate, like EtOH, induces HSF1–HSE complexes with slower migration properties during electrophoresis than HS, but, unlike EtOH, triggers HSF1 hyperphosphorylation to some extent (Jurivich *et al*, 1992, 1995). In addition, only HSF1, and not HSF2, displays DNA-binding activity upon sodium salicylate treatment, and this treatment is not capable of triggering *Hsp* gene transcription. This suggests that the characteristic behavior of EtOH-specific HSF1–HSF2 complexes in gel-shift assays and glycerol gradients might be due to the distinctive post-translational modification profile of HSF1, rather than to the presence of HSF2. The differences observed in the acetylation or sumoylation of HSF1 and/or its lack of hyperphosphorylation after alcohol exposure, when compared to other stresses such as HS, could induce conformational peculiarities that might account for this unusual behavior of HSF1–HSF2 complexes.

In addition to the striking abundance and elevated DNA-binding activity of HSF2 in the developing cortex, the HSF heterotrimer-mediated effects of alcohol may also depend upon still unidentified post-translational modifications in HSF2, which might control its stability, its participation in atypical heterotrimer formation (and the peculiar characteristics of these heterotrimers), and consequently its activity in the normal fetal cortex and/or after alcohol exposure. Post-translational modifications in HSF2 might also contribute to the recruitment of partners or complexes that negatively modulate the transcription of target genes upon alcohol exposure, especially in the fetal cortex (see below).

The relative concentrations of HSF1 and HSF2 within the developing cortex (HSF2 > HSF1) might also be critical for its response to alcohol. Surprisingly, in *Hsf2*^−/−^ embryos or upon the *ex vivo* knockdown of HSF2 in neural cell lines, HSF1 activity could not be induced by alcohol exposure, suggesting that HSF2, far from being a “silent partner,” is in fact the driving force behind the changes in the fetal cortex exposed to alcohol. This is in contrast to the situation in *Hsf2*^−/−^ iMEFs (Supplementary Fig S10A), which could be explained by the fact that, unlike fetal cortices and N2A cells, these non-neural cells do not display constitutively high HSF2 DNA-binding activity. The strong expression and constitutive activity of HSF2 during normal brain development (See Supplementary Fig S10D; Rallu *et al*, 1997; Kallio *et al*, 2002; Chang *et al*, 2006) might not only be important for normal brain development but also confer on the fetal cortex a unique vulnerability to *in utero* alcohol exposure, favoring the formation of alcohol-specific HSF1–HSF2 heterotrimers and triggering the prolonged, albeit low-grade, activation of HSF1. However, whether these HSF1–HSF2 interactions prevent HSF1 from becoming heavily post-translationally modified, or conversely, whether the lack of major post-translational modifications favors the stable interaction of HSF1 with HSF2, remains to be elucidated. Nevertheless, any modification in their concentration ratio might compromise or modify the response of HSF1 and HSF2 to alcohol in the fetal cortex. For instance, it is tempting to hypothesize that the variability in HSF1 and HSF2 levels among individuals and consequently on their ratio could constitute a molecular basis for the enigmatic variability in sensitivity to FAS that is observed in the human population (reviewed in Gressens *et al*, 2001).

In terms of their putative function as transcriptional modulators through HSE binding, the alcohol-induced interaction of HSF1 and HSF2 contributes to the downregulation of the expression of various genes involved in radial neuronal migration during different stages of corticogenesis, such as the *MAPs* (e.g. *Dcx*, *Dclk1* and *2,* and *Lis1*), as well as other genes upstream or downstream of them (*p35*, *p39*, *Chl1*, *Mark2,* etc.). We observed two situations: (i) the loss of binding of both HSF1 and HSF2 to the HSE upon alcohol exposure, as in the case of *p35, Dcx*, and *Chl1*, which might contribute to the downregulation of the gene in a manner resembling that described in *Hsf2*^−/−^ cortices (Chang *et al*, 2006): In this case, the formation of heterotrimers seems to sequester HSF2, leading to the loss of target gene binding and the downregulation of their expression; (ii) the binding of an HSF1–HSF2 heterotrimer to the HSE, as in the case of *Dclk1,* leading to altered gene expression. What role, if any, is played by the similarity of the HSE of a given gene to the consensus sequence or its position within the gene is not yet clear. However, *Dcx* and *Chl1* possess HSEs that closely match the canonical HSE sequence, whereas *p35* does not, suggesting that the HSE sequence, by itself, is not sufficient to dictate HSF binding and that the chromatin environment might be an important modulator. For *p35*, *Dcx*, and *Chl1*, it appears that interfering with normal HSF2 binding to an HSE situated in the vicinity of the promoter region leads to the downregulation of gene expression. The fact that the binding of alcohol-induced heterotrimers to an HSE within the body of the gene, as in the case of *Dclk1*, also results in the downregulation of the target gene is not surprising. Indeed, HSF1 is a potent activator of *Hsp* gene expression upon HS, but negatively regulates other genes in other situations, especially those in which, such as *Dclk1*, HSEs are not located in the promoter region (Mendillo *et al*, 2012). The amazing transcriptional modification capabilities of HSF1 have also recently been demonstrated by its complete rewiring of the transcriptome in cancer (Mendillo *et al*, 2012). Similarly, HSF2 can negatively or positively regulate the transcription of its target genes depending on the context. For example, it positively influences the expression of *Hsp70 and Hsp25* but negatively influences that of *Hsp40* and *Hsp110* in response to HS, and moreover, it negatively regulates *MLL* during mitosis (Östling *et al*, 2007; Vihervaara *et al*, 2013). Alcohol-induced alterations of the HSFs, including post-translational modifications and the formation of alcohol-specific heterotrimers, could therefore explain a wide variety of changes in the transcription of *Dclk1*, *Dcx, p35,* and other genes in the developing cortex.

The effects of the HSFs on gene transcription following their alcohol-induced modification need not be direct. Indeed, the role of HSF2 in the control of gene expression has also recently been elucidated by its ability to profoundly remodel the chromatin landscape (Vihervaara *et al*, 2013). The alcohol-mediated loss of HSF2 binding in the case of *p35*, *Dcx*, and *Chl1*, possibly due to changes in the binding specificity of the trimers, and conversely the binding of alcohol-specific heterotrimers to other genes such as *Dclk1*, might result in major changes in the chromatin environment in terms of the recruitment of activator or repressor complexes or histone modifications. The combination of HSF2 and HSF1 might also perturb gene expression by modifying the epigenetic landscape, since HSF1 is also known to recruit chromatin remodelers and other transcriptional activators, as well as to target histone modifications in a stress-specific manner (Sullivan *et al*, 2001; Thomson *et al*, 2004; Inouye *et al*, 2007; Fujimoto *et al*, 2008, 2012; Gómez *et al*, 2008; Khaleque *et al*, 2008; Fritah *et al*, 2009).

Strikingly, however, the overall DNA-binding activity and transcriptional ability of HSF1–HSF2 heterotrimers appeared to be only modestly induced by alcohol when compared to HS. This low-grade activation of the HSFs could be crucial for their potential role in FASD, since the overexpression of HSF1 or the HSPs is known to be toxic to the cell (Feder *et al*, 1992; Krebs & Feder, 1997; Bruce *et al*, 1999). In addition, the low levels of HSP70 and HSP90 induced under conditions of chronic alcohol exposure could limit the negative feedback loop that the HSPs exert on HSF1 (Boyault *et al*, 2007; Åkerfelt *et al*, 2010). Thus, the fact that alcohol induces only a moderate increase in HSF1/HSF2 activity (and in *Hsp* levels) is likely another factor that contributes to the prolonged activation and limited toxicity of these heterotrimers in the fetal brain and favors the pathological role of these factors in FASD.

These modest but significant alcohol-induced disturbances in the expression of at least seven genes involved in neuronal migration, such as some *MAPs* and their activator and effector molecules, are likely to alter radial neuronal migration in a cumulative way. Indeed, DCX and DCLK1 display overlapping and compensatory effects on radial neuronal migration (reviewed in Ayala *et al*, 2007). The concomitant HSF2-dependent downregulation of multiple *MAP* genes within fetal cortices exposed to alcohol could therefore block this compensatory mechanism and lead to migration defects. Likewise, alterations in phosphorylation/dephosphorylation cascades, such as the reduction in the expression of *p35*, the activator of Cdk5, a kinase needed for the phosphorylation of DCX and its association with microtubules (Tanaka *et al*, 2004), could have far-reaching consequences. However, these defects, both in terms of cortical development and in terms of the expression of the most-affected genes, are notably reduced in *Hsf2*^−/−^ cortices, in keeping with the lack of HSF1 activation in the absence of HSF2, suggesting that the perturbations in HSF2 activity in the fetal cortex—via the formation of anomalous HSF1–HSF2 heterotrimers—are detrimental for *MAP* gene expression and neuronal migration following alcohol exposure.

Interestingly, in contrast to genes that are markedly affected by alcohol exposure, the expression of genes involved in radial neuronal migration that are not (or only poorly) affected by fetal alcohol exposure is not influenced by the absence of HSF2. This further reinforces the notion that HSF2 is a major mediator of disturbances in the expression of genes affected by alcohol, and therefore of the radial neuronal migration defects characteristic of FAS, and that the involvement of HSF2 as a fine-tuner of the expression of these genes, which is beneficial under normal conditions, might constitute a “cost” when the fetal brain is exposed to alcohol. This might not be the case in other organs or cell systems such as the iMEFs, in which HSF2 could be protective (Supplementary Fig S10B and C). Similarly, this detrimental effect may be absent in the case of other stresses of pathological importance for the prenatal and perinatal period: For example, HSF2 might protect the developing cortex against fever, since HSF1 is neither induced nor needed in the febrile temperature range (Shinkawa *et al*, 2011).

In conclusion, our work has succeeded in elucidating some of the molecular and genetic alterations by which chronic fetal alcohol exposure leads to brain structural abnormalities characteristic of FASD, via the activation of HSF1 and HSF2. The search for compounds capable of modulating the actions of HSF1 is already a focus for therapeutic strategies for cancer and neurodegenerative diseases (Westerheide & Morimoto, 2005; Whitesell & Lindquist, 2009; Neef *et al*, 2010, 2011), and it is tempting to consider our findings of the essential role of HSF2 in the fetal cortex as a basis for strategies to prevent or limit brain damage in the case of FAS. Future challenges will involve building on this new fundamental insight both into the role of HSF1 and HSF2 in fetal cortical development under normal and alcohol-exposed conditions, and on the different mechanisms by which they could interact and regulate the transcription of their target genes under conditions of environmental stress, to implement clinically relevant solutions.

## Materials and Methods

### Mice, heat shock, and EtOH exposure

*Hsf2* heterozygous mice described in Kallio *et al* (2002) were obtained in a mixed C57Bl/6 N × C57Bl/6 J background (Fig 6). The noon on the day of the vaginal plug was considered as embryonic day 0.5 (E0.5). For chronic EtOH stress on wild-type animals (in Fig 1), pregnant 2- to 4-month-old C57Bl6/J female mice were exposed to EtOH between E7.5 and E18.5 following three different modes of EtOH administration. The first group of animals was fed *ad libitum* with a semi-liquid diet (WUARA00D44, Safe, France; Gressens *et al*, 1992) containing 70 mg/ml (2%) EtOH. Controls were fed *ad libitum* with a semi-liquid diet without EtOH and providing the same amount of calories per volume. The second group of animals received one daily force-feeding (gavage) of EtOH (3 mg/kg/gavage) diluted in a final volume of 200 μl of PBS. Controls received PBS alone. The last group of animals received two daily intraperitoneal injections of EtOH (1.5 mg/kg/injection) diluted in a final volume of 200 μl of PBS. Controls received PBS alone. For acute EtOH stress, pregnant dams were submitted to a single intraperitoneal injection of EtOH at doses known to induce brain defects in rodent fetuses that mimick FAS (3 or 6 g/kg initial solution: 40% EtOH in PBS Ikonomidou *et al*, 2000; Olney *et al*, 2002; Carloni *et al*, 2004; Ieraci *et al*, 2006, 2007). Dams were sacrificed 2 or 4 h later as indicated. Mother alcoholemia was determined using the EnzymChrom Ethanol Assay kit (ECET-100, BioAssay Systems; CAI: 3.9–8 g/l; IPI: 4.5 g/l) or by the facilities in Hospital Bichat, Paris, France (CAI: 1–3 g/l). We verified that in our hands, BrdU incorporation at E16.5 was similarly affected by the three protocols of chronic fetal alcohol exposure (Supplementary Fig S1A). Typically, 10 pregnant females were used for each condition. Experimental protocols were approved by the institutional review committee (Robert Debré and Bichat-University hospitals committee), meet the Inserm guidelines, and were carried out in accordance with the Guide for the Care and Use of Laboratory Animals as adopted and promulgated by the U.S. National Institutes of Health. All efforts were made to reduce stress and pain to animals. *Hsf2*^*tm1Mmr*^ mice and the corresponding experimental protocols have received the agreement # 5314 from Ministère de l'Enseignement Supérieur et de la Recherche were approved by the Institutional Animal Care and Use Ethical Committee of the Paris-Diderot University (registration number CEEA-40).

### Immunohistochemistry

Brains were fixed in 4% formalin 24 to 48 h prior to paraffin embedding. Sixteen micron-thick coronal sections were cut at the level of S1. For BrdU detection, deparaffinized sections were microwaved in citrate buffer and incubated in HCl prior to incubation with primary antibodies. To investigate proliferation and migration, pregnant mice were injected intraperitoneally with 50 mg/kg BrdU (diluted in 100 μl of PBS) at E16.5 and sacrificed either 2 h after BrdU injection or at P0, respectively. BrdU-positive cells were counted on 6 whole VZ and SVZ cortices from three independent litters.

### Electrophoretic mobility shift assay

Cell extracts were prepared as described (Mezger *et al*, 1989). Pools of three embryos of the same litter were submitted to 2–4 rapid freeze–thaw cycles, in five volumes of extraction buffer. Whole cell extracts (30 μg of proteins) were incubated with a (^32^P)-labeled HSE oligonucleotide (5′-CTAGAACGTTCTAGAAGCTTCGAGA-3′), and complexes were separated on a native 4% polyacrylamide gel as described (Rallu *et al*, 1997). The components of the retarded complexes were analyzed by supershift using antibodies against HSF1 or HSF2 (10 ng/μl final; 3E2 and Ab4 neomarkers). The intensities of HSF–HSE complexes were quantified by PhosphoImager FLA3000 or 7000 (Fuji).

### Glycerol density gradients

Three hundred micrograms of total native extracts were loaded on the top of a 5 ml glycerol gradient composed (from top to bottom) of 5, 15, 25, 35 and 45% (1 ml each) and ultracentrifuged (Beckman, rotor MLS50, 226,800 g) for 16 h. The gradient was aliquoted in 200 μl fractions (named 5–1 to 5–5; 15–1 to 15–5, etc. up to 45–5; Fig 4C and D), and the content in HSF1 and HSF2 of 30 μl of the fractions was analyzed by WB on SDS–PAGE. (input, 40 μg of total extract).

### Quantitative RT-PCR for quantification of mRNA levels

The caudo-medial region of the cortical plate (the region which is mostly affected in term of neuronal migration in *Hsf2*^−/−^ fetuses) of E16.5 cortices from embryos of the same litter was dissected and pooled into RNAlater (Ambion). RNA was purified using Illustra RNAspin Mini RNA Isolation kit (GE Healthcare). Reverse transcription was performed from 1 μg RNA using the Superscript II reverse transcriptase (Invitrogen Life Technologies). Quantitative PCR was performed in 96 well plates on the Light Cycler LC480 (Roche) using the Quantitect SYBER green PCR kit (Qiagen). For one given experiment, 3 genes, one highly, one moderately, and one weakly expressed, (cyclophilin B, protoporphyrinogen oxidase (Ppox), α_2_-tubulin, or Gapdh), were used for normalization of the results (Dauphinot *et al*, 2005; Chang *et al*, 2006). For primers sequences, see Supplementary Information.

### Chromatin immunoprecipitation (ChIP)

Putative HSE were identified by Genomatix software in the *Dclk1* and *Nde1* genes.

The ChIP protocol was modified from Takahashi *et al* (2000). E16.5 cortical tissue (corresponding to seven embryos) was cross-linked at 4°C, with a final concentration of 1% formaldehyde. Quenching was performed with a final concentration of 125 mM glycine at 4°C. After cortical cell dissociation, seven embryonic CPs were lysed in 2 ml of lysis buffer. Fragmentation of the chromatin samples was performed by sonication with Bioruptor (Diagenode) to an approximate size of 400 bp (20 min; 30 s on/30 s. off). IP was performed after preclearing with a 50% slurry of protein G-coated agarose beads containing bovine serum albumin (100 μg/ml, Amersham Biosciences) 3 h at 4°C. The following antibodies were used: HSF2 (Abcam ab44824) and HSF1 (Abcam ab81279). Washing of immunocomplexes was performed three times with wash buffer 1, twice with wash buffer 2, and three times in wash buffer 3 (for washing buffers, see Chang *et al*, 2006). Cross-links were reversed by incubating the samples overnight at 65°C. DNA was purified, and PCR analysis was performed on 1:10 of each ChIP sample using qPCR Mix SYBR Green (Amersham Biosciences). For primer sequences, see Supplementary Information.

### Statistical analysis

The statistical analyses for RT-qPCR analyses and ChIP experiments were conducted with two-tailed independent (unpaired) Student's *t*-test, assuming Gaussian distribution. The statistical analyses for BrdU labeling experiments were carried out using unpaired two-tailed Student's *t*-test (Figs 1A and 6A) or unpaired ANOVA, as indicated in the Legends, assuming Gaussian distribution.

**The paper explained**

**Problem**

Fetal alcohol spectrum disorders (FASD) are provoked by maternal consumption of alcohol during pregnancy and represent the most frequent cause of non-genetic mental retardation. The effects of prenatal exposure to alcohol, which can occur at any time during gestation, include the disruption of many aspects of fetal brain development. In particular, postmortem brains of infants with FAS exhibit disturbances in the positioning of neurons in the various cortical layers, and fetal alcohol exposure is known to affect radial neuronal migration, which governs horizontal cortical lamination. However, the molecular mechanisms responsible for these defects are still elusive.

**Results**

Here, we show that HSF2, a member of the heat shock factor (HSF) family of stress-responsive transcription factors, plays a pivotal role in the brain's response to fetal alcohol exposure by steering the long-term activation of HSF1—the major stress-responsive HSF—and the formation of alcohol-specific HSF1/HSF2 heterotrimers, which bind to and alter the expression of genes that control radial neuronal migration (for example, *p35* and microtubule-associated proteins such as *Dcx* and *Dclk1*). The persistent activation of HSF1 involves an alcohol-specific set of post-translational modifications. In the absence of HSF2, disturbances in gene expression and neuronal positioning defects are both less severe than in the presence of HSF2, strongly suggesting that HSF2 and the formation of heterotrimers mediate the neuronal migration defects characteristic of FASD.

**Impact**

Our studies demonstrate that HSF2 plays an important role in mediating cortical neuronal positioning defects in response to prenatal alcohol exposure. Our work has important clinical implications for the prevention or treatment of FAS. Additionally, the search for compounds that could modulate the activity of HSF1 has been at the center of current therapeutic strategies for cancer and neurodegenerative diseases. Our studies indicate that large high-throughput screening strategies should also target HSF2.

## Supporting Information

for this article is available online: http://embomolmed.embopress.org

## References

[b1] Abane R, Mezger V (2010). Novel aspects of heat shock factors: roles in gametogenesis and development. FEBS J.

[b2] Abravaya K, Phillips B, Morimoto RI (1991). Attenuation of the heat shock response in HeLa cells is mediated by the release of bound heat shock transcription factor and is modulated by changes in growth and in heat shock temperatures. Genes Dev.

[b3] Ahlskog JK, Björk JK, Elsing AN, Aspelin C, Kallio M, Roos-Mattjus P, Sistonen L (2010). Anaphase-promoting complex/cyclosome participates in the acute response to protein-damaging stress. Mol Cell Biol.

[b4] Ahn S-G, Thiele DJ (2003). Redox regulation of mammalian heat shock factor 1 is essential for Hsp gene activation and protection from stress. Genes Dev.

[b5] Åkerfelt M, Morimoto RI, Sistonen L (2010). Heat shock factors: integrators of cell stress, development and lifespan. Nat Rev Mol Cell.

[b6] Alkuraya FS, Cai X, Emery C, Mochida GH, Al-Dosari MS, Felie JM, Hill RS, Barry BJ, Partlow JN, Gascon GG (2011). Human mutations in NDE1 cause extreme microcephaly with lissencephaly. Am J Hum Genet.

[b7] Ayala R, Shu T, Tsai LH (2007). Trekking across the brain: the journey of neuronal migration. Cell.

[b10] Boyault C, Zhang Y, Fritah S, Caron C, Gilquin B, Kwon SH, Garrido C, Yao TP, Vourc'h C, Matthias P (2007). HDAC6 controls major cell response pathways to cytotoxic accumulation of protein aggregates. Genes Dev.

[b11] Brocardo PS, Gil-Mohapel J, Christie BR (2011). The role of oxidative stress in fetal alcohol spectrum disorders. Brain Res Rev.

[b12] Bruce JL, Chen C, Xie Y, Zhong R, Wang YQ, Stevenson MA, Calderwood SK (1999). Activation of heat shock transcription factor 1 to a DNA binding form during the G(1)phase of the cell cycle. Cell Stress Chaperones.

[b13] Carloni S, Mazzoni E, Balduini W (2004). Caspase-3 and calpain activities after acute and repeated ethanol administration during the rat brain growth spurt. J Neurochem.

[b14] Chang Y, Östling P, Åkerfelt M, Trouillet D, Rallu M, Gitton Y, El Fatimy R, Fardeau V, Le Crom S, Morange M (2006). Role of heat-shock factor 2 in cerebral cortex formation and as a regulator of p35 expression. Genes Dev.

[b15] Clarke ME, Gibbard WB (2003). Overview of fetal alcohol spectrum disorders for mental health professionals. Can Child Adolesc Psychiatr Rev.

[b16] Dauphinot L, Ryle R, Rivals I, Tran Dang M, Moldrich RX, Golfier G, Ettwiller L, Toyama K, Rossier J, Personnaz L (2005). The cerebellar transcriptome during postnatal development of the Ts1Cje mouse, a segmental trisomy model for down syndrome. Hum Mol Genet.

[b17] Demyanenko GP, Schachner M, Anton E, Schmid R, Feng G, Sanes J, Maness PF (2004). Close homolog of L1 modulates area-specific neuronal positioning and dendrite orientation in the cerebral cortex. Neuron.

[b18] Feder JH, Rossi JM, Solomon J, Solomon N, Lindquist S (1992). The consequences of expressing hsp70 in Drosophila cells at normal temperatures. Genes Dev.

[b19] Francis F, Meyer G, Fallet-Bianco C, Moreno S, Kappeler C, Socorro AC, Tuy FP, Beldjord C, Chelly J (2006). Human disorders of cortical development: from past to present. Eur J Neurosci.

[b20] Fritah S, Col E, Boyault C, Govin J, Sadoul K, Chiocca S, Christians E, Khochbin S, Jolly C, Vourc'h C (2009). Heat-shock factor 1 controls genome-wide acetylation in heat-shocked cells. Mol Biol Cell.

[b21] Fujimoto M, Oshima K, Shinkawa T, Wang BB, Inouye S, Hayashida N, Takii R, Nakai A (2008). Analysis of HSF4 binding regions reveals its necessity for gene regulation during development and heat shock response in mouse lenses. J Biol Chem.

[b22] Fujimoto M, Takaki E, Takii R, Tan K, Prakasam R, Hayashida N, Iemura S, Natsume T, Nakai A (2012). RPA assists HSF1 access to nucleosomal DNA by recruiting histone chaperone FACT. Mol Cell.

[b24] Gibbard WB, Wass P, Clarke ME (2003). The neuropsychological implications of prenatal alcohol exposure. Can Child Adolesc Psychiatr Rev.

[b25] Gómez AV, Galleguillos D, Maass JC, Battaglioli E, Kukuljan M, Andrés ME (2008). CoREST represses the heat shock response mediated by HSF1. Mol Cell.

[b26] Gressens P, Lammens M, Picard JJ, Evrard P (1992). Ethanol-induced disturbances of gliogenesis and neurogenesis in the developing murine brain: an in vitro and in vivo immunohistochemical and ultrastructural study. Alcohol Alcohol.

[b27] Gressens P, Mesples B, Sahir N, Marret S, Sola A (2001). Environmental factors and disturbances of brain development. Semin Neonatol.

[b28] Guettouche T, Boellmann F, Lane WS, Voellmy R (2005). Analysis of phosphorylation of human heat shock factor 1 in cells experiencing a stress. BMC Biochem.

[b29] Hashimoto-Torii K, Kawasawa YI, Kuhn A, Rakic P (2011). Combined transcriptome analysis of fetal human and mouse cerebral cortex exposed to alcohol. Proc Natl Acad Sci USA.

[b30] Hashimoto-Torii K, Torii M, Fujimoto M, Nakai A, El Fatimy R, Mezger VJu MJ, Ishii S, Chao S, Brennand KJ, Gage FH (2014). Roles of Heat Shock Factor 1 in neuronal response to fetal environmental risks and its relevance to brain disorders. Neuron.

[b31] He H, Soncin F, Grammatikakis N, Li Y, Siganou A, Gong J, Brown SA, Kingston RE, Calderwood SK (2003). Elevated expression of heat shock factor (HSF) 2A stimulates HSF1-induced transcription during stress. J Biol Chem.

[b32] Henderson GI, Devi BG, Perez A, Schenker S (1995). In utero ethanol exposure elicits oxidative stress in the rat fetus. Alcohol Clin Exp Res.

[b33] Hietakangas V, Ahlskog JK, Jakobsson AM, Hellesuo M, Sahlberg NM, Holmberg CI, Mikhailov A, Palvimo JJ, Pirkkala L, Sistonen L (2003). Phosphorylation of serine 303 is a prerequisite for the stress-inducible SUMO modification of heat shock factor 1. Mol Cell Biol.

[b34] Ieraci A, Daniel G, Herrera DG (2006). Nicotinamide protects against ethanol-induced apoptotic neurodegeneration in the developing mouse brain. PLoS Med.

[b35] Ieraci A, Daniel G, Herrera DG (2007). Single alcohol exposure in early life damages hippocampal stem/progenitor cells and reduces adult neurogenesis. Neurobiol Dis.

[b36] Ikonomidou C, Bittigau P, Ishimaru MJ, Wozniak DF, Koch C, Genz K, Price MT, Stefovska V, Hörster F, Tenkova T (2000). Ethanol-induced apoptotic neurodegeneration and fetal alcohol syndrome. Science.

[b37] Ikonomidou C, Kaindl AM (2011). Neuronal death and oxidative stress in the developing brain. Antioxid Redox Signal.

[b38] Inouye S, Fujimoto M, Nakamura T, Takaki E, Hayashida N, Hai T, Nakai A (2007). Heat shock transcription factor 1 opens chromatin structure of interleukin-6 promoter to facilitate binding of an activator or a repressor. J Biol Chem.

[b39] Jolly C, Usson Y, Morimoto RI (1999). Rapid and reversible relocalization of heat shock factor 1 within seconds to nuclear stress granules. Proc Natl Acad Sci USA.

[b40] Jones KL, Smith DW (1973). Recognition of the fetal alcohol syndrome in early infancy. Lancet.

[b41] Ju XD, Guo Y, Wang NN, Huang Y, Lai MM, Zhai YH, Guo YG, Zhang JH, Cao RJ, Yu HL, Cui L, Li YT, Wang XZ, Ding YQ, Zhu XJ (2014). Both Myosin-10 isoforms are required for radial neuronal migration in the developing cerebral cortex. Cereb Cortex.

[b42] Jurivich D, Sistonen L, Kroes RA, Morimoto RI (1992). Effect of sodium salicylate on the human heat shock response. Science.

[b43] Jurivich DA, Pachetti C, Qiu L, Welk JF (1995). Salicylate triggers heat shock factor differently than heat. J Biol Chem.

[b44] Kallio M, Chang Y, Manuel M, Alastalo T-P, Rallu M, Gitton Y, Pirkkala L, Loones M-T, Paslaru L, Larney S (2002). Brain abnormalities, defective meiotic chromosome synapsis and female subfertility in HSF2 null mice. EMBO J.

[b45] Khaleque MA, Bharti A, Gong J, Gray PJ, Sachdev V, Ciocca DR, Stati A, Fanelli M, Calderwood SK (2008). Heat shock factor 1 represses estrogen-dependent transcription through association with MTA1. Oncogene.

[b46] Kim D, Li GC (1999). Proteasome inhibitors lactacystin and MG132 inhibit the dephosphorylation of HSF1 after heat shock and suppress thermal induction of heat shock gene expression. Biochem Biophys Res Com.

[b47] Kline MP, Morimoto RI (1997). Repression of the heat shock factor 1 transcriptional activation domain is modulated by constitutive phosphorylation. Mol Cell Biol.

[b48] Krebs RA, Feder ME (1997). Deleterious consequences of Hsp70 overexpression in *Drosophila melanogaster* larvae. Cell Stress Chaperones.

[b49] Lemoine P, Harousseau H, Borteyru J (1968). Les enfants de parents alcooliques: anomalies observées. Ouest Méd.

[b501] Le Masson F, Christians E (2011). HSFs and regulation of Hsp70.1 (Hspa1b) in oocytes and preimplantation embryos: new insights brought by transgenic and knockout mouse models. Cell Stress Chaperones.

[b52] Loison F, Debure L, Nizard P, le Goff P, Michel D, Le Dréan Y (2006). Up-regulation of the clusterin gene after proteotoxic stress: implication of HSF1-HSF2 heterocomplexes. Biochem J.

[b53] Mathew A, Mathur SK, Jolly C, Fox SG, Kim S, Morimoto RI (2001). Stress-specific activation and repression of heat shock factors 1 and 2. Mol Cell Biol.

[b55] Mendillo ML, Santagata S, Koeva M, Bell GW, Hu R, Tamimi RM, Fraenkel E, Ince TA, Whitesell L, Lindquist S (2012). HSF1 drives a transcriptional program distinct from heat shock to support highly malignant human cancers. Cell.

[b56] Mezger V, Bensaude O, Morange M (1989). Unusual levels of heat shock element-binding activity in embryonal carcinoma cells. Mol Cell Biol.

[b57] Mosser DD, Theodorakis NG, Morimoto RI (1988). Coordinate changes in heat shock element-binding activity and HSP70 gene transcription rates in human cells. Mol Cell Biol.

[b58] Neef DW, Turski ML, Thiele DJ (2010). Modulation of heat shock transcription factor 1 as a therapeutic target for small molecule intervention in neurodegenerative disease. PLoS Biol.

[b59] Neef DW, Jaeger AM, Thiele DJ (2011). Heat shock transcription factor 1 as a therapeutic target in neurodegenerative diseases. Nat Rev Drug Discov.

[b60] Newton EM, Knauf U, Green M, Kingston RE (1996). The regulatory domain of human heat shock factor 1 is sufficient to sense heat stress. Mol Cell Biol.

[b61] Olney JW, Tenkovaa T, Dikraniana K, Qina Y-Q, Labruyerea J, Ikonomidou C (2002). Ethanol-induced apoptotic neurodegeneration in the developing C57BL/6 mouse brain. Dev Brain Res.

[b62] Östling P, Björk JK, Roos-Mattjus P, Mezger V, Sistonen L (2007). Heat shock factor 2 (HSF2) contributes to inducible expression of hsp genes through interplay with HSF1. J Biol Chem.

[b63] Pignataro L, Miller AN, Ma L, Midha S, Protiva P, Herrera DG, Harrison NL (2007). Alcohol regulates gene expression in neurons via activation of heat shock factor 1. J Neurosci.

[b64] Rallu M, Loones M, Lallemand Y, Morimoto R, Morange M, Mezger V (1997). Function and regulation of heat shock factor 2 during mouse embryogenesis. Proc Natl Acad Sci USA.

[b65] Raychaudhuri S, Loew C, Körner R, Pinkert S, Theis M, Hayer-Hartl M, Buchholz F, Hartl FU (2014). Interplay of acetyltransferase EP300 and the proteasome system in regulating heat shock transcription factor 1. Cell.

[b66] Reiner O, Sapir T (2009). Polarity regulation in migrating neurons in the cortex. Mol Neurobiol.

[b67] Rubert G, Miñana R, Pascual M, Guerri C (2006). Ethanol exposure during embryogenesis decreases the radial glial progenitor pool and affects the generation of neurons and astrocytes. J Neurosci Res.

[b68] Sandqvist A, Björk JK, Åkerfelt M, Chitikova Z, Grichine A, Vourc'h C, Jolly C, Tiina A, Salminen TA, Nymalm Y (2009). Heterotrimerization of heat-shock factors 1 and 2 provides a transcriptional switch in response to distinct stimuli. Mol Biol Cell.

[b69] Santillano DR, Kumar LS, Prock TL, Camarillo C, Tingling JD, Miranda RC (2005). Ethanol induces cell-cycle activity and reduces stem cell diversity to alter both regenerative capacity and differentiation potential of cerebral cortical neuroepithelial precursors. BMC Neurosci.

[b70] Sapir T, Sapoznik S, Levy T, Finkelshtein D, Shmueli A, Timm T, Mandelkow EM, Reiner O (2008). Accurate balance of the polarity kinase MARK2/Par-1 is required for proper cortical neuronal migration. J Neurosci.

[b71] Sapir T, Frotscher M, Levy T, Mandelkow EM, Reiner O (2012). Tau's role in the developing brain: implications for intellectual disability. Hum Mol Genet.

[b72] Sarge KD, Murphy SP, Morimoto RI (1993). Activation of heat shock gene transcription by heat shock factor 1 involves oligomerization, acquisition of DNA-binding activity, and nuclear localization and can occur in the absence of stress. Mol Cell Biol.

[b73] Shinkawa T, Tan K, Fujimoto M, Hayashida N, Yamamoto K, Takaki E, Takii R, Prakasam R, Inouye S, Mezger V (2011). Heat shock factor 2 is required for maintaining proteostasis against febrile-range thermal stress and polyglutamine aggregation. Mol Biol Cell.

[b74] Sistonen L, Sarge KD, Morimoto RI (1994). Human heat shock factors 1 and 2 are differentially activated and can synergistically induce hsp70 gene transcription. Mol Cell Biol.

[b75] Sullivan EK, Weirich CS, Guyon JR, Sif S, Kingston RE (2001). Transcriptional activation domains of human heat shock factor 1 recruit human SWI/SNF. Mol Cell Biol.

[b76] Tanaka T, Serneo FF, Tseng HC, Kulkarni AB, Tsai LH, Gleeson JG (2004). Cdk5 phosphorylation of doublecortin ser297 regulates its effect on neuronal migration. Neuron.

[b502] Takahashi Y, Rayman JB, Dynlacht BD (2000). Analysis of promoter binding by the E2F and pRB families in vivo: Distinct E2F proteins mediate activation and repression. Genes & Dev.

[b77] Thompson BL, Levitt P, Stanwood GD (2009). Prenatal exposure to drugs: effects on brain development and implications for policy and education. Nat Rev Neurosci.

[b78] Thomson S, Hollis A, Hazzalin CA, Mahadevan LC (2004). Distinct stimulus-specific histone modifications at Hsp70 chromatin targeted by the transcription factor HSF1. Mol Cell.

[b79] Valenzuela CF, Morton RA, Diaz MR, Topper L (2012). Does moderate drinking harm the fetal brain? Insights from animal models. Trends Neurosci.

[b503] Vihervaara A, Sergelius C, Vasara J, Blom MA, Elsing AN, Roos-Mattjus P, Sistonen L (2013). Transcriptional response to stress in the dynamic chromatin environment of cycling and mitotic cells. Proc Natl Acad Sci U S A.

[b80] Wang G, Zhang J, Moskophidis D, Mivechi NF (2003). Targeted disruption of the heat shock transcription factor (hsf)-2 gene results in increased embryonic lethality, neuronal defects, and reduced spermatogenesis. Genesis.

[b81] Westerheide SD, Morimoto RI (2005). Heat shock response modulators as therapeutic tools for diseases of protein conformation. J Biol Chem.

[b82] Westerheide SD, Anckar J, Stevens SM, Sistonen L, Morimoto RI (2009). Stress-inducible regulation of heat shock factor 1 by the deacetylase SIRT1. Science.

[b83] Whitesell L, Lindquist S (2009). Inhibiting the transcription factor HSF1 as an anticancer strategy. Expert Opin Ther Targets.

[b84] Wynshaw-Boris A (2007). Lissencephaly and LIS1: insights into the molecular mechanisms of neuronal migration and development. Clin Genet.

[b85] Zhou FC, Sari Y, Zhang JK, Goodlett CR, Li T-K (2001). Prenatal alcohol exposure retards the migration and development of serotonin neurons in fetal C57BL mice. Dev Brain Res.

