# Supplementary material for: Heat shock factor 2 is a stress-responsive mediator of neuronal migration defects in models of fetal alcohol syndrome
Source: EMBO Mol Med. 2014 Jul 15;6(8):1043–61. doi: 10.15252/emmm.201303311 (PMC4154132; doi:10.15252/emmm.201303311)
Supplement: Supplementary file 1 [file emmm0006-1043-sd1.pdf]

Supplementary Information

TABLE OF CONTENTS

Supplementary Experimental Procedures **p. 1**

Cell culture and experimental treatments *p. 1*

In situ hybridization *p. 2*

Wound Healing Assay *p. 2*

Immunocytochemistry *p. 3*

Western blot analysis *p. 3*

Nuclear/cytosolic fractionation *p. 3*

Immunoprecipitation *p. 4*

In vitro EGS cross-linking *p. 4*

Transfection and SiRNA *p. 5*

Antibodies used in immunohistochemistry *p. 6*

Bioinformatic web sites *p. 6*

Primers for ChIP and RT-qPCR experiments *p. 6*

Supplemental References **p. 7**

Legends to Supplementary Figures **p. 12**

Supplementary Figures S1 to S11

Table S1

Supplementary Experimental Procedures

Cell culture and experimental treatments

F9 embryonal carcinoma cells were cultured DMEM with 15% fetal calf serum, 12% CO2, 37°C. MEFs immortalized with SV40 T and t antigens using plasmid 830Tt (iMEFs; Östling et al, 2007), murine neuroblastoma cells Neuro2A (N2A), and HeLa cells were cultured in DMEM with 10% fetal calf serum, 5% CO2, 37°C. Cells were exposed to EtOH or heat for 15 min to 24 h. EtOH was added directly to the culture medium. EtOH concentrations were determined as described in the paper. Cells were subjected to HS at 42°C in a water bath, or, alternatively into a cell incubator for long kinetics (from 3 to 24 h).

N2A cells in 24-well plates were transfected with *Hsp70 promoter-Firely Luciferase* and *Renilla Luciferase* expression vectors (ratio 1:5), using Lipofectamine 2000 (Invitrogen). After 24 hrs, the transfectants were exposed to EtOH (0.2 or 0.5%) or HS (42°C) treatments for the indicated times. Luciferase assays were performed with the Dual Luciferase Reporter Assay System (Promega) according to the manufacturer's instructions and firefly luciferase activity value was normalized to *Renilla* activity value. N2A cells, at 70% confluency, were cotransfected (ratio 1:1) with expression plasmids encoding Myc-epitope-tagged HSF1 (Holmberg et al, 2001) and green fluorescent protein (GFP)-fused expression plasmid SUMO-1 (Poukka et al, 2000) using Lipofectamine 2000 (Invitrogen) in Opti-MEM medium (Invitrogen) with a total of 24 μg DNA per 60 cm^2 dish. The green fluorescent protein (GFP)-fused expression plasmids SUMO-1 WT (kindly provided by Hetti Poukka, University of Helsinki, Helsinki, Finland), as well as the Myc epitope-tagged human HSF1 expression plasmid, have been described by Holmberg et al (2001). HDAC inhibitors were added 3 hours before and during exposure to HS or EtOH (trichostatin (TSA), 1μM; valproic acid (VPA) 1 mM; nicotinamide (NAM) 10 mM).

For rescue experiments in iMEFs, Hsf2α and β cDNA, cloned from E16.5 mouse brain, were inserted into vector PCEMM_CTAP (Euroscarf P30536) with CMV-driven expression of insert and GFP used as an indirect reporter (IRES). Retrovirus production and iMEFs transduction was performed as described (Sandrin & Cosset, 2006).

In situ hybridization

Radioactive labelled probe for HSF1 or HSF2 corresponded to full-length cDNA (Sarge et al, 1991; Goodson et al, 1995). Radioactive ISH was performed as described (Wilkinson, 1992).

Wound Healing Assay

Independent clones of immortalized MEFs (iMEFs isolated after immortalization of MEFs by SV40 T and t) from *Hsf2*^{tm1Mmr} (3 *Hsf2*$^{+/+}$ and 3 *Hsf2*$^{-/-}$; Östling et al, 2007) or from the *Hsf2*^{tm1Ijb} knockout mice (1 *Hsf2*$^{+/+}$ and 1 *Hsf2*$^{-/-}$, immortalized according to the same protocol) were cultivated in DMEM high glucose pyruvate glutamax (Invitrogen) complemented with 10% FBS and antibiotics. Three to four independent experiments for each clone were performed. Subconfluent cultures were wounded by about 16 linear scratches (0.1 cm wide) per 10cm^2 dish, and subsequently treated with 1% EtOH. After 24h, control and EtOH-treated cells were stained with Coomassie blue and rinsed twice by PBS. Between 50 and 100 pictures per culture dish (the totality of scratch length) were taken by a Leica DFC420 C camera and binocular Leica S6D (magnification 16x). Either cell numbers that have migrated in the scratched zone, or the surface covered by these cells was calculated for each clone with Image J software. The same results were obtained with both methods.

Cell proliferation after 24 hrs was determined after Coomassie blue staining and by cell counting using the same method. No significant differences in proliferation were observed between untreated and EtOH treated samples.

Immunocytochemistry

HeLa cells were fixed with ice-cold acetone for 8-10 min. at -20°C and then submitted to immunocytochemistry with rabbit polyclonal anti-HSF1 (1/500; rabbit polyclonal Abcam) or mouse monoclonal anti-SUMO-1 (1/500; Zymed). Secondary antibodies: anti-rabbit Alexa488 and anti-mouse-Texas red. Pictures were taken on Zeiss, magnification 63x to 100x.

Western blot analysis

Tissue extracts and whole-cell extracts were prepared as described by Mosser et al (1988) or Loones et al (2000). 30–40 µg of proteins per sample was used for 9% SDS-PAGE. Anti-HSF1 monoclonal antibody was used at 1:2500 dilution (Ab4, Abcam ab81279 or ThermoFisher AB4-RT629); anti-HSF2 antibody at 1:2500 (3E2, Abcam); anti-SUMO-1 (GNP1, 33-2400, Invitrogen) was used at 1:400 dilution for tissue extracts; 1:1500 for whole cell extracts; anti-GFP (Santa Cruz, sc-3996) was used at 1:1500; anti-actin at 1:5000 (Ab20272, Abcam), anti-HSC70 at 1:5000 (SPA-815, Stressgen). In supplementary Fig S1B, HSF2 was detected using polyclonal antibody SFI57 (Östling et al., 2007; 1:2000 dilution) and HSF1 with rabbit anti-HSF1 (Santa Cruz sc-9144; 1:2000 dilution). Hyperphosphorylation of HSF1 was checked on 7% SDS-PAGE. Calf intestinal phosphatase (Biolabs) was used for 1 h at 37°C, according to the manufacturer recommendations.

Nuclear/cytosolic fractionation

Nuclear/cytosolic fractionation was done following a modified protocol from Mendéz and Stillman (2000). Three E16.5 mouse cortices were resuspended at 4°C in 100µL fresh Buffer A (10mM Hepes pH7.9; 10mM KCl; 0.34M sucrose, 10%glycerol, 1 mM DTT, 1 mM PMSF,

0.03% NP40, 0.15mM spermine, 0.15mM Spermidine, 1x phosphatases and protease inhibitors cocktails (Roche)). Extracts were incubated for 5 min on ice. Nuclei were collected in pellet 1 (P1) by low-speed centrifugation (4 min, 1,300 × *g*, 4°C). The supernatant (S1) was further clarified by high-speed centrifugation (15 min, 20,000 × *g*, 4°C) to remove cell debris and insoluble aggregates and constitute the cytosolic extract. Nuclei were washed once in buffer A, resuspended in 125μL extraction buffer (10mM Hepes pH7.9; 0.4M NaCl; 0.1mM EGTA, 5%glycerol, 0.5 mM DTT, 0.5mM mM PMSF, 1x phosphatase and protease inhibitors cocktails (Roche)) and were then submitted to 2–4 rapid freeze-thaw cycles, as described (Mezger et al., 1989). High-speed centrifugation (30 min, 20,000 × *g*, 4°C) clarified supernatant (S2) that constitute the soluble nuclei extract used for western blot. The following antibodies were used: anti- Phospho-Ser^{326}-HSF1 (ADI-SPA-902, Enzo Life Sciences; 1:1000); anti-Histone H3 (Abcam ab1791; 1:1500).

Immunoprecipitation

HSF was immunoprecipitated from 300 µg of total extracts from embryonic cortices or cell extracts with 5 µL of anti-HSF antibodies (1 μg). Conversely, immunoprecipitation was performed with 5 µL (1 µg) of anti-acetyl-Lysine (Millipore, AB3879) or of anti-SUMO-1 (GNP1, 33-2400, Invitrogen) and blotted against anti-HSF1 (1:2000). The pull-down was performed using Agarose beads coupled to protein G according to the manufacturer's instructions (Roche). For HSP70 or HSP90 detection, mouse mAbs (SPA820 P conjugate) were purchased from and used as described (StressGen). In Fig 3D, anti-HSF1 monoclonal antibody (Ab81279) was used at a 1:2500 dilution. Anti-SUMO-1 was used at 1:400.

In vitro EGS cross-linking

EGS (Ethylene glycol disuccinate bis (sulfo-N-succinimidyl) ester sodium salt (1); Sigma

Aldrich, 39104), was added from 1 mM to 10 mM (for cells) and up to 20 mM (for fetal cortices) final concentrations, followed by incubation at 22°C for 30 min. After quenching of the cross-linking reactions with an excess of glycine (75mM final concentration), samples were resolved by electrophoresis through a 9% or 7% SDS-polyacrylamide gel and analysed by immunoblotting with antibodies specific to HSF1 (1:2500; rat monoclonal, Abcam ab81279) or HSF2 (1/2500, 3E2, rat monoclonal, Abcam ab44824).

Transfection and SiRNA

SiRNAs was transfected using Lipofectamine 2000 (Invitrogen) according to the manufacturer's instructions. Functional, non-targeting control siRNA (Dharmacon) was used as negative control (D-001810). The targeted sequences of the *Hsf2* gene were: #1: 5'GAGGGGAGUACAACUGCAUU3'; #2: 5"CAGCAGGACUGAAGGUUUAUU3'. For each well, 500 pmol RNAi duplex in 250 μl Opti-MEM (Invitrogen, 31985), and 5 μl of lipofectamine in 250 μl Opti-MEM media were combined and mixed gently. After 15 min at room temperature, the RNAi mixture was added to each well containing cells. Medium was changed 6 h after transfection, and cells were incubated for an additional 24 h prior to the experiments.

Antibodies used in immunohistochemistry

The following antibodies and dilution used for immunohistochemistry and further detected using avidin-biotin Vector kits, using Diaminobenzidine as a chromogen.

Antibody	Manufacturer	Dilution
HSF1	Neomarker rt-629	1:50
HSF1	Abcam ab47369	1:100
HSF2	Abcam ab44824	1:10

Bioinformatic web sites

Transfac (http://www.gene.regulation.com)

Genomatix (http://www.genomatix.de)

iHOP (http://www.ihop-net.org)

ENSEMBL (http://ensembl.org)

These web sites were used for the search of HSE in HSF2 target genes or primer design.

Primers for ChIP and RT-qPCR experiments

**ChIP primers**	**Forward**	**Reverse**
Gapdh	5'-TGTGGCCAAGCACTTGTATAAC-3'	5'-TATGTCTGACCAGAGGAGAGCA-3'
Cyclophilin	5'-CGTCGCCTCCGCAACAGTGTC-3'	5'-TCAGCGTCTGGTGCCCTGCTC-3
Hsp70.1	5'-CGTCGCCTCCGCAACAGTGTC-3'	5'-TCAGCGTCTGGTGCCCTGCTC-3'
Hsp90	5'-CGGTTGGGGAGGGTTCTTCC-3'	5'-CCGCACCAGGACACTGAAGCA-3'
*Dclk*1	5'-CCCACACACAGCGCCCACTG-3'	5'-TCACATTATCCGAGAGAGTTCGG-3'
Dcx	5' -CTAAGAGAAAGGGCTAGCACATTG- 3'	CGCACAGGAAATTGCAGGGTAG
p35	5'-TCC GGA GAG GCG GCT TGC A-3'	5'-TTA CCC GCA GTC AGG CAG GC-3'
Chl1	5' -TCTTCTGAGAATGAAAGTGTTATTGTG-3'	5' -TTATGGTCTATTTGTGCACTCACT- 3'
Myo10	5' –GAGGGAGGGTGATGAGAAAA- 3'<br>or<br>5' - CAGGAAACGCGGTAAAAGG- 3'	5' - CCGGCTCACACTTCGTTAG- 3'<br>or<br>5' - GACTTGAAAGCCCAATGAGG-3'
MapT	5'-TTGCTCTCCAGGGATCTGTT- 3'	5'-GAGGGAGGGTGATGAGAAAA- 3'
Mark2	5'- TTGCCAAGAGCTGAGCAGT-3'	5' -TTGCTCTCCAGGGATCTGTT-3'

**RT-qPCR primers**	**Forward**	**Reverse**
Hsp70.1 (Hspa1a)	5' -GGCCACATTGTTGATACATGC- 3'	5'-CTACAGTGCAACCACCATGC- 3'
Hsp90	5' -GCTGAAGGAGACCCAGAAG- 3'	5' -CACTGAGACCAGGCTCTTCC- 3'
Dclk1	5'-GCTCTTGATAAGGAGAGGCAGG- 3'	5'-CGAACAGTCTCAGAGGAGCTGG-3'
Dcx	5'-CAACACCTAAGTCAAAGCAGTCTCC- 3'	5'-TCGCCAAGTGAATCAGAGTCATCC-3'
Dclk2	5' –CCTCATCAACTCCTTGCTGAAAG- 3'	5' -GACATGGCCAGGAACTAGAGAG- 3'
Nde1	5'-GGACGAGGAACCAAAAACAGAG-3'	5'-AGTAAGGAAGACAGGGGAGGG-3'
Ndel1	5' - TGGAGCGAGCAAAAAGGGCAAC- 3'	5' – GACCGCAGAATCCATCTTCTCAC- 3'
NudC	5' -AACCGCTTGGTTACCAGTGA- 3'	5' -GCGAGTCTCACTGTCGAGGT- 3'
Lis1	5' -CTGGACCTTTCTTGCTATCTGG- 3'	5' -TCTTATAATCCCATACACGGAGG- 3'
p35	5'-CACTAGTGAGCTGCTGCGCTG-3'	5'-ACCACATTGGCCGGTGTGATGA- 3'
p39	5' -ACTGCTCCGCTGCCTCCGCGA- 3'	5' -GCGGCCTGCAGTTCCGCGGC- 3'
Cdk5rap2	5' –CACCATATGCTGGGCCTGAT- 3'	5' -GCTCAGAGCCTCTTGGGTTT- 3'
Myo10	5' -CCTTGGAGCAGAAACTGGAA- 3'	*5'* – CGTGCCAAGTAGCCTAAGATG- *3'*
MapT	5'-CGAAGATCGCCACACCTC- 3'	5' -TGACCCTGGAGGAGTCTTAGG- 3'
Mark2	5' -GGGACCAGCAGAATCTACCC- 3'	5' -GCTGAAGATGCTGCCAGAG- 3'
Dync1h1	5' -CTAACAGCTGGTCCCTGGAG- 3'	5' -AGGACTGTGGAGATGGCATT- 3'
Rttn	5' -GTTACTCTTCTGGCTGCCTGTT- 3'	5' -GTTAGAGCCCAAAGTGAAGCTG- 3'
Tubb2b	5' –CAAGATGTCAGCCACCTTCA- 3'	5' –GCCGGAACATAGCAGTGAAC- 3'

Supplemental References

Cappello S, Monzo P, Vallee RB (2011) NudC is required for interkinetic nuclear migration and neuronal migration during neocortical development. Dev Biol 357: 326-335

Clos J, Westwood JT, Becker PB, Wilson S, Lambert K, Wu C (1990) Molecular cloning and expression of a hexameric Drosophila heat shock factor subject to negative regulation. Cell 63: 1085-1097

Cotto J, Fox S, Morimoto R (1997) HSF1 granules: a novel stress-induced nuclear compartment of human cells. J Cell Sci 110: 2925-2934

Edelman AM, Kim WY, Higgins D, Goldstein EG, Oberdoerster M, Sigurdson W (2005) Doublecortin kinase-2, a novel doublecortin-related protein kinase associated with terminal segments of axons and dendrites. J Biol Chem 280: 8531-8543

Fiorenza MT, Farkas T, Dissing M, Kolding D, Zimarino V (1995) Complex expression of murine heat shock transcription factors. Nucleic Acids Res 23: 467-474

Goodson ML, Sarge KD (1995) Regulated expression of heat shock factor 1 isoforms with distinct leucine zipper arrays via tissue-dependent alternative splicing. Biochem Biophys Res Commun 211: 943-949

Holmberg CI, Hietakangas V, Mikhailov A, Rantanen JO, Kallio M, Meinander A, Hellman J, Morrice N, MacKintosh C, Morimoto RI, Eriksson JE, Sistonen L (2001) Phosphorylation of serine 230 promotes inducible transcriptional activity of heat shock factor 1. EMBO J 20: 3800-3810

Jaglin XH, Poirier K, Saillour Y, Buhler E, Tian G, Bahi-Buisson N, Fallet-Bianco C, Phan-Dinh-Tuy F, Kong XP, Bomont P, Castelnau-Ptakhine L, Odent S, Loget P, Kossorotoff M, Snoeck I, Plessis G, Parent P, Beldjord C, Cardoso C, Represa A, Flint J, Keays DA, Cowan NJ, Chelly J (2009) Mutations in the beta-tubulin gene TUBB2B result in asymmetrical polymicrogyria. Nat Genet 41: 746-752

Kamiya A, Kubo K, Tomoda T, Takaki M, Youn R, Ozeki Y, Sawamura N, Park U, Kudo C, Okawa M, Ross CA, Hatten ME, Nakajima K, Sawa A (2005) A schizophrenia-associated mutation of DISC1 perturbs cerebral cortex development. Nat Cell Biol 7: 1167-1178

Karl C, Couillard-Despres S, Prang P, Munding M, Kilb W, Brigadski T, Plötz S, Mages W, Luhmann H, Winkler J, Bogdahn U, Aigner L (2005) Neuronal precursor-specific activity of a human doublecortin regulatory sequence. J Neurochem 92: 264-282.

Kerjan G, Koizumi H, Han EB, Dubé CM, Djakovic SN, Patrick GN, Baram TZ, Heinemann SF, Gleeson JG (2009) Mice lacking doublecortin and doublecortin-like kinase 2 display altered hippocampal neuronal maturation and spontaneous seizures. Proc Natl Acad Sci U S A 106: 6766-6671

Kheradmand Kia S1, Verbeek E, Engelen E, Schot R, Poot RA, de Coo IF, Lequin MH, Poulton CJ, Pourfarzad F, Grosveld FG, Brehm A, de Wit MC, Oegema R, Dobyns WB, Verheijen FW, Mancini GM (2012) RTTN mutations link primary cilia function to organization of the human cerebral cortex. Am J Hum Genet 91: 533-540

Loones MT, Chang Y, Morange M (2000) The distribution of heat shock proteins in the nervous system of the unstressed mouse embryo suggests a role in neuronal and non-neuronal differentiation. Cell Stress Chaperones 5: 291-305

Méndez J, Stillman B (2000) Chromatin association of human origin recognition complex, cdc6, and minichromosome maintenance proteins during the cell cycle: assembly of prereplication complexes in late mitosis. Mol Cell Biol 20:8602-8612.

Poirier K, Lebrun N, Broix L, Tian G, Saillour Y, Boscheron C, Parrini E, Valence S, Pierre BS, Oger M, Lacombe D, Geneviève D, Fontana E, Darra F, Cances C, Barth M, Bonneau D, Bernadina BD, N'guyen S, Gitiaux C, Parent P, des Portes V, Pedespan JM, Legrez V, Castelnau-Ptakine L, Nitschke P, Hieu T, Masson C, Zelenika D, Andrieux A, Francis F, Guerrini R, Cowan NJ, Bahi-Buisson N, Chelly J (2013) Mutations in TUBG1, DYNC1H1,

KIF5C and KIF2A cause malformations of cortical development and microcephaly. Nat Genet 45: 639-647

Poukka H, Karvonen U, Janne OA, Palvimo JJ (2000) Covalent modification of the androgen receptor by small ubiquitin-like modifier 1 (SUMO-1). Proc Natl Acad Sci U S A 97: 14145-14150

Sandrin V, Cosset FL (2006) Intracellular versus cell surface assembly of retroviral pseudotypes is determined by the cellular localization of the viral glycoprotein, its capacity to interact with Gag, and the expression of the Nef protein. J Biol Chem 281: 528-542

Sarge K, Zimarino V, Holm K, Wu C, Morimoto RI (1991) Cloning and Characterization of Two Mouse Heat Shock Transcription Factors with Distinct Inducible and Constitutive DNA Binding Ability. Genes Dev 5: 1902-1911

Takitoh T, Kumamoto K, Wang CC, Sato M, Toba S, Wynshaw-Boris A, Hirotsune S (2012) Activation of Aurora-A is essential for neuronal migration via modulation of microtubule organization. J Neurosci. 32:11050-11066

Wilkinson DG (1992) in *Whole Mount in Situ Hybridization of Vertebrate Embryos*, ed. Wilkinson D G (Oxford Univ. Press, New York), pp. 75–83

Xiao H, Perisic O, Lis JT (1991) Cooperative binding of Drosophila heat shock factor to arrays of a conserved 5 bp unit. Cell 64: 585-593

Young-Pearse TL, Suth S, Luth ES, Sawa A, Selkoe DJ (2010) Biochemical and functional interaction of disrupted-in-schizophrenia 1 and amyloid precursor protein regulates neuronal migration during mammalian cortical development. J Neurosci 30:10431-10440

Legends to Supplementary Figures

Figure S1. Modes of chronic prenatal alcohol exposure, HSF1 and HSF2 expression profiles in the developing brain, and induction of HSF1 DNA-binding activity and persistence of HSF2 DNA-binding activity in the fetal cortex exposed to alcohol (related to Fig 1).

(A) **Effect of different modes of chronic fetal alcohol exposure on BrdU incorporation:** feeding with alcohol-containing food (CAI), daily force-feeding (gavage, GAV), or twice-daily chronic intraperitoneal injections (CIP). (Left) Quantification of BrdU-labelled cells at E16.5, per 0.05 mm^2 (n = 5 different section on 5 different animals; upper panel; p-values: CAI, p= 0.0049; GAV, p = 0.0011; CIP = 0.0099) or per 100 cells (lower panel; p-values: CAI, p= 0.0010; GAV, p = 0.0531; CIP, p = 0.0153). A decrease in BrdU incorporation upon FAE was observed in the germinal proliferative zones (gz). *: $p \leq 0.05$; **: $p \leq 0.01$. Data information: Differences were considered statistically significant when P values were <0.05. When Anova was found significative (p<0.05) we performed the Student's *t*-test with Bonferroni adjustment. Data are presented as mean ± s.e.m. (Right) Representative images corresponding to the left panels (scale bars, 10 μM).

(B and C) **HSF1 is ubiquitously expressed in the developing brain.** (B) Upper and middle panel: WB analysis of whole-cell protein extracts from fetal mouse telencephala (E13) or cortices (E15-E18) at different stages, with anti-HSF1 or anti-HSF2 antibodies. For each stage, equal loading (40 µg) was verified by antibodies against actin. Tel, telencephalon; Fl, forelimb; Hl, hindlimb; N2A, Neuro2A neuroblastoma cells (positive control). Lower panel: major HSF2 isoforms α and β are indicated (Fiorenza et al, 1995; Goodson et al, 1995); F9 embryonal carcinoma cells (positive control; Rallu et al, 1997).

(C) (Left) *In situ* hybridization analysis of *Hsf1* and *Hsf2* mRNA levels (white signal) in parasagittal sections of the mesencephalon at E14.5. vz, ventricular zone; ven, ventricle.

(Right) Immunohistochemical detection of HSF1 or HSF2 in the cortex at E16.5. cp, cortical plate (brown signal). Arrowhead points to outer layers.

(D) **Chronic EtOH exposure by gavage or twice-daily intraperitoneal injections activates HSF1 and keeps HSF2 active in the fetal cortex.** Gel-shift assays of HSF1 and HSF2 DNA-binding activities using supershifting with HSF1 (α1) or HSF2 antibodies (α2) in E16.5 cortices after gavage (GAV), or twice-daily intraperitoneal injections (CIP). The specific supershifting induced by the addition of anti-HSF1 (α1) or anti-HSF2 antibodies (α2) is illustrated by black and white arrowheads, respectively. HSF-HSE, CHBA, NS, free: as in Figure 1B. The specific supershift induced by the addition of anti-HSF1 (α1) or anti-HSF2 antibodies (α2), was illustrated by red and blue bars, respectively (no antibody: black bars), as in Fig 4.

Supplemental information relative to Figure S1 (related to Fig 1).

HSF1 is expressed in the neuroepithelium of various species (reviewed in Abane & Mezger, 2010). We observed that HSF1 was expressed at each stage of corticogenesis, as was HSF2 (Fig S1B). As previously described (Rallu et al, 1997; Kallio et al, 2002; Chang et al, 2006), HSF2 expression was restricted to the proliferative zones of the brain (FigS1 C, left panel), including the cortex, and was also expressed in the upper layers of the developing cortical plate from E15.5, (cp; FigS1 C, right panel; arrow). In contrast, *Hsf1* mRNA and protein were ubiquitously expressed in the proliferative and non-proliferative zones of the developing brain, including the cortex (Fig S1C).

Figure S2. HSF1 nuclear localization increases after CAI.

(A) Representative images of subcellular localization of HSF1 detected by immunohistochemistry. Lateral cortex from control (CTR) and CAI fetuses at E16.5 (magnification 4 100x; Scale bars, 10 µM).

(B) Quantification of the number of HSF1-positive nuclei before or after CAI (**, $p < 0.01$).

(C) **Representative immunoblot analysis of nuclear-cytoplasmic subcellular distribution of Phospho-Ser326-HSF1 after fractionation** of control (CTR) and E16.5 cortices exposed to CAI, and the corresponding quantification (n=2).

Supplemental information relative to Figure S2 (related to Fig 1).

The number of neurons showing nuclear HSF1 labelling increases significantly in cortices treated with CAI (FigS2A and B). To verify that HSF1 translocated to the nucleus after CAI was in an active form, we performed nuclear-cytoplasmic fractionation of E16.5 cortices and checked for the presence of HSF1 phosphorylated on serine 326 (conserved between human and mouse species) using an antibody against PhosphoSer^{326}-HSF1. An increase in the signal corresponding to PhosphoSer^{326}-HSF1 was observed in the nucleus after CAI, but not in the cytoplasm (although an increase in total HSF1 protein levels was observed in the cytoplasm). Some "leaking" of nuclear material was observed in the cytoplasmic fraction as often occurs with tissues, however, despite this, an enrichment of the active PhosphoSer^{326}-HSF1 form was observed in the nucleus (and not in the cytoplasm). We therefore concluded that the nuclei of cortical neurons are enriched in an active form of HSF1 after CAI, in line with Hashimoto-Torii *et al*, 2014.

Figure S3. (A) **Schematic representation of HSE sites in mouse *p35*, *Dcx*, *Dclk1, Chl1, MapT, Myo10,* and *Mark2* genes**. The position of the HSEs, which was determined using Genomatix software and conserved between at least 3 mammalian species (humans, mice and rats or humans, cows and mice) and that were found to be bound by HSF2 in our ChIP experiments (*p35*, *Dcx*, *Dclk1*, *Chl1* Fig 2A and see also Fig S8C) or in ChIPSeq experiments (*Myo10*, *MapT*, and *Mark2*; Mendillo *et al*, 2012; Vihervaara *et al*, 2013) and whose expression was affected by fetal alcohol exposure in an HSF2-dependent manner (Fig 6C), are schematically indicated. For *p35*, where GC boxes and the HSE have already been described in the literature, they are indicated as grey boxes as in Chang et al., 2006). For the other genes, HSEs are illustrated by grey ellipses. For *Dcx*, the HSE was positioned at -160 nucleotides from the beginning of the 5'-UTR in a region known to be important for *Dcx* expression (Karl *et al*, 2005). In the case of *Dclk1*, the HSE was positioned according to the ATG (+171 nucleotides downstream). In *Myo10*, the HSE was positioned at +814 nucleotides from the transcription start, for *Mark2*, the first intron at +8536 nucleotides from the beginning of the 5'-UTR. The sequence of the HSE (in red) in *p35* and *Chl1* allows for the binding of only one HSF trimer.

Figure S4. CAI results in a decrease in p35 protein levels, in line with reduction in *p35* mRNA levels in fetal cortices.

(Left) Representative immunoblot of p35 protein expression in cortices in *Hsf2*$^{+/+}$ mice before (CTR) or after CAI, and comparison with the levels of p35 in *Hsf2*$^{-/-}$ control cortices. (Right) quantification (n= 2 experiments).

Figure S5. Alcohol exposure activates HSF1 and HSF2 iMEFs. (Related to Fig 3).

(A and B) **Ethanol exposure induces HSF1 and HSF2 DNA-binding activity in iMEFs**. Gel-shift analyses of HSF1 and HSF2 DNA-binding activities in immortalized *Hsf2*$^{+/+}$ iMEFs, using supershifting with anti-HSF1 (α1; black arrowhead) or -HSF2 antibodies (α2; white arrowhead) after *ex vivo* exposure to 1% EtOH for 3 hours (A; 1-2% is the optimal EtOH concentration for HSF1 induction in iMEFs) or after a 1-hour HS at 42°C (B). HSF-HSE, CHBA, NS, free: as in Fig 1.

(C) **ChIP analysis of the recruitment of HSF1 and HSF2 by the *Hsp70* promoter** in control iMEFs (CTR), after HS (42°C) or 1% EtOH exposure (35 min). *Cyclo*: the *Cyclophilin B gene* that does not contain HSE was used as a negative control. *Hsf2*$^{-/-}$ iMEFs were used as a negative control for *in vivo* HSF2 binding.

(D and E) **EtOH induces persistent HSF1 DNA-binding activity *ex vivo.***

(D) Gel-shift assays of the kinetics of induction of HSF DNA-binding activity upon *ex vivo* treatment of *Hsf2*-/- iMEFs by either a 41°C HS or 2% EtOH. HSF-HSE, CHBA, NS, free, α1: as in Fig 1.

(E) Quantification of the intensity of the HSF1-HSE complex in two additional independent kinetic experiments.

(F) **Induction of Hsp70 by ethanol exposure in iMEFs.** (Left) **Quantitative RT-PCR analysis of *Hsp70* mRNA levels** upon exposure to different EtOH concentrations (upper panel) or HS at 41°C for 0.5 or 1.0 hour, without or with 1.0 hour of recovery (1.0+1.0 R) at 37°C (lower panel). n= 6. *: $p < 0.001$, **: p= 0.00189, and ***: p= 0.0013. The increase in *Hsp70* mRNA levels with 0.2 and 0.5% EtOH was not statistically significant (upper panel) in the case of iMEFs. Data information: Differences were considered statistically significant when *P* values were <0.05, using unpaired two-way Student's *t*-test. Data are presented as mean ± s.e.m. (Right) **Western blot analysis of Hsp70 accumulation** after EtOH exposure at

different concentrations (1 hour; upper panel) or HS stress (1 hour at 42°C) and 3 h of recovery (lower panel). Tub: tubulin.

Supplemental information relative to Figure S5 (related to Fig 3).

We analyzed the HSE-binding activities of HSFs in iMEFs upon EtOH exposure: iMEFs displayed an increase in HSF DNA-binding activity to heat shock elements (HSE) in gel-shift assays, with the contribution of HSF1 as well as HSF2, as indicated by the supershifting of the whole HSF-HSE complex by either anti-HSF1 or HSF2-antibodies (Fig S5A; black and white arrowheads, respectively). In contrast, heat-shocked iMEFs typically showed only HSF1 activation, as expected in gel-shift assays (Fig S5B; black and white arrowheads, respectively). By ChIP, we observed that the *in vivo* binding of HSF2 to the *Hsp70* promoter was increased in iMEFs treated by EtOH as it is upon HS (Fig S5C; see also Östling et al, 2007; Ahlskog et al, 2010). In addition, HSF1 DNA-binding activity is persistently induced by alcohol exposure (Fig S5D and E).

HSF1 and HSF2 binding at the *Hsp70* promoter region was followed by a 2- and 4-fold increase in *Hsp70* mRNA levels, in response to 1 or 2% EtOH exposure, respectively (Fig S5F, left panel). The elevation in *Hsp70* mRNA and protein levels was not as high as for HS, in line with the lowest DNA-binding activity of HSF observed in EMSA, compared with HS (Fig S5A and B).

These experiments led us to conclude that HSF1 and HSF2 are both activated in terms of DNA-binding in response to *ex vivo* alcohol exposure, and influence the expression of classic *Hsp* target genes.

Figure S6. Persistent HSF1 activation and post-translational modifications.

A) Gel-shift assays from N2A cells exposed to heat shock (42°C; 0.5 h) or EtOH (0.5%, which we have determined is the optimal dose of EtOH to provoke HSF1 activation in N2A cells) for 5 or 7 h.

(B) N2A cells were transfected with an *Hsp70-Luciferase* reporter construct. After 24 hours, transfected cells were exposed to EtOH (0.2 or 0.5%) or HS (42°C) for the indicated times. Values are indicated as the ratio of luciferase activity present in stressed samples versus control samples. The data represent means ± SD from 12 independent experiments performed for each time point and each condition.

(C) Quantitative RT-PCR analysis of *Hsp25*, *Hsp70*, and *Hsp90* mRNA levels in N2A cells exposed to 0.5% EtOH for 1 or 2 hours. (n=3; asterisk, $p<0.001$). P-values for *Hsp25*, p = 0,000567 for 1 hour and p = 0,000467 for 2 hours; for *Hsp40*, p = 0,000912 for 1 hour and p = 0,000601 for 2 hours; for *Hsp70*, p = 0,000334 for 1 jour and p = 0,000167 for 2 hours; for Hsp90, p = 0,000587 for 1 hour and p = 0,000321 for 2 hours. Data information: Differences were considered statistically significant when *P* values were <0.05, using unpaired two-way Student's *t*-test. Data are presented as mean ± s.e.m.

(D) Representative immunoblots (left) and corresponding quantification (n= 2 experiments), showing increased levels of HDAC1 and SIRT1 (but not of HDAC2) in N2A cells exposed to EtOH, that might at least transiently explain the lack of HSF1 acetylation upon EtOH exposure.

(E) (Left) Control for HSF1 hyperphosphorylation in iMEFs: calf intestinal phosphatase (Calf Int. Phos.) abrogates the HS-induced HSF1 migration shift characteristic of HSF1 hyperphosphorylation in WB. (Right) Immunoblotting of HSF1 phosphoSer303/307 (Phos-Ser303/307): iMEFs (HS at 43°C for 15 min or 2% EtOH for 0.5 h).

(F) (Upper panel) Immunoblotting of HSF1 phosphoSer303/307 (Phos-Ser303/307). N2A extracts as in (Fig 3G). Immunoblot analysis of the level of Ser303/307 phosphorylation of HSF1. (Lower panel) Corresponding quantification of the ratio of HSF1 phosphorylation at Ser303/307 in stress versus control situations. Loading control: β-actin.

Figure S7. EtOH induces delayed colocalization of HSF1 and SUMO-1 in nuclear stress bodies, compared with HS (related to Fig 3).

Immunocytochemical detection of HSF1 and SUMO-1. HeLa cells were untreated (C), heat-shocked at 42°C or treated by 0.5% EtOH, and/or allowed to recover (after a 2h stress) at 37°C for the indicated times. White arrows point to nuclear stress bodies (nSBs), in which HSF1 and SUMO-1 are colocalized. Magnification: 63x.

Supplemental information relative to Figure S7. (related to Fig 3).

We confirmed the specific profile of HSF1 sumoylation upon EtOH exposure, by taking advantage of the propensity of human cells to accumulate HSF1 in nuclear stress granules or bodies (nSBs) upon stress. nSBs typically appear as brightly stained irregular subnuclear structures in immunocytochemistry (Cotto et al 1997; Jolly et al 1999; Sarge et al 1993). HSF1 and SUMO-1 are prominently colocalized in nSBs, although only transiently even during prolonged HS (Hietakangas et al, 2003). Accordingly, we found that the distribution of HSF1 was initially diffuse, and did not overlap with that of SUMO-1 in control HeLa cells, but accumulated in nSBs and was colocalized with SUMO-1 upon a 1-h or 2-h HS. This HSF1 localization in nSBs started to disappear at 4 h and was absent during recovery. In contrast, upon EtOH exposure, no nSB could be observed at 1 h (although the diffuse HSF1 pattern was slightly different from what was observed in control cells). nSBs with HSF1 and SUMO-1 colocalization were observed from 2 h of EtOH exposure and persisted both at 4 h,

and after a 3-h recovery period. Similar delayed and persistent HSF1 and SUMO-1 colocalization was obtained with 0.2% or 2% EtOH (data not shown).

Figure S8. Alcohol induces the formation of HSF1-HSF2 heterocomplexes with specific migration signatures in gel-shift assays in F9 cells (related to Fig 4 and 6C (C) for the part).

(A) Gel-shift assays after EtOH exposure (0.5%) or HS at 43°C in F9 embryonal carcinoma cells. In these cells, as in the developing cortex, HSF2 displays high constitutive DNA-binding activity, but HSF1 does not (Rallu et al, 1997). Black, red, and blue bars as in Fig 4.

(B) Corresponding quantification of the intensity and supershifting of HSF-HSE complexes in stressed F9.

(C) **ChIP analysis of the binding of HSF1 and HSF2 to the HSEs of the *Nde1* and *Chl1* *genes* in CTR and CAI situations at E16.5.**

(Upper panels) **Quantification of the occupancy of HSE by HSF1 or HSF2 using ChIP and quantitative PCR** (ratio of the ChIP signal versus input signal) on *Nde1* and *Gapdh* (left) and *Chl1* and *Cyclophiline B* genes in E16.5 fetal cortices from control dams (C) or those subjected to CAI (CAI); for *Nde1* and *Gapdh,* n=6 independent litters for CAI; n=3 independent litters for controls; for *Chl1 and Cyclophiline B*, n=2 independent litters for C and n=4 for CAI. Hatched and solid bars: ChIP with anti-HSF2 and anti-HSF1, respectively. Color code for gene families: *Ndel1* (orange), *Chl1* (dark gray). P-values for *Hsp70*: p = 0.001482 for HSF1 enrichment in CAI compared to C, and p = 0.000686 for HSF2; for *Nde1* (CTR/CAI: for HSF1 p = 0.00291. For *Chl1*: no significant enrichment for HSF1 (p = 0,09675); significant reduction in enrichment for HSF2 in CAI compared to C (p = 0,04123). *** p≤0.001, ** for p≤0.01; * for p≤0.05. as in Fig 2A. For *Chl1*: (p=0,09675). No enrichment of HSF2 or HSF1 for *Gapdh* (p = 0.176985 for HSF1 and p =0.195512 for HSF2) and *Cyclo*

(p= 0.133646 for HSF1 and p=0.152153 for HSF2). Data information: Differences were considered statistically significant when *P* values were <0.05, using unpaired two-way Student's *t*-test. Data are presented as mean ± s.e.m.

(**Lower panel**) *Nde1* HSE is located within the gene body at +14077 nucleotides from the ATG. This HSE can by occupied by only one trimer, which, altogether with our biochemichal analyses, is in favor of binding of both HSF1 and HSF2 as heterotrimers upon CAI.

Supplemental information relative to Figure S8. (related to Fig 4 and 6C).

Nde1 is a gene that is not notably affected by alcohol or by HSF2 (see supplementary Fig 11C). However, it is bound by HSF2 in control conditions and by HSF1 and HSF2 upon CAI. This HSE can by occupied by only one trimer, which, altogether with our biochemical analyses, is in favour of binding of both HSF1 and HSF2 as heterotrimers upon CAI. We therefore used this gene as a model for in vivo binding of HSF1and HSF2 heterotrimers (see working hypothesis, Figure 4A).

Figure S9. Alcohol induces HSF1 and HSF2 oligomerization (related to Fig 4)

(A) WB analysis of the oligomeric state of HSF1 and HSF2 after chemical crosslinking with EGS in N2A whole cell extracts (9% SDS PAGE).

(B) Same as in (A) but run on a 7% SDS PAGE to allow better observation of trimeric forms.

(C) Same as in (B) but with E17.5 cortical extracts from control or CAI fetuses.

Arrow: position of HSF1 or HSF2 monomers. Arrowhead: position of HSF1 or HSF2 trimers. Mono-, di- and trimers are indicated by gray ellipses. Asterisk: band typically observed for HSF1 and HSF2 in E10.5 to E17.5 cortical extracts, possibly derived from alternative splicing, transcription starts, or post-translational modifications. In cortices, both HSF1 and HSF2 were present in complexes of similar sizes, which seemed to extend towards even

higher molecular weights (≥ 250 kDa). In addition, their intensity was enhanced after CAI at 10μM EGS, likely corresponding to trimers or higher order oligomers, which may correspond to interactions between heterotrimers as demonstrated for Drosophila HSF (Clos et al, 1990; Xiao et al, 1991), or between heterotrimers and other proteins. Note that discrete bands were detected in uncrosslinked samples due to difficulties in EGS quenching and, possibly, to the diffusion of the remaining EGS in the 7% gel. Higher concentrations of EGS were necessary to crosslink HSF complexes from embryonic cortices (20 μM).

Supplemental information relative to Figure S9. (related to Fig 4).

HSF2 and HSF1 both participate in trimeric complexes

Since fetal alcohol exposure in embryonic cortices led to both persistent HSF1 and HSF2 binding activities, we asked whether these two molecules co-existed as homo- or heterotrimers. The binding of both HSF1 and HSF2 to the *Nde1* gene, which has an HSE site that cannot accept more than a single trimer (Fig S8C and 4A) was suggestive of the induction of heterotrimer formation upon fetal alcohol exposure. It is known that the activation of either HSF1 or HSF2 is accompanied by homotrimerization (Sarge et al, 1993; Sistonen et al, 1994; Sandqvist et al, 2009). We thus analyzed the oligomeric status of the EtOH-resistant/induced HSF2 and EtOH-induced HSF1 by chemical crosslinking with EGS, followed by WB analysis. In contrast to HS, which did not notably modify HSF2 oligomerization, EtOH exposure led to increased accumulation of HSF2 in dimeric and trimeric forms (>170 kDa and < 250kDa; Supporting Information Fig S9A). Similar results were obtained for HSF1, although in this case, the effect of EtOH was not as strong as that of HS (Supporting Information Fig S9A), in line with the intensities of HSF-DNA complexes in gel-shift assays (Fig 4B). Trimers clearly appeared as the major oligomeric species in low acrylamide percentage gels, upon HS or EtOH treatments (Supporting Information Fig S9B). Importantly,

the presence of HSF1 and HSF2 in alcohol-induced trimers was also observed in fetal cortices from dams treated with CAI (Supporting Information Fig S9C). These trimers could be homotrimers containing only HSF1 or HSF2 or, conversely, heterotrimers.

Figure S10. HSF2 is not necessary for HSF1 activation in iMEFs and protects iMEFs from migration defects in response to alcohol (related to Fig 5 and 6).

(A) **In contrast to its essential role in fetal cortices, HSF2 is not necessary for HSF1 activation in iMEFs.** Gel-shift assays of $Hsf2^{-/-}$ iMEF extracts (from $Hsf2^{tm1Mmr}$ knockout mice), showing that HSF1 activity can be observed and induced by 1.0% EtOH in the absence of HSF2, in contrast to what is observed in $Hsf2^{-/-}$ fetal cortices. HS: 1 hour at 42°C. Note that, as in WT iMEFs, HSF1 activity induced by EtOH is lower than with HS.

(B) **The lack of HSF2 enhances alcohol-induced migration defects in iMEFs.**

(Left) Representative wound healing assays upon 1% EtOH exposure for 2 $Hsf2^{tm1Mmr}$ iMEF clones ($Hsf2^{+/+}$ and $Hsf2^{-/-}$; Kallio et al, 2002) before and after 24 h of migration. (Right) Quantification of the number cells that migrated into the wound (Ratio between values from EtOH-exposed and control cells), for 3 independent clones of each genotype from $Hsf2^{tm1Mmr}$ or one from the $Hsf2^{tm1Ijb}$ knockout model (McMillan et al, 2002); average of the values for $Hsf2^{tm1Mmr}$ clones. Hatched and dotted bars: $Hsf2^{+/+}$ and $Hsf2^{-/-}$, respectively.

(C) **Reintroducing HSF2 in $Hsf2^{-/-}$ iMEFs reinstates alcohol-induced migration defects.** Rescue experiment and quantification as in (B). (Left) Ratio between values from EtOH-exposed and control cells. Stable HSF2 α or β expression in an $Hsf2^{tm1Mmr/tm1Mmr}$ clone diminishes or abolishes EtOH-induced migration defects, respectively. The dotted line indicates a ratio equal to one. (Right) Representative immunoblot of HSF2 α or β expression levels in $Hsf2^{tm1Mmr/tm1Mmr}$ iMEFs stably expressing HSF2 α or β.

(D) Western blot analysis of the HSF1 expression levels in the developing cortex

compared to other organs Tel, telencephalon; FL, forelimbs; HL, hindlimbs. F9 embryonal carcinoma cells were loaded as positive control. Loading control: non-stress inducible Heat shock cognate, Hcs70. **HSF1 expression levels are lower in the developing cortex compared with other organs** (illustrated here at E13.5). In contrast, we previously observed that HSF2 is expressed at high levels in the developing cortex compared with other organs (see Rallu et al, 1997), This means that the HSF1:HSF2 ratio is lower in the developing cortex compared with fore- or hind-limbs.

Supplemental information related to Figure S10. (related to Fig 5 and 6).

HSF2 is not required for HSF1 activation by alcohol exposure in iMEFS

We first examined the response of *Hsf2*$^{-/-}$ iMEFs to ethanol. In contrast to what was seen in the fetal cortex and in N2A cells, the absence of HSF2 did not compromise the activation of HSF1 upon ethanol exposure in iMEFs.

Loss of HSF2 increases the severity of EtOH-induced migration defects in iMEFs

We compared the migration of *Hsf2*$^{+/+}$ and *Hsf2*$^{-/-}$ mouse embryonic fibroblasts (iMEFs) in wound-healing assays, after exposure to EtOH. We analyzed three independent *Hsf2*$^{+/+}$ iMEF clones (Östling et al, 2007) and three independent *Hsf2*$^{-/-}$ clones from the *Hsf2*^{tm1Mmr} knockout (Kallio et al, 2002); and one of each genotype from the *Hsf2*^{tm1Ijb} knockout (McMillan et al, 2002). The migration of *Hsf2*$^{-/-}$ iMEFs was markedly and reproducibly impaired in the presence of EtOH, whereas the migration of *Hsf2*$^{+/+}$ clones was only slightly affected by EtOH (Fig S11C). HSF2 is expressed as two major isoforms, HSF2α and β, which seem to display differential effects on *Hsp* genes (See Fig S1B; Fiorenza et al, 1995; Goodson et al, 1995; He et al, 2003; Leppä et al, 1997). Rescue experiments in one *Hsf2*$^{-/-}$ iMEF clone from the *Hsf2*^{tm1Mmr} knockout showed that the overexpression of HSF2α or HSF2β, respectively, partially or totally abolished the impairment of *Hsf2*$^{-/-}$ iMEFs (Fig S11C). These results

suggested that the migration defects induced by alcohol in iMEFs are limited in the presence of HSF2, but exacerbated by its absence, in contrast to what happens in the fetal cortex upon FAE.

Figure S11. (A) Neuronal positioning defects induced by ETOH are less pronounced in *Hsf2*$^{-/-}$ (KO) compared with *Hsf2*$^{+/+}$ (WT) fetuses (related to Fig 6). Non-normalized (raw) data of the number of BrdU-labelled cells in fetal cortices from pregnant dams, non-exposed (CTR) or chronically intoxicated with food containing EtOH *ad libitum* (CAI) per 0.05 mm^2. Superficial layers, I-III; deep layers, IV-VI (n = 3 litters per condition). P-values for *Hsf2*$^{+/+}$: layers I-III, p = 0.0281; layers IV-VI, p = 0.0061.

(B) Quantitative RT-PCR analysis of mRNAs for *Dclk2, Nde1, Ndel1, NudC, Lis1, p39, Cdk5rap2, Rttn, Dis1, Dync1h1, Tubb2b*. Ratio of levels in chronically intoxicated (CAI) versus control embryonic cortices (CTR) A cut-off at 20% is indicated by dashed lines (see below). The graph represents the mean value of the ratio between each sample value and each control value. P values were calculated by comparing ratios obtained for the control (CTR) condition versus the CAI one. For *Dclk2*, *Nde1*, *Ndel1*, *Lis1*, *p39*, *Cdk5rap2*, n=5 cortices from individual embryos WT CTR; n=7 KO CTR, n=6 WT CAI; n=4 KO CAI. For *Disc1*, *Dyn*, *NudC*, *Tubb2*, n=3 for each conditions for the following genes.

For P-values, see Table S1. *** p< 0.0001; ** p< 0.001; * p<0.01. A ratio of 1 is indicated by the black line. P-values (see Table S1 for details): Same colour code as in

Fig 1C and D, 2A, and 6B. Note that D*clK2* and *Rttn* display an intermediate behaviour, compared to affected genes (Fig 6C) and non-affected genes: the ratio CAI/CTR is slightly below 20% for *Dclk2* and *Rttn*, but, respectively, only slightly or not dependent on HSF2. Data information: Differences were considered statistically significant when *P* values were <0.05, using unpaired two-way Student's *t*-test. Data are presented as mean ± s.e.m.

Table S1. Mean, s.e.m and p-values for RT-qPCR experiments in Figure 6 and Supplemental Figure S11 B. Data information: Differences were considered statistically significant when *P* values were <0.05, using unpaired two-way Student's *t*-test. Data are presented as mean ± s.e.m.

El Fatimy Supporting Information Figure S1

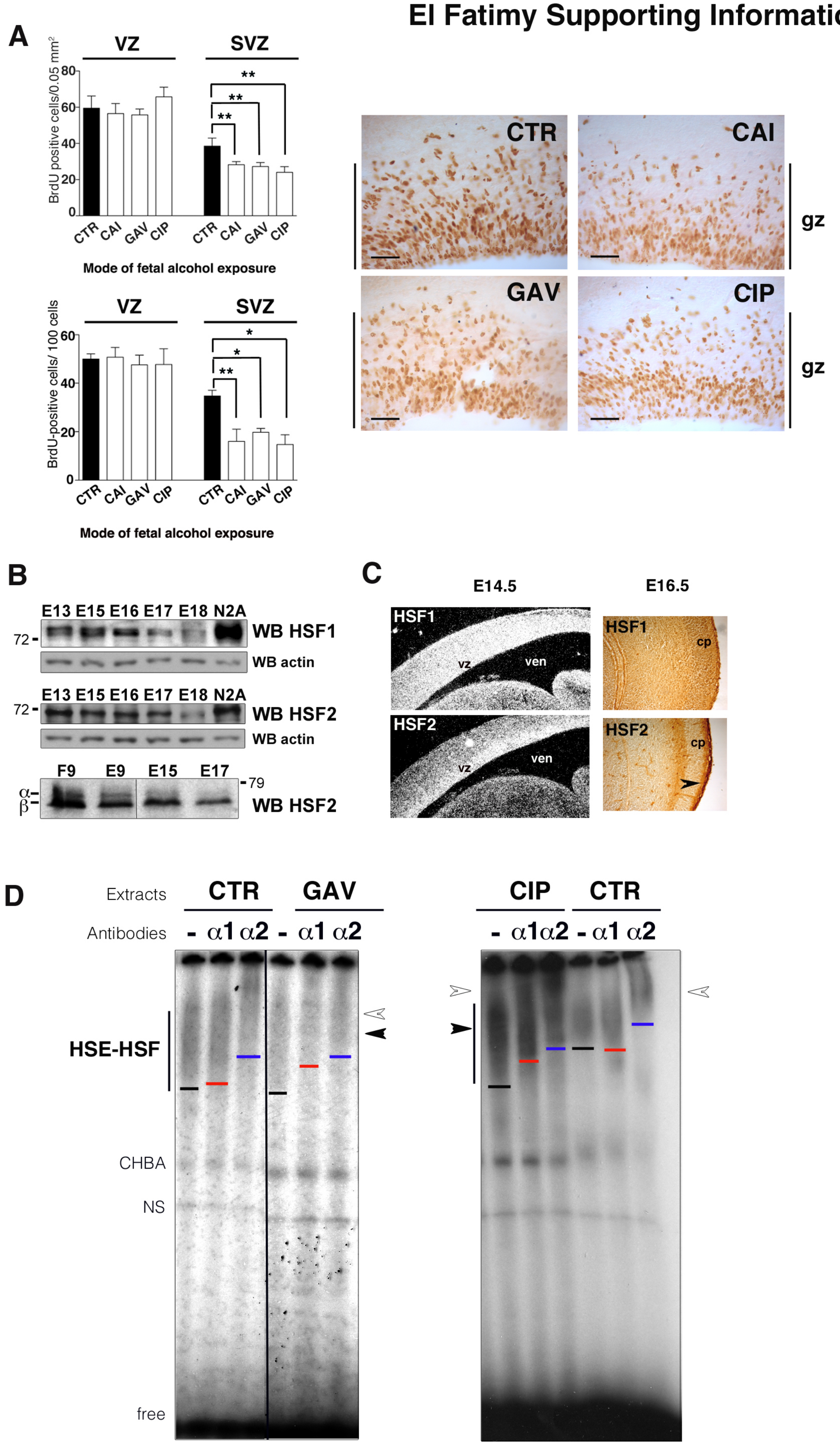

El Fatimy Supporting Information Figure S2

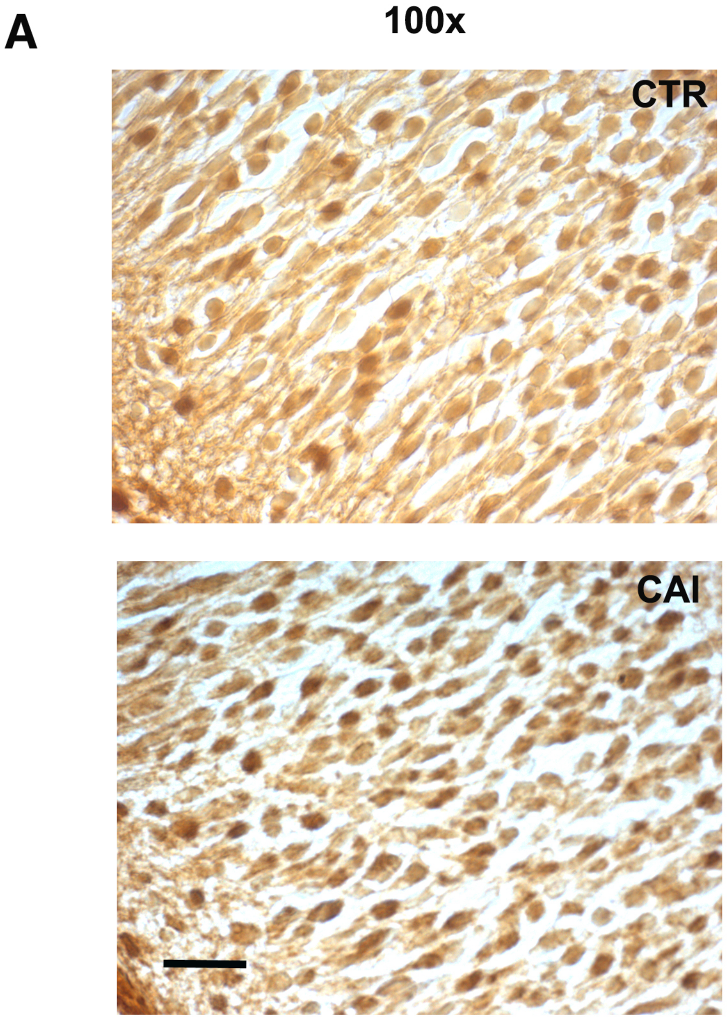

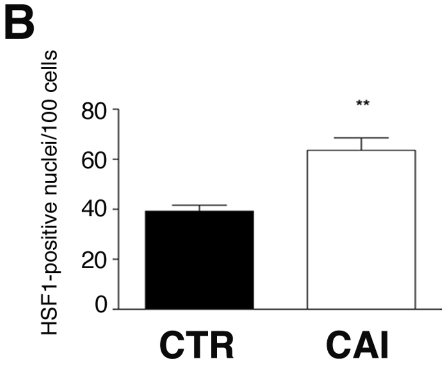

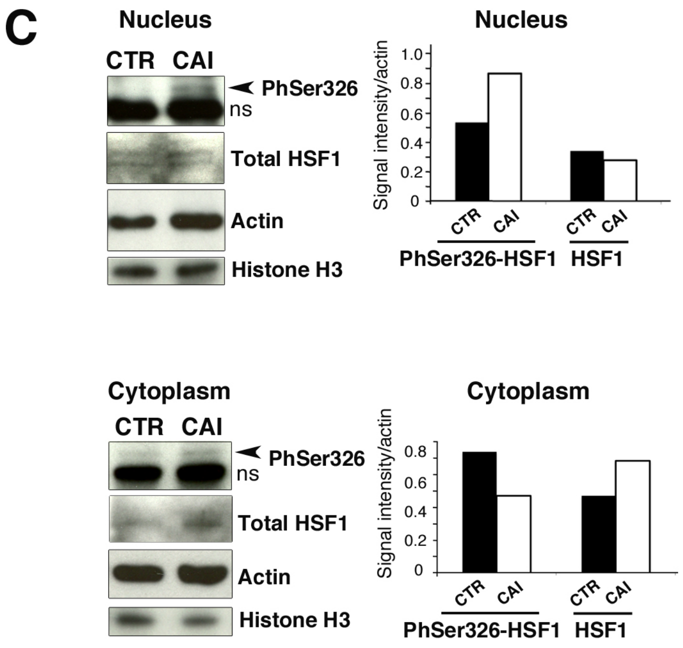

El Fatimy Supporting Information Figure S3

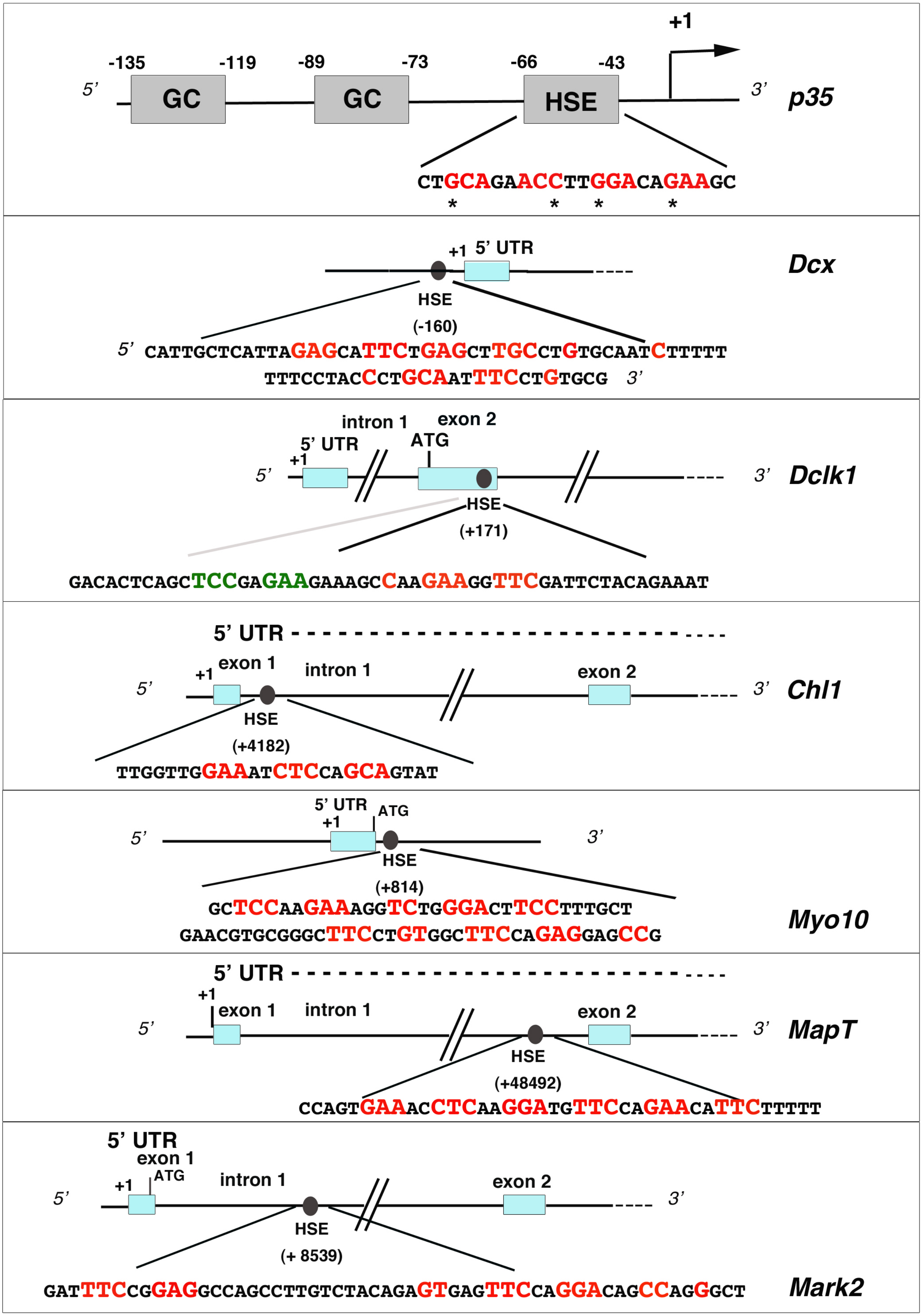

El Fatimy Supporting Information Figure S4

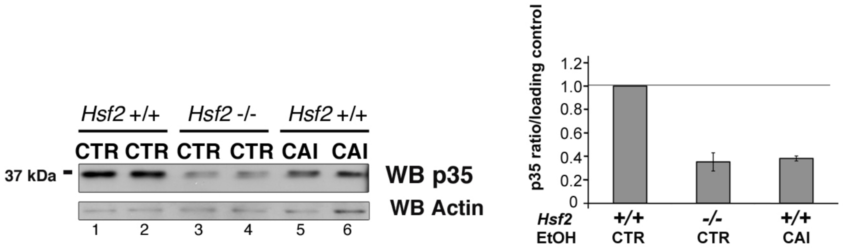

El Fatimy **Supporting Information** Figure S5

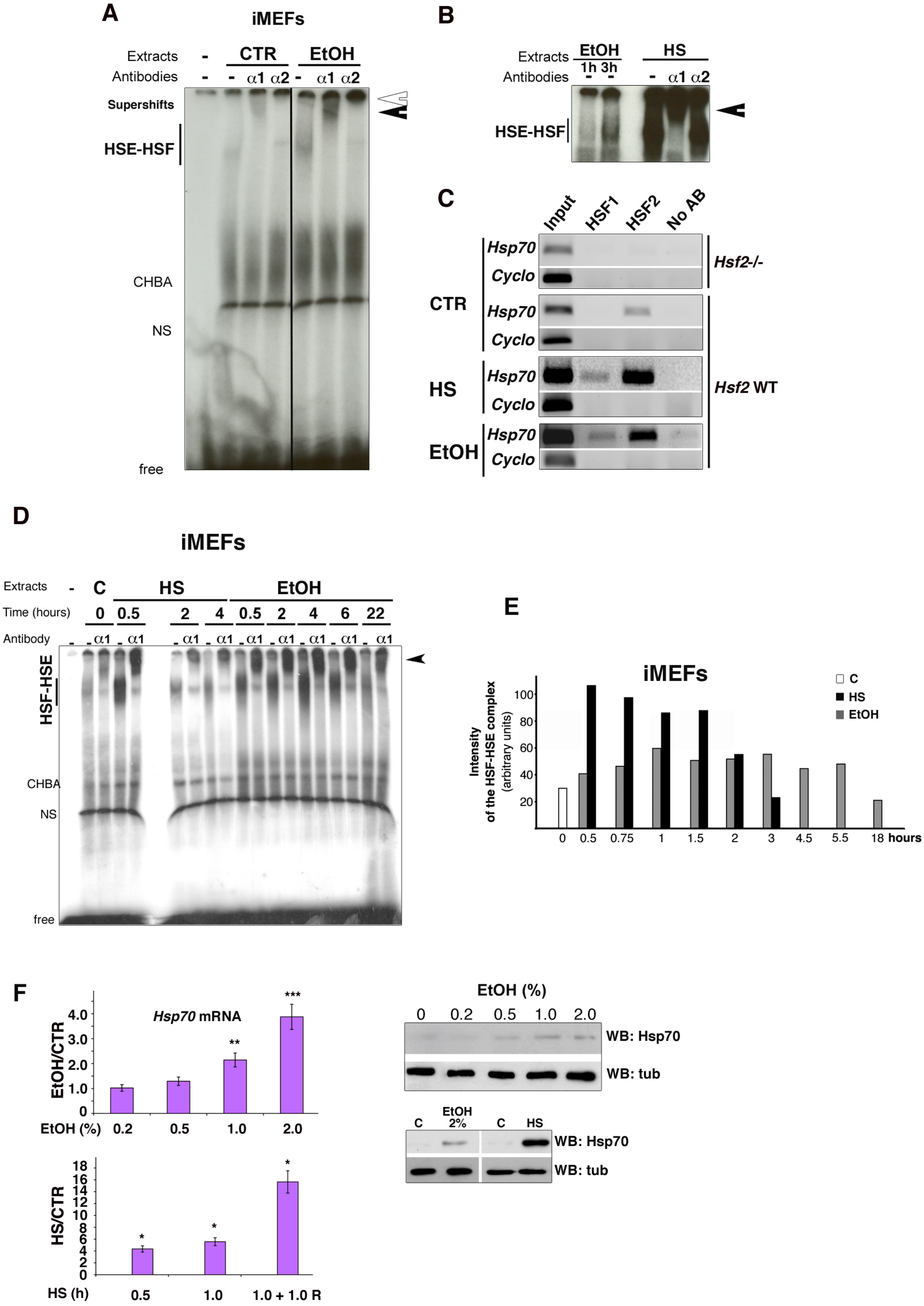

El Fatimy Supporting Information Figure S6

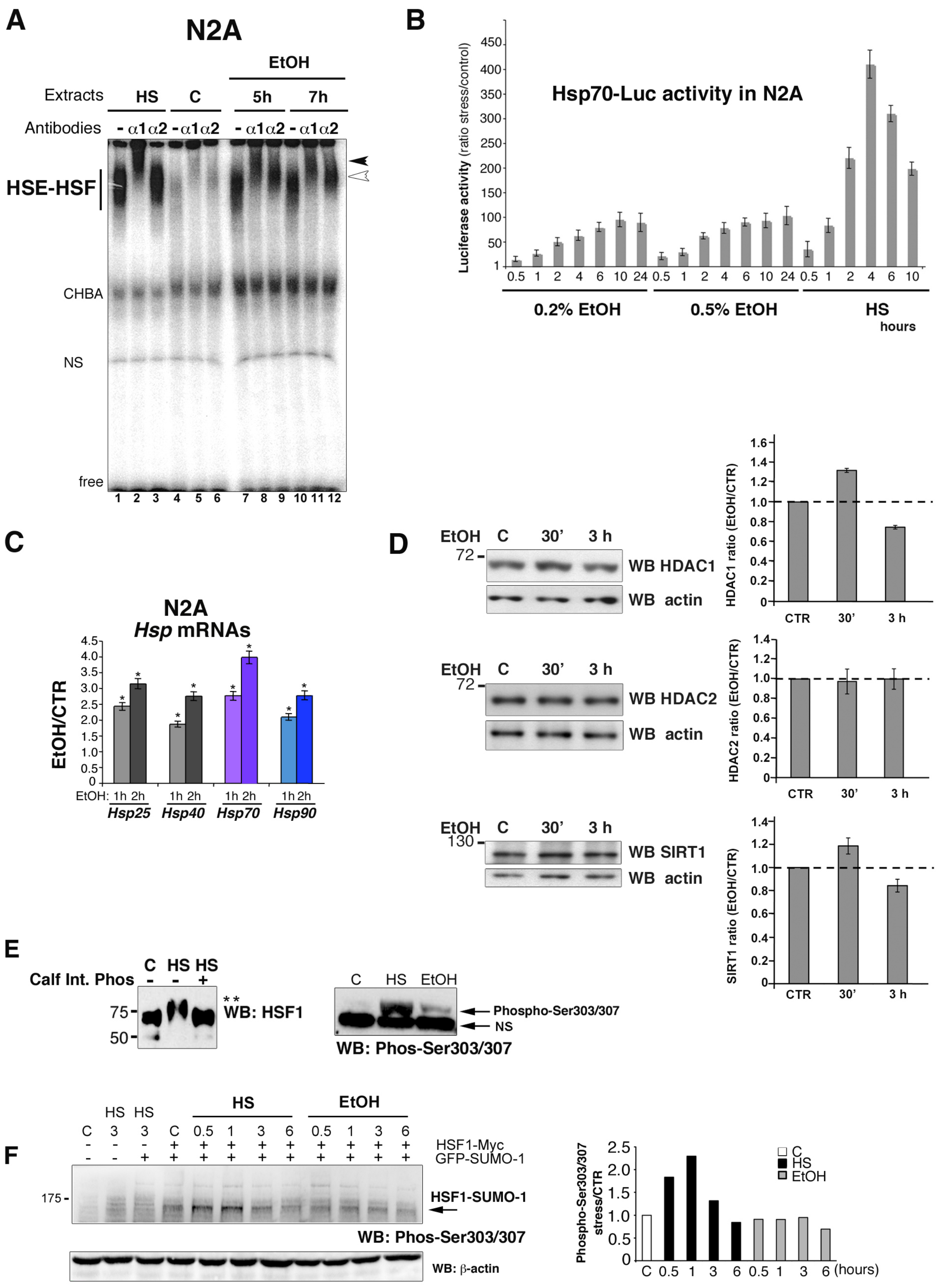

El Fatimy Supporting Information Figure S7

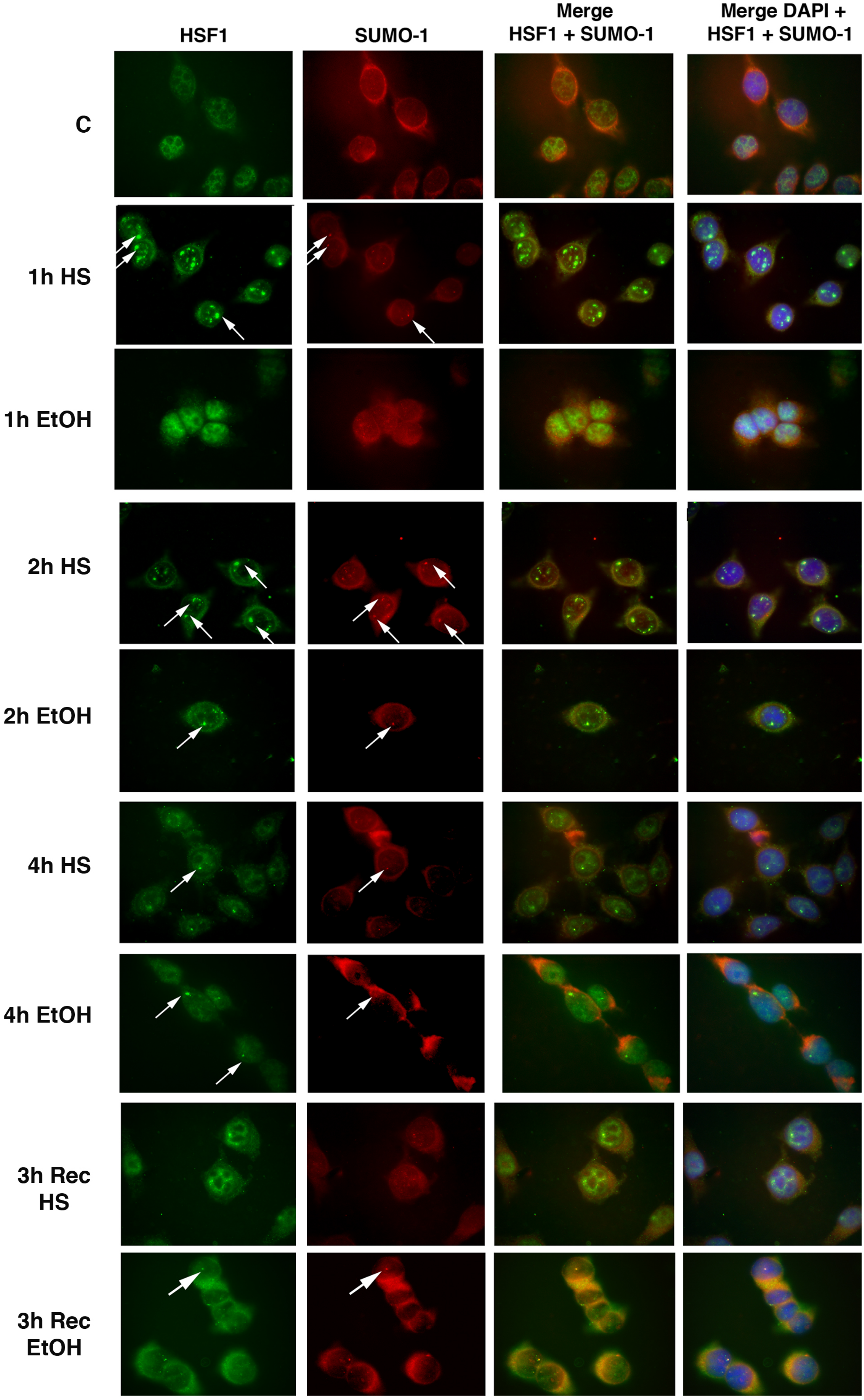

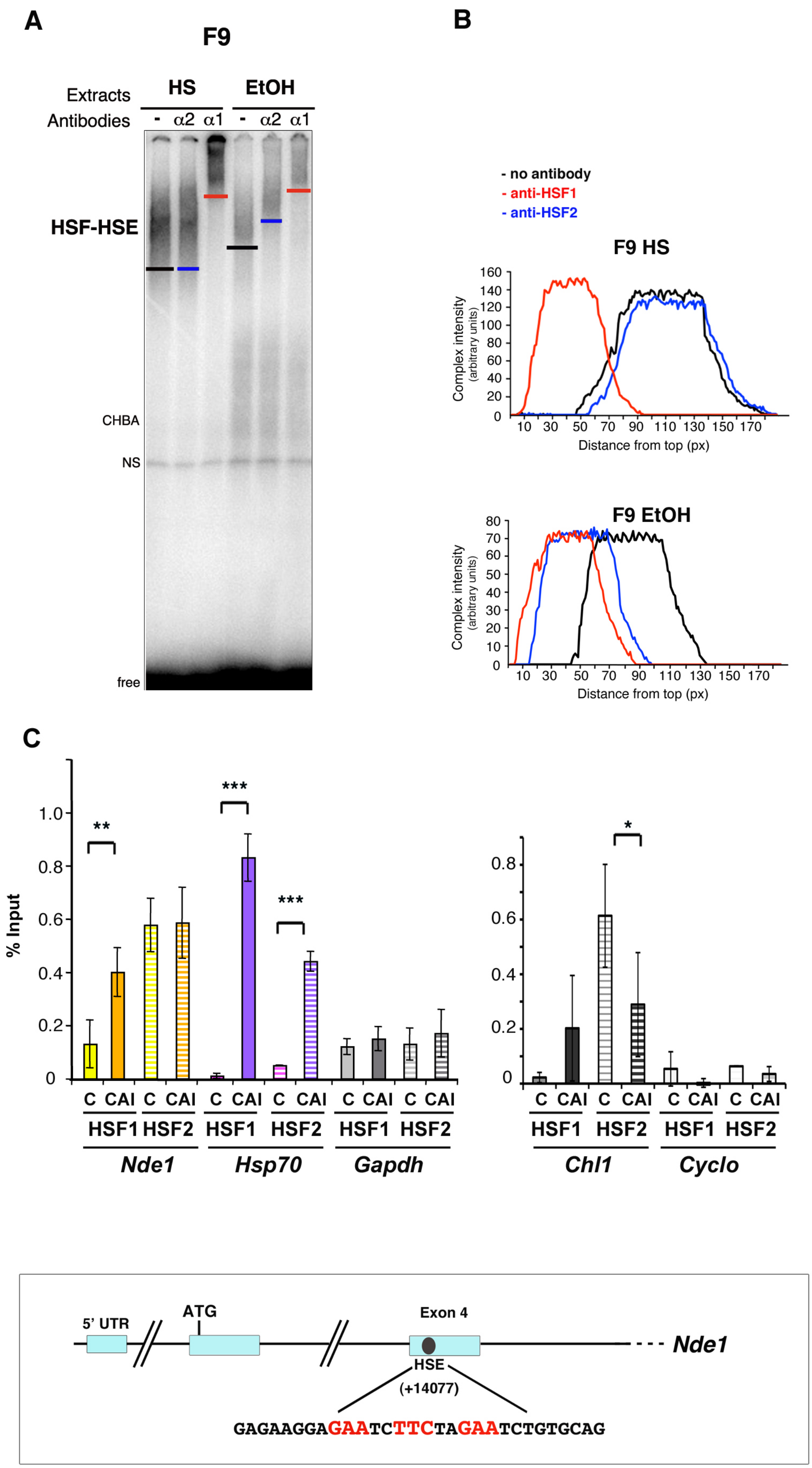
A
F9
Extracts
HS
EtOH
Antibodies
- α2 α1
- α2 α1
HSF-HSE
CHBA
NS
free
B
- no antibody
- anti-HSF1
- anti-HSF2
F9 HS
Complex intensity (arbitrary units)
Distance from top (px)
F9 EtOH
C
% Input
**
***
***
*
C CAI C CAI C CAI C CAI C CAI C CAI
HSF1 HSF2 HSF1 HSF2 HSF1 HSF2
Nde1
Hsp70
Gapdh
C CAI C CAI C CAI C CAI
HSF1 HSF2 HSF1 HSF2
Chl1
Cyclo
5' UTR
ATG
Exon 4
Nde1
HSE
(+14077)
GAGAAGGAGAATCTTCTAGAATCTGTGCAG

El Fatimy Supporting Information Figure S9

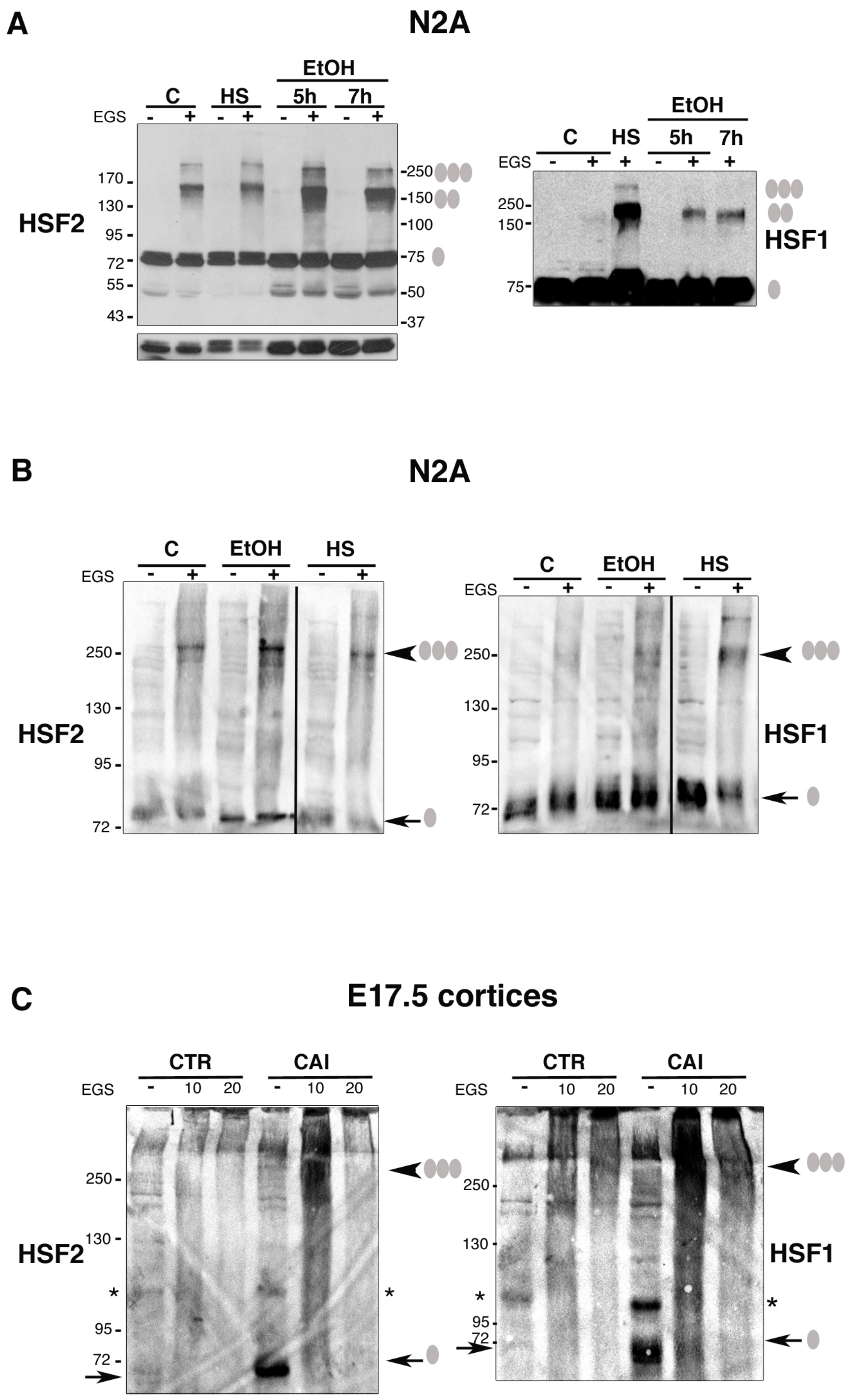

El Fatimy Supporting Information Figure S10

A

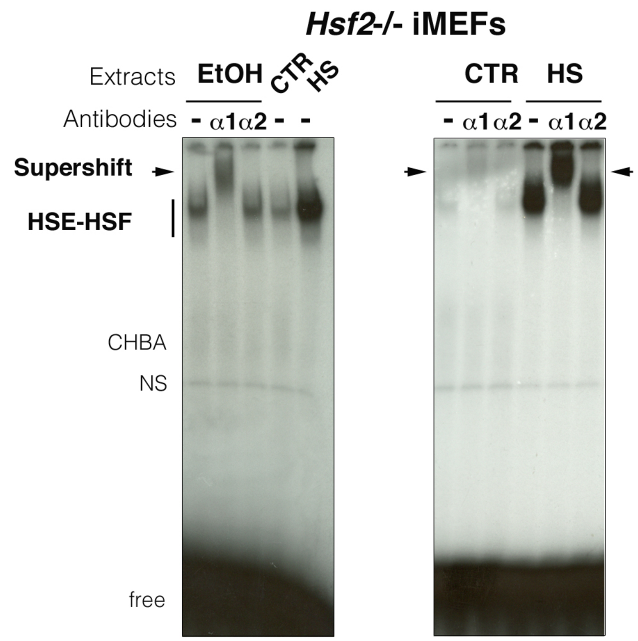

B

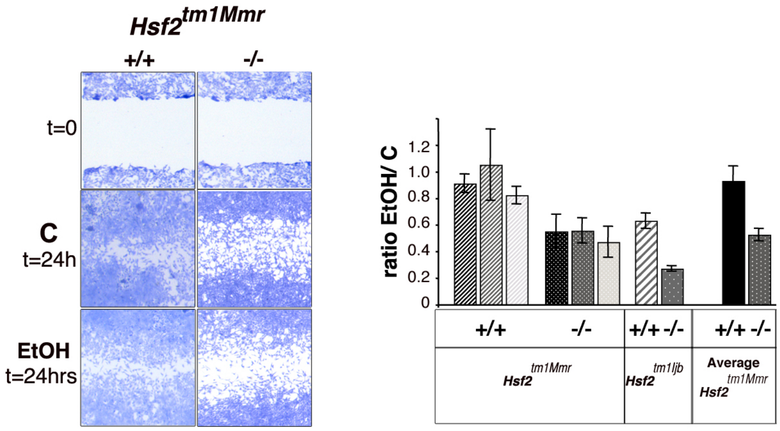

C

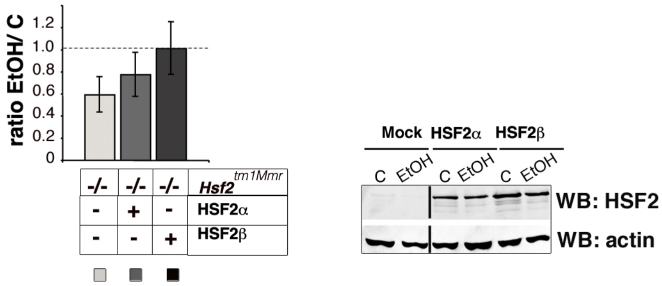

D

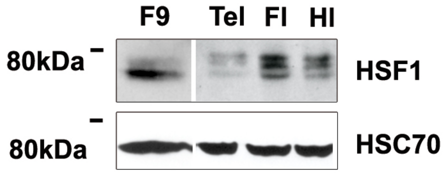

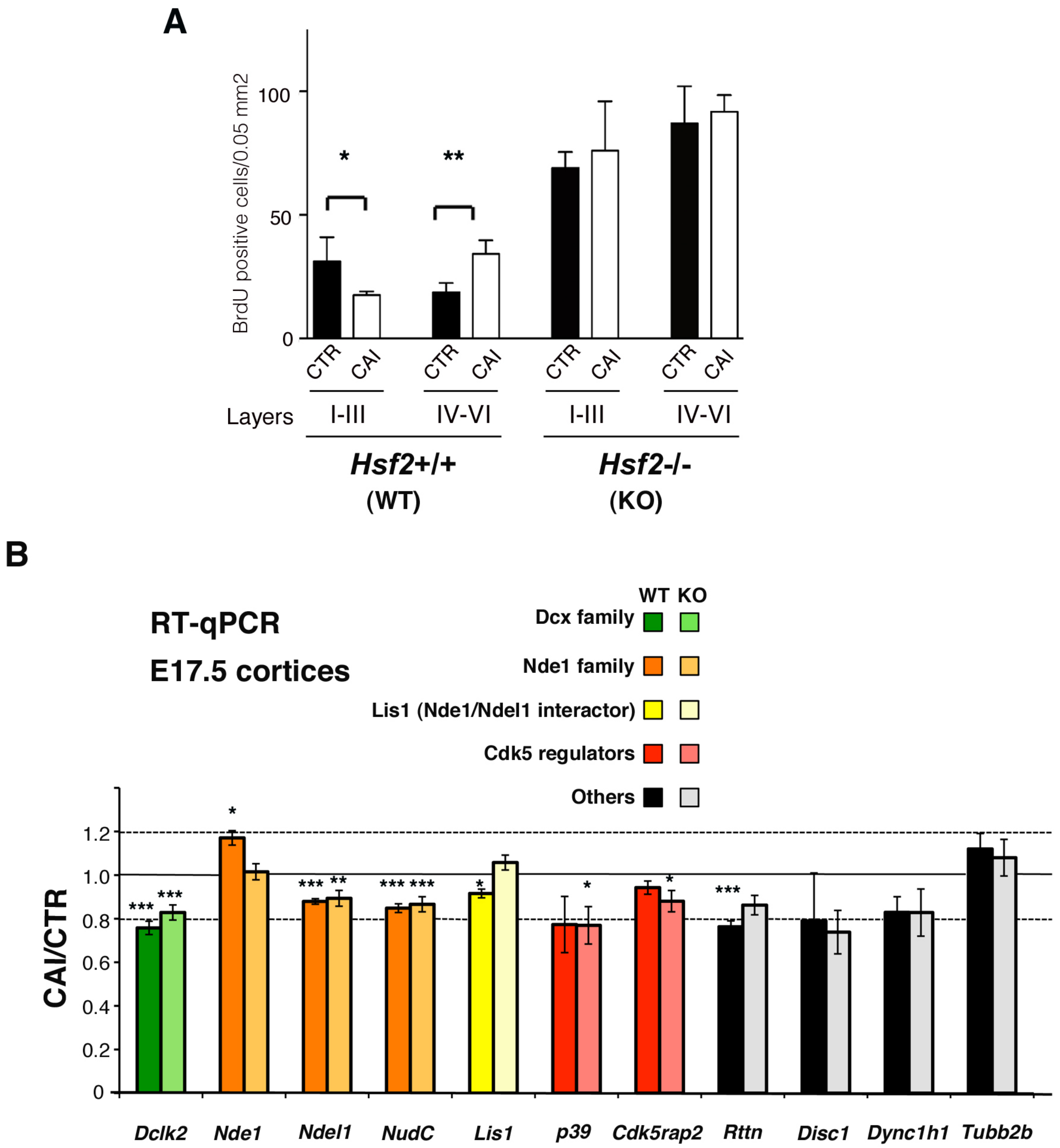
A
BrdU positive cells/0.05 mm2
100
50
0
*
**
CTR
CAI
Layers
I-III
IV-VI
Hsf2+/+
(WT)
Hsf2-/-
(KO)
B
RT-qPCR
E17.5 cortices
WT KO
Dcx family
Nde1 family
Lis1 (Nde1/Ndel1 interactor)
Cdk5 regulators
Others
CAI/CTR
1.2
1.0
0.8
0.6
0.4
0.2
0
Dclk2
Nde1
Ndel1
NudC
Lis1
p39
Cdk5rap2
Rttn
Disc1
Dync1h1
Tubb2b

Table S1. Mean, sem and p-values for RT-qPCR experiments in Figure 6 and S11

Genes families	Ratio CAI/CTR			p value CAI/CTR vs CTR/CTR	Ratio CTR/CTR							
	SEM	CAI/CTR	Mean of ratio CAI/CTR		SEM	CTR/CTR	Mean of ratio CTR/CTR	WT CTR	WT CAI	KO CTR	KO CAI	
Doublcortin family		dclk1				dclk1						
	0,0209	WTA/WTC	0,6261	1,20E-12	0,0404	WTC/WTC	1,0189	n=5	n=6	n=7	n=4	
	0,0432	KOA/KOC	0,7879	1,149E-05	0,0303	KOC/KOC	1,0212					
		dclk2				dclk2						
	0,0305	WTA/WTC	0,7578	6,789E-07	0,0367	WTC/WTC	1,0156	n=5	n=6	n=7	n=4	
	0,0347	KOA/KOC	0,8285	2,72E-06	0,0197	KOC/KOC	1,0091					
		dcx				dcx						
	0,0398	WTA/WTC	0,7474	5,127E-05	0,0593	WTC/WTC	1,0393	n=5	n=6	n=7	n=4	Affected genes
	0,0299	KOA/KOC	0,9926	0,3121065	0,0206	KOC/KOC	1,0100					
Nde1 family and interactor		nde1				nde1						
	0,0332	WTA/WTC	1,1707	0,0022292	0,0397	WTC/WTC	1,0183	n=5	n=6	n=7	n=4	
	0,0370	KOA/KOC	1,0154	0,4769253	0,0237	KOC/KOC	1,0129					
		NudeL				NudeL						
	0,0126	WTA/WTC	0,8791	5,853E-09	0,0130	WTC/WTC	1,0020	n=5	n=6	n=7	n=4	
	0,0363	KOA/KOC	0,8936	0,0003682	0,0112	KOC/KOC	1,0030					
		Lis1				Lis1						
	0,0199	WTA/WTC	0,9158	0,0014645	0,0202	WTC/WTC	1,0049	n=5	n=6	n=7	n=4	
	0,0344	KOA/KOC	1,0576	0,0612531	0,0156	KOC/KOC	1,0058					
		NudC				NudC						
	0,0200	WTA/WTC	0,8484	2,685E-05	0,0197	WTC/WTC	1,0016	n=3	n=3	n=3	n=3	
	0,0348	KOA/KOC	0,8661	3,214E-08	0,0057	KOC/KOC	1,0001					
Cdk5 pathway		p35				p35						
	0,0303	WTA/WTC	0,7252	1,662E-06	0,0538	WTC/WTC	1,0327	n=5	n=6	n=7	n=4	
	0,0401	KOA/KOC	0,8776	0,003806	0,0339	KOC/KOC	1,0262					
		p39				p39						
	0,1290	WTA/WTC	0,7731	0,0125959	0,2413	WTC/WTC	1,3754	n=5	n=6	n=7	n=4	
	0,0860	KOA/KOC	0,7695	0,0033206	0,0871	KOC/KOC	1,1391					
		cdk5rap2				cdk5rap2						
	0,0304	WTA/WTC	0,9426	0,0605938	0,0305	WTC/WTC	1,0110	n=5	n=6	n=7	n=4	
	0,0489	KOA/KOC	0,8803	0,0031283	0,0212	KOC/KOC	1,0105					
Others		Chl1				Chl1						
	0,0163	WTA/WTC	0,7461	4,042E-15	0,0178	WTC/WTC	1,0038	n=5	n=6	n=7	n=4	
	0,0304	KOA/KOC	0,8805	2,887E-05	0,0135	KOC/KOC	1,0043					
		Myo10				Myo10						
	0,0574	WTA/WTC	0,7250	0,0053415	0,0885	WTC/WTC	1,0298	n=3	n=3	n=3	n=3	
	0,1170	KOA/KOC	1,1956	0,0676838	0,0332	KOC/KOC	1,0044					
		MapT				MapT						
	0,0586	WTA/WTC	0,5618	7,774E-05	0,0727	WTC/WTC	1,0204	n=3	n=3	n=3	n=3	
	0,0497	KOA/KOC	0,7845	0,0001531	0,0441	KOC/KOC	1,0891					
		Mark2				Mark2						
	0,0648	WTA/WTC	0,6743	0,0015859	0,0768	WTC/WTC	1,0227	n=3	n=3	n=3	n=3	
	0,1291	KOA/KOC	0,9704	0,3071125	0,0936	KOC/KOC	1,0331					
		Rttn				Rttn						
	0,0265	WTA/WTC	0,7633	1,087E-05	0,0309	WTC/WTC	1,0038	n=3	n=3	n=3	n=3	
	0,0445	KOA/KOC	0,8624	0,0137827	0,0392	KOC/KOC	1,0061					
		Disc1				Disc1						
	0,2195	WTA/WTC	0,7901	0,0960448	0,5895	WTC/WTC	1,6468	n=3	n=3	n=3	n=3	
	0,0995	KOA/KOC	0,7383	0,010179	0,0251	KOC/KOC	1,0025					
		Dyn				Dyn						
	0,0718	WTA/WTC	0,8293	0,1981563	0,0449	WTC/WTC	1,0080	n=3	n=3	n=3	n=3	
	0,1082	KOA/KOC	0,8283	0,0693941	0,0324	KOC/KOC	1,0042					
		Tubb2b				Tubb2b						
	0,0712	WTA/WTC	1,1199	0,2217436	0,0892	WTC/WTC	1,0302	n=3	n=3	n=3	n=3	
	0,0842	KOA/KOC	1,0798	0,1856104	0,0159	KOC/KOC	1,0010					
