# Supplementary material for: Heat shock factor 2 is a stress-responsive mediator of neuronal migration defects in models of fetal alcohol syndrome
Source: EMBO Mol Med. 2014 Jul 15;6(8):1043–61. doi: 10.15252/emmm.201303311 (PMC4154132; doi:10.15252/emmm.201303311)

Source data Suppl. Figure S1 B El Fatimy et al.

Raw data WB Suppl. FigS1 B (upper and middle panel)

The membrane was cut

into 2 pieces,

incubated with either

anti-HSF or anti Actin antibodies

and reconstituted for exposure

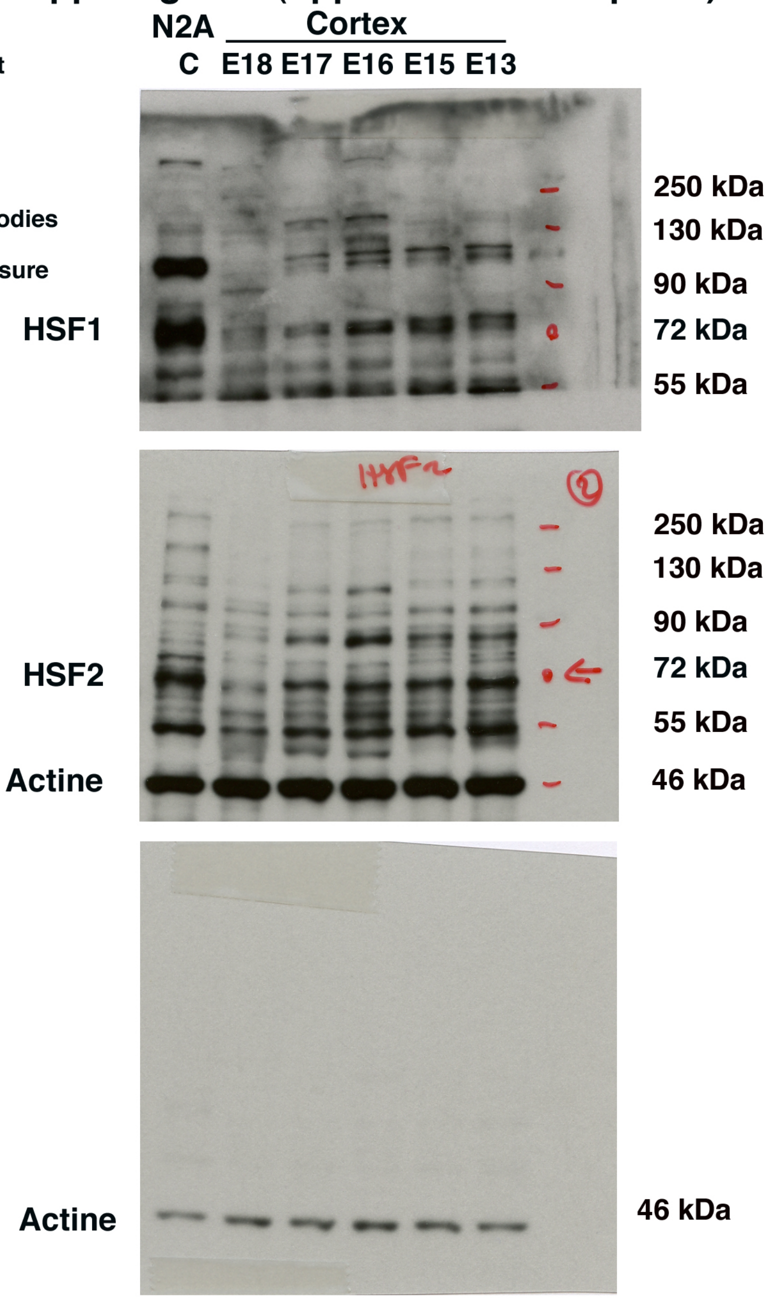

Raw data WB Suppl. FigS1 B lower panel (frame: lanes of interest)

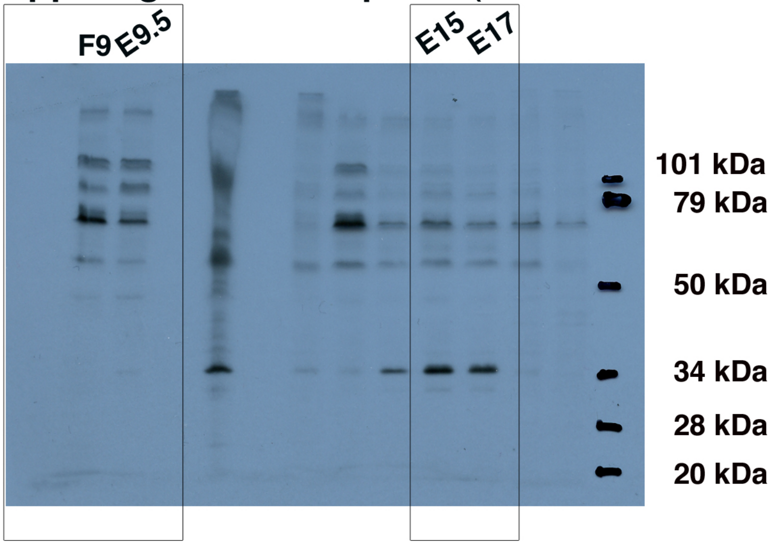

Source data Suppl. Figure S1D El Fatimy p.1

Raw data EMSA gel (within frame) (left panel)

CTR **GAV**

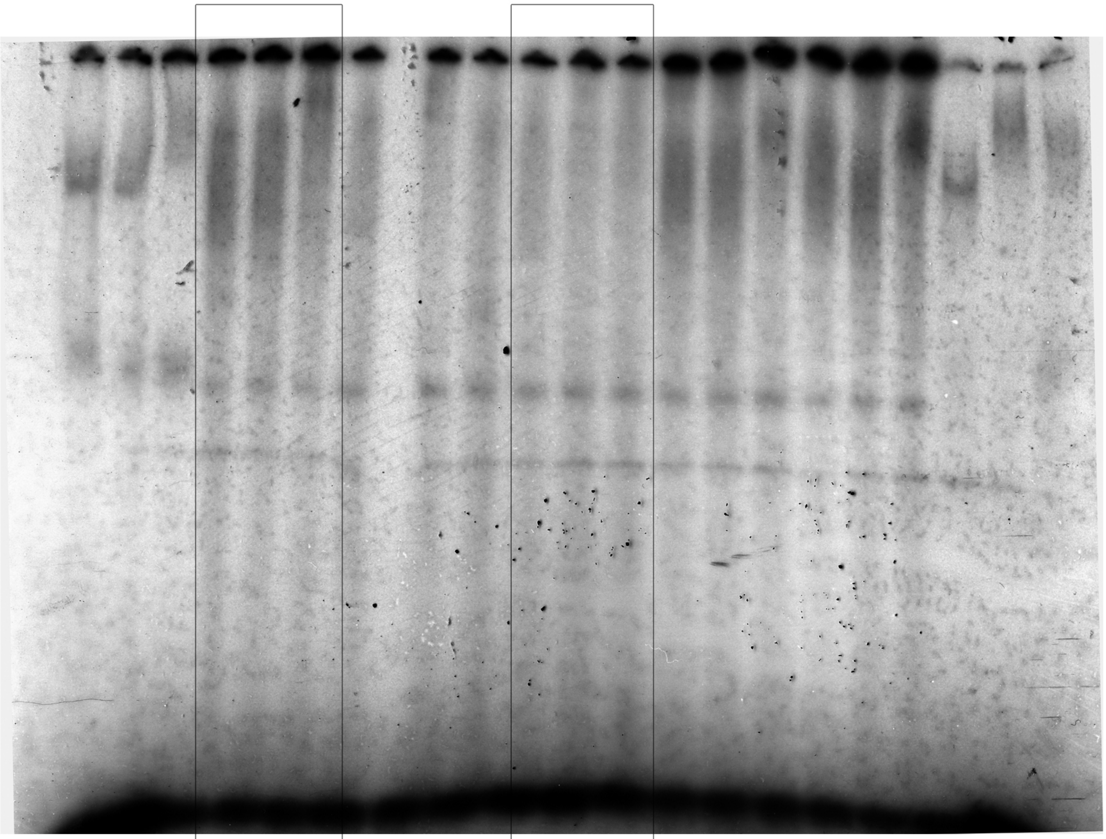

idem (with lane indications)

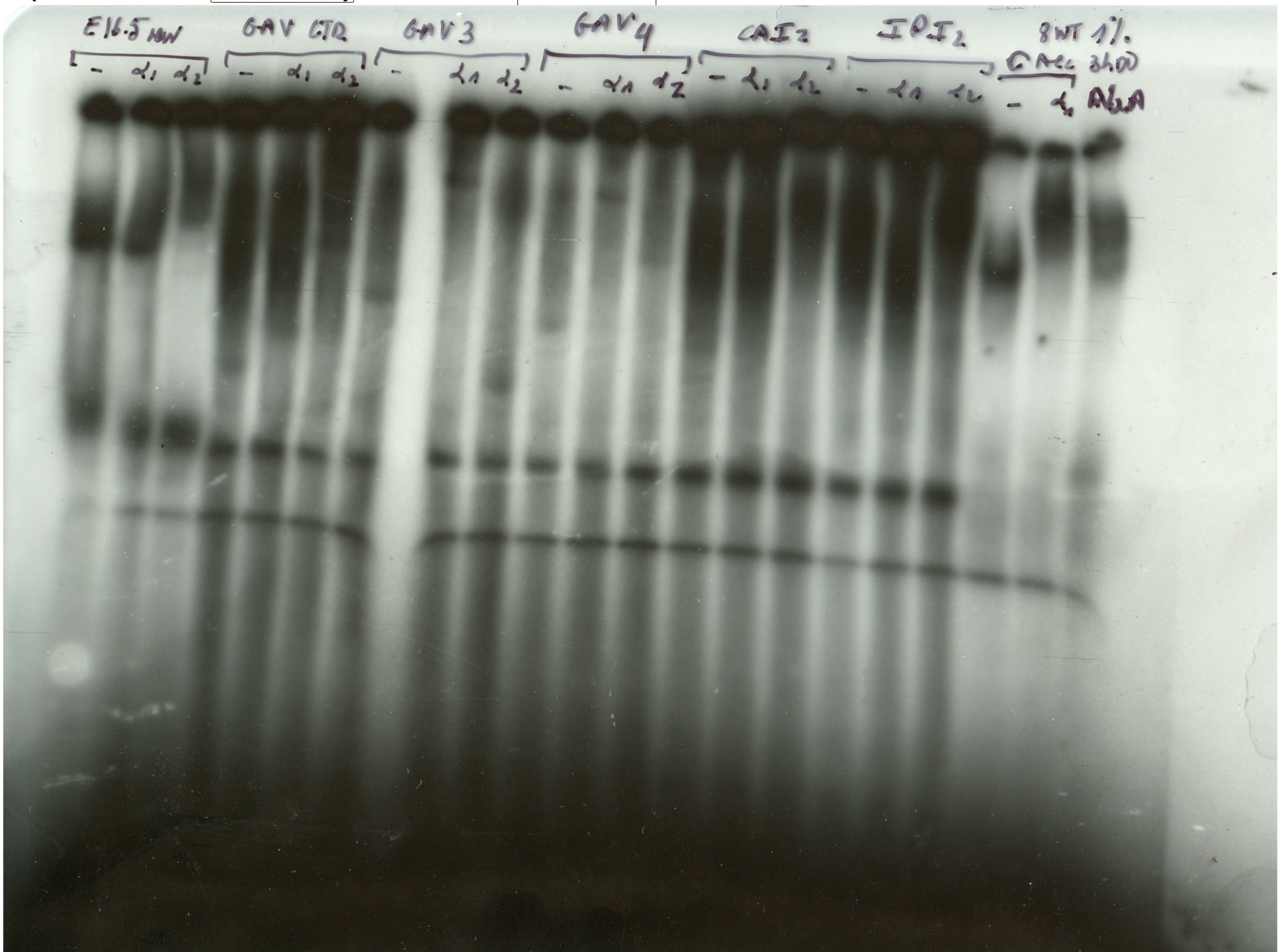

Source data Suppl. Figure S1D El Fatimy p2

Raw data EMSA gel Fig S1D (right panel) (frame indicates the relevant lanes)

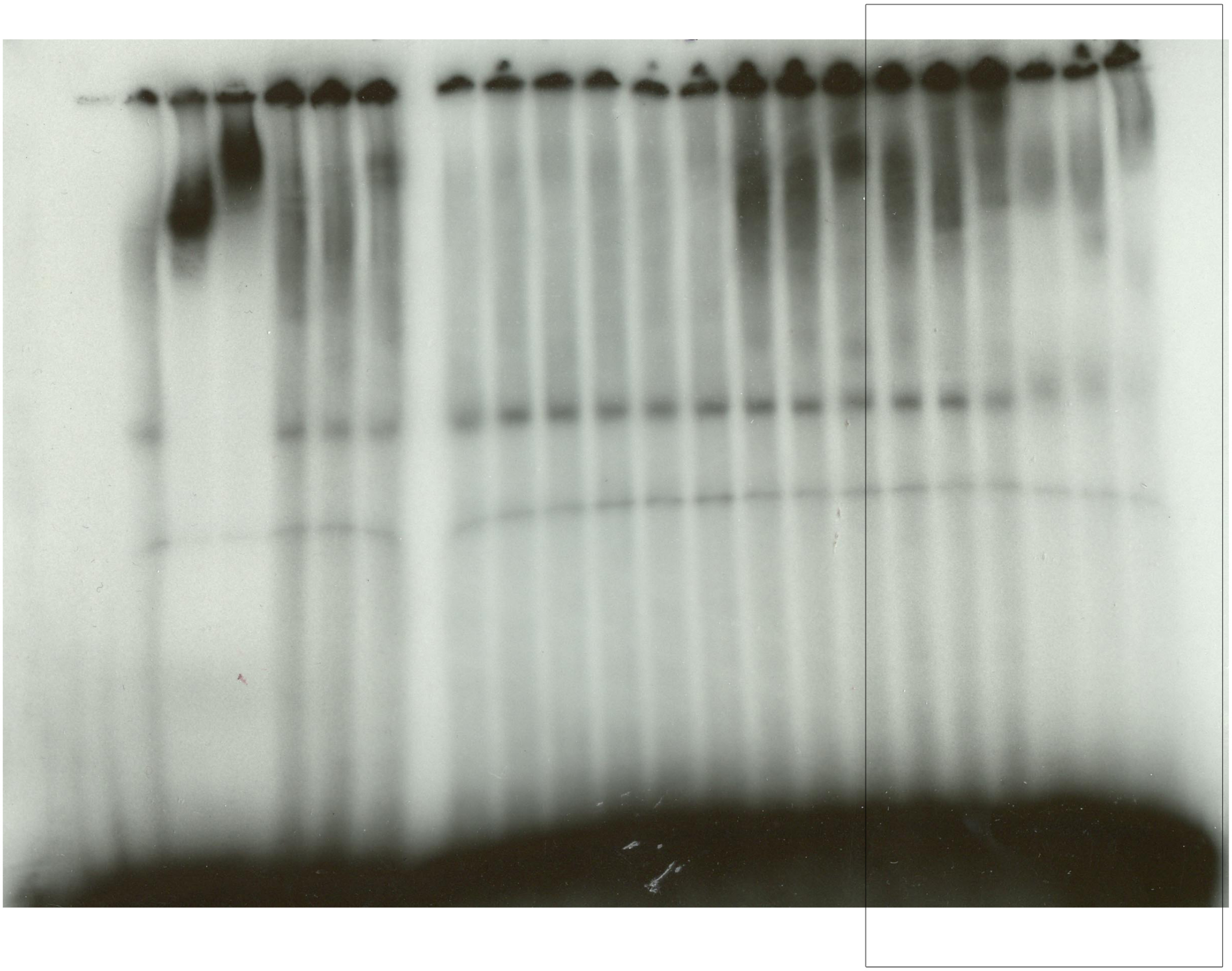

Supplement: Supplementary file 2 [file emmm0006-1043-sd2.pdf]
