# Supplementary material for: Heat shock factor 2 is a stress-responsive mediator of neuronal migration defects in models of fetal alcohol syndrome
Source: EMBO Mol Med. 2014 Jul 15;6(8):1043–61. doi: 10.15252/emmm.201303311 (PMC4154132; doi:10.15252/emmm.201303311)
Supplement: Supplementary file 3 [file emmm0006-1043-sd3.pdf]

Source data Suppl. Figure S2 C El Fatimy p.1

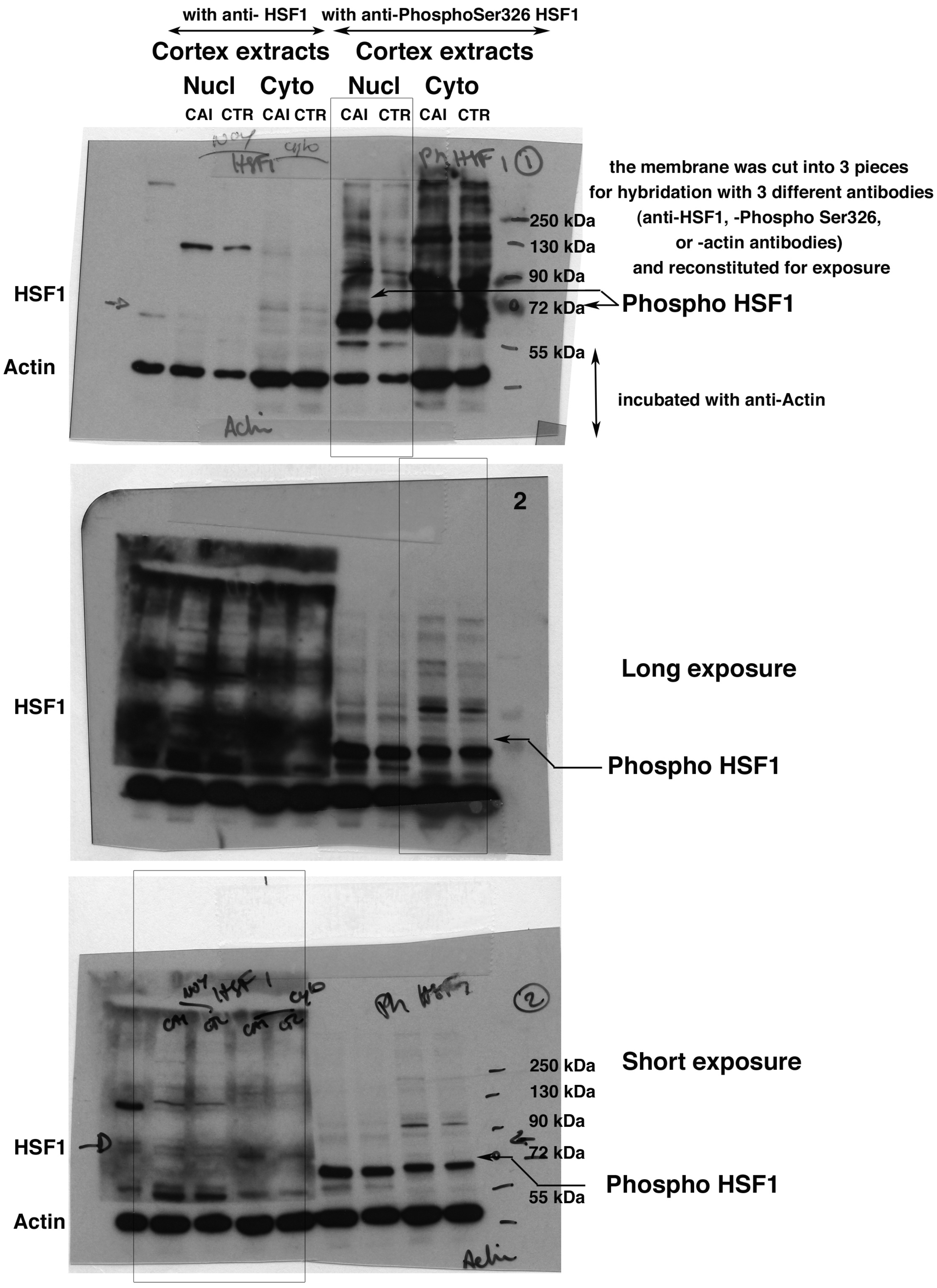

Source data Suppl. Figure S2 C El Fatimy p. 2

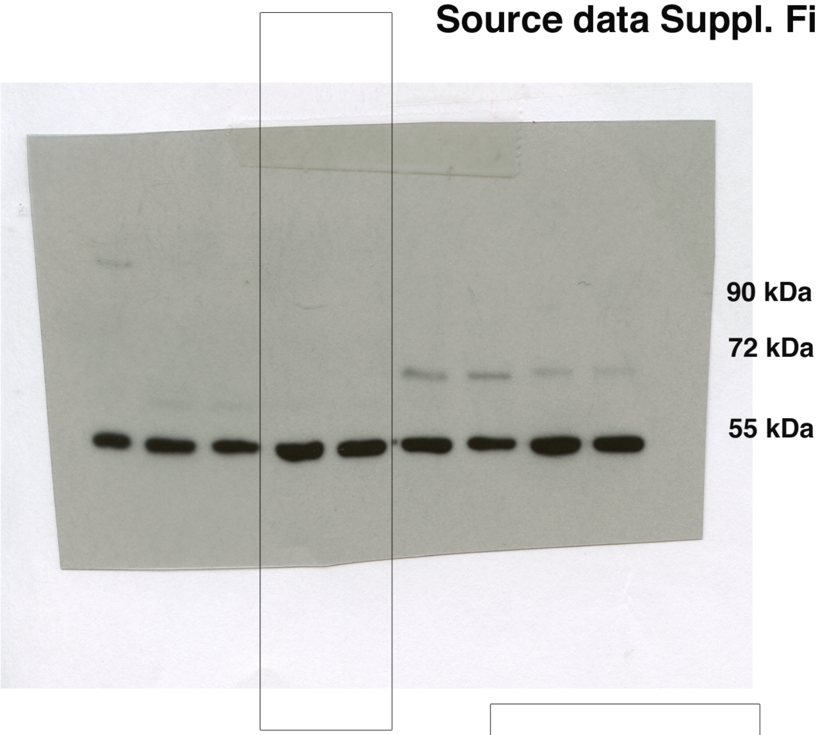

Actin
(lower exposure corresponding to the film named «2» on page 1)

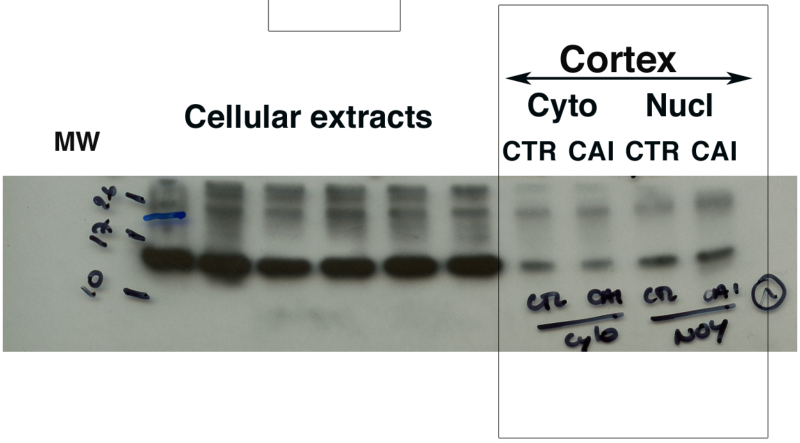

Histone H3
