# Supplementary material for: Heat shock factor 2 is a stress-responsive mediator of neuronal migration defects in models of fetal alcohol syndrome
Source: EMBO Mol Med. 2014 Jul 15;6(8):1043–61. doi: 10.15252/emmm.201303311 (PMC4154132; doi:10.15252/emmm.201303311)

Source data Suppl. Figure S4 El Fatimy

Raw data for WB p35 (upper panel)

+/+ CTR | +/+ CTR | -/- CTR | -/- CTR | +/+ CAI | +/+ CAI

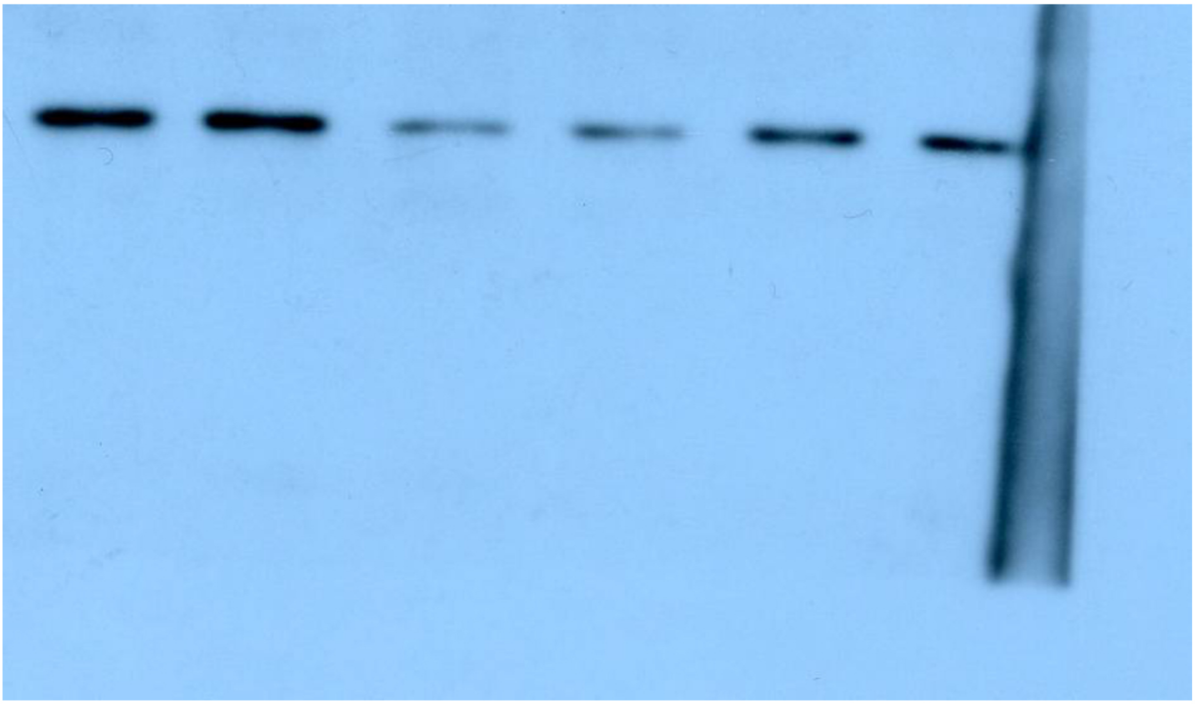

with MW (indicated on paper print)

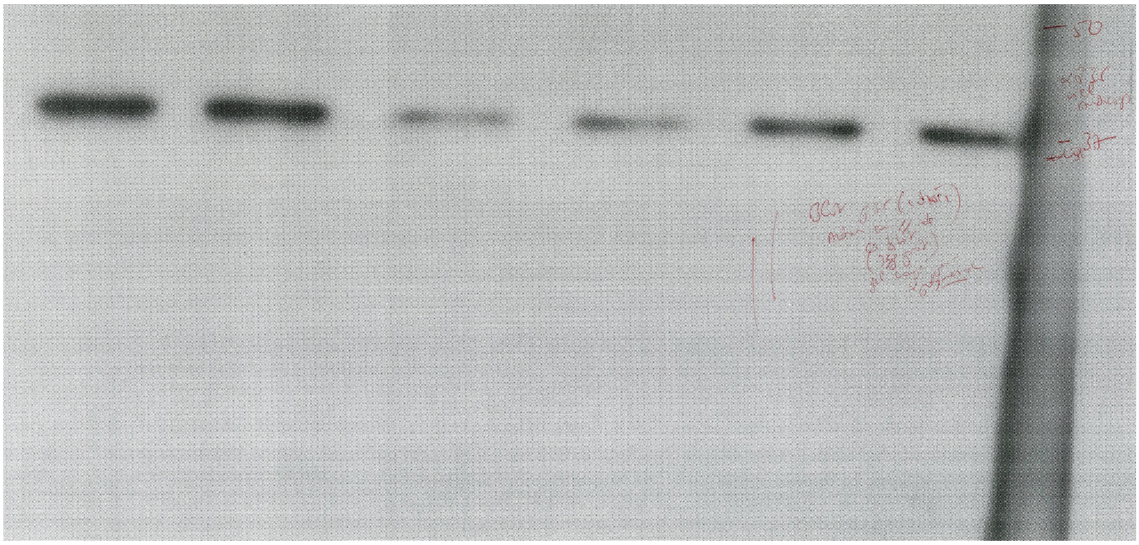

- 50 kDa
- 37 kDa

Raw data for loading control Actin (lower panel)

+/+ CTR | +/+ CTR | -/- CTR | -/- CTR | +/+ CAI | +/+ CAI

50

37

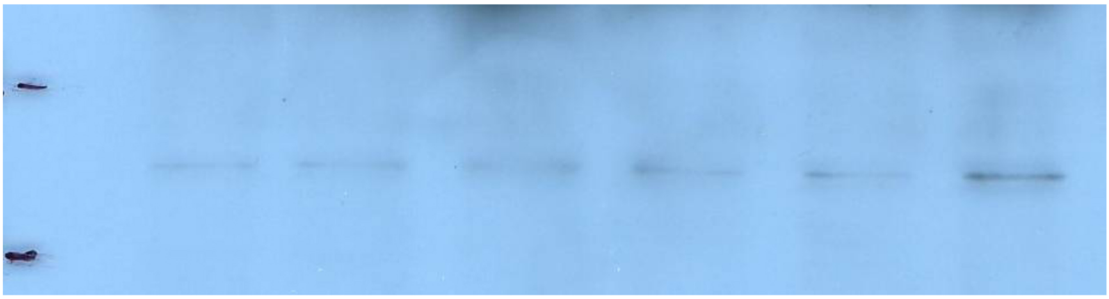

Supplement: Supplementary file 4 [file emmm0006-1043-sd4.pdf]
