# Supplementary material for: Heat shock factor 2 is a stress-responsive mediator of neuronal migration defects in models of fetal alcohol syndrome
Source: EMBO Mol Med. 2014 Jul 15;6(8):1043–61. doi: 10.15252/emmm.201303311 (PMC4154132; doi:10.15252/emmm.201303311)

Source data Suppl. Figure S5 El Fatimy

Raw data EMSA Suppl. Fig 5A (left panel)

lanes of interest within frame

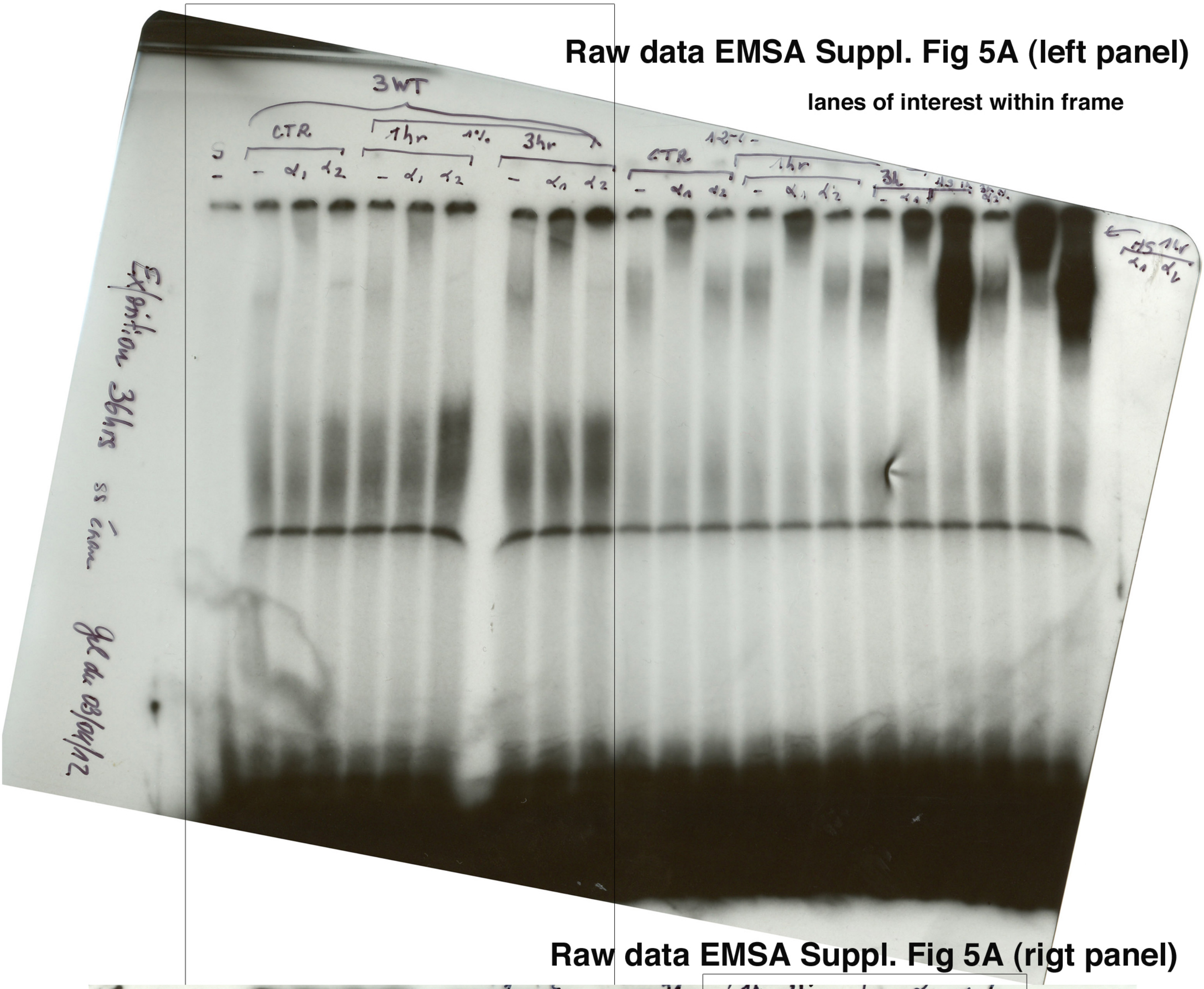

Raw data EMSA Suppl. Fig 5A (rigt panel)

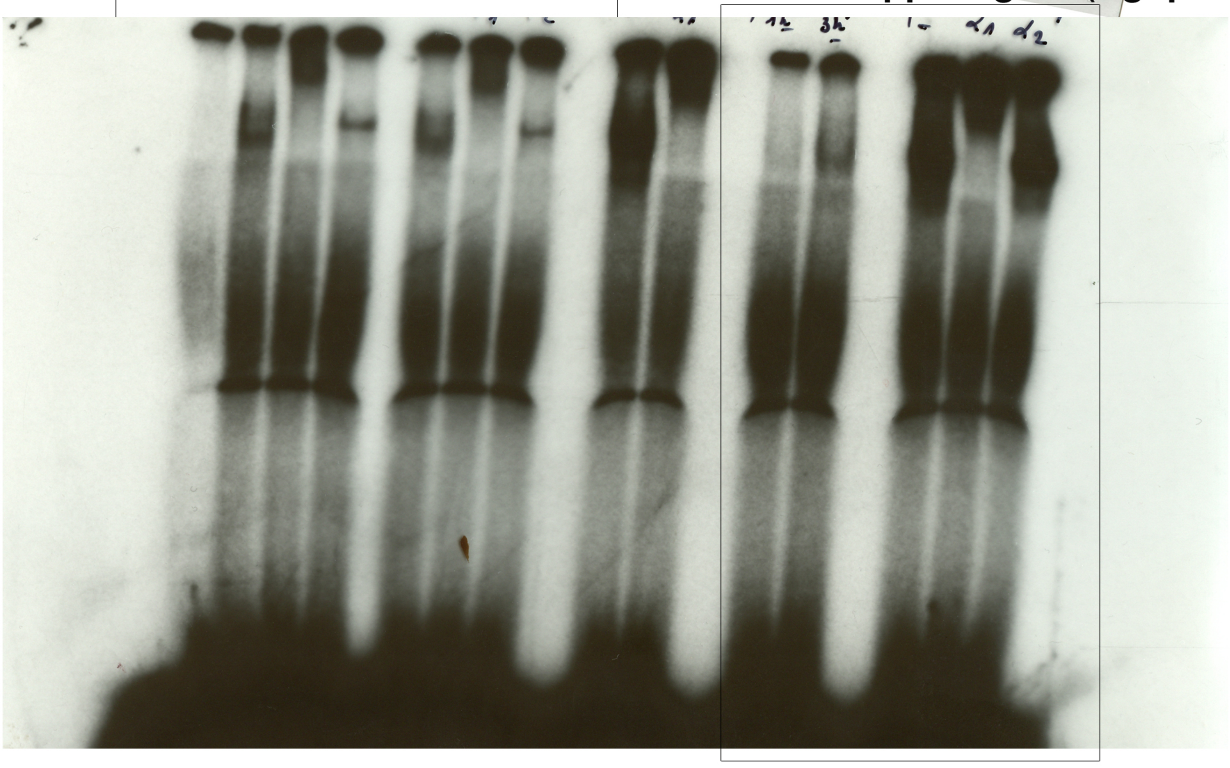

Source data Suppl. FigS5 C El Fatimy et al. p.1

Raw data ChIP iMEF Hsf2 -/- CTRL

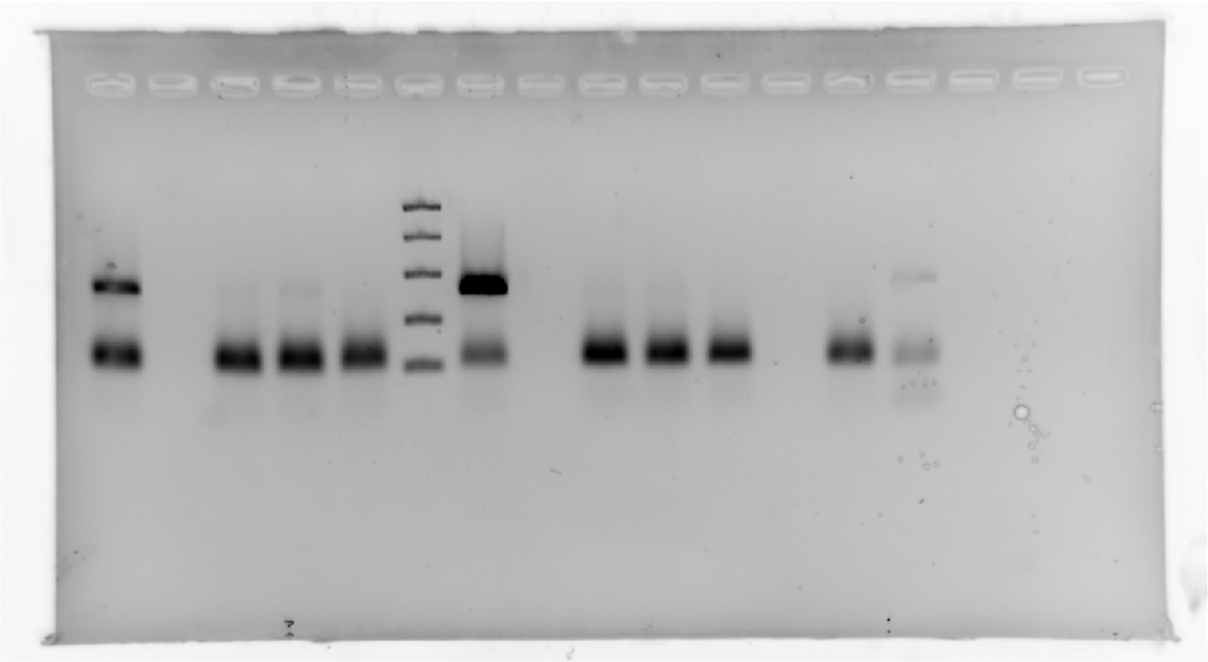

Raw data ChIP iMEF Hsf2 WT CTRL & HS

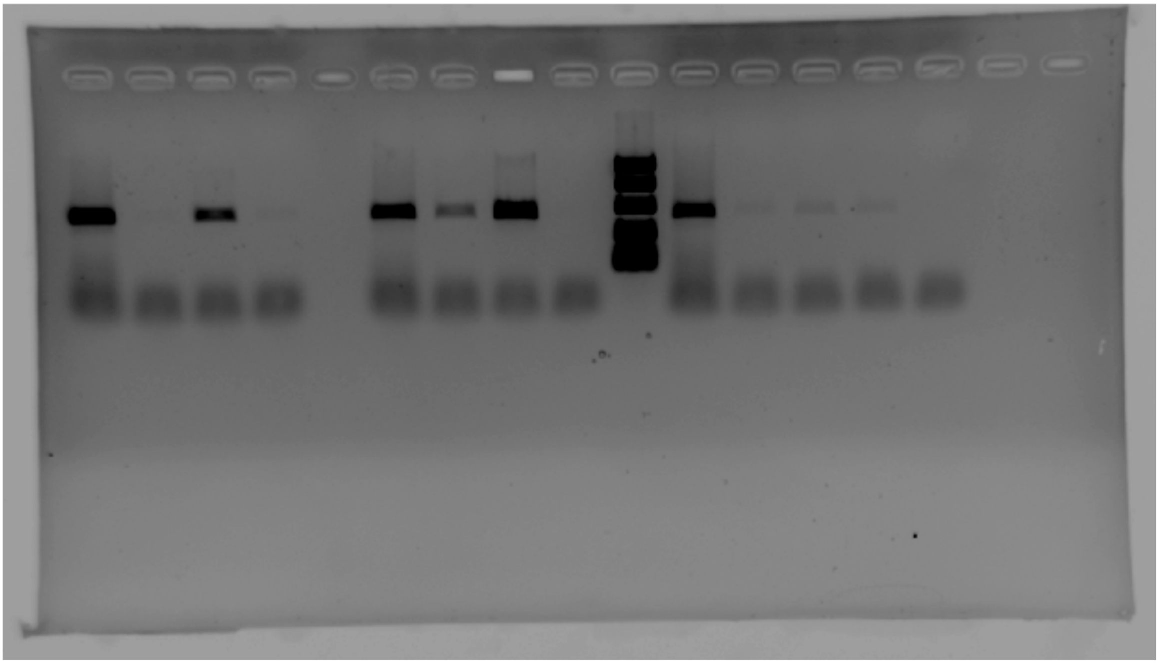

Raw data ChIP iMEF Hsf2 WT EtOH

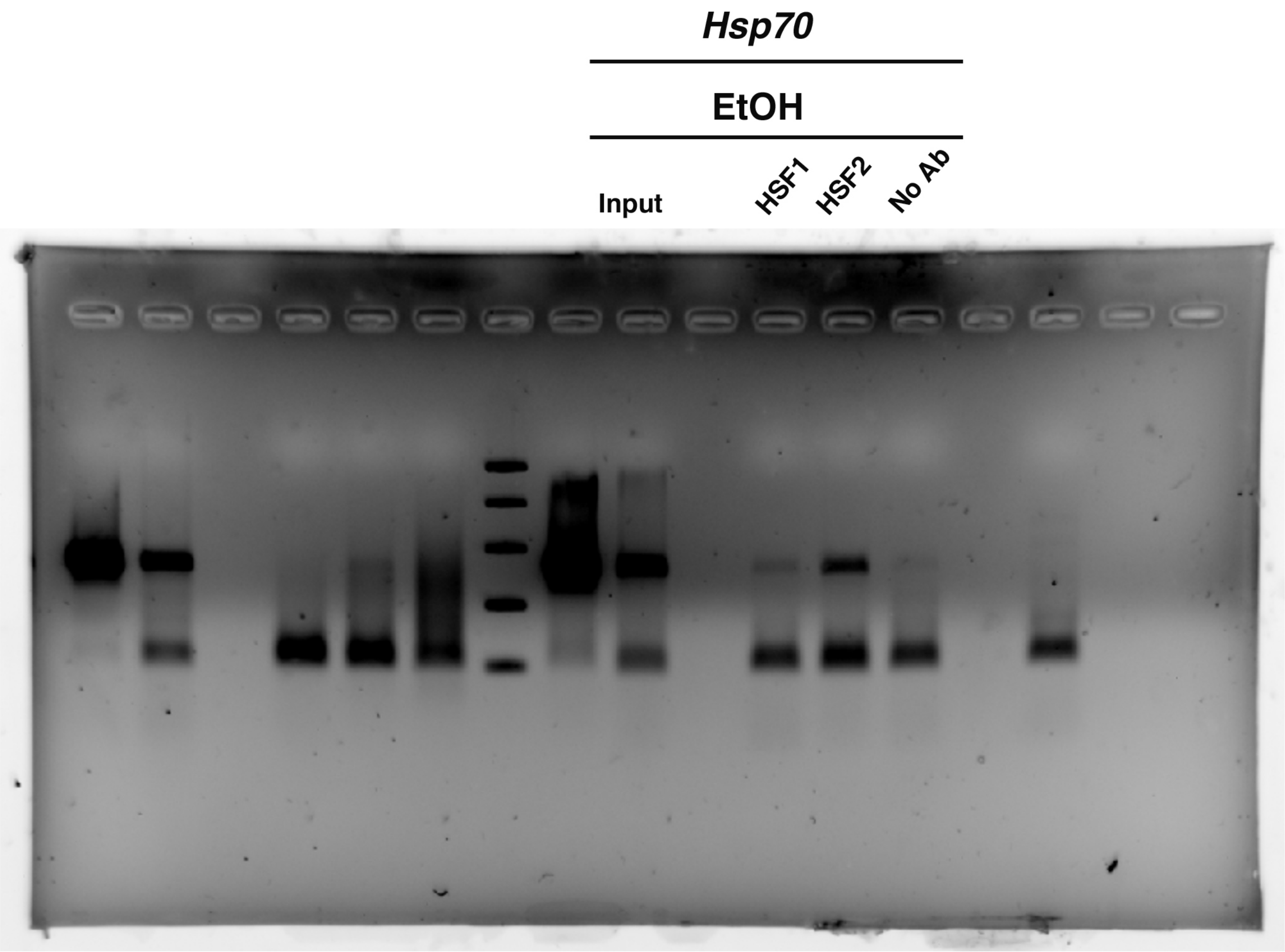

Source data Suppl. FigS5 C El Fatimy et al. p.3

Raw data ChIP iMEF Hsf2 WT CTRL & HS

Cyclo

CTR | HS

Input HSF1 HSF2 No Ab | Input HSF1 HSF2 No Ab

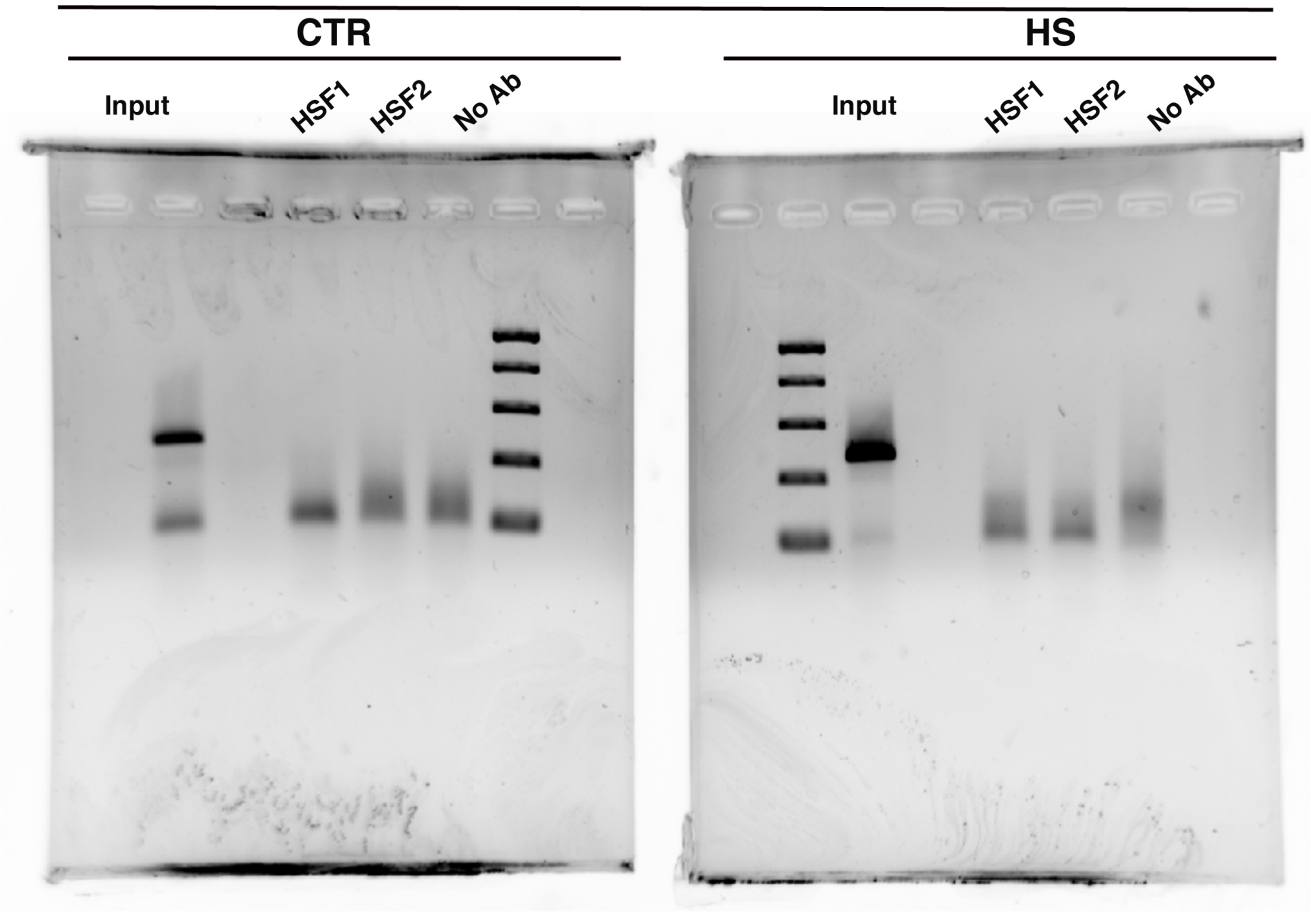

Raw data ChIP iMEF Hsf2 WT CTRL & HS

Cyclo

CTR | EtOH

Input HSF1 HSF2 No Ab | Input HSF1 HSF2 No Ab

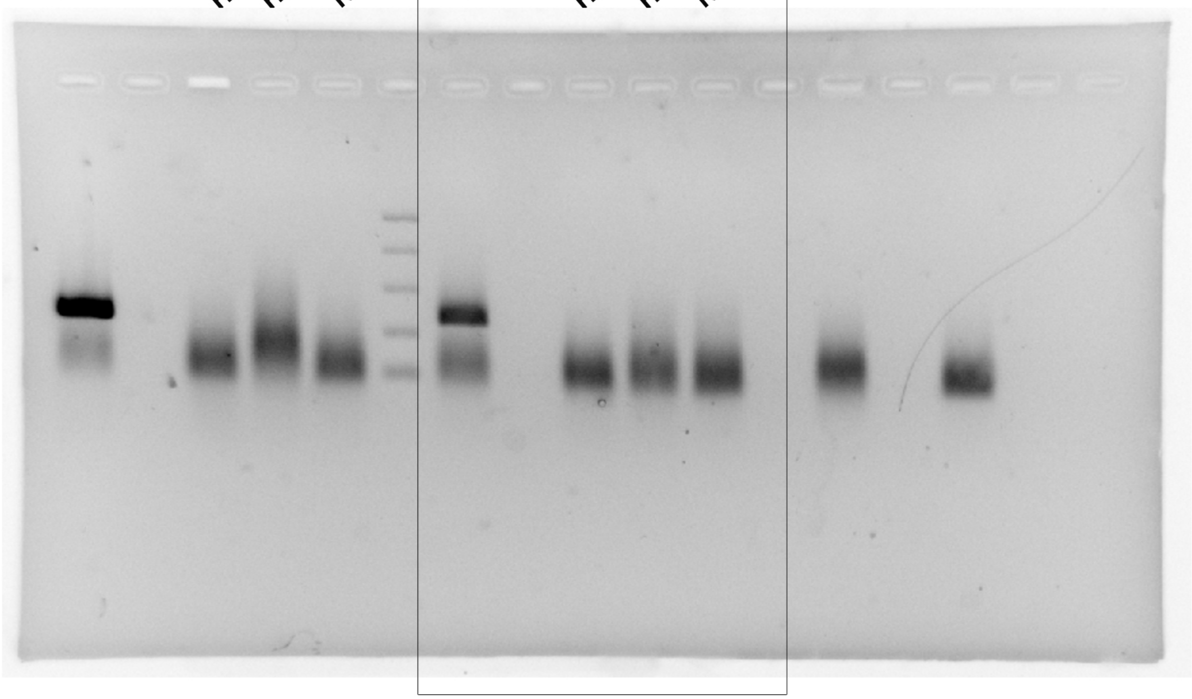

Source data Suppl. Figure S5D El Fatimy

Raw data EMSA gel Fig S5D

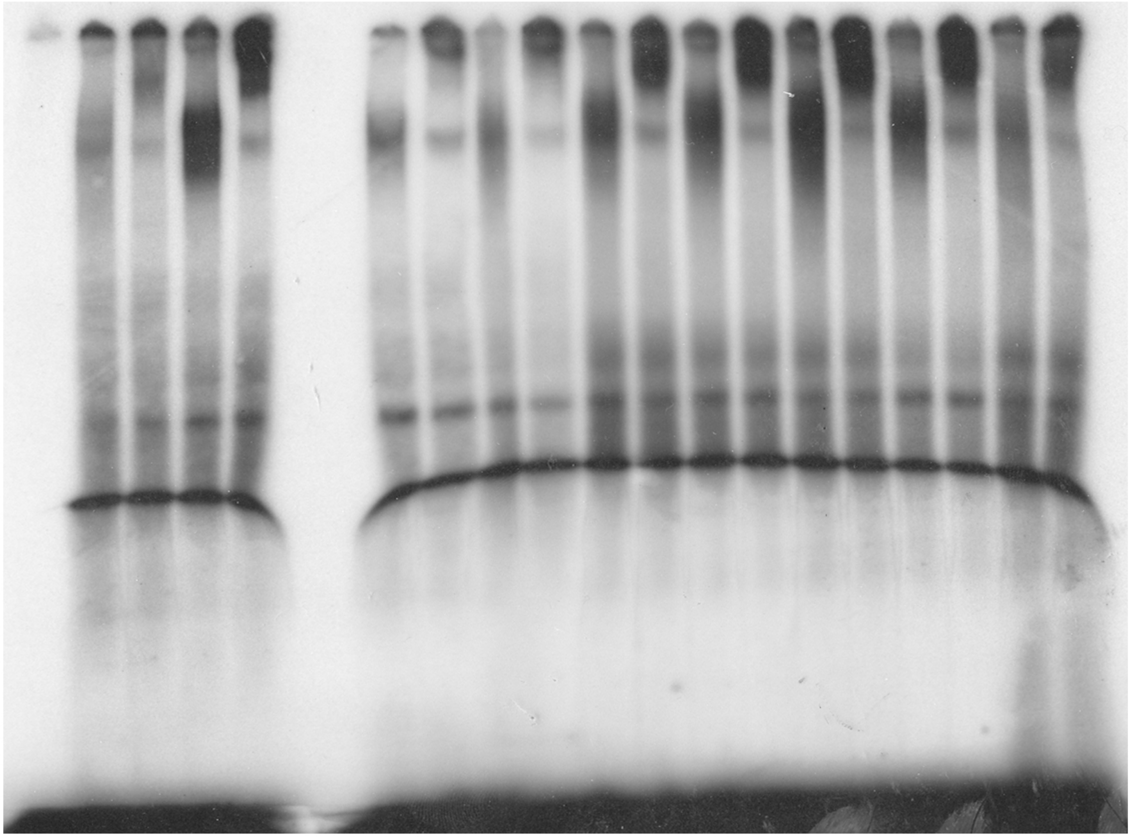

Source data Suppl. Figure S5 F El Fatimy et al

Raw data Suppl. Figure 5F (upper panels)

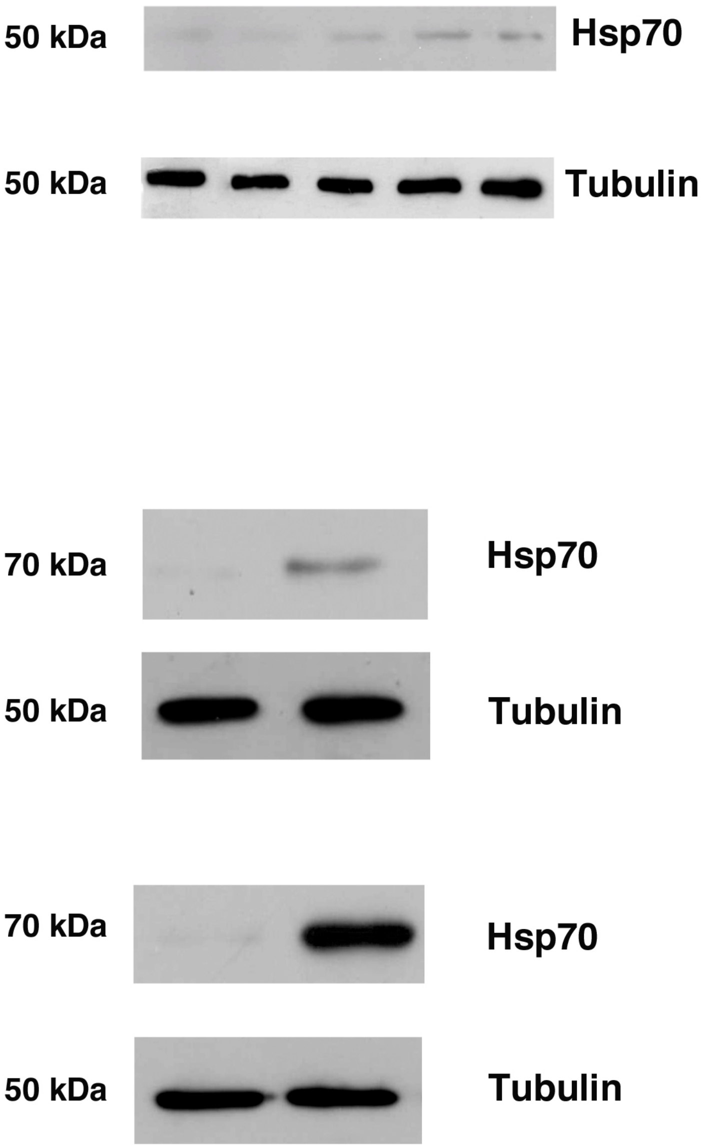

Supplement: Supplementary file 5 [file emmm0006-1043-sd5.pdf]
