# Supplementary material for: Heat shock factor 2 is a stress-responsive mediator of neuronal migration defects in models of fetal alcohol syndrome
Source: EMBO Mol Med. 2014 Jul 15;6(8):1043–61. doi: 10.15252/emmm.201303311 (PMC4154132; doi:10.15252/emmm.201303311)
Supplement: Supplementary file 6 [file emmm0006-1043-sd6.pdf]

Source data Suppl. Figure S6 A El Fatimy et al.

Raw data EMSA gel (lanes of interst within frame)

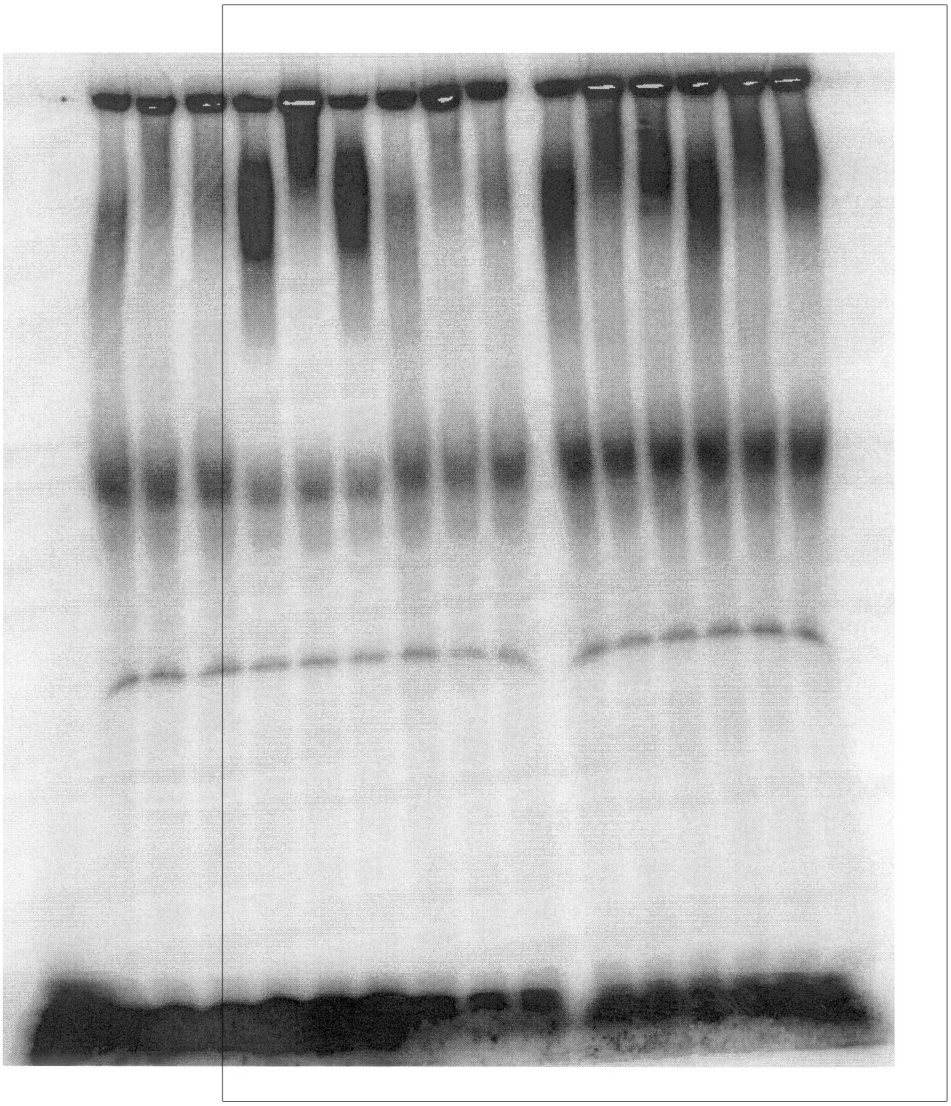

Raw data WB HDAC1 FigS6D

The membranes were cut into 2 pieces incubated either with anti-actin or anti- HDAC antibodies and reconstituted for exposure

Short exposure for HDAC1 and 2 signal

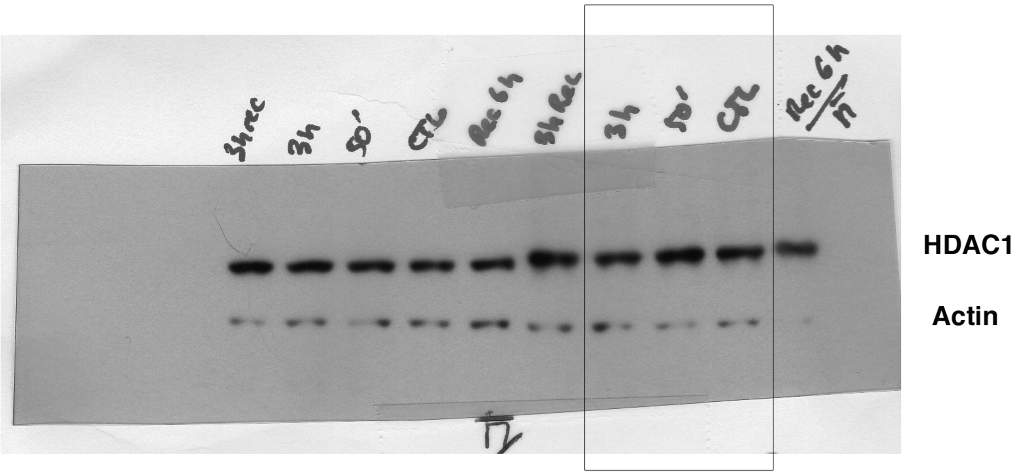

Longer exposure for actin signal

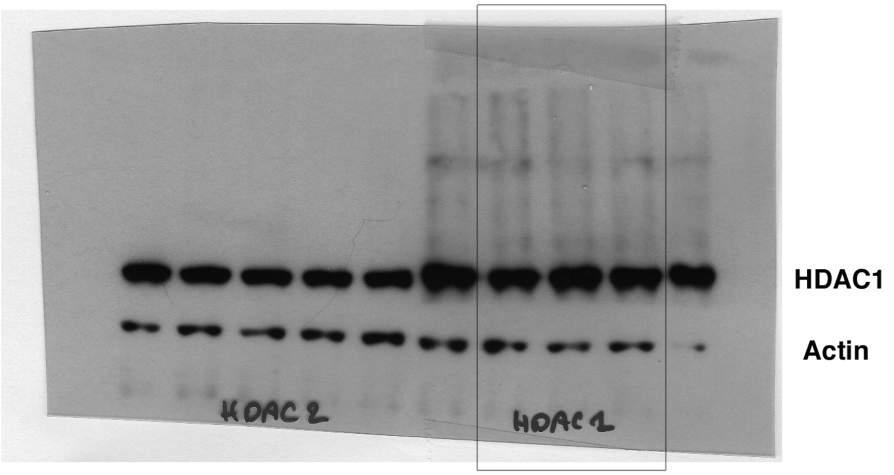

Same membrane with MW

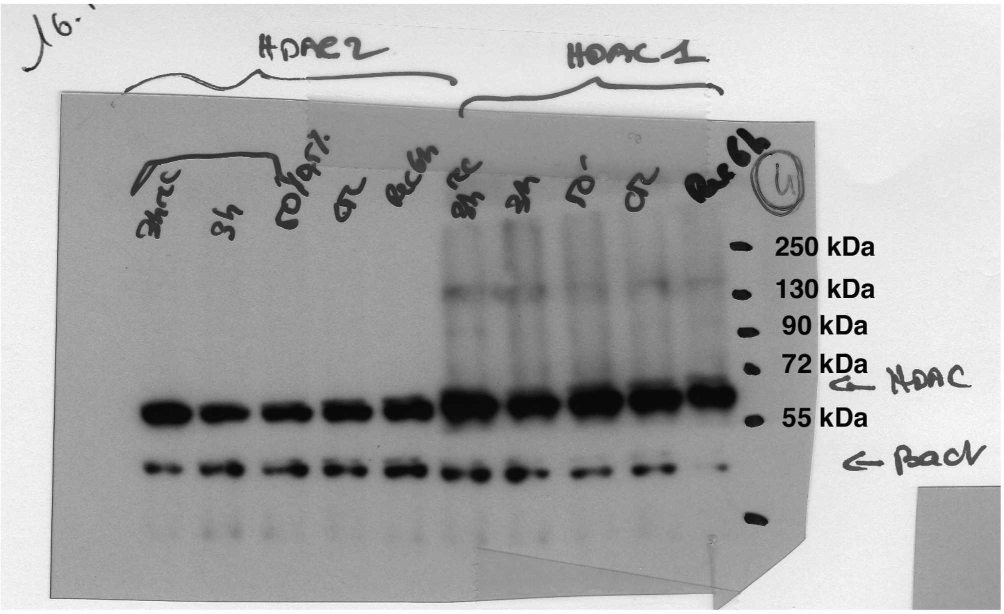

Raw data WB HDAC2 FigS6D

CTR 30' 3 hr

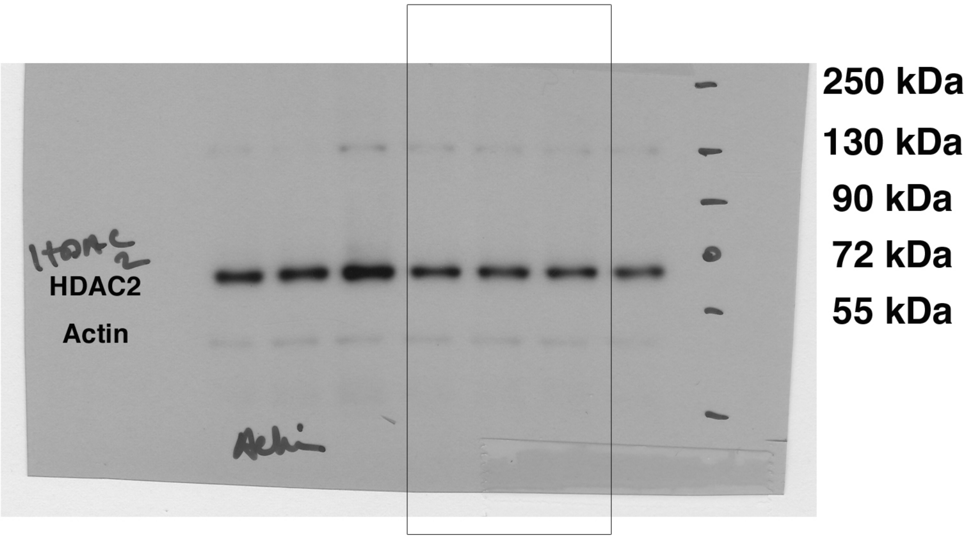

CTR 30' 3 hr

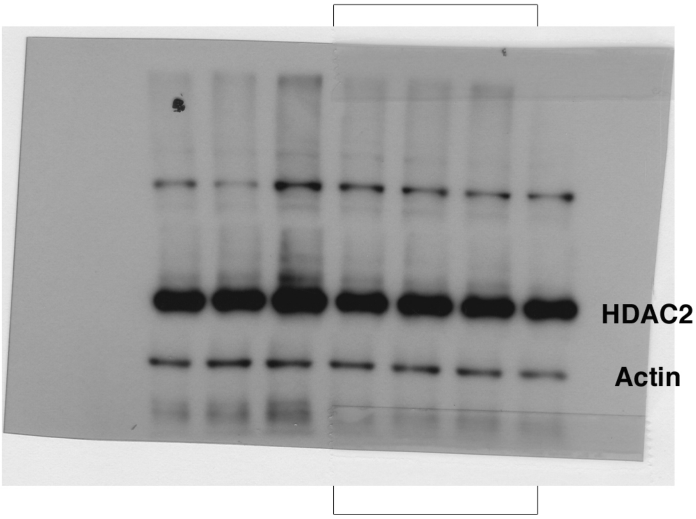

Longer exposure for actin signal

The membranes were cut into 2 pieces
incubated either with anti-actin or anti- HDAC antibodies
and reconstituted for exposure

Raw data WB SIRT1 FigS6D

The membranes were cut into 3 pieces incubated either with anti-actin or anti- HDAC antibodies and reconstituted for exposure

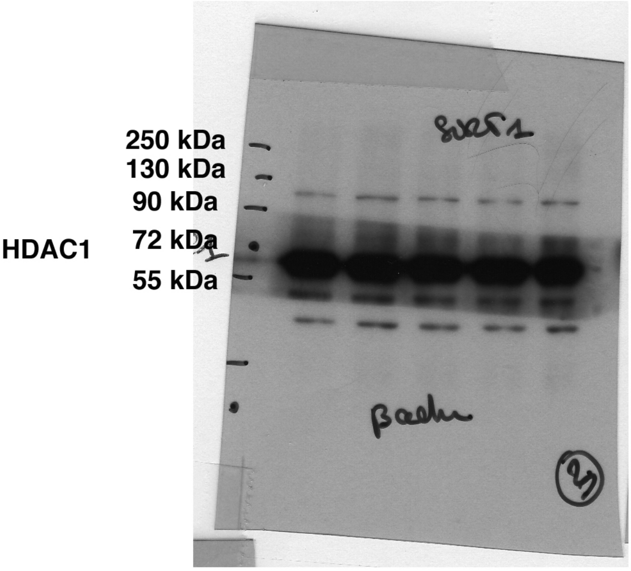

Source data Suppl. Figure S6 E & F El Fatimy et al.

Raw data WB HSF1 Fig S6E (left panel)

75 kDa -

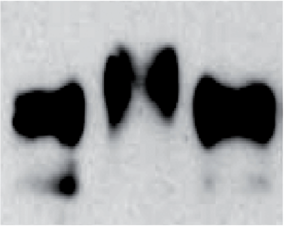

Raw data WB Phospho Ser303/307-HSF1 Fig S6E (right panel)

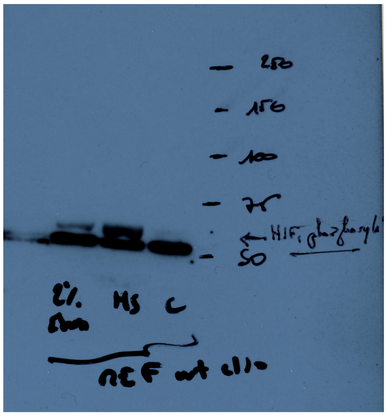

Raw data WB Fig S6F (upper panel)

short exposure

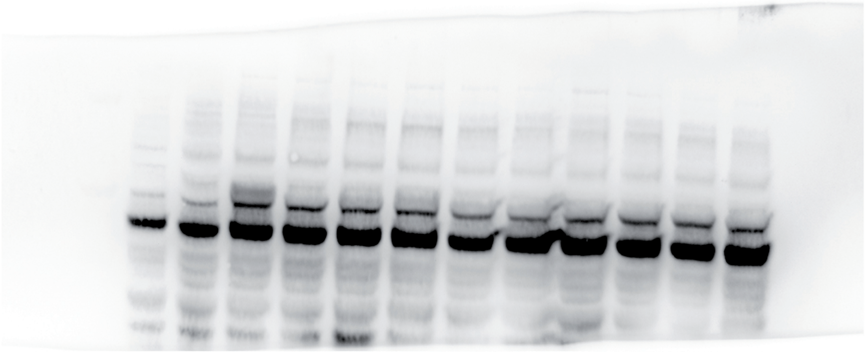

HSF1-SUMO

long exposure

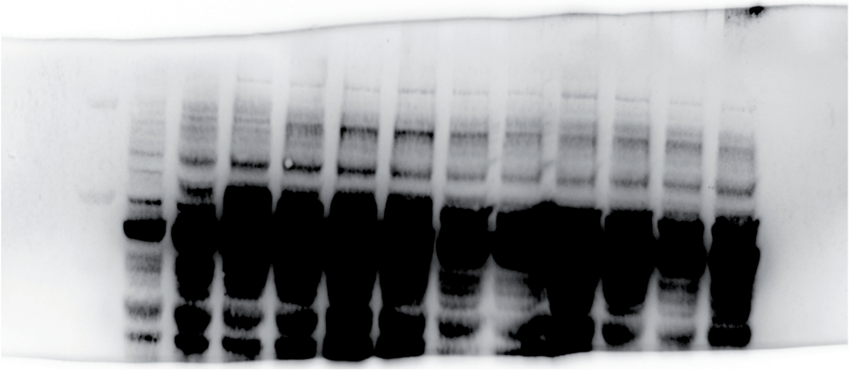

HSF1-SUMO

same membrane MW

175

80

58

46

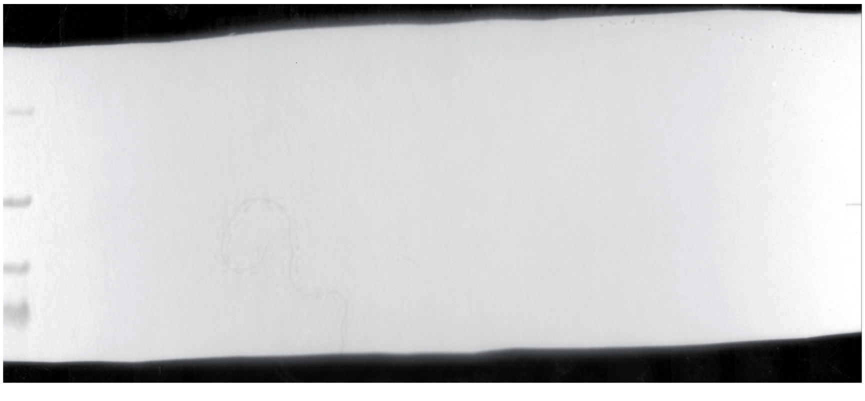

lower panel

58

46

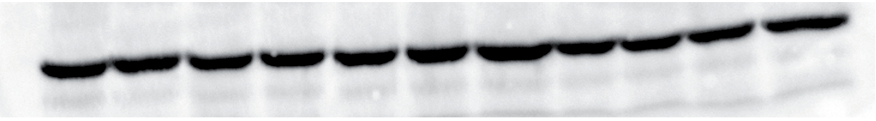

Actin
