# Supplementary figures and images for: Heat shock factor 2 is a stress-responsive mediator of neuronal migration defects in models of fetal alcohol syndrome
Source: EMBO Mol Med. 2014 Jul 15;6(8):1043–61. doi: 10.15252/emmm.201303311 (PMC4154132; doi:10.15252/emmm.201303311)

Source data Suppl. Figure S8 & F El Fatimy et al.

Raw data EMSA gel Fig S8 (lanes indicated by frame)

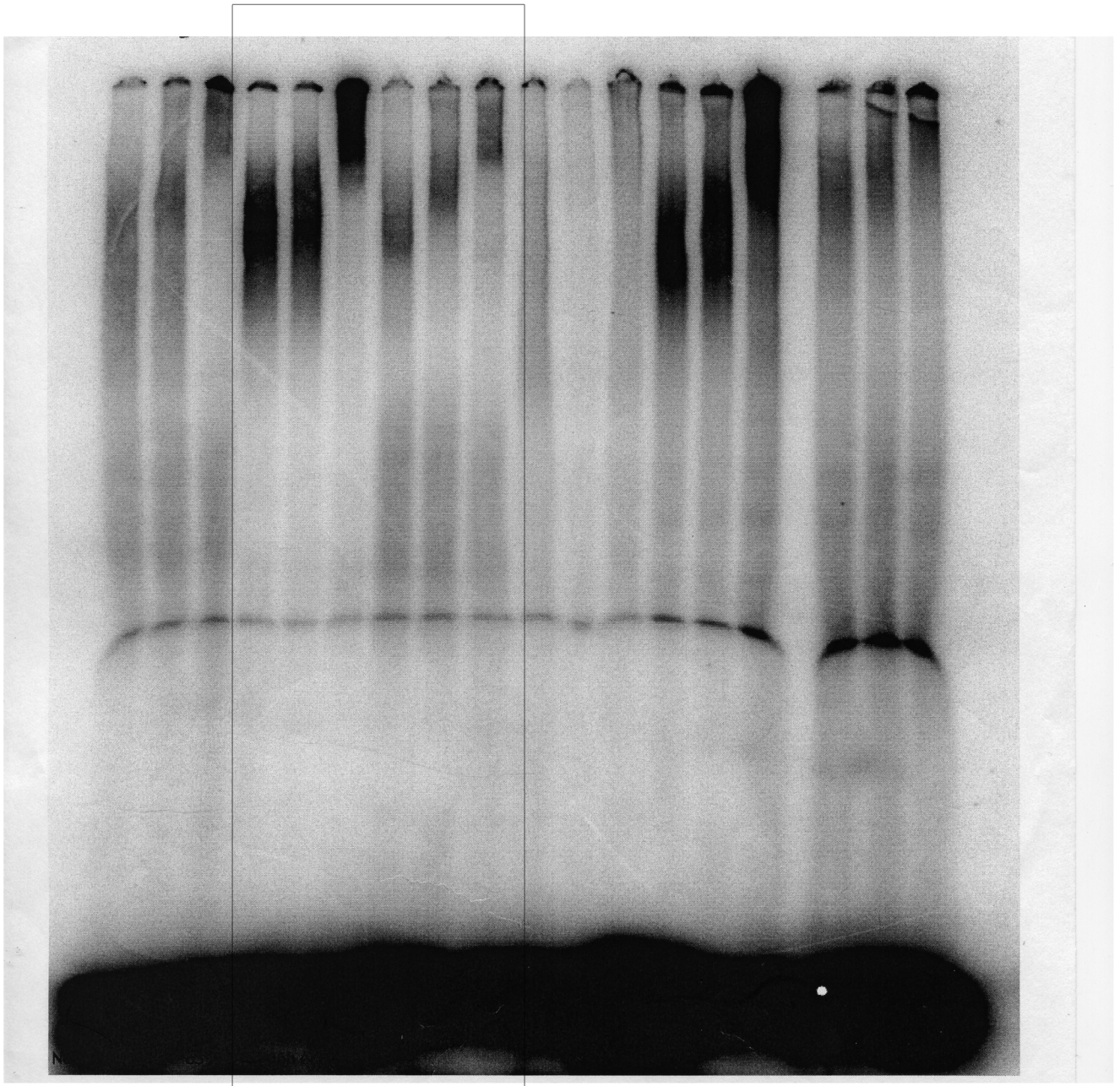

Supplement: Supplementary file 7 [file emmm0006-1043-sd7.pdf]

Raw data EMSA Figure 1B

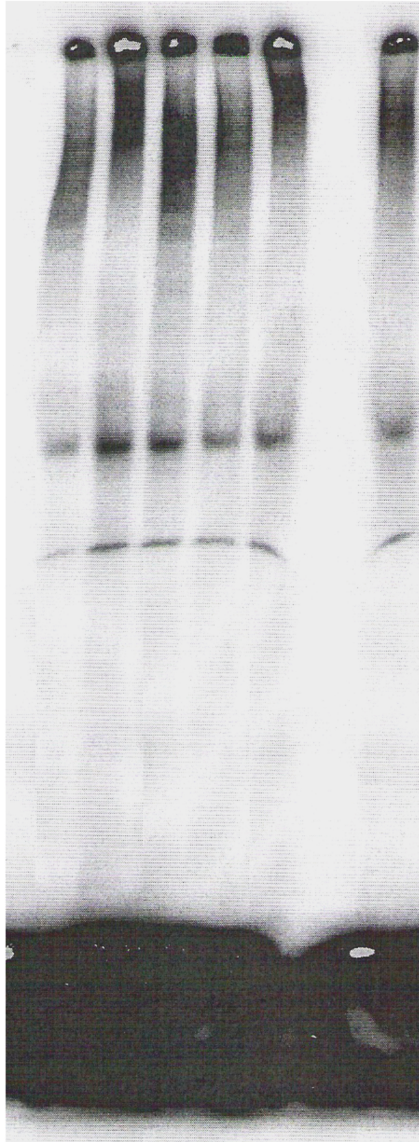

Supplement: Supplementary file 11 [file emmm0006-1043-sd11.pdf]
