# Supplementary material for: Heat shock factor 2 is a stress-responsive mediator of neuronal migration defects in models of fetal alcohol syndrome
Source: EMBO Mol Med. 2014 Jul 15;6(8):1043–61. doi: 10.15252/emmm.201303311 (PMC4154132; doi:10.15252/emmm.201303311)
Supplement: Supplementary file 8 [file emmm0006-1043-sd8.pdf]

Source data Suppl. Figure S9 A El Fatimy

Raw data WB HSF2 EGS crosslinking left-upper panel

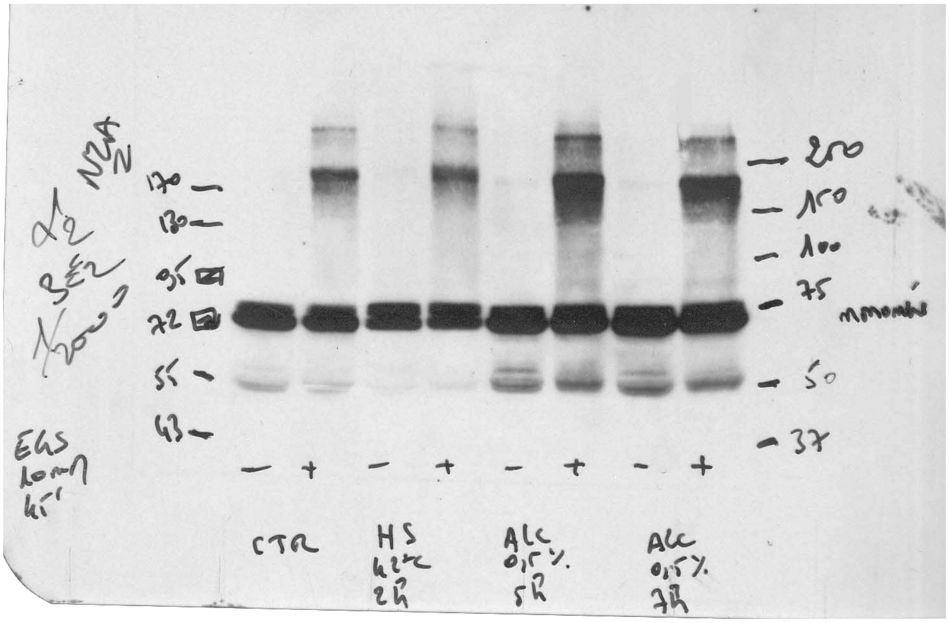


Raw data WB HSF2 EGS crosslinking left-lower panel (shorter exposure)

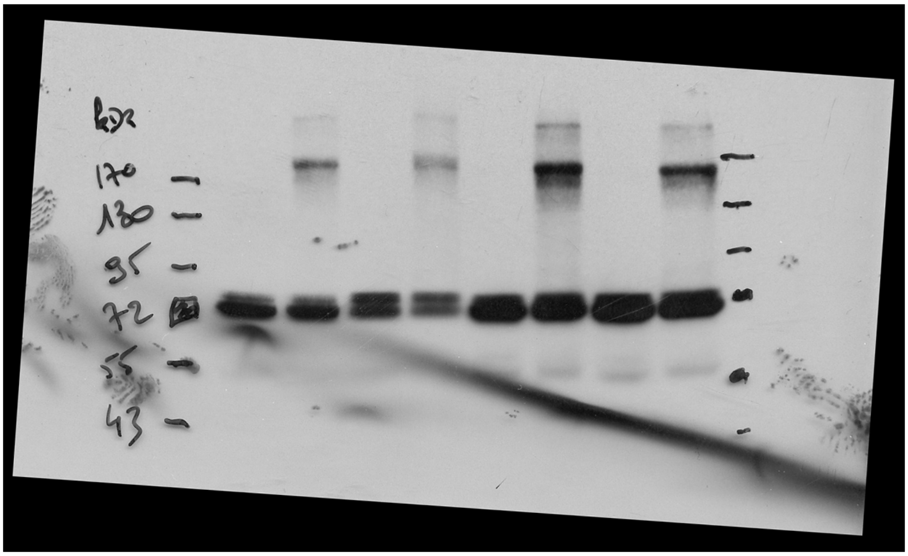


Raw data HSF1 WB EGS crosslinking right panel

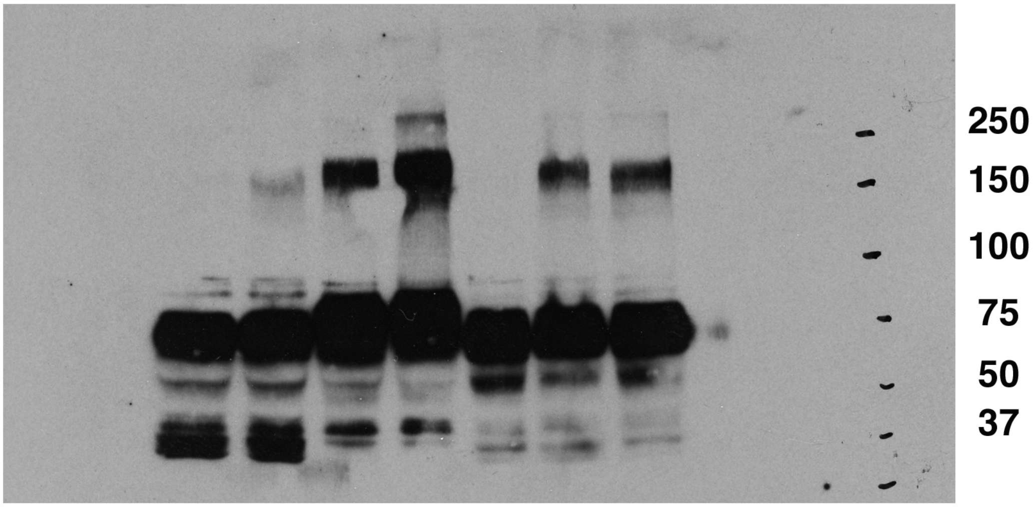

## Source data Suppl. Figure S9B El Fatimy

### Raw data WB EGS crosslinking S9B

**HSF2 (left panel)**

| C | | | EtOH | | | HS | | |
|---|---|---|---|---|---|---|---|---|
| - | EGS | EGS’ | - | EGS | EGS’ | - | EGS | EGS’ |

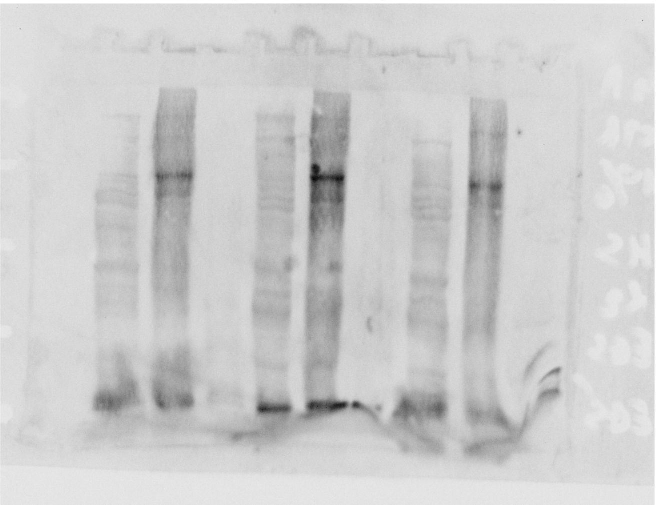

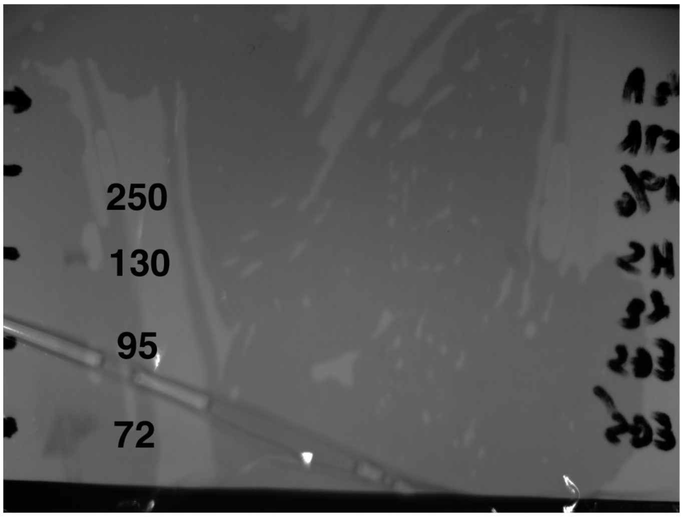


**EGS & EGS’ = 2 different batches of EGS from 2 Manufacturers**

**HSF1 (right panel)**

| C | | | EtOH | | | HS | | |
|---|---|---|---|---|---|---|---|---|
| - | EGS | EGS’ | - | EGS | EGS’ | - | EGS | EGS’ |

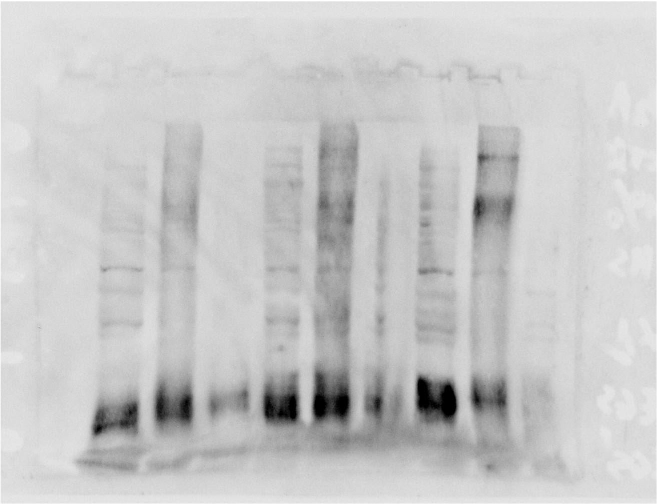

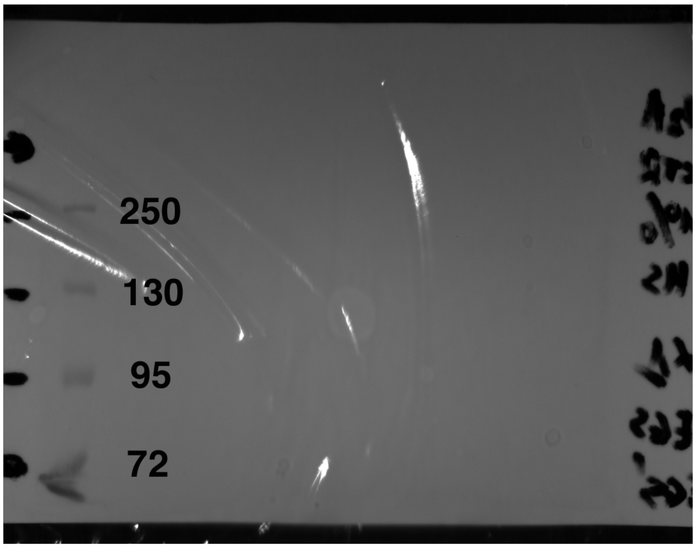

## Source data Suppl. Figure S9C El Fatimy

### Raw data WB EGS crosslinking S9C

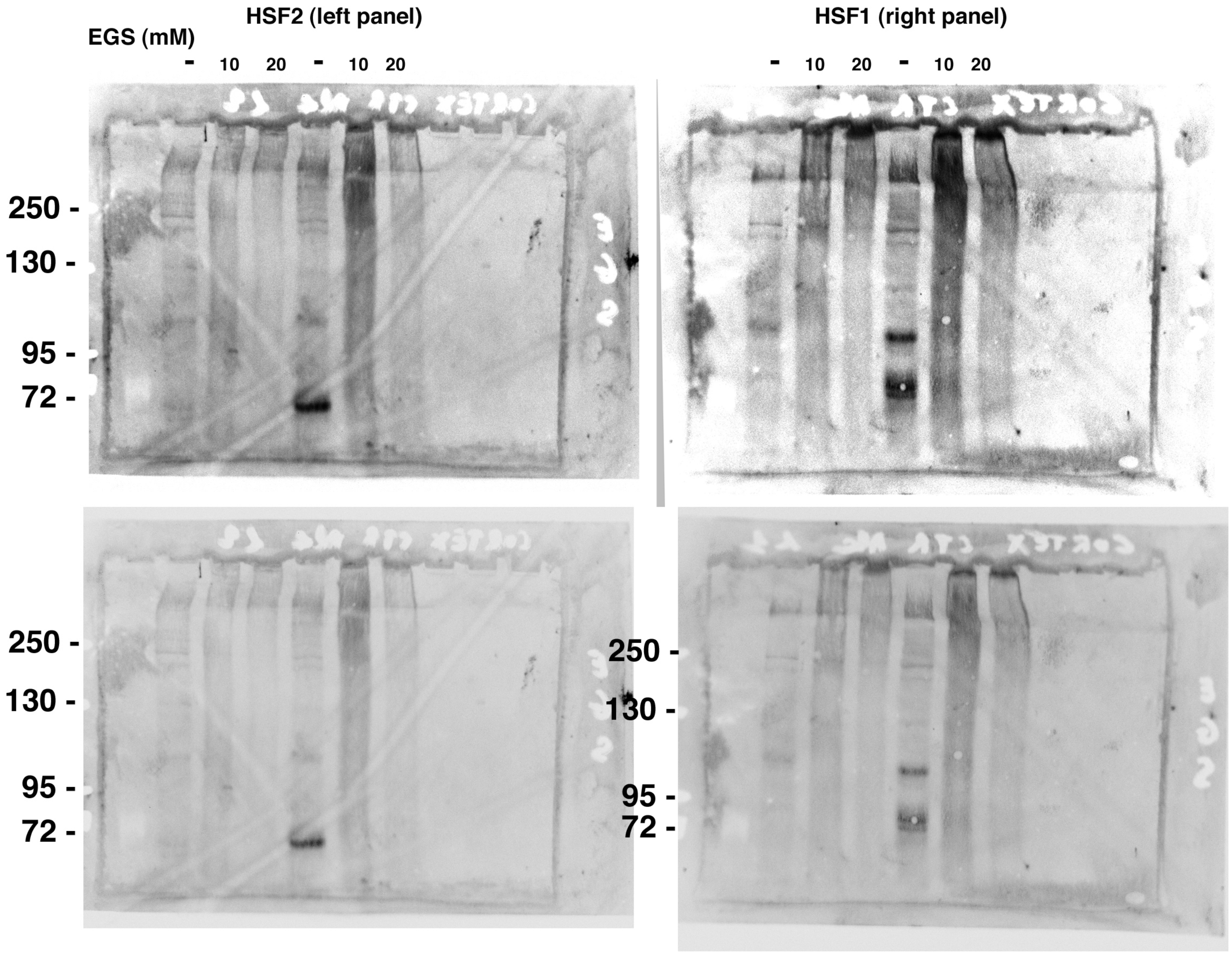


(same membranes - lower exposure)
