# Supplementary material for: Heat shock factor 2 is a stress-responsive mediator of neuronal migration defects in models of fetal alcohol syndrome
Source: EMBO Mol Med. 2014 Jul 15;6(8):1043–61. doi: 10.15252/emmm.201303311 (PMC4154132; doi:10.15252/emmm.201303311)

Source data Suppl. Figure S10 A El Fatimy et al.

Raw data EMSA gel (left panel) (lanes of interest within frame)

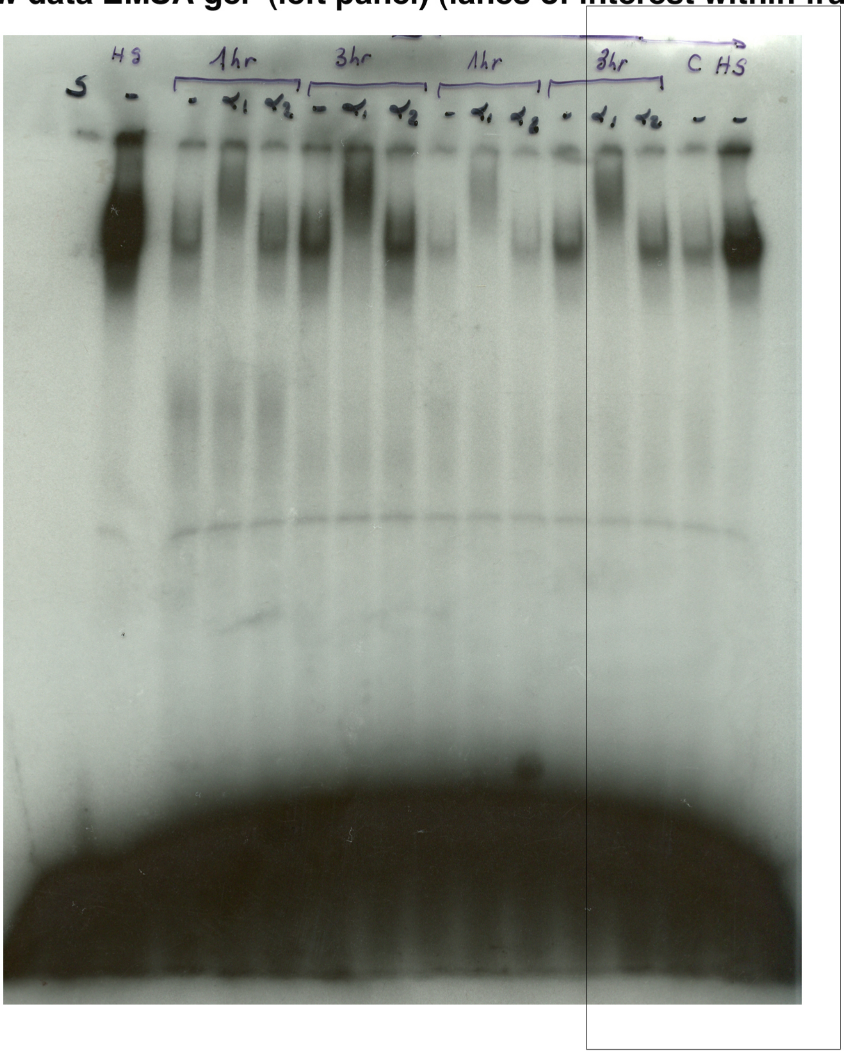

Raw data EMSA gel (left panel) (lanes of interest within frame)

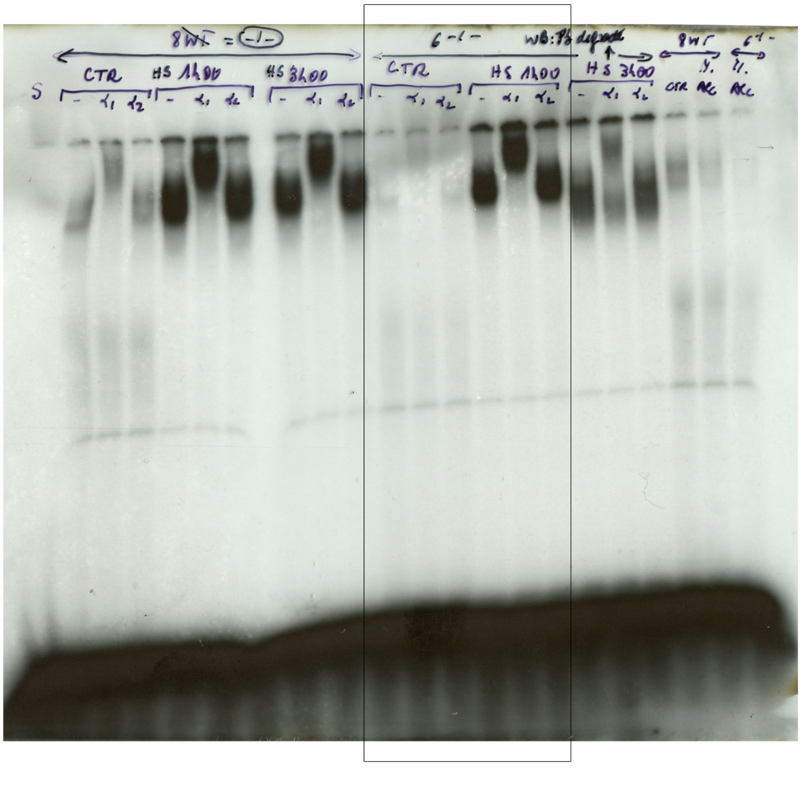

Source data Suppl. Figure S10 C El Fatimy

Raw data WB HSF2 Fig S10 C

HSF2 (taggued with Streptavidin binding protein and 2 protein G domains)

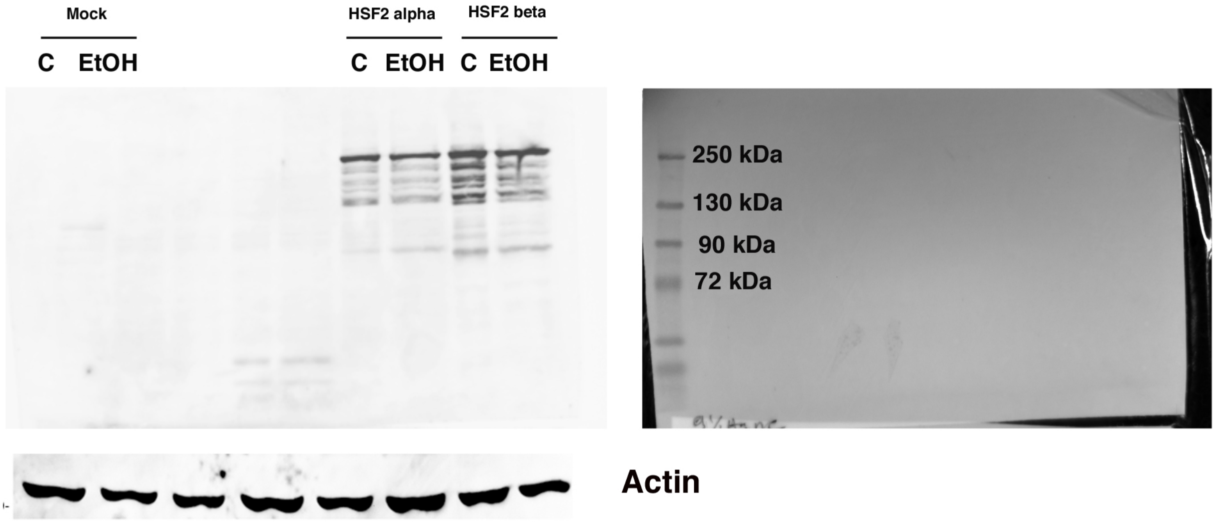

Source data Suppl. Figure S10 D El Fatimy

Raw data WB HSF1 (within frames) Fig S10 D

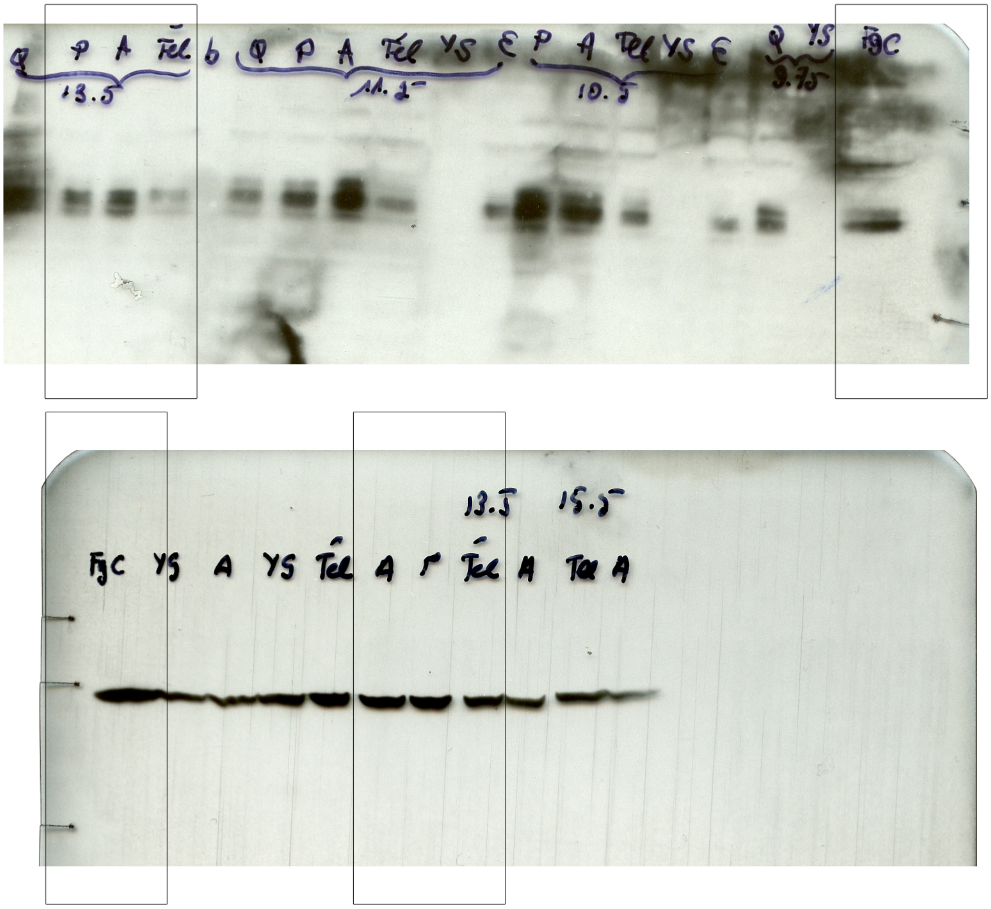

Supplement: Supplementary file 9 [file emmm0006-1043-sd9.pdf]
