# Supplementary material for: Heat shock factor 2 is a stress-responsive mediator of neuronal migration defects in models of fetal alcohol syndrome
Source: EMBO Mol Med. 2014 Jul 15;6(8):1043–61. doi: 10.15252/emmm.201303311 (PMC4154132; doi:10.15252/emmm.201303311)
Supplement: Supplementary file 10 [file emmm0006-1043-sd10.pdf]

Heat shock factor 2 is a stress-responsive mediator of neuronal migration defects in models of fetal alcohol syndrome

Rachid El Fatimy, Federico Miozzo, Anne Le Mouël, Ryma Abane, Leslie Schwendimann, Délara Sabéran-Djoneidi, Aurélie de Thonel, Illiasse Massaoudi, , Liliana Paslaru, Kazue Hashimoto-Torii, Elisabeth Christians, Pasko Rakic, Pierre Gressens, Valérie Mezger

Corresponding author: Valérie Mezger, CNRS University Paris Diderot

Review timeline:

Submission date:	23 July 2013
Editorial Decision:	21 August 2013
Revision received:	22 March 2014
Editorial Decision:	14 April 2014
Revision received:	01 June 2014
Accepted:	02 June 2014

Transaction Report:

(Note: With the exception of the correction of typographical or spelling errors that could be a source of ambiguity, letters and reports are not edited. The original formatting of letters and referee reports may not be reflected in this compilation.)

Editor: Céline Carret

1st Editorial Decision 21 August 2013

Thank you for the submission of your manuscript to EMBO Molecular Medicine. We have now heard back from the two referees whom we asked to evaluate your manuscript. As you will see from the reports below, the referees had a very different take on the data. While Ref.1 raised a long list of issues and serious concerns not supporting publication, Ref.2 found the study "comprehensive" and was much more positive about it. We have therefore asked the opinion of an editorial adviser, whose comments are also pasted below. Following extensive discussion within our editorial office, we decided to give you the opportunity to provide a revised version of the manuscript with the understanding that he referees' concerns must be addressed as much as possible, in an order of priority, as ranked by our adviser (see below).

I should remind you that it is EMBO Molecular Medicine policy to allow a single round of revision only and that, therefore, acceptance or rejection of the manuscript will depend on the completeness of your responses included in the next, final version of the manuscript. I realize that addressing the referees' comments as advised would involve a lot of additional experimental work and will be time consuming, and I am uncertain whether you will be able (or willing) to return a revised manuscript to satisfy our referees and adviser within the 3-6 months deadline.

On the other hand, given the potential interest of the findings, I would be willing to consider a revised manuscript as I said, but in order to save you from any frustrations in the end I would

© EMBO

evaluate the revision first at the editorial level and may send it back to the referees only if the revision is deemed as complete as it can be. I would therefore understand your decision if you choose to rather seek rapid publication elsewhere at this stage.

Should you find that the requested revisions are not feasible within the constraints outlined here and choose, therefore, to submit your paper elsewhere, we would welcome a message to this effect.

***** Reviewer's comments *****

Referee #1 (Comments on Novelty/Model System):

Novelty is low because the data do not support the overarching claims of the manuscript.

Referee #1 (Remarks):

Reviewer's report

In this paper the authors analyze the involvement of the Heat Shock Factor 2 (HSF2) in radial neuronal migration defects caused by Fetal Alcohol Exposure (FAE). To asses the importance of this transcription factor in brain development under ethanol exposure the authors study three different protocols of chronic fetal alcohol exposure in mice and different cell lines. The authors analyze the effect of ethanol in HSFs DNA-binding ability by gel shift assays and ChIP experiments and showed that under control conditions HSF2 presents higher DNA-binding activity to Heat Shock Elements (HSE) compared to HSF1. Under ethanol exposure, HSF1 and HSF2 form heterotrimers and it negatively affects HSF2-target genes expression involved in neural migration. The importance of HSF2 in neural migration defects under ethanol exposure is suggestd by the observation the Hsf2-/- mice showed less severe effects in brain development. Some concerns about the results and conclusions presented in this manuscript indicate that additional work and statement clarification is extremely important before publication. At the current stage, while there is a large amount of data presented, the data do not support the conclusions drawn in the manuscript.

Major concerns

-The authors state that HSF2 is involved in brain cortical development, as suggested by other studies, through target genes distinct from Hsps. In addition, the authors previously published the importance of HSF2 in the regulation of the two activators of neuronal migration, p35 and p39 (Chang et al., 2006). The main conclusion of this work is based on the less severe effect of Hsf2-/- mice exposed to ethanol compare to Hsf2+/+. However, it is difficult to connect the fact that Kallio et al., 2002 and other authors demonstrated that Hsf2-/- mice suffer brain abnormalities under control conditions with the results provided in this study. How do the authors explain that HSF2 is essential for neural migration gene expression but does not have negative phenotype when it is knockout?

-In the results section the authors claim that ®HSF1 was expressed at each stage of corticogenesis® but the results showed in Figure 1A only showed HSF1 protein expression in the telencephalon for E10.5 stage and no expression for E15.5, neither in telencephalon nor in forelimb. How do the authors explain that HSF1 is visualized ubiquitiously in the developing brain in figure 2B (right panel) at E16.5 stage but it is not observed by western blot in figure 1A?

-The fact that CAI induced nuclear localization in the cortical plate is not clear from the pictures showed in figure 1F. The authors may considere performing nuclear and cytoplasm fractionation from cortex samples and analyze HSF1 protein levels by western blot.

-In the introduction the authors state that ®HSF2 controls the expression of NdeI, Dcx and Dclk1...®. However the results shown in figures 2C and 6B do not support this statement for the NdeI gene. The expression of NdeI, in figure 2C, does not change as a consequence of CAI treatment and the purported HSF1-HSF2 heterotrimer formation. Additionally, the expression

obtained for NdeI in Hsf2+/+ control conditions in figure 6B does not correlate with results shown in 2C.

-The genes analyzed by ChIP in figure 2B do not show a characteristic pattern to demonstrate that CAI affects HSF2 gene expression targets. NdeI and Dclk1 show similar HSF1-HSF2 promoter-binding profile in control and CAI conditions, but different expression changes analyzed by RT-qPCR. In addition, Dclk1 and p35 showed similar mRNA decreases after CAI treatment but there is no correlation with HSF1-HSF2 promoter-binding. The authors might analyze a higher number of HSF2 gene targets to demonstrate that ethanol exposure indeed affects HSF1-HSF2 promoter-colocalization and that alters the expression levels of HSF2 gene targets. Does Dcx gene have an HSE sequence? Why is this gene not included in ChIP experiments?

-The authors may also consider analyzing the effect of Heat Shock treatment by ChIP experiments on the neuronal migration genes to demonstrate that alteration in HSF1-HSF2 promoter-binding is specific for CAI. Although the authors claim that gel shift assays are different in EtOH and HS, the HSE used for gel shift assays correspond to a consensus HSE while the analyzed genes NdeI, Dclk1 and p53 do not present a standard HSE and they could be regulated by HSF1 under different conditions.

-The use of Neuro2A as a model for the experiments is based on the statement that ®this cell line displayed constitutive HSF2-binding activity in control conditions, similar to fetal cortices®. However, results shown in figure 3A for N2A under control conditions do not show constitutive HSF2-binding activity as it is observed for fetal cortices in figures 1C and 1E. In addition, the authors also say in the results section that HSF1 displayed some constitutive DNA-binding activity in N2A, which does not occur for fetal cortices. If this is the case, how is it possible to state that CAI changes the HSF DNA-binding ability in this cell line when HSF2 and HSF1 present constitutive DNA-binding activity? Is N2A a good cell model for these studies?

-The ethanol treatment seems to decrease HSF1 acetylation levels compared to HS conditions, a factor that is associated with HSF1 repression, and incubation with Histone deacetylases inhibitors restored HSF1 acetylation levels after EtOH treatment. The authors may consider analyzing HDAC mRNA or protein levels in their experimental conditions to better correlate their findings using TSA. In addition, they might consider the work published by Agudelo et al., 2011 (doi: 10.1111/j.1530-0277.2011.01492.x.) that demonstrates that HDAC2 is upregulated by EtOH using neuronal cell lines. The authors might also consider that alteration in HDAC activities and protein acetylation levels can also provoke chromatin remodeling alterations as it has been previously published by Padey et al., 2008 (Brain chromatin remodeling: a novel mechanism of alcoholism). They might consider the possibility that chromatin remodeling can be the main factor of HSF1-HSF2 DNA-binding activity alteration under EtOH exposure and that can explain changes in gene expression of neural migration genes.

-In the results and discussion sections, the authors claim that sumoylation modification is prolonged under ethanol exposure compared to HS conditions. However, this statement is not supported by the results shown in figure 3G. Sumoylation levels are lower than in HS conditions at 0.5 and 1 h treatment but similar at 3 and 6 h treatment between EtOH and HS. They authors might remove this conclusion that is not supported by the data presented.

-In figure 5D, the authors analyze the effect of Hsf2-/- in mice under intraperitoneal injection of EtHO and the HSF1-HSF2 DNA-binding activity. However, Hsf2+/+ control does not show HSF1 DNA-binding, as previously shown for CAI. In this case it is not possible to correlate that the loss of binding of HSF1 is due to the absence of HSF2 when HSF1 is not binding under EtOH treatment in Hsf2+/+.

-They authors might perform ChIP experiments in Hsf2-/- mice under ethanol conditions for HSF1, to analyze if the neuronal migration genes NdeI, Dcx, Dclk1 and p35 are regulated entirely by HSF1 in the absence of HSF2.

Minor comments:

-In the results section the authors claim that ®CAI caused the loss of HSF2 binding and HSF1 binding to the p35 HSE...®. However, results shown in figure 2C only demonstrated that HSF2 losses binding after CAI but HSF1 never binds in control or CAI conditions. Similarly, in the introduction section the authors state that ®the modification of HSF1 and 2 activities disturb the expression of p35®. However, I would say that only HSF2 activity alteration disturbs p35 but not HSF1 because this protein does not seem to regulate p35.

-Figure 3D bottom panel says VPA instead of TSA.

- Using a HSE probe that could bind only one trimer and gel shift assays are not direct experiments to state that HSF1-HSF2 form heterotrimers. It can be a possibility that HSF1 and HSF2 homotrimers can independently bind to different molecules of the HSE in the same sample or one protein off the DNA can interact with the other protein bound to DNA. They authors should explain this.

-Figure 5D legend must explain what is e1,e2,e3,e4.

-Figure 6C is not commented in the text.

Referee #2 (Remarks):

The manuscript by EL FATIMY et al., represents an important study investigating the molecular mechanisms underlying fetal alcohol syndrome, which is common pathology. The authors have used an interdisciplinary approach, which revealed that upon exposure to ethanol, HSF2 is essential for the triggering of HSF1 activation by alcohol, thereby steering the formation of alcohol-specific HSF1-HSF2 heterocomplexes. This disturbs the physiological in vivo binding of HSF2 to heat shock elements (HSEs) in genes that control neuronal migration in normal conditions, such as p35 or MAPs (microtubule-associated proteins; Dclk1, Dcx, Nde1), and alters the expression of these genes. Overall, this is a comprehensive and exciting study. I have some comments, which may improve the manuscript.

1. In the introduction the authors do not refer to HSF4.

2. Fig. 1 shows only a histogram of the neuronal positioning by BrdU. Neuronal migration is one of the major issues of this manuscript, and appears in the title. At least representative images of the migration phenotype should be presented (this comment relates also to figure 6). In the methods part the related sentence should be corrected "BrdU positive cells were counted on 6 whole VZ and SVZ? cortices from 3 independent litters". The methods relate also to short BrdU experiments in the mice, but I do not see those results. In addition, the methods mention the wound healing assay, however this assay is not referred to in the rest of the text.

3. Fig. 1A, shows a western blot, however the control cells do not seem to express HSF2 alpha.

4. Fig. 1F, a higher magnification may allow to visualize better the differences.

5. Fig. 2B, the related test should be somewhat modified; no difference in c/ca1 of nde1 HSF2 and there is a reduction in HSF2 in Dclk1 c/ca1. It should be useful to add ChIP for Hsp70.

6. Fig. 2C: the changes in mRNA levels of Dcx, Dclk, Nde1 following fetal alcohol exposure are presented and are very interesting. The confirmation on the protein level will support the hypothesis

that migration defect in FAE is mediated by reduced transcription of these genes. Are the changes in Nde1 significant as indicated?

7. Fig 6F: loading control is lacking.

8. In the text - p12, 6 lines from bottom: refer to Fig. 4A, should be 4B.

Editorial Adviser:

The work by El Fatimy et al. addresses a critical developmental problem with direct public relevance: fetal alcohol syndrome. Whilst the Authors estimated and referenced 1 in 100 pregnancies negatively impacted by alcohol, at-risk populations in Europe and beyond exist with much higher rates of incidence for this condition. The Authors present a mechanistic study identifying HSF2 as a "sensor" for developmental defects through its interaction with HSF1. The data seem to be novel and by and large convincing. Yet some elements are tenuous, which perhaps is due to the "tour de force" type analysis the Authors have taken as their course of action.

As a third reviewer, arbitrating between the original ones, it would seem inappropriate to ask new questions. Instead, I will focus on supporting the original Referees' queries by providing a subjective yet likely helpful list of priorities.

Overall, the manuscript is a very difficult read because it is packed with data. At some point it tends to slip into being excessively confirmatory of previous findings. The six figures are unusually packed, the panels are not referenced in their order of appearance, and often too small to see details. Therefore, it would be helpful for the reader to only show what really is important in the main figures, and then drop some of the more auxiliary data into supporting information. Perhaps a new figure should be generated for the first half of Fig. 1, which Referee 1 clearly, and rightfully, has criticized.

Referee 1's points #1, #3, #4, #7, #8, #10 are most critical and must be followed up. For #7 (N2A cell line), primary cortical neurons or similar positive controls must be introduced.
Point #2: is due to having a too small figure and overloading the positive control, which rendered the really important lanes partly obscure.
Point #5: is again very credible and needs to be dealt with, particularly in relation to the Dcx question.
Point #6: (HSE sequences) comment is entirely fair, which I think anyone non-familiar with these interactions would have problem with. Perhaps explanation of how relaxed these consensus sequences can be, and how many different types there are should be mentioned in the text.
Point #9: The SUMOylation data needs to be rearranged and the text clarified such that it becomes a readable piece. This is one of the key components of the paper but seems to be crammed into a few panels, which are small, of relatively low quality (on the PDF), and less informative than they should be. The Referee's comment certainly must be followed.
Point #11 might be of less importance if the above concerns are exhaustively addressed.

(Minor comments are straightforward).

For Referee 2, I agree with her/his critical point (#2) on the lack of representative images in Figures 1 and 6, which gives the lingering impression of substandard data quality. This certainly should be addressed, and accommodated. Points #3, #4, #5 and #7 will need to be dealt with. I would perhaps deemphasize #6, except the query about the statistical significance, which I think is erroneously reported. The other queries are self-explanatory.

1st Revision - authors' response 22 March 2014

RESPONSE TO REFEREES

We have tried to keep our responses as short as possible, as requested by the editor. However, several of the points raised by the referees are subtle, and we have preferred to explain these in detail here rather than adding unnecessary bulk to the manuscript. We have numbered our answers point **#1.1** for Referee #1's **point 1**, etc. The figures included in our response to the Referees have also been numbered accordingly, e.g. Figure #1.1 corresponding to point #1.1.

Referee #1 (Comments on Novelty/Model System):

Novelty is low because the data do not support the overarching claims of the manuscript.

Many aspects of this study are strikingly different from previous reports in the literature. While we now discuss the novelty of our study in greater detail in the revised manuscript, we would like to make certain aspects of our work clear here.

- Our work is the first to demonstrate a prominent role for HSF2 in the stress response *in* vivo in the developmental period: it is necessary to activate HSF1 under conditions of environmental stress and not merely to modulate HSF1-dependent gene expression, as for example in the case of cell systems or preimplantation embryos subjected to classic heat shock (42°C-43°C; Östling 2007, *JBC*; Le Masson et al., 2011, *Mol Cell Biol*).
- The persistent activation of HSF1 and HSF2 by prolonged stress is also new. Indeed, both HSF1 and HSF2 are inactivated by prolonged heat shock- the former over time and the latter rapidly (Kline & Morimoto 1997; Ahlskog 2010). However, this inactivation does not occur with chronic alcohol exposure, possibly due to the alcohol-specific post-translational modifications that we observed in HSF1.
- The existence of HSF1-HSF2 heterotrimers, while a subject of speculation for a long time, has never been conclusively demonstrated *in vivo* and under conditions of stress relevant for human health.

In addition, the alcohol-induced heterotrimers we observed both *in vivo* and *ex vivo* display biochemical properties (e.g. in gel-shift assays and glycerol gradients) that differ from those described previously.

- Finally, we describe that this new and essential mode of HSF2 action affects the occupancy and expression of target genes involved in the radial migration of cortical neurons, thus providing - for the first time - a molecular explanation for the neuronal positioning defects characteristic of Fetal Alcohol Syndrome.

The fact that the enigmatic HSF2, whose role in the stress response has remained elusive for so many years, occupies such a prominent place, both under adverse

environmental conditions and at the level of the development of the organism, is therefore of great significance. In addition, our alcohol-induced stress conditions have real pathological relevance in terms of human health.

#1.1- "The main conclusion of this work is based on the less severe effect of Hsf2-/- mice exposed to ethanol compare to Hsf2+/+. However, it is difficult to connect the fact that Kallio et al., 2002 and other authors demonstrated that Hsf2-/- mice suffer brain abnormalities".

We agree with Referee #1 that this crucial point (Fig. 6A) needs to be better explained. We have previously reported brain abnormalities under non-stress conditions (Kallio 2002) and radial neuronal migration and positioning defects (Chang 2006) in *Hsf2*$^{-/-}$ mice. In order to distinguish between the effects of the presence or absence of HSF2 under control conditions and the role of HSF2 in response to fetal alcohol exposure, we took the following steps. First, we chose a mixed C57Bl/6N x C57Bl/6J genetic background for our mice, in which the effects of *Hsf2* gene inactivation on neuronal migration under control conditions are less severe than in the pure C57Bl/6N strain used previously. As illustrated by the raw data plotted in **Supplementary Fig S11A**, the abnormal distribution of cortical neurons in *Hsf2*$^{-/-}$ mice in this background is slight but not significant.

Secondly, under control conditions, *Hsf2* gene inactivation clearly affects the number of BrdU-positive cells, suggesting proliferation defects (compare black bars in KO with black bars in WT mice; **Supplementary Fig S11A**), as observed in our previous study on the C57Bl/6N background (Chang 2006). Notably, this proliferative effect is not worsened by fetal alcohol exposure (compare black bars and white bars in the *Hsf2*$^{-/-}$ context). In order to avoid confusing the reader with these two phenomena, we chose to normalize all data to control levels for a given genotype, set at 1.

Thanks to these two measures, our main conclusion becomes clearer: the effects of fetal alcohol exposure on the distribution of neurons between deep (IV-VI) and superficial cortical layers (I-III) are less severe in *Hsf2*$^{-/-}$ mice than in wild-type mice.

To meet Referee #1's requirement, we have added our raw data to **Supplementary Fig S11 A,** and the two explanations above to the *Results* section. In addition, as suggested by Referee #2, we have added images from these neuronal positioning experiments in **Figure 6B**, to illustrate our non-normalized observations. To further clarify our message, we have also deleted the terms "beneficial" and "detrimental" from most of the text, or explained them in a more detailed manner in order to avoid any confusion with the detrimental effects of the loss of HSF2 under control conditions.

#1.2- HSF1 expression during Corticogenesis. "-In the results section the authors claim that ¨HSF1 was expressed at each stage of corticogenesis¨ but the results showed in Figure 1A only showed HSF1 protein expression in the telencephalon for E10.5 stage and no expression for E15.5, neither in telencephalon nor in forelimb. How do the authors explain that HSF1 is visualized ubiquitiously in the developing brain in figure 2B (right panel) at E16.5 stage but it is not observed by western blot in figure 1A?

We have provided a better quality western blot (WB) showing that HSF1 is expressed during corticogenesis, between E13.5 and E18.5. In addition, a paper recently accepted in *Neuron* by Hashimoto-Torii *et al*. (Rakic laboratory) - of which we are co-authors - describes the role of HSF1 in corticogenesis in response to various environmental insults (including alcohol). We have therefore deleted these data from Fig. 1, but transferred HSF1 expression profiles to **supplementary Fig S1B and C**. The lanes for the E13 stage of the former WB (in which the HSF1 signal was clearly observed) can now be found in **supplementary Fig S10D** in order to illustrate the point that the HSF1/HSF2 ratio is different in the developing brain than in other organs, as mentioned in the discussion.

#1.3-Nuclear versus cytoplasmic localization of HSF1 after CAI. -"The fact that CAI induced nuclear localization in the cortical plate is not clear from the pictures showed in figure 1F. The authors may considere performing nuclear and cytoplasm fractionation from cortex samples and analyze HSF1 protein levels by western blot."

We have replaced the original images with clearer high-magnification images. Nuclear/cytoplasmic fractionation experiments are usually very difficult to do in tissues because of frequent leaking of some nuclear material in the cytoplasm. This is the case for the developing cortex, because cortical nuclei are very fragile. Since HSF1 is rapidly shuttled between the cytoplasm and the nucleus, its increased nuclear localization is only one indication of its activation. Therefore, we also used the phosphorylation of HSF1 on Ser326, a *bona fide* hallmark of its activation. These data are now shown in **supplementary Fig S2**.

Note that in **Figure 3**, we provide additional evidence about the post-translational status of HSF1 following its activation by alcohol (sumoylation and phosphorylation on residues Ser303/307).

#1.4- The expression of Nde1 does not change as a consequence of CAI treatment. -In the introduction the authors state that ¨HSF2 controls the expression of NdeI, Dcx, and Dclk1...¨. However the results shown in figures 2C and 6B do not support this statement for the NdeI gene. The expression of NdeI, in figure 2C, does not change as a consequence of CAI treatment and the purported HSF1-HSF2 heterotrimer formation. Additionally, the expression obtained for NdeI in Hsf2+/+ control conditions in figure 6B does not correlate with results shown in 2C.

Indeed, in contrast to *p35*, *Dcx*, and *Dclk1*, the expression of *Nde1* is not changed by fetal alcohol exposure in a significant manner (**supplementary FigS11B**). While we showed these data in the previous version of the manuscript to provide an example of a gene bound by but not modified by HSF2, we have now removed *Nde1* to clarify our message, and added it to **supplementary FigS11B** along with other genes involved in radial neuronal migration whose expression is not affected by fetal alcohol exposure. Note that we also use this gene for our demonstration in favour of HSF1-HSF2 heterotrimers, because its HSE can bind only one trimer (see our working model in **Figure 4A** and **Supplementary Figure S8C**)

#1.5 – a) The genes analyzed by ChIP in figure 2B do not show a characteristic pattern to demonstrate that CAI affects HSF2 gene expression targets. NdeI and Dclk1 show similar HSF1-HSF2 promoter binding profile in control and CAI conditions, but different expression changes analyzed by RT-qPCR.

The purpose of Figure 2B is to show that HSF2 binding, which occurs on HSEs in control situations, is disturbed by CAI. There are two different outcomes in the analysis of the genes presented here. Either CAI provokes the loss of HSF2 binding (and therefore the loss of HSF1 binding as well because HSF2 is necessary for HSF1 binding; see Figure 5 in our manuscript) as in the case of *p35*; or it promotes the binding of HSF1-HSF2 complexes (like on *Dclk1* HSE). As mentioned in point **1.4c**, the consequence of this binding can be either an increase or a decrease in transcription, even for the same pattern of binding. Such a situation has been already described for the classic HSF target genes, the *Hsps,* in response to heat shock: the binding of HSF2 concomitantly with HSF1 results in an increase in the transcription of *Hsp70* but not of *Hsp40* or *Hsp110* (Östling 2007). Thus, the binding of both HSF1 and HSF2 does not always alter transcription in the same way. In the case of *p35*, as mentioned in our manuscript, it is interesting to note that the loss of HSF2 binding after CAI (and absence of HSF1 binding; see Figure 5) results in a reduction in transcription similar to that observed in *Hsf2*$^{-/-}$ cortices.
See also **point #1.4** regarding *Nde1*; this is an example of a gene in which the binding of both HSF1 and HSF2 after CAI does not significantly alter its expression, suggesting that these are not the major factors regulating its expression, although they bind to HSEs; other transcription factors may help to compensate for these binding modifications. This is in line

with studies in *Drosophila* that show that only 10% of the genes associated with HSF binding sites display changes in transcription after heat shock (Gonsalves *et al*, 2011). In addition, it is known from ChIPSeq analyses that binding of HSF to HSEs can occur with or without changes in expression, given the target gene considered (Vihervaara et al, 2013).

These effects could depend on the HSE environment in terms of other transcription factors (that may or may not compensate for the lack of HSF2), on chromatin status, etc., and are now discussed (**Results, pages 8-9**; **Discussion pages 21-22**, **and 23** for the chromatin environment).

#1.5 – b) Additionally, the expression obtained for NdeI in Hsf2+/+ control conditions in figure 6B does not correlate with results shown in 2C.

The EtOH/CTR ratio for *Nde1* transcripts was very close to 1 in both previous Fig 2C and Fig 6B. The slight difference between the ratios in WT cortices in Fig. 2C and Fig. 6B is due to the fact that cortices were analyzed at E16.5 in Fig. 2C and at E17.5 in Fig. 6B. Nevertheless, it is important to note that, globally, the EtOH/CTR ratio of transcript levels in WT cortices was extremely similar between E16.5 and E17.5 for all the genes analyzed in the previous Figs. 2C and 6B (*p35*, *Dcx*, and *Dclk*1), supporting the robustness of our data. We have however deleted *Nde1* from the new **Fig 2A and B** and transferred it to **supplementary Fig S11B**, as mentioned in point **#1.4**.

#1.5 – c) In addition, Dclk1 and p35 showed similar mRNA decreases after CAI treatment but there is no correlation with HSF1-HSF2 promoter-binding. "-The genes analyzed by ChIP in figure 2B do not show a characteristic pattern to demonstrate that CAI affects HSF2 gene expression targets. NdeI and Dclk1 show similar HSF1-HSF2 promoter binding profile in control and CAI conditions, but different expression changes analyzed by RT-qPCR. In addition, Dclk1 and p35 showed similar mRNA decreases after CAI treatment but there is no correlation with HSF1-HSF2 promoter-binding ».

Much of the explanation for these seemingly contradictory results is provided in point **#1.5a** above. This aspect of gene activation vs. repression by HSFs has been often overlooked due to the historical focus on the activation of heat shock genes by HSF1. However, HSF2 can affect target gene transcription upon heat shock either positively or negatively (Östling 2007), likely through the formation of HSF1-HSF2 heterotrimers (which are distinct from the heterotrimers induced by ethanol). In addition, HSF1 has also recently been shown to repress transcription, as illustrated by its amazing ability to completely rewire the transcriptome in cancer (Mendillo 2012; Santagata 2013). The unusual features of HSF1 activation by ethanol in terms of low transcriptional abilities might also account for the differential (positive or negative) impact that HSF1/HSF2 might have on gene transcription. We now mention these aspects in the ***Discussion*** (**pages 21-23**).

#1.5 – d) The authors might analyze a higher number of HSF2 gene targets to demonstrate that ethanol exposure indeed affects HSF1-HSF2 promoter co-localization and that alters the expression levels of HSF2 gene targets. Does Dcx gene have an HSE sequence? Why is this gene not included in ChIP experiments?

We completely agree with the Referee.
- We now provide ChIP data for *Dcx* in **Fig 2A**, right panel. Since we used both cortical material already tested in the left panel as well as other samples, we plotted the data for Dcx in a separate graph.
- We have analyzed 14 additional genes belonging to the *Dcx* family (*Dclk2*), the Cdk5 pathway (*p39* and *Cdk5rap2*) and the *Nde1*/*Lis1* pathway and their interactors (*Ndel1*, *Lis1*, *NudC*, and *Dics1*).
In addition, we explored unbiased ChIPSeq analyses from Mendillo *et al* (2012) and

Vihervaara *et al* (2013), which identified genes targeted by HSF1 and/or HSF2, for genes involved in radial neuronal migration. Using this approach, we identified *Rttn* (encoding Rotatin), *Myo10* (Myosin 10 isoforms), *MapT* (tau), and *Mark2* (*par-1*, encoding a kinase) as targeted by HSFs.

Among these, the expression of *Myo10*, *MapT* and *Mark2* was found to be significantly affected by alcohol in an HSF2-dependent manner (**Fig 6C**). The expression of the other genes was not markedly affected by alcohol, and interestingly their expression not dependent on HSF2 in alcohol conditions, reinforcing the conclusion that HSF2 is a major mediator of disturbances in the expression of genes affected by alcohol in an HSF2-dependent manner.

Note that for these additional genes, we focused to E17 for RT-qPCR analyses, a window during which most of genes were expected to be expressed and/or be dependent on HSF2 (for example, the dependency of p39 on HSF2 under control conditions is greater at E17 than at E16 in our hands (Chang et al, 2006)).

#1.6- The authors may also consider analyzing the effect of Heat Shock treatment by ChIP experiments on the neuronal migration genes to demonstrate that alteration in HSF1-HSF2 promoter-binding is specific for CAI.

We have tried this experiment many times in the past using different temperatures, durations of stress, medium in which cortices were heat-shocked *ex vivo* etc., without success. E15.5-E17.5 cortices are extremely sensitive to heat shock and rapidly damaged. As illustrated by the EMSA in **Figure #1.6**, complexes observed after heat shock in E16.5 cortices are “smeared” (blue line) and not supershifted by antibodies to either HSF1 or HSF2, unlike those from iMEFs or E16.5 control cortices, suggesting that they do not correspond to typical HSF-HSE complexes detected after heat shock or alcohol, but rather to HSF-HSE complexes altered by protein degradation.

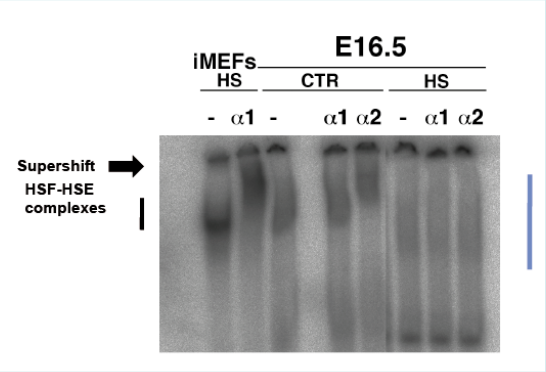

Figure #1.6: Gel-shift assay of HSF1 and HSF2 DNA-binding activity in iMEFs or E16.5 cortices under control or heat shock conditions (42°C, 30 min). The black bar indicates the position of HSE-HSF complexes in heat-shocked iMEFs (positive controls) or control E16.5 cortices. The presence of HSF1 or HSF2 in the HSF-HSE complex (HSF-HSE) was assessed by supershifting (black arrow) in the presence of anti-HSF1 (α1) or anti-HSF2 antibodies (α2). The blue line points to the “smeared” complexes observed in heat-shocked E16.5 cortices. In E16.5 CTR cortices, the typical constitutive HSE-DNA binding activity of HSF2 was observed (supershifting with anti-HSF2 antibody), whereas, as expected, HSF1 did not display any constitutive DNA-binding activity under control conditions.

Note that E10.5-E12.5 cortices are not as sensitive to *ex vivo* heat shock as are E16.5 cortices. This could be seen in our previous Fig. 1C, where HSF1 was clearly activated by heat shock (and was HSF2 inactivated). In order to lighten our manuscript, we deleted these data from the revised version, because we considered that it did not bring much to our argumentation. These stages are not relevant to address this question, because HSF2 does not influence radial neuronal migration at these stages (Chang et al., 2006).

It should be noted that we cannot subject pregnant mice to temperatures high enough to reach the classic 42-45°C temperature range known to activate HSF1 *ex vivo,* in fetuses, because the mothers would die. Applying temperatures in the 39-41°C fever range, although more feasible in pregnant mothers, is known to activate only HSF2 and not HSF1 *ex vivo* (Shinkawa 2011), which would not be representative of the classic Heat Shock Response.

Besides, we do not claim that heat shock cannot modulate these genes in an HSF-dependent manner. Indeed, heat shock has also been shown *ex vivo* by ChIP to involve – transiently – the binding of HSF1 and HSF2 to the *Hsp* gene (Östling 2007; Ahlskog 2010), likely through the formation of HSF1-HSF2 heterotrimers (Sandqvist 2009). We have also demonstrated the concomitant occupancy by HSF1 and HSF2 of HSEs of the *Hsp70* gene upon heat shock and ethanol exposure in iMEFs (see Suppl. Fig. S2C). Notably, *p35* (also called *Cdk5R1*) is bound by HSF1 after heat shock in cancer HME cell models (Mendillo 2012), supporting the stress-responsive binding of the *p35* regulatory region by HSF1. See our point **#1.1** concerning the novelty of our findings with regard to these heterotrimers.

#1.7-The use of Neuro2A as a model for the experiments is based on the statement that ¨this cell line displayed constitutive HSF2-binding activity in control conditions, similar to fetal cortices¨. However, results shown in Figure 3A for N2A under control conditions do not show constitutive HSF2-binding activity as it is observed for fetal cortices in figures 1C and 1E. (see also **Referee#3**)

In previous Figure 3A, now **Fig S6A**), the exposure time was chosen to illustrate the induction of HSF1 and HSF2 DNA-binding activity upon ethanol exposure, and the fact that the intensity of the complex induced by ethanol was lower than that induced by heat shock. We deleted the first (redundant) 3 N2A C (control) lanes, in which the HSF2 constitutive DNA-binding activity was not easily observable, in order to focus attention on the other N2A C lanes (**lanes 4-6** in the new Fig S6A). In addition, as shown in **Fig. 5A**, control N2A cells display constitutive HSF2 DNA-binding activity, as does the developing cortex (although in lesser extend), and also display constitutive HSF1 DNA-binding activity, as is often the case for cell lines of tumoural origin.

Over the past few years, we have tried to check our hypothesis in other models, including neurosphere cultures of cortical neural progenitors or primary cortical neurons, in collaboration with Dr. Véronique Dubreuil and Prof. Jan Sap of our Unit, who are experts in the field. However, in each case, the levels of HSF1 and HSF2 rapidly decreased within 2-3 days in culture (see **Figure #1.7b**), making them inappropriate models for the developing mouse cortex.

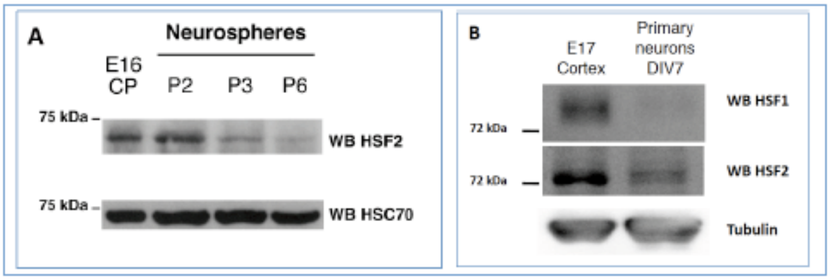

Figure #1.7b. **A.** Neural progenitors were isolated from E16.5 cortices and grown as neurospheres for 2 to 6 passages (P2 to P6) and HSF2 protein levels analyzed by western blotting (Chojnacki 2008). **B.** Primary neurons were isolated from E17 cortices (Beaudoin 2012) and cultivated up to 7 days *ex vivo*, at which time they start to display typical neuronal morphology and characteristics.

Note that Pignataro et al. (2007) used postnatal rat primary cortical neurons. However, in our hands, HSF2 is not active anymore and expressed at very low levels in these neurons. We have also tested other neural cell lines, but so far have not found any with constitutive HSF2 binding activity but little or no constitutive HSF1 activity.

We must specify that we only used N2A to confirm what we had already shown in the fetal alcohol-exposed cortex or when transfection experiments were needed. In addition, while the formation of HSF1/HSF2 heterotrimers in fetal cortices is observed after alcohol exposure, in N2A cells, the constitutive HSF1 and HSF2 activation likely leads to the formation of heterotrimers even under control conditions (**supplementary Fig S6 A and B**). However, ethanol exposure clearly increases the formation of heterotrimers and the activity of HSF1 and HSF2 in N2A cells, which is what we were hoping to show. Ethanol also triggers

HSF1 and HSF2 activation in iMEFs, in which HSF1 and HSF2 DNA-binding activities are very low (**supplementary Fig S2**), and in F9 cells, which have high levels of HSF2 DNA-binding activity but are not a neural cell model (Rallu 1997; **supplementary Fig. S5**).

#1.8 -a) The ethanol treatment seems to decrease HSF1 acetylation levels compared to HS conditions, a factor that is associated with HSF1 repression, and incubation with Histone deacetylases inhibitors restored HSF1 acetylation levels after EtOH treatment. The authors may consider analyzing HDAC mRNA or protein levels in their experimental conditions to better correlate their findings using TSA. In addition, they might consider the work published by Agudelo et al., 2011 (doi: 10.1111/j.1530-0277.2011.01492.x.) that demonstrates that HDAC2 is upregulated by EtOH using
neuronal cell lines. The authors might also consider that alteration in HDAC activities and protein
acetylation levels can also provoke chromatin remodeling alterations as it has been previously published by Padey et al., 2008 (Brain chromatin remodeling: a novel mechanism of alcoholism).
They might consider the possibility that chromatin remodeling can be the main factor of HSF1-HSF2 DNA-binding activity alteration under EtOH exposure and that can explain changes in gene expression of neural migration genes.

We agree with Referee #1 and had already thought about this aspect, based on the report on HDAC2 by Agudelo et al. (2011). In addition, in the liver, alcohol associated with fatty-acid-rich diets modifies SIRT1 mRNA levels (Oliva 2008; Lieber 2008; You 2008; Liang 2011), while HSF1 is regulated by SIRT1 (Westerheide 2009).

We thus investigated HDAC1, HDAC2, and SIRT1 protein levels following exposure to 0.5% ethanol in N2A cells, and the effect of their inhibition on HSF1 acetylation. These data have been added to **supplementary Figure S6 D**), and suggest that alcohol-induced modifications in HDAC levels (including SIRT1) might, at least transiently, contribute to maintaining HSF1 in a deacetylated state. However, there are also other explanations for the lack of HSF1 acetylation upon longer ethanol exposure, for example, the long-term association of HSF1 and HSF2 within alcohol-induced heterotrimers. This hypothesis needs to be considered because most post-translational modifications of HSF1 (classically induced by heat shock) are not or only weakly triggered by alcohol (**Fig 3**).

We hope that we have answered the Referee's questions, but the subject is not simple and will likely require deeper investigations that are, in our opinion, beyond the scope of this manuscript.

#1.8 -b) The authors might also consider that alteration in HDAC activities and protein acetylation levels can also provoke chromatin remodeling alterations as it has been previously published by Padey et al., 2008 (Brain chromatin remodeling: a novel mechanism of alcoholism). They might consider the possibility that chromatin remodeling can be the main factor of HSF1-HSF2 DNA-binding activity alteration under EtOH exposure and that can explain changes in gene expression of neural migration genes.

We completely agree with Referee #1, and we are currently exploring the mechanism by which HSF2 acts in the normal and pathological (alcohol-exposed) brain in collaboration with Lea Sistonen (Turku, Finland). Indeed, HSF2 has only a weak ability to modulate transcription by itself and likely acts by recruiting chromatin remodelers and histone modifiers, depending on the stress (heat shock, mild heat shock, ethanol exposure…) and the target gene considered. Since this study is still in progress and in collaboration with another group, we cannot present these data here, but have mentioned this possibility in the *Discussion* (**page 20 and paragraph 2**). We have also added a paragraph to explain how modifications in HSF2 binding by alcohol could recruit chromatin remodelers and modify the chromatin environment, in line with a recent report in the literature (Vihervaara *et al*, 2013; *Discussion* **page 23 and first paragraph)**.

#1.9- The authors claim that sumoylation modification is prolonged under ethanol exposure compared to HS conditions. They authors might remove this conclusion that is not supported by the data presented

Actually, this can be seen in the experiment in HeLa cells showing nuclear stress bodies (nSBs, **supplementary Fig S7**), but we agree that prolonged sumoylation is not observed in **Fig 3E** at 6 hours. As suggested by Referee #1, we have deleted this conclusion and just mentioned the fact that the induction of HSF1 sumoylation is reduced/delayed in cells exposed to ethanol, as compared to heat shock.

#1.10- In figure 5D, the authors analyze the effect of Hsf2-/- in mice under intraperitoneal injection of EtHO and the HSF1-HSF2 DNA-binding activity. However, Hsf2+/+ control does not show HSF1 DNA-binding, as previously shown for CAI. In this case it is not possible to correlate that the loss of binding of HSF1 is due to the absence of HSF2 when HSF1 is not binding under EtOH treatment in Hsf2+/+.

Figure 5D compares the binding activity of HSF1 and HSF2 after ethanol exposure in *Hsf2*$^{+/+}$ (embryos ε1 and ε2) and *Hsf2*$^{-/-}$ embryos (embryos ε3 and ε4). In the *Hsf2*$^{+/+}$ embryos, both HSF1 and HSF2 binding activities were detected by EMSA upon CAI, as shown in **Fig 1**. In the *Hsf2*$^{-/-}$ embryos, no HSF binding activity could be detected. In **Fig 5E** (graph quantifying the signal intensity of the complex in Figure 5D), we realized that there was an error in the position of the curve corresponding to the supershift induced by the anti-HSF1 antibody in WT embryos and have corrected it.

#1.11- They authors might perform ChIP experiments in Hsf2-/- mice under ethanol conditions for HSF1, to analyze if the neuronal migration genes NdeI, Dcx, Dclk1 and p35 are regulated entirely by HSF1 in the absence of HSF2.

Actually, based on the EMSA experiment in *Hsf2*$^{-/-}$ cortices, we would expect virtually no alcohol-induced activation of HSF1 in the absence of HSF2 (see **Fig 5D**). Consequently, in ChIP experiments, these genes would not be bound by either HSF1 or HSF2, although there could be some residual HSF1 activity.

We tried to collect enough material to answer this point, even though it was deemphasized by Referee #3. Nevertheless, due to the hypofertility of *Hsf2*$^{-/-}$ females and mortality of some *Hsf2*$^{-/-}$ fetuses (Kallio 2002), it was not possible to fulfil this requirement on time, and all collected *Hsf2*$^{-/-}$ material was dedicated to RT-qPCR of the 14 additional genes that we identified as putative target genes. Indeed, RT-qPCR experiments can be performed on individual fetal cortices, whereas ChIP experiments require at least 3-4 cortices per point.

Minor comments:
-In the results section the authors claim that ¨CAI caused the loss of HSF2 binding and HSF1 binding to the p35 HSE...¨. However, results shown in figure 2C only demonstrated that HSF2 losses binding after CAI but HSF1 never binds in control or CAI conditions. Similarly, in the introduction section the authors state that ¨the modification of HSF1 and 2 activities disturb the expression of p35¨. However, I would say that only HSF2 activity alteration disturbs p35 but not HSF1 because this protein does not seem to regulate p35.

We understand the Referee's concern. In the case of *p35*, it is indeed the disturbance of HSF2 binding (and not the binding of HSF1-HSF2 trimers) that perturbs p35 expression. We have modified the text accordingly.

-Figure 3D bottom panel says VPA instead of TSA.

This error was fixed in what is now **Fig 3B**.

- Using a HSE probe that could bind only one trimer and gel shift assays are not direct experiments
to state that HSF1-HSF2 form heterotrimers. It can be a possibility that HSF1 and HSF2 homotrimers can independently bind to different molecules of the HSE in the same sample or one
protein off the DNA can interact with the other protein bound to DNA. They authors should explain
this.

As explained in the text, if the HSF-HSE band were composed of a mixture of homotrimers, then supershifting of the entire band by either anti-HSF1 or anti-HSF2 antibody would not occur. Because complete supershifting did occur with any one antibody, we concluded that this was due to the formation of heterotrimers. This conclusion is also supported by EGS cross-linking experiments and ChIP experiments using an HSE that can only accept one trimer (like the *Nde1* HSE).

-Figure 5D legend must explain what is e1,e2,e3,e4.

Indeed, these symbols correspond to individual embryos, now added to the legend.

-Figure 6C is not commented in the text.

This error has been fixed.

Referee #2

#2.1. In the introduction the authors do not refer to HSF4.

We now mention that we focused on HSF1 and 2, since HSF4 does not display any activity in the developing brain, citing our Review on this point (Abane & Mezger, 2010). Neither we nor other groups have observed HSF4 in the prenatal brain, although this is mentioned *per se* in the literature.

#2.2. Fig. 1 shows only a histogram of the neuronal positioning by BrdU. Neuronal migration is one of the major issues of this manuscript, and appears in the title. At least representative images of the migration phenotype should be presented (this comment relates also to Figure 6).

We thank the Referee for this useful comment. We now provide images of the corresponding cortices in **Fig 1A**, supplementary **Fig S1A**, and **Fig 6B**.

#2.3. Fig. 1A, shows a western blot, however the control cells do not seem to express HSF2 alpha. The control cells are F9 cells that express both isoforms (alpha and beta), but beta is much more expressed that alpha.

We have replaced this image by a more representative one, and transferred the data to **supplementary Fig S1B** (see point #**1.2**). They serve to discuss the use of HSF2 α and HSF2 β in the rescue experiments in **supplementary Fig S10 C**.

#2.4. Fig. 1F, a higher magnification may allow to visualize better the differences.

We completely agree. This has been added to supplementary **Fig S2A** and **B**. In addition, we have performed fractionation experiments as suggested by Referee #1 and added the corresponding data to Figure S2C (please also see point **#1.3** for more details).

#2.5. Fig. 2B, the related test should be somewhat modified; no difference in c/ca1 of nde1 HSF2 and there is a reduction in HSF2 in Dclk1 c/ca1. It should be useful to add ChIP for Hsp70.

- We would like to underline the fact that HSF2 binding on the *Dclk1* HSE was not significantly different between control and CAI conditions (indeed, the error bars widely overlap), as is the case with *Nde1*, as stated in the text. The slight differences in HSF2 binding upon CAI to *Nde1* and *Dclk1* might be due to a different chromatin environment in the two cases, a difference in the composition of HSF1-HSF2 heterotrimers binding to the two HSEs, or other unknown factors.
The main point that we wish to make here is that HSF2 is bound to the HSE of each of these two genes both under control and CAI conditions.
- We have added ChIP data for the *Hsp70* promoter, performed on the same samples (see Figure 1H). It is important to note that HSF2 does not significantly bind *Hsp70* HSEs under control conditions. In contrast, it binds constitutively to *MAP* HSEs. However, a marked increase in the binding of both HSF1 and HSF2 on *Hsp70* is observed upon CAI; moreover, HSF2 binding at this site is comparable to that observed for *Dclk1* (see **Figure #2.5**).

#2.6. Fig. 2C: The changes in mRNA levels of Dcx, Dclk, Nde1 following fetal alcohol exposure are presented and are very interesting. The confirmation on the protein level will support the hypothesis that migration defect in FAE is mediated by reduced transcription of these genes.

We show in **supplementary Fig S4C** that in agreement with the RT-qPCR data, p35 protein is reduced in fetal cortices upon CAI.
We investigated some of the members of DCX/DCLK1 family in western blots, which are difficult to distinguish because of their numerous isoforms and the cross-reactivity of some antibodies. There seems to be interesting differential effects on some variants that could be due to impact on alternative splicing. This complexity deserves deeper analyses, which is beyond the scope of this study.

Referee #3

#3.1. Overall, the manuscript is a very difficult read because it is packed with data. At some point it tends to slip into being excessively confirmatory of previous findings. The six figures are unusually packed, the panels are not referenced in their order of appearance, and often too small to see details. Therefore, it would be helpful for the reader to only show what really is important in the main figures, and then drop some of the more auxiliary data into supporting information. Perhaps a new figure should be generated for the first half of Fig. 1, which Referee 1 clearly, and rightfully, has criticized.

We have shortened and completely rewritten the manuscript. We have also simplified the figures, especially **Fig 1** and **Fig 3**, moved auxiliary data to the **Expanded View** and **supplementary Figures** and split our previous **Fig 6 into Fig 6 and Fig 7**.

Referee 1's points **#1, #3, #4, #7**, **#8**, **#10** are most critical and must be followed up. For #7 (N2A cell line), primary cortical neurons or similar positive controls must be introduced.

Point **#1.** We think we have satisfied these requirements, except for ChIP in *Hsf2*$^{-/-}$ samples, for the reasons given in point **#1.11**. We hope that this will not be a problem, given the fact that Referee #3 has deemphasized this point (see below).
Point **#1.3**. We provide nuclear/cytoplasmic fractionation data, which is always technically very difficult in tissues, especially the developing cortex, by showing enrichment of the active (Ser326-phosphorylated) HSF1 form in the nucleus.
Point **#1.4**. We provide an explanation for the observation that loss of HSF binding and

binding of HSF1-HSF2 heterotrimers can both result in the downregulation in gene expression, based on ChIPSeq data analyses in the literature. These data also point out genes bound by HSFs whose expression is not altered by HSF knockout.
Point **#1.7**. We provide data showing that cultures of neuronal progenitors or primary cortical neurons are unfortunately not good models for the study of the role of HSF2. As a consequence, N2A neuroblastoma cells are for the moment the best cell model that is available for our study.
Point **#1.8**. We followed the advice of Referee 1 and have mentioned the possible role of HSF2 on the chromatin environment in the discussion.
Point **#1.10**. We have corrected the text and graph according to Referee 1's requirement.

Point #2: is due to having a too small figure and overloading the positive control, which rendered the really important lanes partly obscure.

We have performed new western blot experiments to correct this. The data have been moved to **Figure S1B** for the reasons given in point **#1.2.**

Point #5: is again very credible and needs to be dealt with, particularly in relation to the Dcx question.

Please see our detailed response in point **#1.5**.

Point #6: (HSE sequences) comment is entirely fair, which I think anyone non-familiar with these interactions would have problem with. Perhaps explanation of how relaxed these consensus sequences can be, and how many different types there are should be mentioned in the text.

Our data suggest that the position of the HSE (in the vicinity of the regulatory region versus gene body) in the gene might be more relevant than the HSE sequence itself, possibly due to the chromatin environment. We have now mentioned the possible effects of HSE sequence homology and position in the discussion, and provided these informations for more genes in **supplementary Fig S3** (see *Discussion* **page 22**).

Point #9: The SUMOylation data needs to be rearranged and the text clarified such that it becomes a readable piece. This is one of the key components of the paper but seems to be crammed into a few panels, which are small, of relatively low quality (on the PDF), and less informative than they should be. The Referee's comment certainly must be followed.

We have rectified the text accordingly.

Point #11 might be of less importance if the above concerns are exhaustively addressed.

We are very grateful for this Referee's advice and think we have answered most of his/her questions. We have concentrated on identifying new target genes involved in neuronal migration and affected by alcohol in an HSF2-dependent manner by performing RT-qPCR for 14 new genes identified as binding HSF1 or HSF2. We identified the position of their HSEs by bioinformatic analyses and validated HSF2 binding status before and after CAI for two genes (*Dcx* and Chl1) that were not found in ChIPSeq data. Due to limited materials, we could not perform ChIP on *Hsf2*$^{-/-}$ (see point **#1.11**).

For Referee 2, I agree with her/his critical point (#2) on the lack of representative images in Figures 1 and 6, which gives the lingering impression of substandard data quality. This certainly should be addressed, and accommodated.

We have provided representative images as requested.

Points #3, #4, #5 and #7 will need to be dealt with. I would perhaps deemphasize #6, except the query about the statistical significance, which I think is erroneously reported. The other queries are self-explanatory.

Please see our detailed responses in points **#2.3**, **#2.4**, **#2.5** and **#2.6**. Other corrections have been made as requested.

CONCLUSION

We think that we answered most of the Referees' requirements and hope that the amended manuscript will meet their expectations and qualify for publication in *EMBO Molecular Medicine.*

REFERENCES

- Abane R*, Ahlskog J*, Bergman H, Saberan-Djoneidi D, Henry, M, Westerheide S, Sistonen L§, Mezger V.§ HSF2 acetylation interferes with its ubiquitination and contributes to regulate its turnover during stress. In preparation. * or §, equal contributions. (CNRS PICS programme)

- Ahlskog JK, Björk JK, Elsing AN, Aspelin C, Kallio M, Roos-Mattjus P, Sistonen L. Anaphase-promoting complex/cyclosome participates in the acute response to protein-damaging stress Mol Cell Biol 2010. 30(24):5608-20. PMID: 20937767

- Agudelo M, Gandhi N, Saiyed Z, Pichili V, Thangavel S, Khatavkar P, Yndart-Arias A, Nair M. Effects of alcohol on histone deacetylase 2 (HDAC2) and the neuroprotective role of trichostatin A (TSA). Alcohol Clin Exp Res 2011. 35(8):1550-6. PMID: 21447001

- Beaudoin GM 3rd, Lee SH, Singh D, Yuan Y, Ng YG, Reichardt LF, Arikkath J. Culturing pyramidal neurons from the early postnatal mouse hippocampus and cortex. Nat Protoc 2012. 7(9):1741-54. PMID: 22936216

- Cahana A, Escamez T, Nowakowski RS, Hayes NL, Giacobini M, von Holst A, Shmueli O, Sapir T, McConnell SK, Wurst W, Martinez S, Reiner O. Targeted mutagenesis of Lis1 disrupts cortical development and LIS1 homodimerization. Proc Natl Acad Sci U S A 2001. 98(11):6429-34. PMID: 11344260

- Chang Y, Ostling P, Akerfelt M, Trouillet D, Rallu M, … Morange M, Sistonen L, Mezger V. Role of Heat Shock Factor 2 in cerebral cortex formation and as regulator of p35 expression. Genes Dev 2006. 20:836-47. PMID: 16600913

- Chojnacki AK, Weiss S. Production of neurons, astrocytes and oligodendrocytes from mammalian CNS stem cells. Nat Protoc. 2008. 3(6):935-40. PMID: 18536641

- Coquelle FM, Levy T, Bergmann S, Wolf SG, Bar-El D, Sapir T, Brody Y, Orr I, Barkai N, Eichele G, Reiner O. Common and divergent roles for members of the mouse DCX superfamily. Cell Cycle 2006. 5(9):976-83. PMID: 16628014

- Demyanenko GP, Schachner M, Anton E, Schmid R, Feng G, Sanes J, Maness PF. Close homolog of L1 modulates area-specific neuronal positioning and dendrite orientation in the cerebral cortex. Neuron 2004. 44(3):423-37. PMID: 15504324

- Fujimoto M, Oshima K, Shinkawa T, Wang BB, Inouye S, Hayashida N, Takii R, Nakai A. Analysis of HSF4 binding regions reveals its necessity for gene regulation during development and heat shock response in mouse lenses. J Biol Chem 2008. 283:29961-70. PMID: 18755693

- Gonsalves SE, Moses AM, Razak Z, Robert F, Westwood JT (2011) Whole-genome analysis reveals that active heat shock factor binding sites are mostly associated with non-heat shock genes in Drosophila melanogaster. PLoS One 6:e15934. PMID: 21264254

- Hirotsune S, Fleck MW, Gambello MJ, Bix GJ, Chen A, Clark GD, Ledbetter DH, McBain CJ, Wynshaw-Boris A. Graded reduction of Pafah1b1 (Lis1) activity results in neuronal migration defects and early embryonic lethality. Nat Genet 1998. 19(4):333-9. PMID: 9697693

- Kallio M, Chang Y, Manuel M, Alastalo TP, Rallu M, Gitton Y, Pirkkala L, Loones MT, Paslaru L, Larney S, Hiard S, Morange M, Sistonen L, Mezger V. Brain abnormalities, defective meiotic chromosome synapsis and female subfertility in HSF2 null mice. EMBO J 2002. 21:2591-601. PMID: 12032072

- Kerjan G, Koizumi H, Han EB, Dubé CM, Djakovic SN, Patrick GN, Baram TZ, Heinemann SF, Gleeson JG. Mice lacking doublecortin and doublecortin-like kinase 2 display altered hippocampal neuronal maturation and spontaneous seizures.Proc Natl Acad Sci U S A 2009. 106(16):6766-71. PMID: 19342486

- Kline MP, Morimoto RI. Repression of the heat shock factor 1 transcriptional activation domain is modulated by constitutive phosphorylation. Mol Cell Biol 1997. 17(4):2107-15. PMID: 9121459

- Ko J, Humbert S, Bronson RT, Takahashi S, Kulkarni AB, Li E, Tsai LH. p35 and p39 are essential for cyclin-dependent kinase 5 function during neurodevelopment. J Neurosci 2001. 21(17):6758-71. PMID: 11517264

- Le Masson F, Razak Z, Kaigo M, Audouard C, Charry C, Cooke H, Westwood JT, Christians ES. Identification of heat shock factor 1 molecular and cellular targets during embryonic and adult female meiosis. Mol Cell Biol 2011. 31(16):3410-23. PMID: 21690297

- Liang X, Hu M, Rogers CQ, Shen Z, You M. Role of SIRT1-FoxO1 signaling in dietary saturated fat-dependent upregulation of liver adiponectin receptor 2 in ethanol-administered mice. Antioxid Redox Signal 2011. 15(2):425-35. PMID: 21194380

- Lieber CS, Leo MA, Wang X, Decarli LM. Effect of chronic alcohol consumption on Hepatic SIRT1 and PGC-1alpha in rats. Biochem Biophys Res Commun 2008. 370(1):44-8.
PMID: 18342626

- Mendillo ML, Santagata S, Koeva M, Bell GW, Hu R, Tamimi RM, Fraenkel E, Ince TA, Whitesell L, Lindquist S. HSF1 drives a transcriptional program distinct from heat shock to support highly malignant human cancers. Cell 2012. 150:549-62. PMID: 22863008

- Oliva J, French BA, Li J, Bardag-Gorce F, Fu P, French SW. Sirt1 is involved in energy metabolism: the role of chronic ethanol feeding and resveratrol. Exp Mol Pathol. 2008 Dec;85(3):155-9. Erratum in: Exp Mol Pathol 2009. 87(1):87. PMID: 18793633

- Östling P, Björk JK, Roos-Mattjus P, Mezger V, Sistonen L. Heat shock factor 2 (HSF2) contributes to inducible expression of hsp genes through interplay with HSF1. J Biol Chem 2007. 282: 7077-86. PMID: 17213196

- Pascual M, Baliño P, Alfonso-Loeches S, Aragón CM, Guerri C. Impact of TLR4 on behavioral and cognitive dysfunctions associated with alcohol-induced neuroinflammatory damage. Brain Behav Immun 2011. Suppl 1:S80-91. PMID: 21352907

- Pignataro L, Miller AN, Ma L, Midha S, Protiva P, Herrera DG, Harrison NL. Alcohol regulates gene expression in neurons via activation of heat shock factor 1. J Neurosci 2007. 27(47):12957-66. PMID: 18032669

- Rallu M, Loones M, Lallemand Y, Morimoto R, Morange M, Mezger V. Function and regulation of heat shock factor 2 during mouse embryogenesis. Proc Natl Acad Sci U S A 1997. 94(6):2392-7. PMID: 9122205

- Santagata S, Mendillo ML, Tang YC, Subramanian A, Perley CC, Roche SP, Wong B, Narayan R, Kwon H, Koeva M, Amon A, Golub TR, Porco JA Jr, Whitesell L, Lindquist S. Tight coordination of protein translation and HSF1 activation supports the anabolic malignant state. Science 2013. 341(6143):1238303. PMID: 23869022

- Shinkawa T, Tan K, Fujimoto M, Hayashida N, Yamamoto K, Takaki E, Takii R, Prakasam R, Inouye S, Mezger V, Nakai A. Heat shock factor 2 is required for maintaining proteostasis against febrile-range thermal stress and polyglutamine aggregation. Mol Biol Cell 2011. 22:3571-83. PMID: 21813737

- Shu T, Ayala R, Nguyen MD, Xie Z, Gleeson JG, Tsai LH. Ndel1 operates in a common pathway with LIS1 and cytoplasmic dynein to regulate cortical neuronal positioning. Neuron 2004. 44(2):263-77. PMID: 15473966

- Tuy FP, Saillour Y, Kappeler C, Chelly J, Francis F. Alternative transcripts of Dclk1 and Dclk2 and their expression in doublecortin knockout mice. Dev Neurosci 2008. 30(1-3):171-86. PMID: 18075264

- Vihervaara A, Sergelius C, Vasara J, Blom MA, Elsing AN, Roos-Mattjus P, Sistonen L. Transcriptional response to stress in the dynamic chromatin environment of cycling and mitotic cells. Proc Natl Acad Sci U S A 2013. 110(36):E3388-97. PMID: 23959860

- Westerheide SD, Anckar J, Stevens SM Jr, Sistonen L, Morimoto RI. Stress-inducible regulation of heat shock factor 1 by the deacetylase SIRT1. Science 2009. 323:1063-6. PMID: 19229036

- You M, Cao Q, Liang X, Ajmo JM, Ness GC. Mammalian sirtuin 1 is involved in the protective action of dietary saturated fat against alcoholic fatty liver in mice. J Nutr 2008.138(3):497-501. PMID: 18287356

- You M, Liang X, Ajmo JM, Ness GC. Involvement of mammalian sirtuin 1 in the action of ethanol in the liver. Am J Physiol Gastrointest Liver Physiol 2008. 294(4):G892-8. PMID: 18239056

2nd Editorial Decision 14 April 2014

Thank you for the submission of your revised manuscript to EMBO Molecular Medicine. We have now received the enclosed reports from the referees that were asked to re-assess it. As you will see, the reviewers are now globally supportive and I am pleased to inform you that we will be able to accept your manuscript pending the following final amendments:

1) Figures 3, 4D, 5C,D,F (western blots) appear to have suffered too much contrast and light. I encourage you to reduce both in order to be able to identify the background.

We now encourage the publication of source data, particularly for electrophoretic gels and blots, with the aim of making primary data more accessible and transparent to the reader. Would you be willing to provide a PDF file per figure that contains the original, uncropped and unprocessed scans of all or key gels used in the figure? The PDF files should be labeled with the appropriate figure/panel number, and should have molecular weight markers; further annotation may be useful but is not essential. The PDF files will be published online with the article as supplementary "Source Data" files. If you have any questions regarding this just contact me.

I look forward to seeing a revised form of your manuscript as soon as possible.

***** Reviewer's comments *****

Referee #1 (Remarks):

The authors have satisfactorily addressed my primary concerns.

Referee #2 (Remarks):

The manuscript has undergone an extensive revision and the authors addressed most of the concerns in a suitable way.

2nd Revision - authors' response 01 June 2014

I have now uploaded all the new files corresponding to amended Figures, Supplemental Figures and Source data files and submitted the new version.

Please find below a description of the amendments made trying to meet your requirements.

*Figures: *

In *Figure 1A*, brackets were added for more clarity about the statistical process.

In *Figure 3*, the contrasts have been modified to allow better detection of the back ground process, or when the exposures allowed it, we came back to the raw files to work on it (ex: Figure 3C). Sometimes, (*i.e.* in Figure 3A), using Chemismart device, exposures with unsaturated signals, gave a very low background; see for example the corresponding raw data comparing two different exposures).

Note that in Figure 3C, we realized that the upper panel had been pasted in the inverted orientation. Because the gel was perfectly symmetrical, this did not change at all the conclusion of the study (namely that ethanol exposure does not provoked a shift in HSF1 migration (no HSF1 hyperphosphorylation)) as heat shock does (HSF1 hyperphosphorylation). We fixed this panel to stick to the raw data.

In *Figure 4*, the blot HSF2 CTR has been replaced by the original raw data to improve the background detection and lower the contrast problems.

In *Figure 5 C and F*, we tried to "push" the background to make it more detectable, but as can be seen from the raw data, the background was originally very low on the *Chemismart *exposures. For *Figure 5D* (EMSA), we also have started again from the raw picture to diminish contrast.

Supplemental Figures:

In Figure S1A, brackets were added for more clarity about the statistical process

In Figure S4, the loading control is actually "Actin" and not "Hsc70" as was first indicated. This was fixed on the Figure S4 (one of the good things of going through the raw data again).

In Figure S10 C, we added a bar to the WB (see the corresponding raw data).

We also deleted the HSF2 lanes in Fig S10D (see below).

I hope that we have met most of your requirements and thank you very much for your patience,
