# Supplementary material for: Heat shock factor 2 is a stress-responsive mediator of neuronal migration defects in models of fetal alcohol syndrome
Source: EMBO Mol Med. 2014 Jul 15;6(8):1043–61. doi: 10.15252/emmm.201303311 (PMC4154132; doi:10.15252/emmm.201303311)

Source data Figure 3A & B El Fatimy et al

Raw data WB Fig 3A upper panel

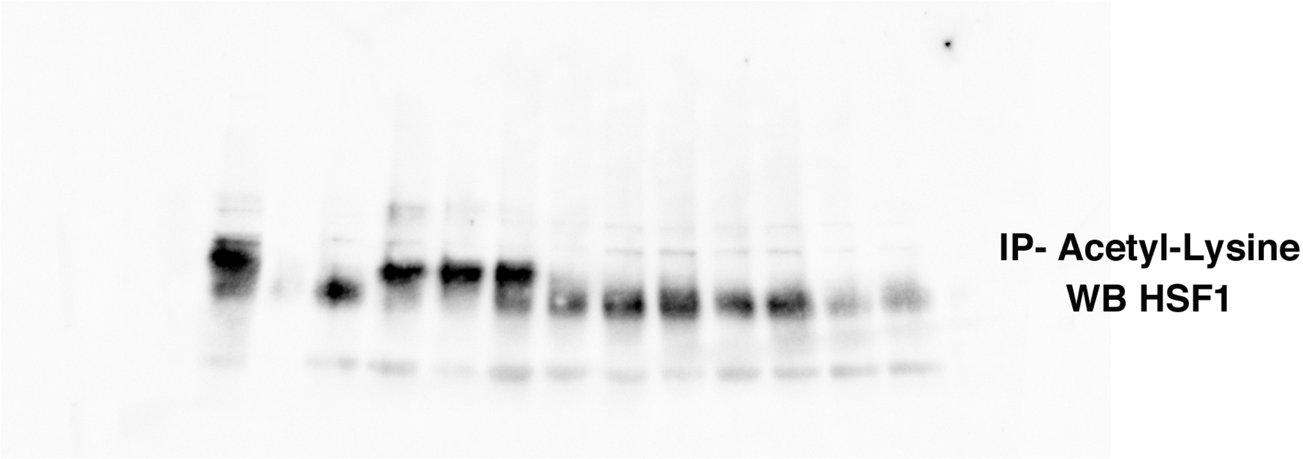

IP- Acetyl-Lysine
WB HSF1

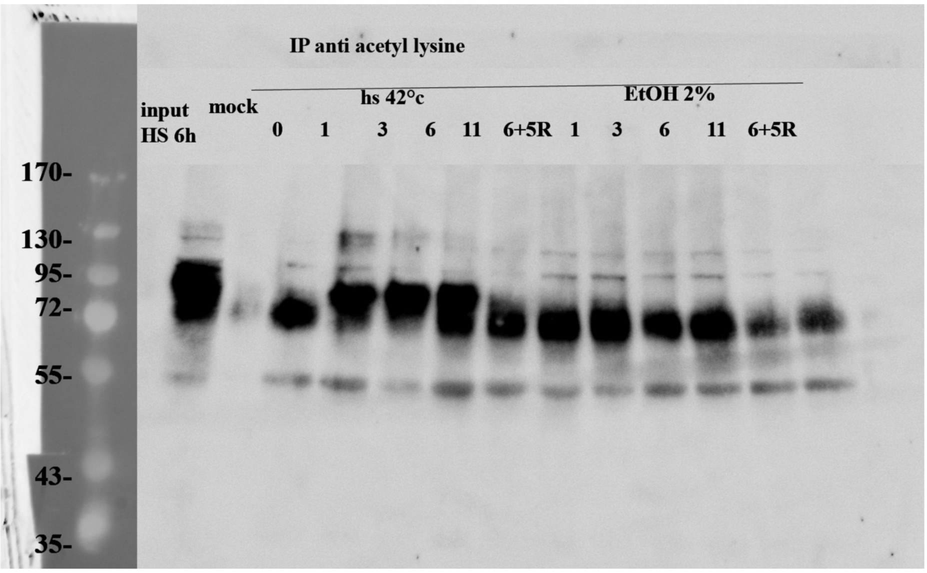

idem
(longer exposure
+ MW)

Raw data WB Fig 3A middle panel

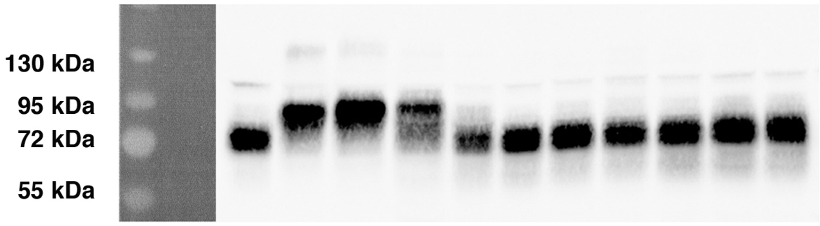

Whole Cell Extract
WB total HSF1

Raw data WB Fig 3B

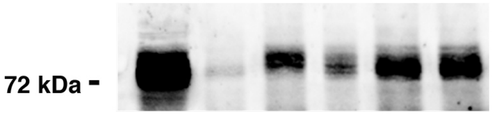

IP- Acetyl-Lysine
WB HSF1

Source data Figure 3C & D El Fatimy et al

Raw data WB HSF1 Fig 3C upper panel

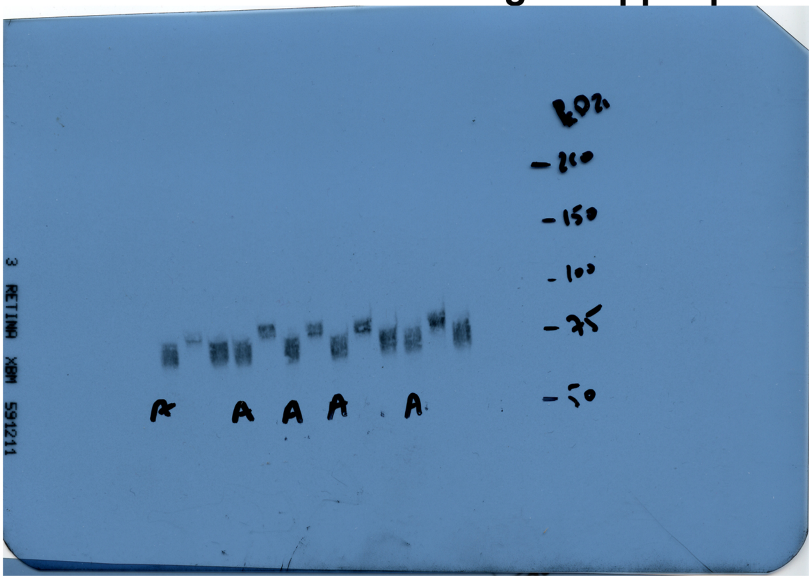

(idem with lane indications)

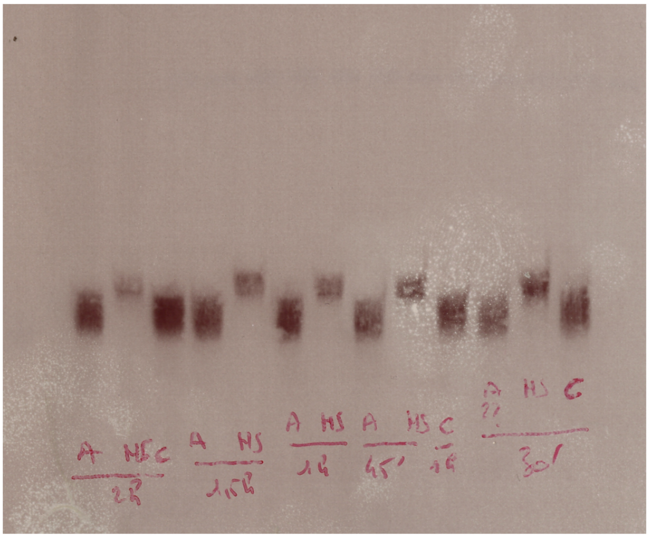

Raw data WB HSC70 Fig 3C lower panel

250 kDa

150 kDa

100 kDa

75 kDa

50 kDa

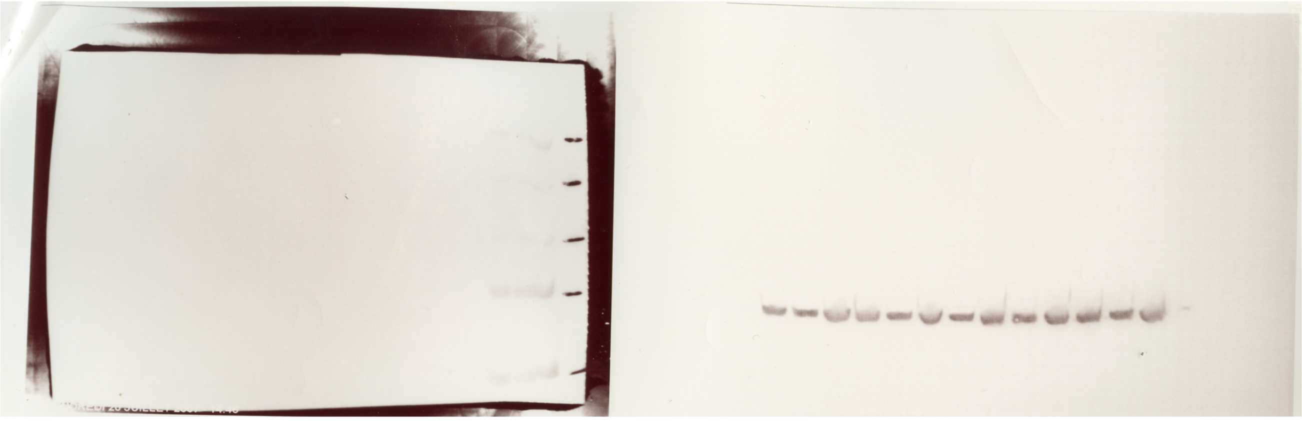

Raw data WB SUMO Fig 3D upper panel

S NI Co CAI CoCAICAI Co

175 kDA

IP SUMO

WB HSF1

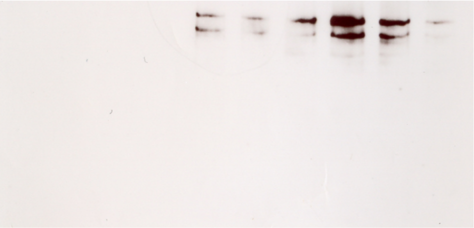

Raw data WB HSF1 Fig 3D lower panel

S NI Co CAI CoCAICAI Co

72 kDa

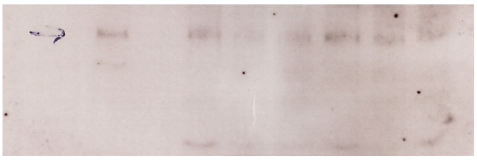

WB HSF1

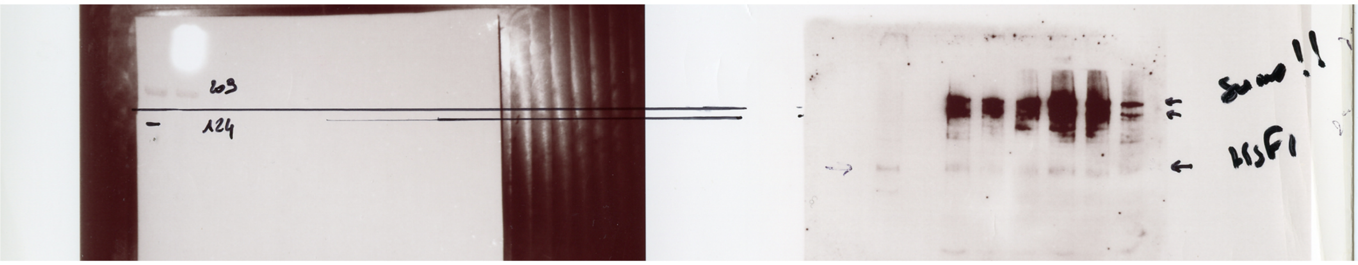

same WB HSF1 lower exposure with MW

Source data Figure 3 E El Fatimy et al

Raw data WB Fig 3E upper panel

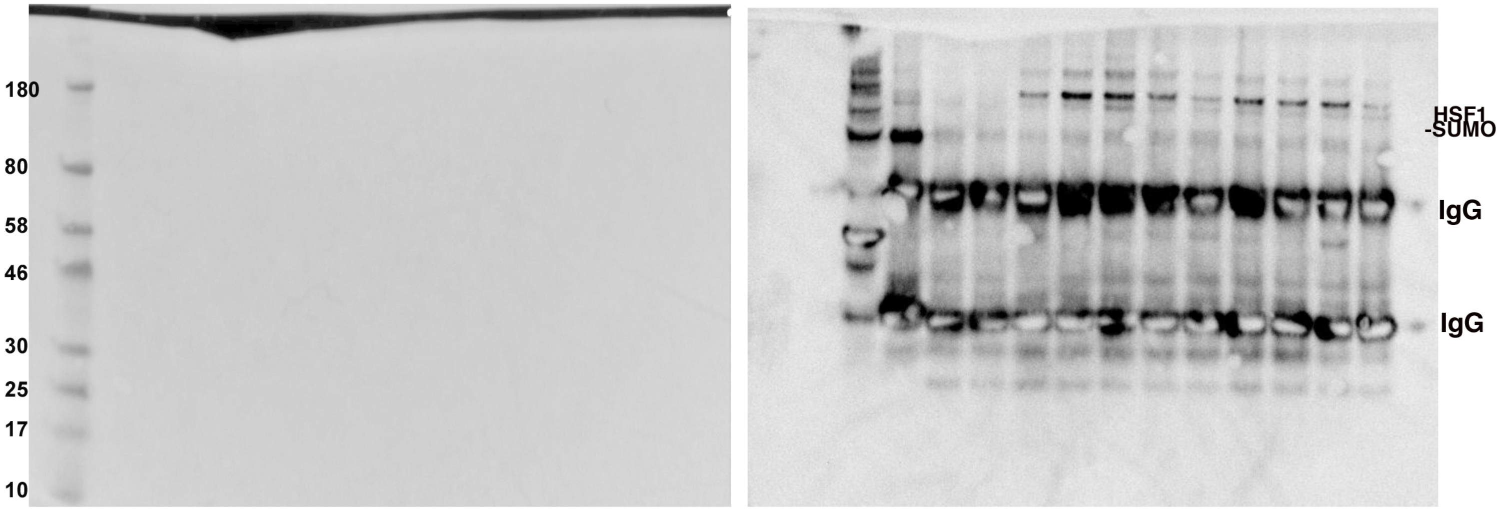

Raw data WB Fig 3E lower panel

short exposure

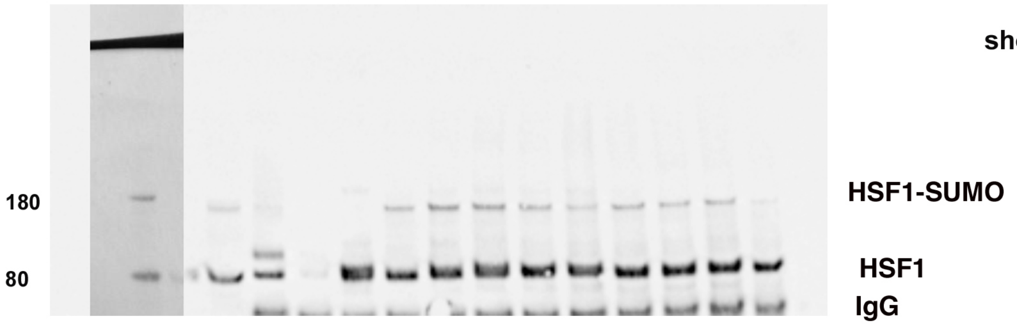

long exposure with total MW

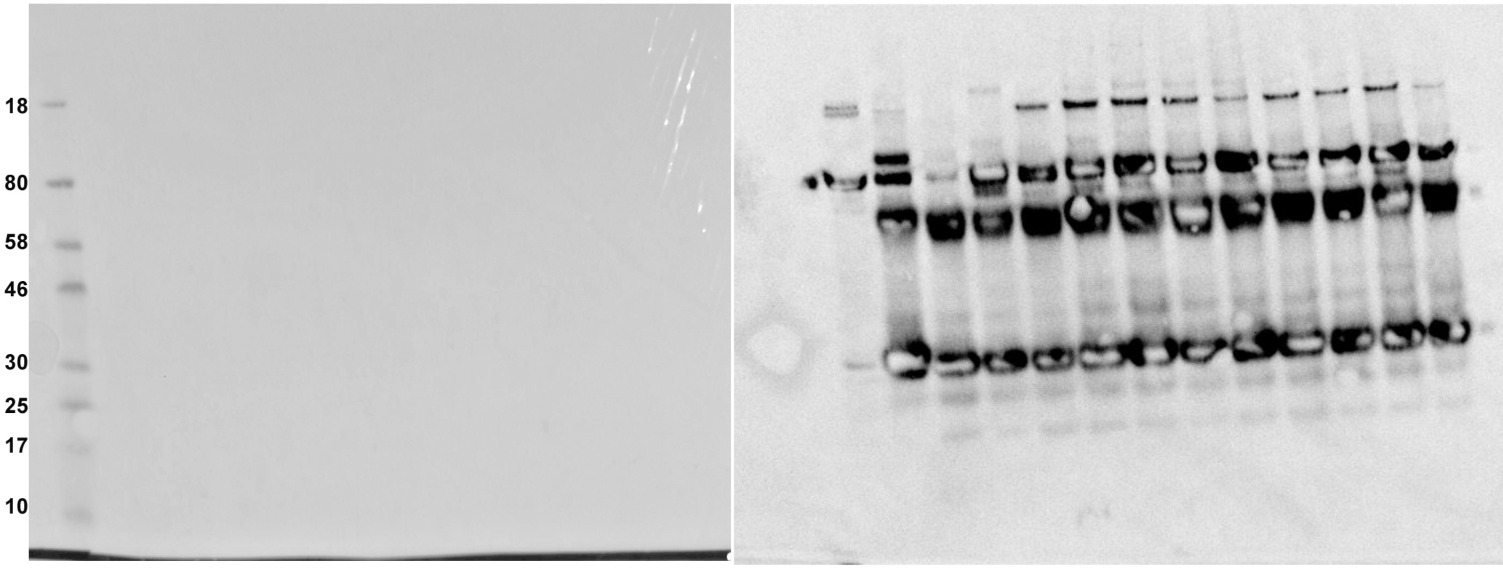

Supplement: Supplementary file 12 [file emmm0006-1043-sd12.pdf]
