# Supplementary material for: Heat shock factor 2 is a stress-responsive mediator of neuronal migration defects in models of fetal alcohol syndrome
Source: EMBO Mol Med. 2014 Jul 15;6(8):1043–61. doi: 10.15252/emmm.201303311 (PMC4154132; doi:10.15252/emmm.201303311)
Supplement: Supplementary file 13 [file emmm0006-1043-sd13.pdf]

Source data Fig 4B El Fatimy et al

Raw data EMSA gel (lanes of interest within frame) Fig 4B

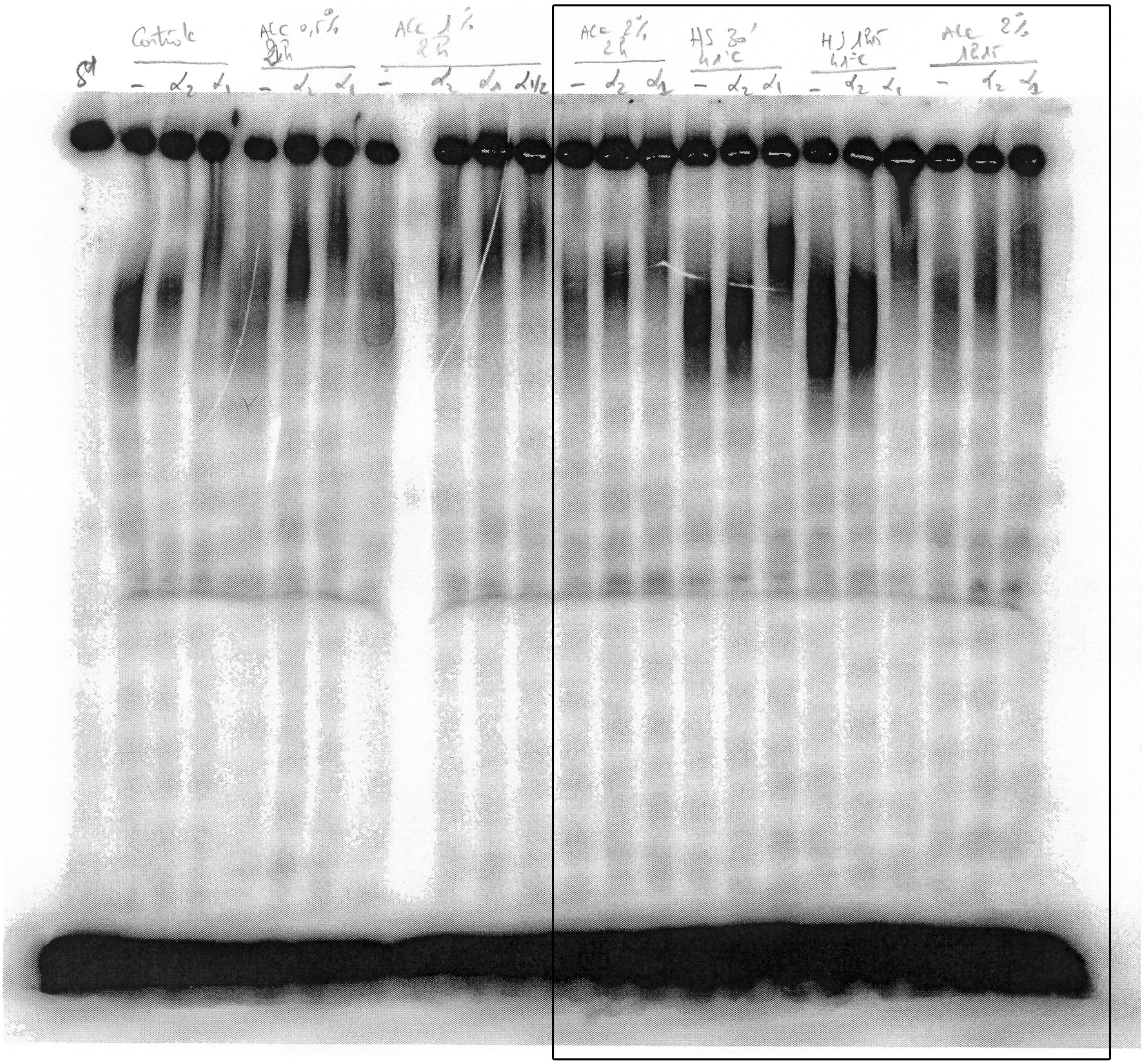

(PhosphoImager file)

Source data Fig 4D El Fatimy et al p.1

Raw data WB HSF1 CTR

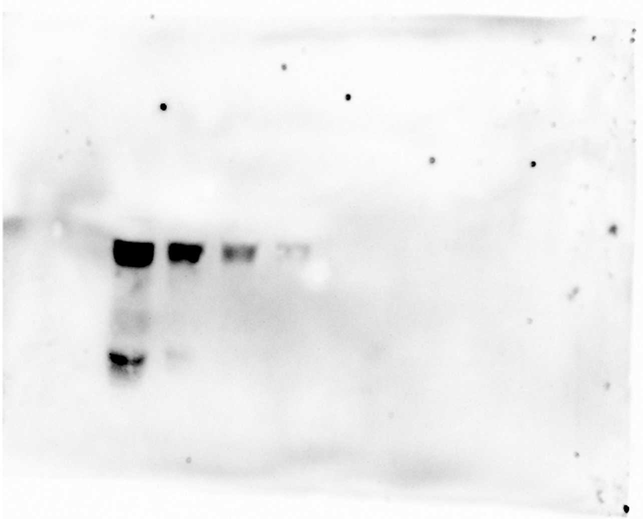

same membrane MW

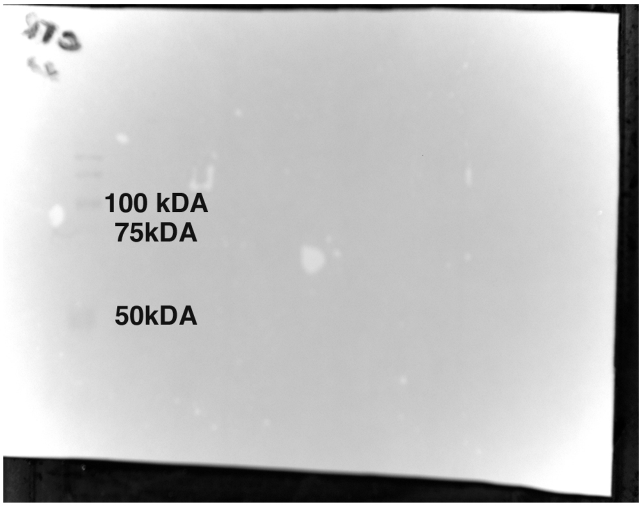

Raw data WB HSF1 EtOH

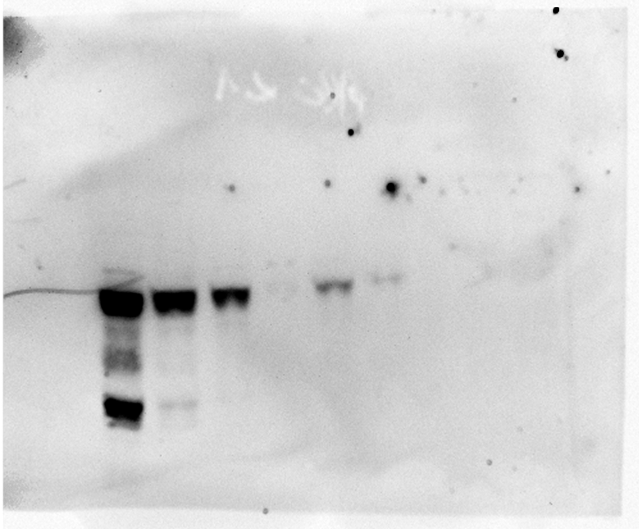

same membrane MW

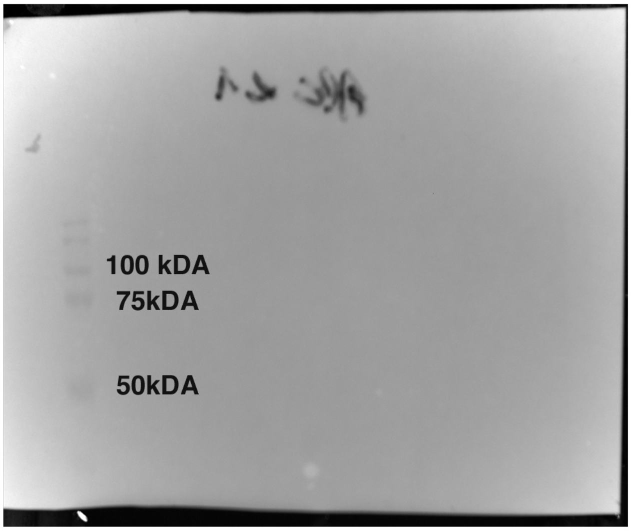

Raw data WB HSF1 HS

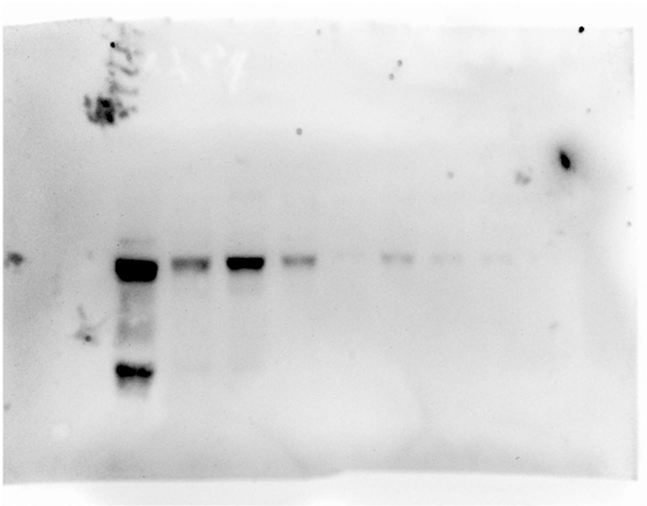

same membrane MW

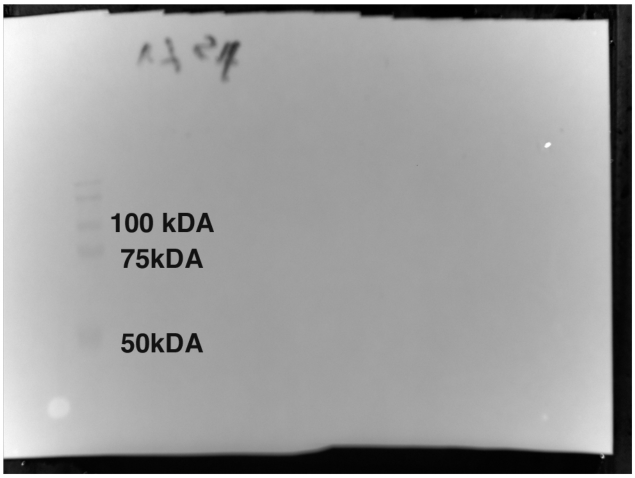

Source data Fig 4D El Fatimy et al p.2

Raw data WB HSF2 CTR

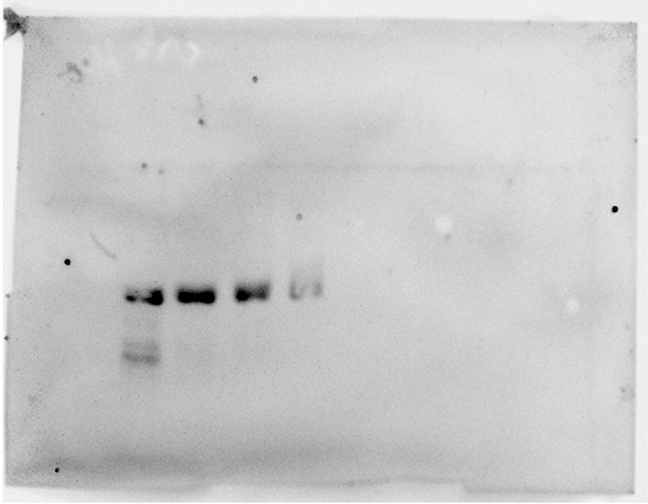

same membrane with MW

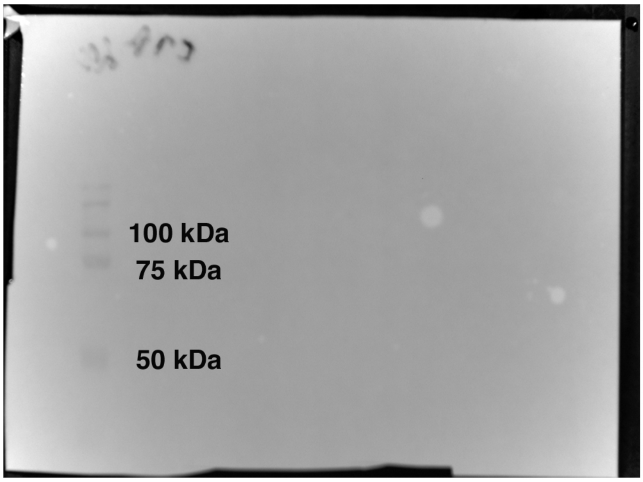

Raw data WB HSF2 EtOH

100 kDa
75 kDa
50 kDa

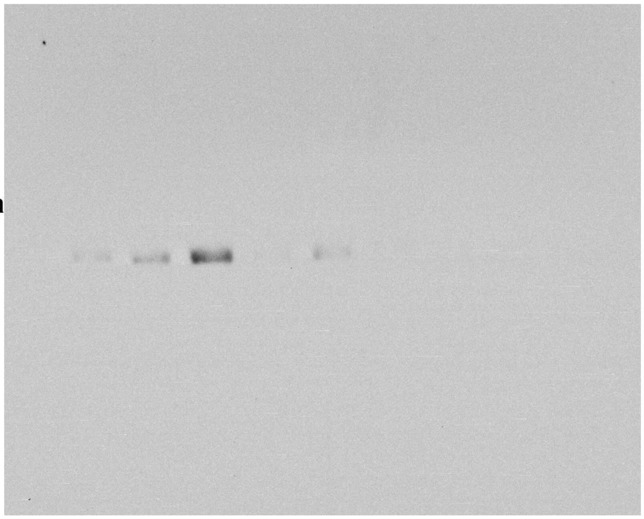

Gel run in parallel with the WB HSF2 CTR

Raw data WB HSF2 HS

100 kDa
75 kDa
50 kDa

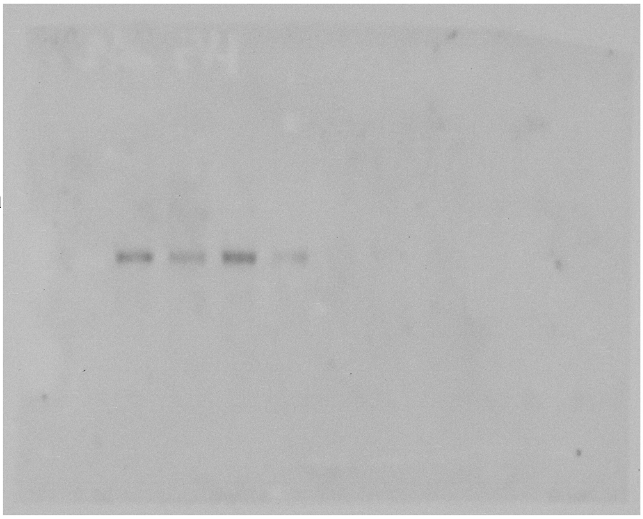

Gel run in parallel with the WB HSF2 CTR
