# Supplementary material for: Heat shock factor 2 is a stress-responsive mediator of neuronal migration defects in models of fetal alcohol syndrome
Source: EMBO Mol Med. 2014 Jul 15;6(8):1043–61. doi: 10.15252/emmm.201303311 (PMC4154132; doi:10.15252/emmm.201303311)

Source Data Fig 5 A El Fatimy et al

Raw data (PhosphoImager File) EMSA Fig 5A

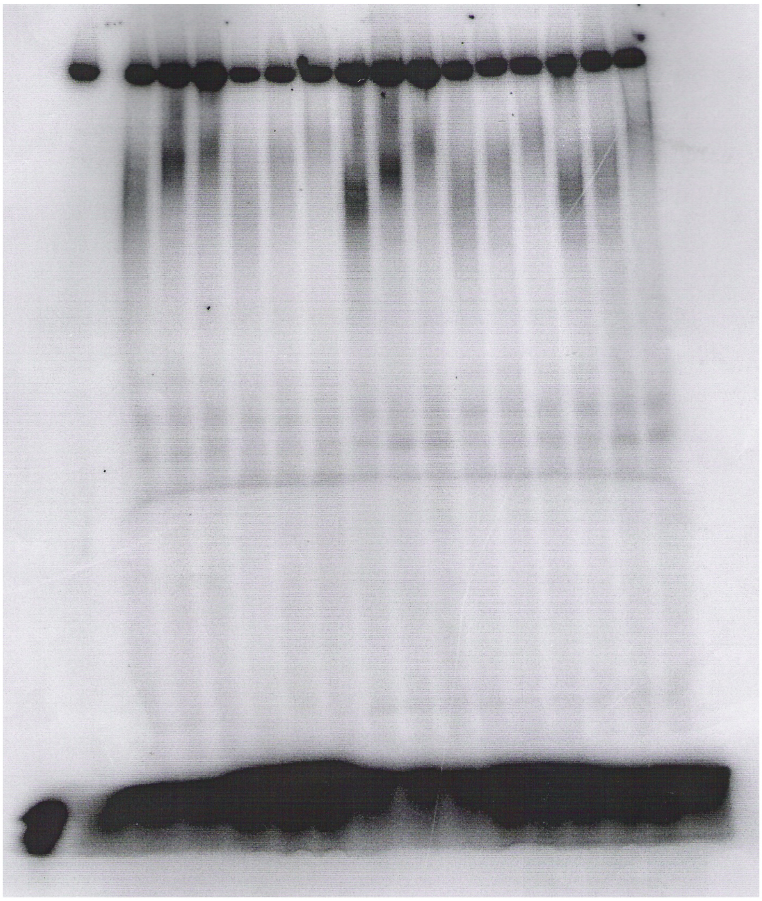

Source Data Fig 5 C El Fatimy et al

Raw data WB HSF1 Fig 5C (upper panel)

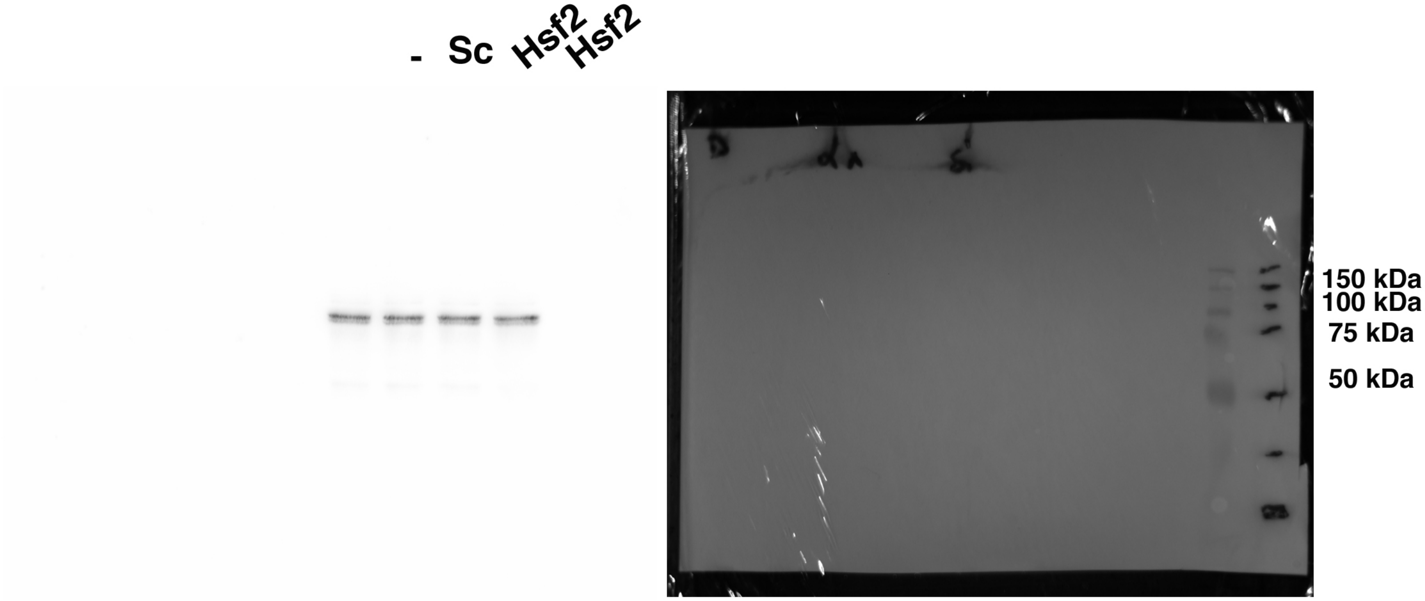

Raw data WB HSF2 Fig 5C,(middle panel)

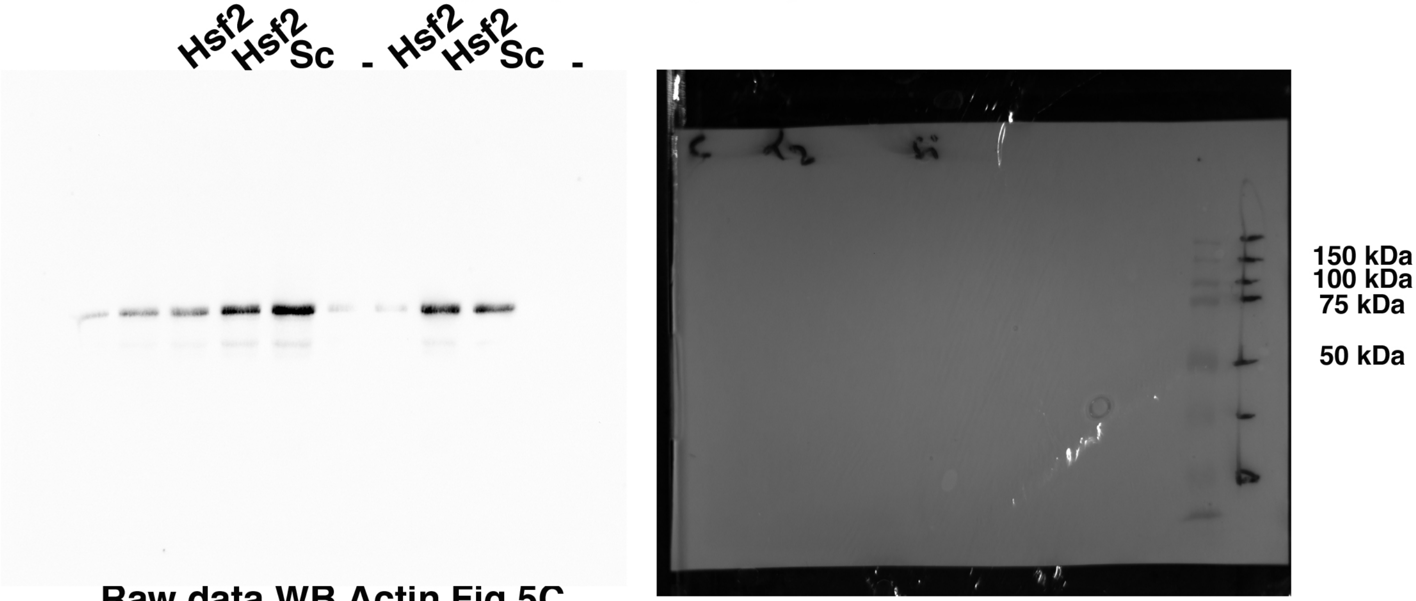

Raw data WB Actin Fig 5C

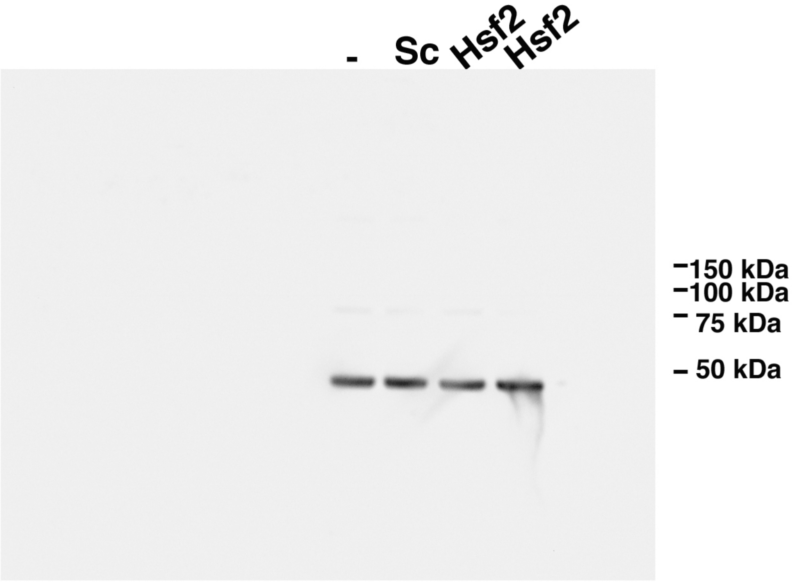

Raw data EMSA gel (lanes of interest within frame)

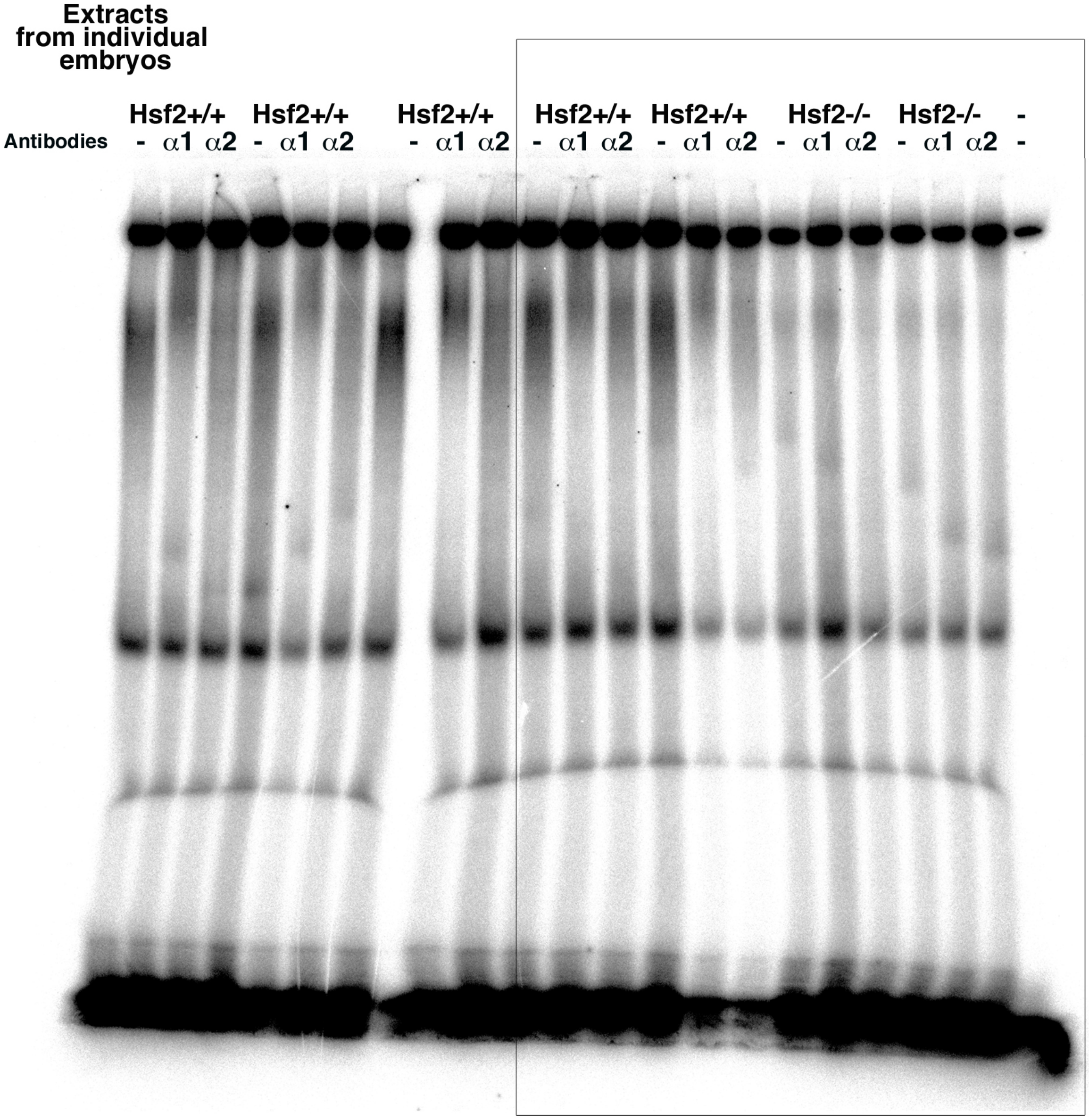

Source Fig 5 F El Fatimy et al.

Raw data for WB HSF1 (within frame) (upper panel)

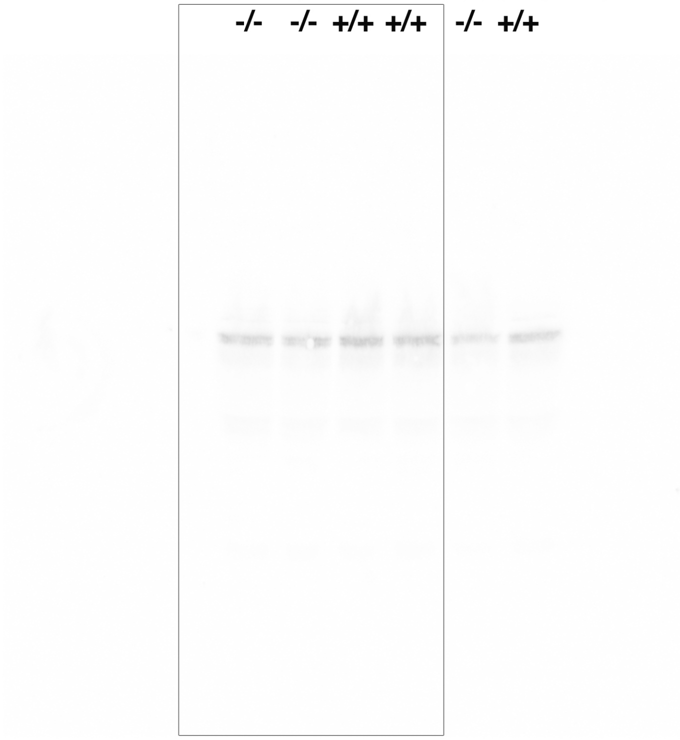

same membrane with MW

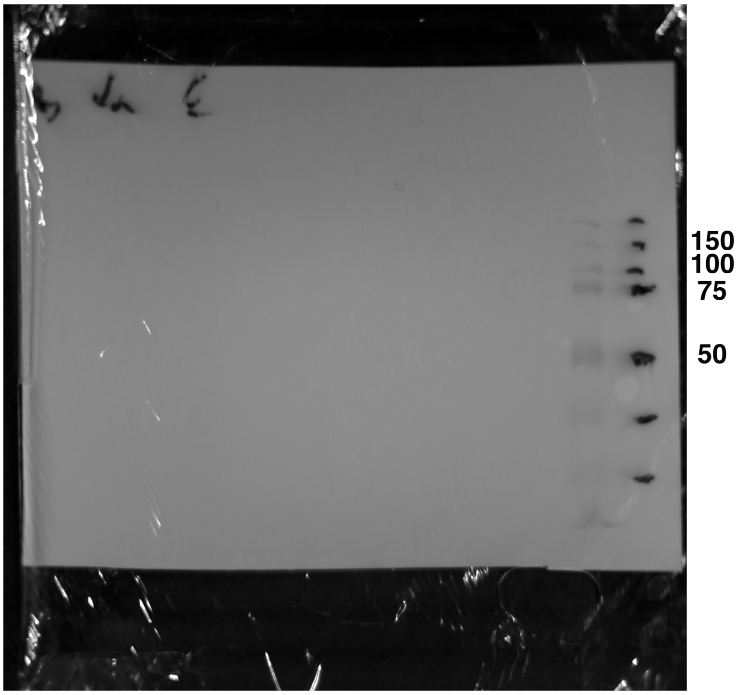

Raw data for WB HSF2 (within frame) (middle panel)

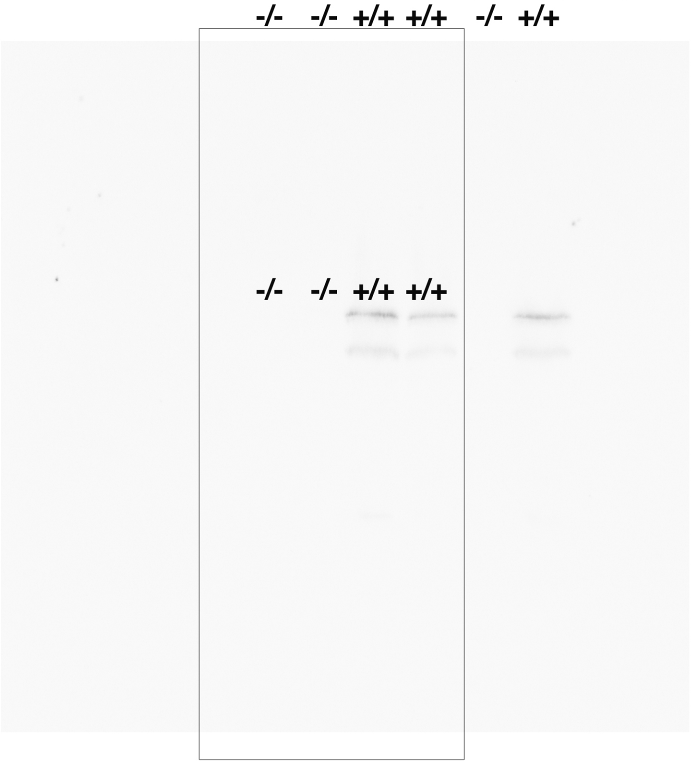

same membrane with MW

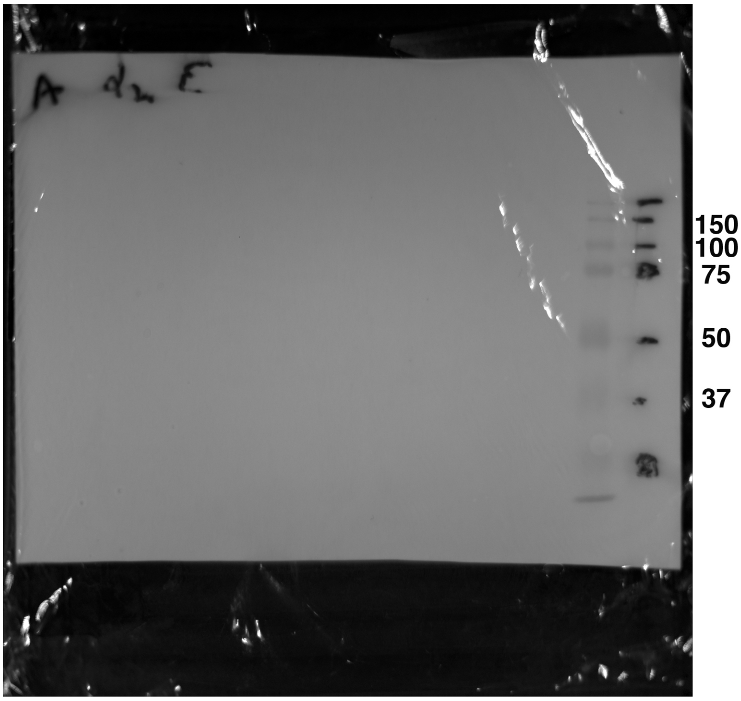

Raw data for WB Actin (lower panel)

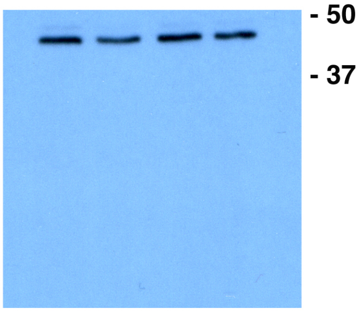

Supplement: Supplementary file 14 [file emmm0006-1043-sd14.pdf]
